# New 3-(Dibenzyloxyphosphoryl)isoxazolidine Conjugates of N1-Benzylated Quinazoline-2,4-diones as Potential Cytotoxic Agents against Cancer Cell Lines

**DOI:** 10.3390/molecules29133050

**Published:** 2024-06-27

**Authors:** Magdalena Łysakowska, Iwona E. Głowacka, Ewelina Honkisz-Orzechowska, Jadwiga Handzlik, Dorota G. Piotrowska

**Affiliations:** 1 Bioorganic Chemistry Laboratory, Faculty of Pharmacy, Medical University of Lodz, Muszynskiego 1, 90-151 Lodz, Poland; magdalena.lysakowska@umed.lodz.pl (M.Ł.); iwona.glowacka@umed.lodz.pl (I.E.G.); 2 Department of Technology and Biotechnology of Drugs, Faculty of Pharmacy, Jagiellonian University Medical College, Medyczna 9, 30-688 Krakow, Poland; ewelina.honkisz@uj.edu.pl (E.H.-O.); j.handzlik@uj.edu.pl (J.H.)

**Keywords:** *cis/trans*-isoxazolidines, quinazoline-2,4-diones, phosphonates, anticancer activity, apoptosis, safety

## Abstract

In this study, a new series of *cis* and *trans* 5-substituted-3-(dibenzyloxyphosphoryl)isoxazolidines **16a**–**g** were synthesized by the 1,3-dipolar cycloaddition reaction of *N*-benzyl-*C*-(dibenzyloxyphosphoryl)nitrone and selected *N*^1^-allyl-*N*^3^-benzylquinazoline-2,4-diones. All the obtained *trans*-isoxazolidines **16a**–**g** and the samples enriched in respective *cis*-isomers were evaluated for anticancer activity against three tumor cell lines. All the tested compounds exhibited high activity against the prostate cancer cell line (PC-3). Isoxazolidines *trans*-**16a** and *trans*-**16b** and diastereoisomeric mixtures of isoxazolidines enriched in *cis*-isomer using HPLC, namely *cis*-**16a**/*trans*-**16a** (97:3) and *cis*-**16b**/*trans*-**16b** (90:10), showed the highest antiproliferative properties towards the PC-3 cell line (IC_50_ = 9.84 ± 3.69–12.67 ± 3.45 μM). For the most active compounds, induction apoptosis tests and an evaluation of toxicity were conducted. Isoxazolidine *trans*-**16b** showed the highest induction of apoptosis. Moreover, the most active compounds turned out safe in vitro as none affected the cell viability in the HEK293, HepG2, and HSF cellular models at all the tested concentrations. The results indicated isoxazolidine *trans*-**16b** as a promising new lead structure in the search for effective anticancer drugs.

## 1. Introduction

**Figure 1:**
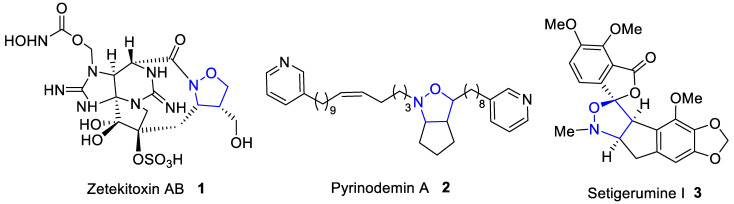
Structures of alkaloids with isoxazolidine ring.

Among various heterocyclic systems, isoxazolidine derivatives have numerous applications in organic synthesis and medicinal chemistry [1]. Compounds functionalized with an isoxazolidine moiety show a broad spectrum of biological activity, e.g., anticancer [2,3,4,5,6,7,8,9,10,11,12,13,14,15], antifungal [16,17,18], antibacterial [17,18,19,20,21], antiviral [2,10,22,23,24], antioxidant [25,26], anti-inflammatory [27], and antidiabetic [28,29]. Additionally, the isoxazolidine ring occurs in the structure of several alkaloids, e.g., **1**–**3** (Figure 1) isolated from various species of plants, aquatic invertebrates, and amphibians [30,31,32,33]. Among them, zetekitoxin AB (**1**) was found to be a potent blocker of voltage-dependent sodium channels expressed in *Xenopus* oocytes [30,31], while pyrinodemin A (**2**) isolated from the sea sponge *Amphimedon* was cytotoxic towards the murine leukemia cell line (L1210) (IC_50_ = 0.058 µg/mL) and the epidermoid carcinoma cell line (KB) (IC_50_ = 0.5 µg/mL) [32]. Although the biological activity of the trace racemic alkaloid Setigerumine I (**3**) naturally occurring in *Papaveraceae* family plants has not yet been investigated, studies on its biosynthetic availability have been undertaken [33].

**Figure 2:**
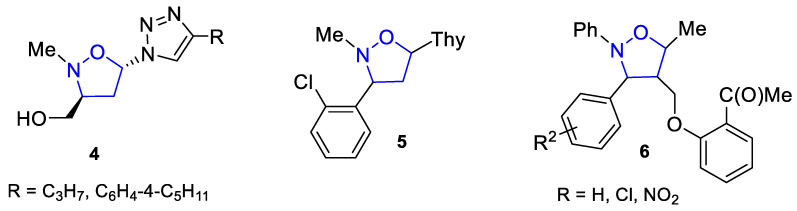
Structures of isoxazolidine analogs of nucleosides with anticancer activity.

**Figure 3:**
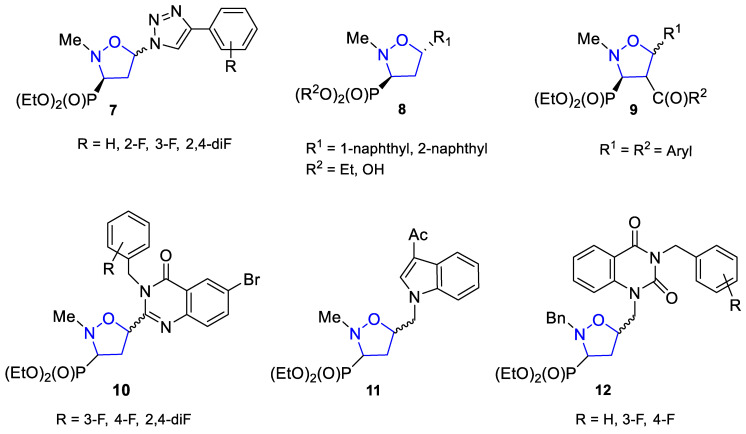
Structures of isoxazolidine analogs of nucleotides with anticancer activity.

The idea of the replacement of an isoxazolidine moiety by a furanose ring in nucleosides was first described by Tronchet and co-workers [34]. It resulted in the discovery of several isoxazolidine analogs of nucleosides and nucleotides exhibiting anticancer activity (Figure 2 and Figure 3). Cytotoxic activity of 1,2,3-triazole-isoxazolidine hybrids **4** (Figure 2) towards thyroid cancer cell lines (FTC-133) (IC_50_ = 3.87–3.95 µM) was recognized [4]. Compound **5** (Figure 2) showed an antitumor effect against the Jurkat cell line (IC_50_ = 8.8 ± 4.4 µM) [5]. Inhibitory properties of *N*-phenylisoxazolidines **6** (Figure 2) against colorectal adenoma cancer cell line growth (HT-29) (GI = 42–57%) were higher than those for the known anticancer drugs Mitomycin C (GI = 31%) and 5-Fluorouracil (GI = 34%), while the activity of **6** towards the breast cancer cell line was found to be (MCF-7) (GI = 26%), comparable to 5-Fluorouracil (GI = 31%) [6].

Among isoxazolidine analogs of nucleotides, compounds of general formula **7** (Figure 3) revealed cytotoxic activity against the human lung fibroblast cell line (HEL) (IC_50_ = 40.0–43.0 µM) [7]. 5-Naphthylisoxazolidines **8** (Figure 3) induced apoptosis in the HeLa and K562 cell lines with IC_50_ values of 0.05–0.2 mM and 0.03–0.2 mM for the HeLa and K562 cell lines, respectively [8]. A similar cytotoxicity of 3-(diethoxyphosphoryl)isoxazolidines **9** (Figure 3) towards K562 was also observed (IC_50_ = 0.07–0.09 mM) [9]. On the other hand, *C*-nucleotides **10** (Figure 3) exhibited anticancer activity against the human lymphocyte cell line (CEM) (IC_50_ = 9.6 ± 2.2–10.0 ± 0 µM), higher than 5-Fluorouracil used as the reference drug (IC_50_ = 18.0 ± 5.0 µM) [10]. Homonucleotides **11** and **12** (Figure 3) containing a methylene group incorporated between a modified nucleobase and isoxazolidine ring were found to be active anticancer compounds. Isoxazolidines **11** inhibited the proliferation of the murine leukemia cell line (L1210) at an IC_50_ = 33 ± 3.5 µM [3], whereas nucleotides **12** with a functionalized quinazoline-2,4-dione moiety as a false nucleobase were cytotoxic towards the CEM cell line (IC_50_ = 10.0 ± 6.0–17.0 ± 3.0 µM) [2].

**Figure 4:**
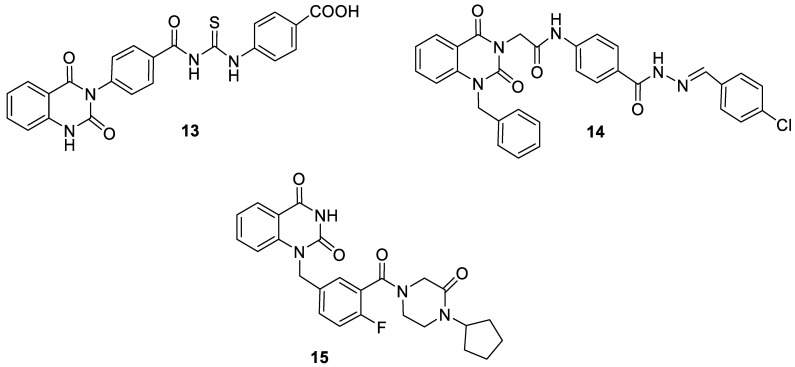
Structures of quinazoline-2,4-diones with anticancer activity.

Incorporation of the quinazoline-2,4-dione motif into the structure of compounds with the desired pharmacological effect is fully justified by the current knowledge about a wide range of biological functions of various derivatives within this class of compounds [35]. Taking into account the intention to achieve an optimal cytotoxic effect on cancer cells, several quinazoline-2,4-dione derivatives with well-documented anticancer properties should be mentioned [36,37,38]. And thus, **13** (Figure 4) exhibited promising cytotoxic activity against HCT-116 (IC_50_ = 1.184 ± 0.06 µM) and was found to be more active than the cabozantinib used as the positive control (IC_50_ = 16.35 ± 0.86 µM) [36]. While derivative **14** functionalized at both the N1 and N3 position of quinazoline-2,4-dione (Figure 4) showed significant antiproliferative activities against three cancer cell lines, namely, HepG2, HCT-116, and MCF-7 (GI_50_ = 9.16 ± 0.8, 5.69 ± 0.4, and 5.27 ± 0.2 µM, respectively) [37], the N1-monosubstituted quinazoline-2,4-dione **15** (Figure 4) inhibited both PARP1 and 2 (poly(ADP-ribose)polymerase 1 and 2) [38].

**Scheme 1:**
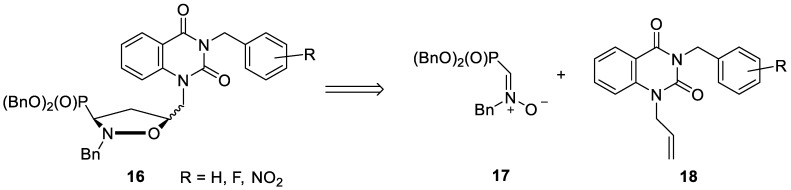
Retrosynthesis of isoxazolidine conjugates of quinazoline-2,4-dione **16**.

In continuation of our search for isoxazolidine analogs of homonucleotides **12** with anticancer activity and following the concept of combining two or more pharmacophores into one compound commonly applied in medicinal chemistry, we designed a new series of compounds of the general formula **16** (Scheme 1). The replacement of the diethoxyphosphoryl function with the dibenzyloxyphosphoryl group in the designed isoxazolidines **16** would result in obtaining compounds with better permeability through the cellular membrane [39]. The strategy relied on the application of the 1,3-dipolar cycloaddition of *N*-benzyl-*C*-(dibenzyloxyphosphoryl)nitrone **17** [40] with selected *N*^1^-allyl-*N*^3^-benzylquinazoline-2,4-diones **18** [2] as a key step in the synthesis of the designed series of homonucleotides **16** (Scheme 1).

## 2. Results and Discussion

### 2.1. Chemistry

**Scheme 2:**
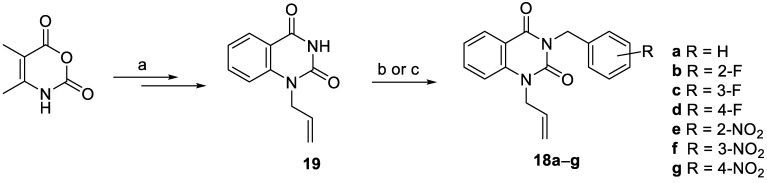
Reaction and conditions: (a) details for the preparation of *N*^1^-allylquinazoline-2,4-dione **19** given in [2]; (b) respective benzyl bromide KOH, MeCN, reflux, 4h for the synthesis of **18a**–**d** [2]; (c) respective nitrobenzyl bromide, K_2_CO_3_, DMF, rt. 72 h, for the synthesis of **18e**–**g**.

The synthesis and full characteristics of nitrone **14** were recently reported [40]. The known *N*^3^-benzylated *N*^1^-allylquinazoline-2,4-diones **15a**–**d** were prepared from isatoic anhydride according to the methodology described previously [2]. The analogous reaction sequence was also applied for the preparation of *N*^3^-nitrobenzyl derivatives **15e**–**g** (Scheme 2).

**Scheme 3:**
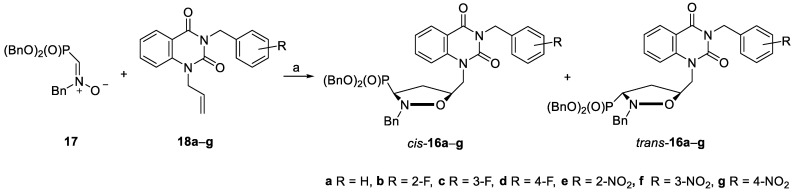
Reaction and conditions: (a) toluene, 60 °C, 5 days.

The cycloaddition of *N*-benzyl-*C*-(dibenzyloxyphosphoryl)nitrone [40] **17** and respective *N*^1^-allyl-*N*^3^-benzylquinazoline-2,4-diones **18a**–**g** was carried out using the reaction conditions established before for analogous isoxazolidines **12** [2] and proceeded slowly at 60 °C for 5 days in anhydrous toluene. The corresponding mixtures of diastereomeric 3-(dibenzyloxyphosphoryl)isoxazolidines *cis*-**16a**–**g** and *trans*-**16a**–**g** with *trans*-isomer predominating were obtained (Scheme 3, Table 1). The diastereomeric ratios of *cis*-**16a**–**g** and *trans*-**16a**–**g** were determined by comparing the integrals of the appropriate signals in the ^31^P NMR spectra taken for the crude reaction mixtures (Supplementary Materials, Figures S7–S13). The reactions proceeded with low to moderate diastereoselectivities (d.e. 16–34%) and in good yields (61–69%). The reaction mixtures were purified on a silica gel column followed by HPLC; however, only pure major isomers *trans*-**16a**–**g** were isolated. Although attempts at the additional chromatographic separation of the enriched fractions were undertaken, they have not resulted in the isolation of pure isomeric isoxazolidines *cis*-**16a**–**g**. For this reason, for further configurational studies as well as biological assays, samples of pure *trans*-isomers *trans*-**16a**–**g** and the selected fractions significantly enriched for minor *cis*-**16a**–**g** were used.

**Figure 5:**
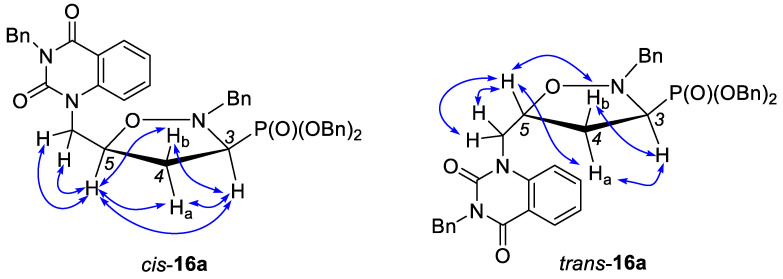
Observed NOEs for *cis*-**16a** and *trans*-**16a**.

The relative configurations of *cis*- and *trans*-isoxazolidines **16a**–**g** were assigned based on 2D NOE experiments performed for *cis*-**16a** and *trans*-**16a** (Figure 5). For the isoxazolidine *cis*-**16a**, in addition to the expected NOESY signals between *H*C5 and both protons at *H*_2_C4 and *H*_2_C protons attached to the quinazoline-2,4-dione unit, a diagnostic signal between *H*C5 and *H*C3 was observed, which unequivocally proves the *cis* orientation of the substituents at C3 and C5. On the other hand, since the correlation between *H*C5 and *H*C3 was not observed in the NOESY spectrum taken for *trans*-**16a**, *trans* orientation between the substituents at C3 and C5 was deduced for this diastereoisomer. A slight modification of the substituents at N3 of the quinazoline-2,4-dione moiety has no influence on the stereochemical outcome of the cycloaddition; therefore, the configuration of all major isoxazolidines **16** was assigned as *trans*, whereas minor ones were identified as respective *cis*-isomers.

### 2.2. Pharmacology

#### 2.2.1. Cytotoxicity towards Cancer Cell Lines

In this study, we applied the most extensively used MTS-based assay, which provides a valuable tool for determining cell viability by evaluating metabolic activity. The cytostatic activity of the tested compounds was defined as the half-maximal inhibitory concentration (IC_50_) causing a 50% decrease in the metabolic activity of the cells. It was determined against breast cancer (MCF-7), fibrosarcoma (HT-1080), and prostate cancer (PC-3) cells.

The inhibitory effect of the synthesized isoxazolidine **16a**–**g** against the proliferation of tumor cell lines MCF-7, HT-1080, and PC-3 is shown in Table 2. All the tested compounds exhibited high activity against prostate cancer cells (PC-3) (IC_50_ = 9.84 ± 3.69 to 26.57 ± 4.69 μM). Among them, isoxazolidines *trans*-**16a** and *trans*-**16b**, as well as the respective diastereoisomeric mixtures of isoxazolidines *cis*-**16a**/*trans*-**16a** (97:3) and *cis*-**16b**/*trans*-**16b** (90:10), were the most active with IC_50_ values in the range of 9.84 ± 3.69 μM to 12.67 ± 3.45 μM. Moreover, from the entire series of compounds, the mixture of isoxazolidines *cis*-**16d**/*trans*-**16d** (96:4) appeared to have the highest inhibitory properties towards fibrosarcoma cell line growth (IC_50_ = 10.36 ± 2.69 μM). The investigated isoxazolidines exhibited the lowest activity against the MCF-7 cell line (Table 2).

#### 2.2.2. Mechanistic Studies: Induction of Apoptosis

**Figure 6:**
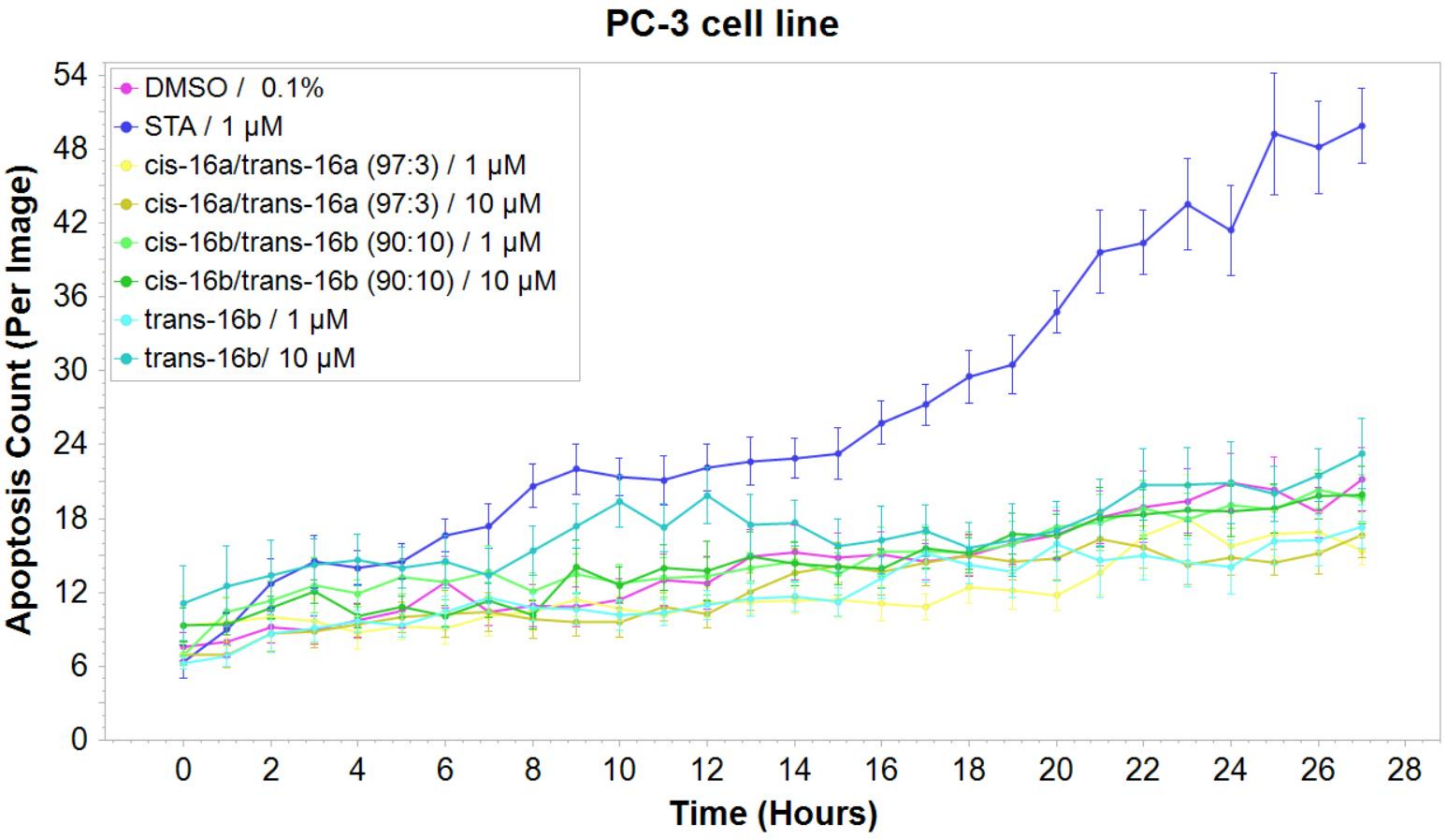
Induction of apoptosis by compounds: *cis*-**16a**/*trans*-**16a** (97:3), *cis*-**16b**/*trans*-**16b** (90:10), and *trans*-**16b**.

Apoptosis induction tests were conducted using the IncuCyte system to test cells (PC-3) in an in vitro culture. Analysis was performed for the isoxazolidines or respective mixtures of diastereoisomers, i.e., *cis*-**16a**/*trans*-**16a** (97:3), *cis*-**16b**/*trans*-**16b** (90:10), and *trans*-**16b** exhibiting the highest antiproliferative properties. The compounds were tested at 1 µM and 10 µM concentrations, and apoptosis was monitored for 28 h (Figure 6). The incubation time was selected based on the induction of apoptosis in the control cells. Staurosporine (STA) at a concentration of 1 µM was used as the positive control, while the negative control cells were treated with 0.1% DMSO (vehicle control). The most active compound was *trans*-**16b** at 10 μM. As shown in Figure 6, apoptosis induction was most pronounced up to 12 h and was comparable to that induced with staurosporine.

#### 2.2.3. Safety Studies In Vitro

**Figure 7:**
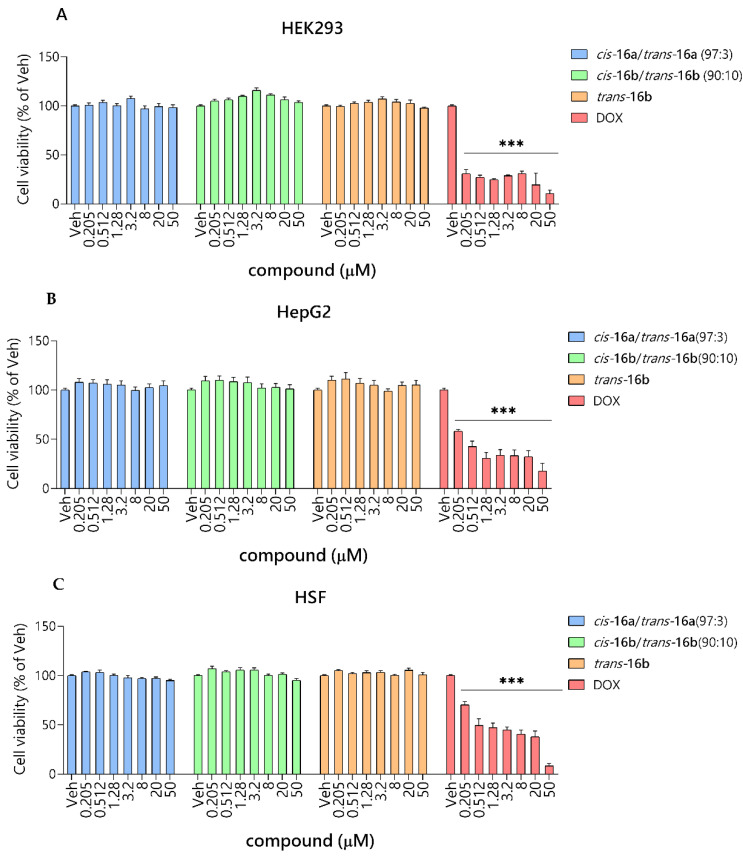
The cytotoxicity effect of *cis*-**16a**/*trans*-**16a** (97:3), *cis*-**16b**/*trans*-**16b** (90:10), *trans*-**16b,** and doxorubicin (DOX) on HEK293 (**A**), HepG2 (**B**), and HSF (**C**) cells after 72 h. Each point represents the mean ± SEM of three independent experiments, each consisting of four replicates per treatment group (n = 12). Statistical analyses were performed using GraphPad Prism 8.0 software. Statistical significance was evaluated by one-way ANOVA with post hoc Dunnett test at significance level α = 0.05 (*** *p* < 0.001).

To preliminarily assess the toxicity parameters against liver, kidney, and human fibroblast cells, selected cell lines were treated with *cis*-**16a**/*trans*-**16a** (97:3), *cis*-**16b**/*trans*-**16b** (90:10), and *trans*-**16b** in a wide range of concentrations (0.205–50 μM). The results are presented in Figure 7A–C. In both cellular models (HEK293 and HepG2), all the tested compounds showed excellent safety profiles, and the range of concentrations tested did not affect cell viability. Similarly, no adverse effect was observed in HSF. In comparison, DOX in the same concentration range exerted a significant cytotoxic effect on each cell type.

#### 2.2.4. ADMET Studies In Silico

In order to predict potential “drug-likeness” for the investigated compounds (**16a**–**g**), the comprehensive in silico ADMET simulation, with doxorubicin (DOX) as the reference drug, was performed using the bioinformatic tool pkCSM (https://biosig.lab.uq.edu.au/pkcsm/prediction, accessed on 8 June 2024) recommended in [41]. Detailed data are provided in the Supplementary Materials (Tables S1–S15). The corresponding diastereomers were drawn and calculated separately. However, the results indicated that the above-mentioned software did not discriminate configuration-dependent changes in the properties predicted for the respective diastereoisomers, and only assessed the impact of the presence of the respective substituents in the benzyl group located at N3 in the quinazoline-2,4-dione moiety on the ADMET profile of the obtained isoxazolidine **16**. The results of the simulation indicate the probability of a lower than that of DOX water solubility for the whole investigated compounds (**16a**–**g**) as well as their low volume of distribution (VDss). The unbound fraction was predicted in the range of 0.272 (for **16a**) to 0.315 (for **16e**), i.e., higher than that of DOX (0.232). An excellent intestine absorption (100%) can be expected for all the tested compounds **16a**–**g**, in this respect, making them clearly superior to doxorubicin. All **16a**–**g** and DOX displayed relatively low skin permeability in the examination in silico, while the Caco-2 permeability predicted indicates a better absorption of compounds **16a**–**g** (0.541–0.766) than doxorubicin (0.152), although in both cases, the compounds did not reach values classifying them as highly permeable (>0.9). Based on the pkCSM calculation, both the investigated series and DOX displayed a low ability to penetrate BBB and to reach CNS. Additionally, DOX is predicted as a substrate of the BBB transporter, Pgp, while all the tested compounds (**16a**–**g**) as Pgp inhibitors. In contrast to DOX, the tested series is characterized by the risk of undesirable proarrhythmic effects related to hERG inhibition, and drug–drug interactions due to probable action on the isoforms of cytochrome P-450, i.e., as substrates for CYP3A4, and inhibitors for CYP2C9 (**16a**–**d**, **16f**, **16g**) and CYP2C19 (**16a**–**d**).

In similarity to DOX, the whole series showed neither signs of a renal OCT substrate nor the risk of skin sensitivity. Superior to DOX, no compound (**16a**–**g**) displayed a risk of mutagenicity, and compounds **16e** and **16f** in accordance with DOX did not demonstrate hepatotoxic risk in the simulation. It is worth remembering that the results of our studies in vitro also excluded the hepatotoxicity risk for compounds **16a** and **16b**. The simulation with the pkCSM bioinformatic tool referring to several animal models suggests probable lower toxic doses for **16a**–**g** than for DOX, and the maximal tolerated dose recommended in humans, 0.42–0.51 and 0.654, for compounds **16a**–**g** and DOX, respectively.

## 3. Materials and Methods

### 3.1. General Information

^1^H, ^13^C, and ^31^P NMR spectra were taken in CDCl_3_ on the Bruker Avance III spectrometers (600 MHz, Bruker Instruments, Karlsruhe, Germany) with TMS as the internal standard at 600, 151, and 243 MHz, respectively. ^1^H–^1^H COSY and NOESY experiments were applied, when necessary, to support spectroscopic assignments. IR spectra were measured on an Infinity MI-60 FT-IR spectrometer (Bruker Optik GmbH, Ettlingen, Germany). Melting points were determined on a Boetius apparatus and were uncorrected. Elemental analyses were performed by the Microanalytical Laboratory of this Faculty on the Perkin-Elmer PE 2400 CHNS analyzer (Perkin Elmer Corp., Norwalk, CT, USA). The following adsorbents were used: column chromatography, Merck silica gel 60 (70–230 mesh); analytical TLC, Merck TLC plastic sheets silica gel 60 F_254_. HPLC separations were performed using a Waters HPLC system (Waters Corporation, Milford, MA, USA) consisting of a binary HPLC pump (Waters 2545), a diode array detector (Waters 2998), an autosampler (Waters 2767), and an XBridge C18 column OBD, 19 × 100 mm with a particle size of 5 µm.

*N*^1^-Allyl-*N*^3^-(fluorobenzyl)quinazoline-2,4(1*H*,3*H*)-diones **18a**–**d** [2] and *N*-benzyl-*C*-(dibenzyloxyphosphoryl)nitrone **17** [40] were obtained according to the literature.

^31^P-NMR spectra of raw materials and ^1^H-, ^13^C-, and ^31^P-NMR spectra and analytical chromatograms of all newly synthesized compounds are provided in Supplementary Materials (Figures S1–S73).

### 3.2. General Procedure for the Preparation of Quinazoline-2,4-Diones ***18e**–**g***

To a suspension of *N*^1^-allylquinazoline-2,4-dione **19** (1.00 mmol) in dry DMF (5 mL), potassium carbonate (1.20 mmol) was added followed by the respective nitrobenzyl bromide (1.50 mmol). The reaction mixture was stirred at room temperature for 72 h and co-evaporated several times with toluene. The residue was dissolved in methylene chloride (10 mL) and washed with water (3 × 10 mL). The organic phase was dried over MgSO_4_, filtered, and concentrated. The crude product was chromatographed on a silica gel column with methylene chloride–hexane mixture (1:1, *v*/*v*) and methylene chloride and crystallized from a methylene chloride–petroleum ether mixture.

N^1^-Allyl-N^3^-(2-nitrobenzyl)quinazoline-2,4(1*H*,3*H*)-dione (**18e**). According to the general procedure from *N*^1^-allylquinazoline-2,4-dione **19** (0.400 g, 1.98 mmol), potassium carbonate (0.328g, 2.38 mmol), and 2-nitrobenzyl bromide (0.642 g, 2.97 mmol), *N*^1^-allyl-*N*^3^-(2-nitrobenzyl)quinazoline-2,4-dione **18e** (0.370 g, 55%) was obtained as a white amorphous solid, m.p. = 126–128 °C. IR (KBr, cm^–1^) ν_max_: 3081, 2851, 1708, 1652, 1526, 1483, 1412, 1336, 977, 763. ^1^H NMR (600 MHz, CDCl_3_): δ = 8.29 (dd, *J* = 7.9 Hz, *J* = 1.6 Hz, 1H), 8.10 (dd, *J* = 8.2 Hz, *J* = 1.1 Hz, 1H), 7.72 (dt, *J* = 7.3 Hz, *J* = 1.6 Hz, 1H), 7.55–7.52 (m, 1H), 7.45–7.42 (m, 1H), 7.32 (t, *J* = 7.9 Hz, 1H), 7.26 (t, *J* = 8.0 Hz, 2H), 5.95 (ddt, ^3^*J* = 17.3 Hz, ^3^*J* = 10.5 Hz, ^3^*J* = 5.2 Hz, 1H, CH_2_–C*H*=CH_2_), 5.72 (s, 2H, C*H*_2_Ph), 5.31 (d, ^3^*J* = 10.5 Hz, 1H, CH_2_–CH=CH*H*), 5.24 (d, ^3^*J* = 17.3 Hz, 1H, CH_2_–CH=C*H*H), 4.83–4.82 (m, 2H, C*H*_2_–CH=CH_2_); ^13^C NMR (151 MHz, CDCl_3_): δ = 161.77 (C=O), 150.65 (C=O), 148.78, 139.98, 135.48, 133.52, 132.48, 131.07, 129.31, 128.02, 127.65, 125.11, 123.34, 117.86, 115.35, 114.42, 46.12, 42.28. Anal. calcd. For C_18_H_15_N_3_O_4_: C, 64.09; H, 4.48; N, 12.46. Found: C, 63.85; H, 4.18; N, 12.28.

*N*^1^-Allyl-*N*^3^-(3-nitrobenzyl)quinazoline-2,4(1*H*,3*H*)-dione (**18f**). According to the general procedure from *N*^1^-allylquinazoline-2,4-dione **19** (0.400 g, 1.98 mmol), potassium carbonate (0.328g, 2.38 mmol), and 3-nitrobenzyl bromide (0.642 g, 2.97 mmol), *N*^1^-allyl-*N*^3^-(3-nitrobenzyl)quinazoline-2,4-dione **18f** (0.493 g, 74%) was obtained as a white amorphous solid, m.p. = 139–141 °C. IR (KBr, cm^–1^) ν_max_: 3081, 2853, 1702, 1641, 1609, 1527, 1483, 1419, 1329, 974, 739, 694. ^1^H NMR (600 MHz, CDCl_3_): δ = 8.35–8.34 (m, 1H), 8.25 (dd, *J* = 7.9 Hz, *J* = 1.6 Hz, 1H), 8.13–8.12 (m, 1H), 7.85 (d, *J* = 7.6 Hz, 1H), 7.67 (dt, *J* = 7.3 Hz, *J* = 1.6 Hz, 1H), 7.49 (t, *J* = 8.0 Hz, 1H), 7.29–7.26 (m, 1H), 7.19 (d, *J* = 8.5 Hz, 1H), 5.93 (ddt, ^3^*J* = 17.2 Hz, ^3^*J* = 10.2 Hz, ^3^*J* = 4.9 Hz, 1H, CH_2_–C*H*=CH_2_), 5.37 (s, 2H, C*H*_2_Ph), 5.28 (d, ^3^*J* = 10.2 Hz, 1H, CH_2_–CH=CH*H*), 5.21 (d, ^3^*J* = 17.2 Hz, 1H, CH_2_–CH=C*H*H), 4.79–4.78 (m, 2H, C*H*_2_–CH=CH_2_); ^13^C NMR (151 MHz, CDCl_3_): δ = 161.73 (C=O), 150.72 (C=O), 148.37, 139.87, 138.94, 135.43, 135.13, 131.01, 129.42, 129.22, 123.82, 123.32, 122.75, 117.84, 115.43, 114.37, 46.16, 44.37. Anal. calcd. For C_18_H_15_N_3_O_4_: C, 64.09; H, 4.48; N, 12.46. Found: C, 63.80; H, 4.18; N, 12.17.

*N*^1^-Allyl-*N*^3^-(4-nitrobenzyl)quinazoline-2,4(1*H*,3*H*)-dione (**18g**). According to the general procedure from *N*^1^-allylquinazoline-2,4-dione **19** (0.400 g, 1.98 mmol), potassium carbonate (0.328g, 2.38 mmol), and 4-nitrobenzyl bromide (0.642 g, 2.97 mmol), *N*^1^-allyl-*N*^3^-(4-nitrobenzyl)quinazoline-2,4-dione **18g** (0.443g, 66%) was obtained as a white amorphous solid, m.p. = 139–141 °C. IR (KBr, cm^–1^) ν_max_: 3108, 3008, 1701, 1665, 1481, 1397, 1345, 1213, 959, 836 ^1^H NMR (600 MHz, CDCl_3_): δ = 8.24 (dd, *J* = 9.4 Hz, *J* = 1.6 Hz, 1H), 8.17–8.15 (m, 2H), 7.68–7.65 (m, 3H), 7.28 (t, *J* = 7.7 Hz, 1H), 7.19 (d, *J* = 8.4 Hz, 1H), 5.77 (ddt, ^3^*J* = 17.2 Hz, ^3^*J* = 10.2 Hz, ^3^*J* = 5.0 Hz, 1H, CH_2_–C*H*=CH_2_), 5.36 (s, 2H, C*H*_2_Ph), 5.28 (d, ^3^*J* = 10.2 Hz, 1H, CH_2_–CH=CH*H*), 5.20 (d, ^3^*J* = 17.2 Hz, 1H, CH_2_–CH=C*H*H), 4.78–4.77 (m, 2H, C*H*_2_–CH=CH_2_); ^13^C NMR (151 MHz, CDCl_3_): δ = 161.74 (C=O), 150.69 (C=O), 147.42, 144.20, 139.84, 135.47, 130.97, 129.72, 129.20, 123.74, 123.37, 117.87, 115.40, 114.38, 46.17, 44.44. Anal. calcd. For C_18_H_15_N_3_O_4_: C, 64.09; H, 4.48; N, 12.46. Found: C, 63.79; H, 4.24; N, 12.31.

### 3.3. General Procedure for the Preparation of Isoxsazolidines ***16a**–**g***

A mixture of *N*-benzyl-*C*-(dibenzyloxyphosphoryl)nitrone **17** (1.00 mmol) and the respective *N*^1^-allyl-*N*^3^-benzylquinazoline-2,4-dione **18a**–**g** (1.00 mmol) in toluene was stirred at 60 °C for 5 days. After solvents were removed, crude products were purified by silica gel chromatography with a toluene–ethyl acetate mixture (20:1, 5:1 *v*/*v*). Diastereoisomers *cis*-**16a**–**g**/*trans*-**16a**–**g** were separated by HPLC with a mobile phase of water–isopropanol (60:40–57:43, *v*/*v*) at a flow rate of 17 mL/min to yield *trans*-**16a**–**g** and mixture of *cis*-**16a**–**g** and *trans*-**16a**–**g**.

Dibenzyl *cis*- and *trans*-2-benzyl-5-((3-benzyl-3,4-dihydro-2,4-dioxoquinazolin-1(2*H*)-yl)methyl)isoxazolidin-3-yl-3-phosphonate (*cis*-**16a** and *trans*-**16a**).

According to the general procedure from *N*-benzyl-*C*-(dibenzyloxyphosphoryl)nitrone **17** (0.100 g, 0.322 mmol) and *N*^1^-allyl-*N*^3^-benzylquinazoline-2,4-dione **18a** (0.127 g, 0.322 mmol), pure *trans*-**16a** (0.064 g, 27%) and a mixture of *cis*-**16a** and *trans*-**16a** (0.085 g, 35%) were obtained by column chromatography (toluene–ethyl acetate 20:1, 5:1, *v*/*v*) and next by HPLC with a mobile phase of water–isopropanol (59:41, *v*/*v*).

*Compound cis*-**16a**. Data noted below correspond to a 97:3 mixture of *cis*-**16a** and *trans*-**16a**. A colorless oil. IR (film, cm^–1^) ν_max_: 3453, 3061, 2954, 1951, 1892, 1817, 1698, 1485, 1304, 1233, 1008. NMR signals of *cis*-**16a** were extracted from the spectrum of a 97:3 mixture of *cis*-**16a** and *trans*-**16a**. ^1^H NMR (600 MHz, CDCl_3_): δ = 8.12 (dd, *J* = 7.8 Hz, *J* = 1.5 Hz, 1H), 7.52 (d, *J* = 7.3 Hz, 2H), 7.42–7.37 (m, 7H), 7.36–7.30 (m, 5H), 7.29–7.23 (m, 6H), 7.15 (d, *J* = 8.5 Hz, 1H), 7.07 (t, *J* = 7.4 Hz, 1H), 7.01–6.98 (m, 1H), 5.28 (AB, *J*_AB_ = 13.8 Hz, 1H, *H*CHN), 5.21 (AB, *J*_AB_ = 13.8 Hz, 1H, HC*H*N), 5.21–5.18 (m, 2H, CH_2_OP), 5.15–5.09 (m, 2H, CH_2_OP), 4.56 (dddd, ^3^*J*_(H5–H4α)_ = 9.6 Hz, ^3^*J*_(H5–CH)_ = 7.8 Hz, ^3^*J*_(H5–H4β)_ = 3.8 Hz, ^3^*J*_(H5–CH)_ = 3.8 Hz, 1H, *H*C5), 4.39 (d, ^2^*J* = 13.7 Hz, 1H, *H*CHPh), 4.05 (dAB, *J*_AB_ = 15.0 Hz, ^3^*J*_(H5–CH)_ = 3.8 Hz, 1H, *H*CHN), 4.03 (dAB, *J*_AB_ = 15.0 Hz, ^3^*J*_(H5–CH)_ = 7.8 Hz, 1H, HC*H*N), 3.85 (d, ^2^*J* = 13.7 Hz, 1H, HC*H*Ph), 3.23 (ddd, ^3^*J*_(H3–H4α)_ = 9.6 Hz, ^3^*J*_(H3–H4β)_ = 7.2 Hz, ^2^*J*_(H3–P)_ = 2.6 Hz, 1H, *H*C3), 2.75 (dddd, ^2^*J*_(H4α–H4β)_ = 13.2 Hz, ^3^*J*_(H4α–H3)_ = 9.6 Hz, ^3^*J*_(H4α–H5)_ = 9.6 Hz, ^3^*J*_(H4α–P)_ = 9.4 Hz, 1H, *H*αC4), 2.38 (dddd, ^3^*J*_(H4β–P)_ = 19.6 Hz, ^2^*J*_(H4β–H4α)_ = 13.2 Hz, ^3^*J*_(H4β–H3)_ = 7.2 Hz, ^3^*J*_(H4β–H5)_ = 3.8 Hz, 1H, *H*βC4); ^13^C NMR (151 MHz, CDCl_3_): δ = 161.98 (C=O), 151.22 (C=O), 140.37, 137.03, 136.42, 136.02 (d, ^3^*J*_(CCOP)_ = 6.0 Hz), 135.82 (d, ^3^*J*_(CCOP)_ = 5.4 Hz), 134.82, 129.99, 129.08, 128.76, 128.74, 128.72, 128.43, 128.28, 128.22, 128.17, 128.13, 127.59, 127.56, 122.65, 115.39, 115.02, 75.84 (d, ^3^*J*_(CCCP)_ = 6.6 Hz, C5), 68.37 (d, ^2^*J*_(COP)_ = 6.4 Hz, CH_2_OP), 68.15 (d, ^2^*J*_(COP)_ = 6.7 Hz, CH_2_OP), 62.24 (d, ^3^*J*_(CNCP)_ = 5.1 Hz, *C*H_2_Ph), 60.75 (d, ^1^*J*_(CP)_ = 170.2 Hz, C3), 47.62 (*C*H_2_N), 44.87 (*C*H_2_Ph), 34.97 (C4); ^31^P NMR (243 MHz, CDCl_3_): δ = 23.70. Anal. calcd. for C_40_H_38_N_3_O_6_P × 0.25 H_2_O: C, 69.41; H, 5.61; N, 6.07. Found: C, 69.57; H, 5.81; N, 5.89 (obtained on 97:3 mixture of *cis*-**16a** and *trans*-**16a**).

*Compound trans*-**16a**. A colorless oil. IR (film, cm^–1^) ν_max_: 3454, 3061, 2955, 1952, 1892, 1817, 1698, 1485, 1304, 1233, 1008. ^1^H NMR (600 MHz, CDCl_3_): δ = 8.26 (dd, *J* = 7.9 Hz, *J* = 1.4 Hz, 1H), 7.63–7.61 (m, 1H), 7.52 (d, *J* = 7.3 Hz, 2H), 7.33–7.31 (m, 12H), 7.30–7.25 (m, 8H), 5.31 (AB, *J*_AB_ = 13.9 Hz, 1H, *H*CHN), 5.25 (AB, *J*_AB_ = 13.9 Hz, 1H, HC*H*N), 5.16–5.06 (m, 4H, 2 × CH_2_OP), 4.45 (dd, ^2^*J* = 15.1 Hz, ^3^*J*_(HC–H5)_ = 4.2 Hz, 1H, HC*H*N), 4.41 (d, ^2^*J* = 13.8 Hz, 1H, *H*CHPh), 4.31 (dddd, ^3^*J*_(H4β–H5)_ = 8.4 Hz, ^3^*J*_(H4α–H5)_ = 6.6 Hz, ^3^*J*_(HC–H5)_ = 5.9 Hz, ^3^*J*_(HC–H5)_ = 4.2 Hz, 1H, *H*C5), 4.13 (dd, ^2^*J* = 15.1 Hz, ^3^*J*_(HC–H5)_ = 5.9 Hz, 1H, *H*CHN), 3.90 (d, ^2^*J* = 13.8 Hz, 1H, HC*H*Ph), 3.34 (ddd, ^3^*J*_(H3–H4β)_ = 10.2 Hz, ^3^*J*_(H3–H4α)_ = 6.6 Hz, ^2^*J*_(H3–P)_ = 1.8 Hz, 1H, *H*C3), 2.67 (dddd, ^3^*J*_(H4α–P)_ = 18.6 Hz, ^2^*J*_(H4α–H4β)_ = 12.8 Hz, ^3^*J*_(H4α–H3)_ = 6.6 Hz, ^3^*J*_(H4α–H5)_ = 6.6 Hz, 1H, *H*αC4), 2.33 (dddd, ^3^*J*_(H4β–P)_ = 16.8 Hz, ^2^*J*_(H4β–H4α)_ = 12.8 Hz, ^3^*J*_(H4β–H3)_ = 10.2 Hz, ^3^*J*_(H4β–H5)_ = 8.4 Hz, 1H, *H*βC4); ^13^C NMR (151 MHz, CDCl_3_): δ = 161.63 (C=O), 151.26 (C=O), 140.13, 136.92, 136.38, 136.17 (d, ^3^*J*_(CCOP)_ = 5.7 Hz), 136.04 (d, ^3^*J*_(CCOP)_ = 5.8 Hz), 134.85, 129.70, 128.99, 128.95, 128.65, 128.62, 128.59, 128.53, 128.48, 128.17, 128.15, 127.66, 127.17, 123.54, 115.59, 114.90, 75.81 (d, ^3^*J*_(CCCP)_ = 6.1 Hz, C5), 68.75 (d, ^2^*J*_(COP)_ = 6.4 Hz, CH_2_OP), 67.97 (d, ^2^*J*_(COP)_ = 6.7 Hz, CH_2_OP), 62.74 (d, ^3^*J*_(CNCP)_ = 4.7 Hz, *C*H_2_Ph), 60.97 (d, ^1^*J*_(CP)_ = 170.4 Hz, C3), 45.63 (*C*H_2_N), 45.08 (*C*H_2_Ph), 35.04 (C4); ^31^P NMR (243 MHz, CDCl_3_): δ = 22.81. Anal. calcd. for C_40_H_38_N_3_O_6_P × 0.25 H_2_O: C, 69.41; H, 5.61; N, 6.07. Found: C, 69.57; H, 5.83; N, 5.87.

Dibenzyl *cis*- and *trans*-5-((3-(2-fluorobenzyl)-3,4-dihydro-2,4-dioxoquinazolin-1(2*H*)-yl)methyl)-2-benzylisoxazolidin-3-yl-3-phosphonate (*cis*-**16b** and *trans*-**16b**).

According to the general procedure from *N*-benzyl-*C*-(dibenzyloxyphosphoryl)nitrone **17** (0.100 g, 0.322 mmol) and *N^1^*-allyl-*N*^3^-(2-fluorobenzyl)quinazoline-2,4-dione **18b** (0.127 g, 0.322 mmol), pure *trans*-**16b** (0.079 g, 35%) and a mixture of *cis*-**16b** and *trans*-**16b** (0.076 g, 33%) were obtained by column chromatography (toluene–ethyl acetate 20:1, 5:1, *v*/*v*) and next by HPLC with a mobile phase of water–isopropanol (60:40, *v*/*v*).

*Compound cis*-**16b**. Data noted below correspond to a 90:10 mixture of *cis*-**16b** and *trans*-**16b**. A colorless oil. IR (film, cm^–1^) ν_max_: 3453, 3063, 2956, 1956, 1885, 1817, 1662, 1483, 1318, 1230, 1008, 734. NMR signals of *cis*-**16b** were extracted from the spectrum of a 90:10 mixture of *cis*-**16b** and *trans*-**16b**. ^1^H NMR (600 MHz, CDCl_3_): δ = 8.14 (dd, *J* = 7.8 Hz, *J* = 1.4 Hz, 1H), 7.42–7.39 (m, 7H), 7.37–7.31 (m, 3H), 7.30–7.22 (m, 7H), 7.20 (d, *J* = 8.5 Hz, 1H), 7.10–7.05 (m, 3H), 7.03–7.01 (m, 1H), 5.38 (AB, *J*_AB_ = 14.6 Hz, 1H, *H*CHN), 5.32 (AB, *J*_AB_ = 14.6 Hz, 1H, HC*H*N), 5.23–5.18 (m, 2H, CH_2_OP), 5.15–5.08 (m, 2H, CH_2_OP), 4.59–4.55 (m, 1H, *H*C5), 4.40 (d, ^2^*J* = 13.7 Hz, 1H, *H*CHPh), 4.07 (d, ^3^*J* = 5.7 Hz, 2H, *H*C*H*N), 3.86 (d, ^2^*J* = 13.7 Hz, 1H, HC*H*Ph), 3.23 (ddd, ^3^*J*_(H3–H4α)_ = 10.1 Hz, ^3^*J*_(H3–H4β)_ = 7.2 Hz, ^2^*J*_(H3–P)_ = 2.6 Hz, 1H, *H*C3), 2.74 (dddd, ^2^*J*_(H4α–H4β)_ = 13.2 Hz, ^3^*J*_(H4α–H3)_ = 10.1 Hz, ^3^*J*_(H4α–H5)_ = 9.6 Hz, ^3^*J*_(H4α–P)_ = 9.0 Hz,1H, *H*αC4), 2.38 (dddd, ^3^*J*_(H4β–P)_ = 19.8 Hz, ^2^*J*_(H4β–H4α)_ = 13.2 Hz, ^3^*J*_(H4β–H3)_ = 7.2 Hz, ^3^*J*_(H4β–H5)_ = 4.2 Hz, 1H, *H*βC4); ^13^C NMR (151 MHz, CDCl_3_): δ = 161.94 (C=O), 160.74 (d, ^1^*J*_(CF)_ = 247.3 Hz), 151.04 (C=O), 140.45, 136.47, 136.49 (d, ^3^*J*_(CCOP)_ = 5.4 Hz), 136.05 (d, ^3^*J*_(CCOP)_ = 5.6 Hz), 134.96, 129.96, 129.49 (d, ^3^*J*_(CCCF)_ = 8.4 Hz), 129.39 (d, ^4^*J*_(CCCCF)_ = 3.6 Hz), 128.76, 128.74, 128.72, 128.30, 128.23, 128.17, 127.58, 124.09 (d, ^3^*J*_(CCCF)_ = 3.5 Hz), 123.94 (d, ^2^*J*_(CCF)_ = 14.3 Hz), 122.74, 115.47 (d, ^2^*J*_(CCF)_ = 21.7 Hz), 115.51, 114.92, 75.38 (d, ^3^*J*_(CCCP)_ = 6.1 Hz, C5), 68.37 (d, ^2^*J*_(COP)_ = 6.6 Hz, CH_2_OP), 68.18 (d, ^2^*J*_(COP)_ = 6.9 Hz, CH_2_OP), 62.28 (d, ^3^*J*_(CNCP)_ = 5.3 Hz, *C*H_2_Ph), 60.77 (d, ^1^*J*_(CP)_ = 169.9 Hz, C3), 47.63 (CH_2_N), 38.59 (d, ^3^*J*_(CCCF)_ = 4.5 Hz, *C*H_2_Ph), 34.97 (C4); ^31^P NMR (243 MHz, CDCl_3_): δ = 23.73. Anal. calcd. for C_40_H_37_FN_3_O_6_P × 3.25 H_2_O: C, 62.87; H, 5.74; N, 5.50. Found: C, 62.65; H, 5.88; N, 5.21 (obtained on 90:10 mixture of *cis*-**16b** and *trans*-**16b**).

*Compound trans*-**16b**. A colorless oil. IR (film, cm^–1^) ν_max_: 3453, 3063, 2956, 1956, 1886, 1817, 1663, 1482, 1347, 1231, 1008, 735. ^1^H NMR (600 MHz, CDCl_3_): δ = 8.27 (dd, *J* = 7.9 Hz, *J* = 1.2 Hz, 1H), 7.64 (t, *J* = 8.2 Hz, 1H), 7.35–7.30 (m, 11H), 7.29–7.23 (m, 7H), 7.22–7.20 (m, 1H), 7.07–7.02 (m, 2H), 5.40 (AB, *J*_AB_ = 14.8 Hz, 1H, *H*CHN), 5.36 (AB, *J*_AB_ = 14.8 Hz, 1H, HC*H*N), 5.14–5.05 (m, 4H, 2 × CH_2_OP), 4.46 (dd, ^2^*J* = 15.1 Hz, ^3^*J*_(HC–H5)_ = 4.1 Hz, 1H, HC*H*N), 4.40 (d, ^2^*J* = 13.7 Hz, 1H, *H*CHPh), 4.35–4.31 (m, 1H, *H*C5), 4.16 (dd, ^2^*J* = 15.1 Hz, ^3^*J*_(HC–H5)_ = 5.9 Hz, 1H, HC*H*N), 3.90 (d, ^2^*J* = 13.7 Hz, 1H, HC*H*Ph), 3.33 (ddd, ^3^*J*_(H4β–H3)_ = 10.1 Hz, ^3^*J*_(H4α–H3)_ = 6.3 Hz, ^2^*J*_(H3–P)_ = 1.8 Hz, 1H, *H*C3), 2.66 (dddd, ^3^*J*_(H4α –P)_ = 18.6 Hz, ^2^*J*_(H4α–H4β)_ = 12.7 Hz, ^3^*J*_(H4α–H3)_ = 6.3 Hz, ^3^*J*_(H4α–H5)_ = 6.3 Hz, 1H, *H*αC4), 2.32 (dddd, ^3^*J*_(H4β–P)_ = 16.8 Hz, ^2^*J*_(H4β–H4α)_ = 12.7 Hz, ^3^*J*_(H4β–H3)_ = 10.1 Hz, ^3^*J*_(H4β–H5)_ = 9.2 Hz, 1H, *H*βC4); ^13^C NMR (151 MHz, CDCl_3_): δ = 161.64 (C=O), 160.74 (d, ^1^*J*_(CF)_ = 247.4 Hz), 151.09 (C=O), 140.20, 136.35, 136.16 (d, ^3^*J*_(CCOP)_ = 5.6 Hz), 136.03 (d, ^3^*J*_(CCOP)_ = 5.7 Hz), 134.97, 129.69, 129.28 (d, ^4^*J*_(CCCCF)_ = 3.8 Hz), 129.04 (d, ^3^*J*_(CCCF)_ = 6.7 Hz), 128.64, 128.61, 128.58, 128.51, 128.16, 128.13, 128.11, 127.53, 124.11 (d, ^3^*J*_(CCCF)_ = 3.8 Hz), 123.78 (d, ^2^*J*_(CCF)_ = 14.3 Hz), 123.25, 115.50 (d, ^2^*J*_(CCF)_ = 21.4 Hz), 115.47, 114.98, 75.76 (d, ^3^*J*_(CCCP)_ = 6.1 Hz, C5), 68.72 (d, ^2^*J*_(COP)_ = 6.5 Hz, CH_2_OP), 67.95 (d, ^2^*J*_(COP)_ = 6.7 Hz, CH_2_OP), 62.71 (d, ^3^*J*_(CNCP)_ = 5.1 Hz, *C*H_2_Ph), 60.93 (d, ^1^*J*_(CP)_ = 170.1 Hz, C3), 45.61 (CH_2_N), 38.94 (d, ^3^*J*_(CCCF)_ = 4.5 Hz, *C*H_2_Ph), 34.97 (C4); ^31^P NMR (243 MHz, CDCl_3_): δ = 22.80. Anal. calcd. for C_40_H_37_FN_3_O_6_P × 2.5 H_2_O: C, 64.00; H, 5.64; N, 5.60. Found: C, 63.74; H, 5.96; N, 5.34.

Dibenzyl *cis*- and *trans*-5-((3-(3-fluorobenzyl)-3,4-dihydro-2,4-dioxoquinazolin-1(2*H*)-yl)methyl)-2-benzylisoxazolidin-3-yl-3-phosphonate (*cis*-**16c** and *trans*-**16c**). 

According to the general procedure from *N*-benzyl-*C*-(dibenzyloxyphosphoryl)nitrone **17** (0.100 g, 0.322 mmol) and *N*^1^-allyl-*N*^3^-(3-fluorobenzyl)quinazoline-2,4-dione **18c** (0.127 g, 0.322 mmol), pure *trans*-**16c** (0.075 g, 34%) and a mixture of *cis*-**16c** and *trans*-**16c** (0.078 g, 35%) were obtained by column chromatography (toluene–ethyl acetate 20:1, 5:1, *v*/*v*) and next by HPLC with a mobile phase of water–isopropanol (58:42, *v*/*v*).

*Compound cis*-**16c**. Data noted below correspond to a 90:10 mixture of *cis*-**16c** and *trans*-**16c**. A colorless oil. IR (film, cm^–1^) ν_max_: 3442, 3063, 2954, 1960, 1893, 1820, 1657, 1485, 1346, 1233, 1010, 698. NMR signals of *cis*-**16c** were extracted from the spectrum of a 90:10 mixture of *cis*-**16c** and *trans*-**16c**. ^1^H NMR (600 MHz, CDCl_3_): δ = 8.12 (dd, *J* = 7.8 Hz, *J* = 1.0 Hz, 1H), 7.42–7.37 (m, 6H), 7.36–7.30 (m, 4H), 7.29–7.24 (m, 7H), 7.20–7.18 (m, 1H), 7.17 (d, *J* = 8.5 Hz, 1H), 7.08 (t, *J* = 7.4 Hz, 1H), 7.01–6.95 (m, 2H), 5.26 (AB, *J*_AB_ = 14.2 Hz, 1H, *H*CHN), 5.20 (AB, *J*_AB_ = 14.2 Hz, 1H, HC*H*N), 5.15–5.08 (m, 2H, CH_2_OP), 5.23–5.18 (m, 2H, CH_2_OP), 4.58–4.54 (m, 1H, *H*C5), 4.40 (d, ^2^*J* = 13.6 Hz, 1H, *H*CHPh), 4.05 (d, ^3^*J* = 5.6 Hz, 2H, *H*C*H*N), 3.85 (d, ^2^*J* = 13.6 Hz, 1H, HC*H*Ph), 3.23 (ddd, ^3^*J*_(H3–H4α)_ = 10.1 Hz, ^3^*J*_(H3–H4β)_ = 7.4 Hz, ^2^*J*_(H3–P)_ = 2.4 Hz, 1H, *H*C3), 2.76 (dddd, ^2^*J*_(H4α–H4β)_ = 12.6 Hz, ^3^*J*_(H4α–P)_ = 10.1 Hz, ^3^*J*_(H4α–H3)_ = 10.1 Hz, ^3^*J*_(H4α–H5)_ = 9.0 Hz, 1H, *H*αC4), 2.38 (dddd, ^3^*J*_(H4β–P)_ = 19.2 Hz, ^2^*J*_(H4β–H4α)_ = 12.6 Hz, ^3^*J*_(H4β–H3)_ = 7.4 Hz, ^3^*J*_(H4β–H5)_ = 4.1 Hz, 1H, *H*βC4); ^13^C NMR (151 MHz, CDCl_3_): δ = 162.78 (d, ^1^*J*_(CF)_ = 245.7 Hz), 161.89 (C=O), 151.15 (C=O), 140.38, 139.37 (d, ^3^*J*_(CCCF)_ = 7.1 Hz), 136.43, 136.03 (d, ^3^*J*_(CCOP)_ = 5.7 Hz), 135.97 (d, ^3^*J*_(CCOP)_ = 5.6 Hz), 134.97, 129.98, 129.89 (d, ^3^*J*_(CCCF)_ = 8.4 Hz), 128.77, 128.75, 128.73, 128.29, 128.23, 128.18, 128.15, 128.13, 127.57, 124.60 (d, ^4^*J*_(CCCCF)_ = 2.3 Hz), 122.76, 115.96 (d, ^2^*J*_(CCF)_ = 21.9 Hz), 115.49, 114.91, 114.55 (d, ^2^*J*_(CCF)_ = 21.0 Hz), 75.82 (d, ^3^*J*_(CCCP)_ = 6.7 Hz, C5), 68.38 (d, ^2^*J*_(COP)_ = 6.6 Hz, CH_2_OP), 68.18 (d, ^2^*J*_(COP)_ = 7.2 Hz, CH_2_OP), 62.27 (d, ^3^*J*_(CNCP)_ = 5.2 Hz, *C*H_2_Ph), 60.77 (d, ^1^*J*_(CP)_ = 169.8 Hz, C3), 47.66 (CH_2_N), 44.41 (*C*H_2_Ph), 34.98 (C4); ^31^P NMR (243 MHz, CDCl_3_): δ = 23.70. Anal. calcd. for C_40_H_37_FN_3_O_6_P × 0.25 H_2_O: C, 67.65; H, 5.32; N, 5.92. Found: C, 67.76; H, 5.25; N, 5.90 (obtained on 90:10 mixture of *cis*-**16c** and *trans*-**16c**).

*Compound* trans-**16c**. A colorless oil. IR (film, cm^–1^) ν_max_: 3441, 3063, 2954, 1960, 1893, 1820, 1657, 1485, 1346, 1233, 1010, 698. ^1^H NMR (600 MHz, CDCl_3_): δ = 8.26 (dd, *J* = 7.8 Hz, *J* = 1.0 Hz, 1H), 7.63 (t, *J* = 7.4 Hz, 1H), 7.35–7.31 (m, 11H), 7.29–7.21 (m, 9H), 6.97–6.95 (m, 1H), 5.28 (AB, *J*_AB_ = 14.0 Hz, 1H, *H*CHN), 5.23 (AB, *J*_AB_ = 14.0 Hz, 1H, HC*H*N), 5.15–5.05 (m, 4H, 2 × CH_2_OP), 4.45 (dd, ^2^*J* = 15.1 Hz, ^3^*J*_(HC–H5)_ = 4.1 Hz, 1H, *H*CHN), 4.40 (d, ^2^*J* = 13.8 Hz, 1H, *H*CHPh), 4.34–4.29 (m, 1H, *H*C5), 4.14 (dd, ^2^*J* = 15.1 Hz, ^3^*J*_(HC–H5)_ = 6.1 Hz, 1H, HC*H*N), 3.90 (d, ^2^*J* = 13.8 Hz, 1H, HC*H*Ph), 3.34 (ddd, ^3^*J*_(H4β–H3)_ = 10.2 Hz, ^3^*J*_(H4α–H3)_ = 6.3 Hz, ^2^*J*_(H3–P)_ = 1.2 Hz, 1H, *H*C3), 2.67 (dddd, ^3^*J*_(H4α –P)_ = 18.5 Hz, ^2^*J*_(H4α–H4β)_ = 12.6 Hz, ^3^*J*_(H4α–H3)_ = 6.3 Hz, ^3^*J*_(H4α–H5)_ = 6.3 Hz, 1H, *H*αC4), 2.32 (dddd, ^3^*J*_(H4β–P)_ = 15.0 Hz, ^2^*J*_(H4β–H4α)_ = 12.6 Hz, ^3^*J*_(H4β–H3)_ = 10.2 Hz, ^3^*J*_(H4β–H5)_ = 10.2 Hz, 1H, *H*βC4); ^13^C NMR (150 MHz, CDCl_3_): δ = 162.79 (d, ^1^*J*_(CF)_ = 246.1 Hz), 161.63 (C=O), 151.18 (C=O), 140.13, 139.23 (d, ^3^*J*_(CCCF)_ = 7.1 Hz), 136.34, 136.15 (d, ^3^*J*_(CCOP)_ = 5.6 Hz), 136.03 (d, ^3^*J*_(CCOP)_ = 5.6 Hz), 134.99, 129.95 (d, ^3^*J*_(CCCF)_ = 8.5 Hz), 129.70, 128.97, 128.64, 128.61, 128.59, 128.53, 128.15, 128.13, 127.54, 124.54 (d, ^4^*J*_(CCCCF)_ = 3.0 Hz), 123.28, 115.83 (d, ^2^*J*_(CCF)_ = 21.7 Hz), 115.47, 114.97 114.62 (d, ^2^*J*_(CCF)_ = 21.1 Hz), 75.74 (d, ^3^*J*_(CCCP)_ = 5.8 Hz, C5), 68.74 (d, ^2^*J*_(COP)_ = 6.4 Hz, CH_2_OP), 67.98 (d, ^2^*J*_(COP)_ = 6.8 Hz, CH_2_OP), 62.73 (d, ^3^*J*_(CNCP)_ = 4.7 Hz, *C*H_2_Ph), 60.95 (d, ^1^*J*_(CP)_ = 170.3 Hz, C3), 45.71 (CH_2_N), 44.59 (*C*H_2_Ph), 35.05 (d, ^2^*J*_(CCP)_ = 1.8 Hz, C4); ^31^P NMR (243 MHz, CDCl_3_): δ = 22.78. Anal. calcd. for C_40_H_37_FN_3_O_6_P × H_2_O: C, 66.39; H, 5.43; N, 5.81. Found: C, 66.20; H, 5.34; N, 5.72.

Dibenzyl *cis*- and *trans*-5-((3-(4-fluorobenzyl)-3,4-dihydro-2,4-dioxoquinazolin-1(2*H*)-yl)methyl)-2-benzylisoxazolidin-3-yl-3-phosphonate (*cis*-**16d** and *trans*-**16d**).

According to the general procedure from *N*-benzyl-*C*-(dibenzyloxyphosphoryl)nitrone **17** (0.100 g, 0.322 mmol) and *N*^1^-allyl-*N*^3^-(4-fluorobenzyl)quinazoline-2,4-dione **18d** (0.127 g, 0.322 mmol), pure *trans*-**16d** (0.088 g, 39%) and a mixture of *cis*-**16d** and *trans*-**16d** (0.067 g, 30%) were obtained by column chromatography (toluene–ethyl acetate 20:1, 5:1, *v*/*v*) and next by HPLC with a mobile phase of water–isopropanol (57:43, *v*/*v*).

*Compound cis*-**16d**. Data noted below correspond to a 96:4 mixture of *cis*-**16d** and *trans*-**16d**. A colorless oil. IR (film, cm^–1^) ν_max_: 3457, 3063, 2957, 1956, 1893, 1816, 1658, 1496, 1347, 1220, 1007, 825. NMR signals of *cis*-**16d** were extracted from the spectrum of a 96:4 mixture of *cis*-**16d** and *trans*-**16d**. ^1^H NMR (600 MHz, CDCl_3_): δ = 8.12 (dd, *J* = 7.9 Hz, *J* = 1.4 Hz, 1H), 7.54–7.51 (m, 2H), 7.41–7.35 (m, 7H), 7.34–7.30 (m, 3H), 7.29–7.22 (m, 5H), 7.17 (d, *J* = 8.5 Hz, 1H), 7.08 (t, *J* = 7.5 Hz, 1H), 7.01–6.98 (m, 3H), 5.24–5.15 (m, 4H, *H*C*H*N, CH_2_OP), 5.14–5.09 (m, 2H, CH_2_OP), 4.59–4.55 (m, 1H, *H*C5), 4.40 (d, ^2^*J* = 13.7 Hz, 1H, *H*CHPh), 4.06 (d, ^3^*J* = 5.7 Hz, 2H, *H*C*H*N), 3.85 (d, ^2^*J* = 13.7 Hz, 1H, HC*H*Ph), 3.23 (ddd, ^3^*J*_(H3–H4α)_ = 9.8 Hz, ^3^*J*_(H3–H4β)_ = 7.3 Hz, ^2^*J*_(H3–P)_ = 2.5 Hz, 1H, *H*C3), 2.76 (dddd, ^2^*J*_(H4α–H4β)_ = 12.8 Hz, ^3^*J*_(H4α–P)_ = 11.4 Hz, ^3^*J*_(H4α–H3)_ = 9.8 Hz, ^3^*J*_(H4α–H5)_ = 9.8 Hz, 1H, *H*αC4), 2.39 (dddd, ^3^*J*_(H4β–P)_ = 19.0 Hz, ^2^*J*_(H4β–H4α)_ = 12.8 Hz, ^3^*J*_(H4β–H3)_ = 7.3 Hz, ^3^*J*_(H4β–H5)_ = 4.3 Hz, 1H, *H*βC4); ^13^C NMR (151 MHz, CDCl_3_): δ = 162.27 (d, ^1^*J*_(CF)_ = 245.9 Hz), 161.90 (C=O), 151.18 (C=O), 140.37, 136.44, 136.04 (d, ^3^*J*_(CCOP)_ = 5.5 Hz), 135.98 (d, ^3^*J*_(CCOP)_ = 5.5 Hz), 134.90, 132.69 (d, ^4^*J*_(CCCCF)_ = 3.2 Hz), 131.00 (d, ^3^*J*_(CCCF)_ = 8.2 Hz), 129.96, 128.77, 128.75, 128.73, 128.29, 128.23, 128.17, 127.57, 122.71, 115.46, 115.22 (d, ^2^*J*_(CCF)_ = 21.2 Hz), 114.98, 75.82 (d, ^3^*J*_(CCCP)_ = 6.7 Hz, C5), 68.38 (d, ^2^*J*_(COP)_ = 6.6 Hz, CH_2_OP), 68.18 (d, ^2^*J*_(COP)_ = 7.1 Hz, CH_2_OP), 62.27 (d, ^3^*J*_(CNCP)_ = 5.1 Hz, *C*H_2_Ph), 60.79 (d, ^1^*J*_(CP)_ = 170.2 Hz, C3), 47.63 (CH_2_N), 44.16 (*C*H_2_Ph), 34.98 (C4); ^31^P NMR (243 MHz, CDCl_3_): δ = 23.70. Anal. calcd. for C_40_H_37_FN_3_O_6_P × 0.75 H_2_O: C, 66.80; H, 5.40; N, 5.84. Found: C, 66.78; H, 5.56; N, 5.91 (obtained on 96:4 mixture of *cis*-**16d** and *trans*-**16d**).

*Compound trans*-**16d**. A colorless oil. IR (film, cm^–1^) ν_max_: 3455, 3063, 2957, 1956, 1896, 1817, 1659 1497, 1347, 1220, 993, 855. ^1^H NMR (600 MHz, CDCl_3_): δ = 8.24 (dd, *J* = 7.9 Hz, *J* = 1.5 Hz, 1H), 7.64–7.61 (m, 1H), 7.54–7.50 (m, 2H), 7.36–7.31 (m, 10H), 7.30–7.23 (m, 7H), 6.99–6.95 (m, 2H), 5.25 (AB, *J*_AB_ = 13.8 Hz, 1H, *H*CHN), 5.20 (AB, *J*_AB_ = 13.8 Hz, 1H, HC*H*N), 5.12–5.05 (m, 4H, 2 × CH_2_OP), 4.45 (dd, ^2^*J* = 15.1 Hz, ^3^*J*_(HC–H5)_ = 4.1 Hz, 1H, *H*CHN), 4.39 (d, ^2^*J* = 13.8 Hz, 1H, *H*CHPh), 4.32–4.28 (m, 1H, *H*C5), 4.13 (dd, ^2^*J* = 15.1 Hz, ^3^*J*_(HC–H5)_ = 6.1 Hz, 1H, HC*H*N), 3.89 (d, ^2^*J* = 13.8 Hz, 1H, HC*H*Ph), 3.33 (ddd, ^3^*J*_(H3–H4β)_ = 9.9 Hz, ^3^*J*_(H3–H4α)_ = 6.3 Hz, ^2^*J*_(H3–P)_ = 1.7 Hz, 1H, *H*C3), 2.67 (dddd, ^3^*J*_(H4α–P)_ = 18.5 Hz, ^2^*J*_(H4α–H4β)_ = 12.6 Hz, ^3^*J*_(H4α–H3)_ = 6.3 Hz, ^3^*J*_(H4α–H5)_ = 6.3 Hz, 1H, *H*αC4), 2.32 (dddd, ^3^*J*_(H4β–P)_ = 16.7 Hz, ^2^*J*_(H4β–H4α)_ = 12.6 Hz, ^3^*J*_(H4β–H3)_ = 9.9 Hz, ^3^*J*_(H4β–H5)_ = 8.8 Hz, 1H, *H*βC4); ^13^C NMR (151 MHz, CDCl_3_): δ = 162.30 (d, ^1^*J*_(CF)_ = 246.5 Hz), 161.66 (C=O), 151.20 (C=O), 140.09, 136.33, 136.59 (d, ^3^*J*_(CCOP)_ = 6.0 Hz), 136.13 (d, ^3^*J*_(CCOP)_ = 5.5 Hz), 134.92, 132.69 (d, ^4^*J*_(CCCCF)_ = 3.3 Hz), 131.05 (d, ^3^*J*_(CCCF)_ = 8.0 Hz), 129.68, 128.92, 128.64, 128.61, 128.53, 128.15, 128.13, 128.12, 127.54, 123.23, 115.53, 115.26 (d, ^2^*J*_(CCF)_ = 21.1 Hz), 114.92, 75.74 (d, ^3^*J*_(CCCP)_ = 5.7 Hz, C5), 68.73 (d, ^2^*J*_(COP)_ = 6.5 Hz, CH_2_OP), 67.97 (d, ^2^*J*_(COP)_ = 6.8 Hz, CH_2_OP), 62.73 (*C*H_2_Ph), 60.95 (d, ^1^*J*_(CP)_ = 170.2 Hz, C3), 45.67 (CH_2_N), 44.33 (*C*H_2_Ph), 35.05 (d, ^2^*J*_(CCP)_ = 2.2 Hz, C4); ^31^P NMR (243 MHz, CDCl_3_): δ = 22.76. Anal. calcd. for C_40_H_37_FN_3_O_6_P × 0.5 H_2_O: C, 67.22; H, 5.36; N, 5.88. Found: C, 67.38; H, 5.37; N, 5.63.

Dibenzyl *cis*- and *trans*- (2-benzyl-5-((3-(2-nitrobenzyl)-2,4-dioxo-3,4-dihydroquinazolin-1(2H)-yl)methyl)isoxazolidin-3-yl)phosphonate (*cis*-**16e** and *trans*-**16e**).

According to the general procedure from *N*-benzyl-*C*-(dibenzyloxyphosphoryl)nitrone **17** (0.059 g, 0.148 mmol) and *N*^1^-allyl-*N*^3^-(2-nitrobenzyl)quinazoline-2,4-dione **18e** (0.050 g, 0.148 mmol), pure *trans*-**16e** (0.018 g, 17%) and a mixture of *cis*-**16e** and *trans*-**16e** (0.048 g, 44%) were obtained by column chromatography (toluene–ethyl acetate 20:1, 5:1, *v*/*v*) and next by HPLC with a mobile phase of water–isopropanol (60:40, *v*/*v*).

*Compound cis*-**16e**. Data noted below correspond to a 70:30 mixture of *cis*-**16e** and *trans*-**16e**. A colorless oil. IR (film, cm^–1^) ν_max_: 3442, 3062, 2956, 1957, 1896, 1658, 1482, 1338, 1246, 1019, 760. NMR signals of *cis*-**16e** were extracted from the spectrum of a 70:30 mixture of *cis*-**16e** and *trans*-**16e**. ^1^H NMR (600 MHz, CDCl_3_): δ = 8.13 (dd, *J* = 7.8 Hz, *J* = 1.4 Hz, 1H), 8.07 (d, *J* = 8.2 Hz, 1H), 7.51–7.50 (m, 1H), 7.42–7.39 (m, 5H), 7.35–7.32 (m, 5H), 7.29–7.22 (m, 8H), 7.11 (t, *J* = 7.5 Hz, 1H), 7.07–7.04 (m, 1H), 5.67–5.64 (m, 2H, *H*C*H*N), 5.22–5.07 (m, 4H, 2 CH_2_OP), 4.57–4.53 (m 1H, *H*C5), 4.40 (d, ^2^*J* = 13.7 Hz, 1H, *H*CHPh), 4.12 (dd, ^2^*J* = 14.9 Hz, ^3^*J*_(HC–H5)_ = 9.1 Hz, 1H, *H*CHN), 4.05 (dd, ^2^*J* = 14.9 Hz, ^3^*J*_(HC–H5)_ = 2.2 Hz, 1H, HC*H*N), 3.86 (d, ^2^*J* = 13.7 Hz, 1H, HC*H*Ph), 3.23 (ddd, ^3^*J*_(H3–H4α)_ = 10.0 Hz, ^3^*J*_(H3–H4β)_ = 7.3 Hz, ^2^*J*_(H3–P)_ = 2.7 Hz, 1H, *H*C3), 2.74 (dddd, ^2^*J*_(H4α–H4β)_ = 12.0 Hz, ^3^*J*_(H4α–P)_ = 10.0 Hz, ^3^*J*_(H4α–H3)_ = 10.0 Hz, ^3^*J*_(H4α–H5)_ = 10.0 Hz, 1H, *H*αC4), 2.37 (dddd, ^3^*J*_(H4β–P)_ = 19.7 Hz, ^2^*J*_(H4β–H4α)_ = 12.0 Hz, ^3^*J*_(H4β–H3)_ = 7.3 Hz, ^3^*J*_(H4β–H5)_ = 4.0 Hz, 1H, *H*βC4); ^13^C NMR (151 MHz, CDCl_3_): δ = 161.94 (C=O), 151.00 (C=O), 148.77, 140.51, 136.04, 135.97 (d, ^3^*J*_(CCOP)_ = 5.7 Hz), 135.95 (d, ^3^*J*_(CCOP)_ = 5.4 Hz), 135.23, 135.22, 133.50, 129.92, 128.77, 128.74, 128.72, 128.65, 128.62, 128.56, 128.23, 128.17, 128.14, 125.01, 122.94, 115.68, 114.70, 75.79 (d, ^3^*J*_(CCCP)_ = 6.6 Hz, C5), 68.38 (d, ^2^*J*_(COP)_ = 6.6 Hz, CH_2_OP), 68.22 (d, ^2^*J*_(COP)_ = 7.0 Hz, CH_2_OP), 62.27 (d, ^3^*J*_(CNCP)_ = 5.3 Hz, *C*H_2_Ph), 61.81 (d, ^1^*J*_(CP)_ = 145.3 Hz, C3), 47.61 (CH_2_N), 42.00 (*C*H_2_Ph), 34.91 (C4); ^31^P NMR (243 MHz, CDCl_3_): δ = 23.62. Anal. calcd. for C_40_H_37_N_4_O_8_P × 1.5 H_2_O: C, 63.24; H, 5.31; N, 7.38. Found: C, 63.50; H, 5.09; N, 7.55 (obtained on 70:30 mixture of *cis*-**16e** and *trans*-**16e**).

*Compound trans*-**16e**. A colorless oil. IR (film, cm^–1^) ν_max_: 3441, 3063, 2957, 1957, 1884, 1660, 1482, 1338, 1259, 1020, 760. ^1^H NMR (600 MHz, CDCl_3_): δ = 8.26 (dd, *J* = 7.9 Hz, *J* = 1.3 Hz, 1H), 8.07 (dd, *J* = 8.2 Hz, *J* = 1.0 Hz, 1H), 7.67 (t, *J* = 7.5 Hz, 1H), 7.46–7.43 (m, 1H), 7.39–7.31 (m, 12H), 7.27–7.23 (m, 6H), 7.19 (d, *J* = 7.8 Hz, 1H), 5.68 (AB, *J*_AB_ = 16.3 Hz, 1H, *H*CHN), 5.65 (AB, *J*_AB_ = 16.3 Hz, 1H, HC*H*N), 5.14–5.03 (m, 4H, 2 × CH_2_OP), 4.43 (dd, ^2^*J* = 15.0 Hz, ^3^*J*_(HC–H5)_ = 4.0 Hz, 1H, HC*H*N), 4.40 (d, ^2^*J* = 13.7 Hz, 1H, *H*CHPh), 4.36–4.30 (m 1H, *H*C5), 4.18 (dd, ^2^*J* = 15.0 Hz, ^3^*J*_(HC–H5)_ = 6.1 Hz, 1H, *H*CHN), 3.91 (d, ^2^*J* = 13.7 Hz, 1H, HC*H*Ph), 3.37–3.30 (br m, 1H, *H*C3), 2.64 (dddd, ^3^*J*_(H4α–P)_ = 18.5 Hz, ^2^*J*_(H4α–H4β)_ = 12.7 Hz, ^3^*J*_(H4α–H3)_ = 6.3 Hz, ^3^*J*_(H4α–H5)_ = 6.3 Hz, 1H, *H*αC4), 2.32–2.25 (m, 1H, *H*βC4); ^13^C NMR (151 MHz, CDCl_3_): δ = 161.65 (C=O), 151.04 (C=O), 148.73, 140.21, 136.10, 136.08 (d, ^3^*J*_(CCOP)_ = 5.4 Hz), 135.95 (d, ^3^*J*_(CCOP)_ = 5.5 Hz), 135.26, 133.54, 132.33, 129.74, 129.11, 128.66, 128.62, 128.57, 128.17, 128.14, 128.02, 127.68, 127.61, 125.07, 123.46, 115.25, 115.08, 75.71 (d, ^3^*J*_(CCCP)_ = 5.4 Hz, C5), 68.75 (d, ^2^*J*_(COP)_ = 6.5 Hz, CH_2_OP), 67.86 (d, ^2^*J*_(COP)_ = 6.9 Hz, CH_2_OP), 62.59 (*C*H_2_Ph), 60.83 (d, ^1^*J*_(CP)_ = 170.3 Hz, C3), 45.68 (CH_2_N), 42.27 (*C*H_2_Ph), 34.96 (d, ^2^*J*_(CCP)_ = 1.4 Hz, C4); ^31^P NMR (243 MHz, CDCl_3_): δ = 22.47. Anal. calcd. for C_40_H_37_N_4_O_8_P × 1.75 H_2_O: C, 62.87; H, 5.34; N, 7.33. Found: C, 62.71; H, 5.03; N, 7.03.

Dibenzyl *cis*- and *trans*-2-benzyl-5-((3-(3-nitrobenzyl)-2,4-dioxo-3,4-dihydroquinazolin-1(2*H*)-yl)methyl)isoxazolidin-3-yl)phosphonate (*cis*-**16f** and *trans*-**16f**).

According to the general procedure from *N*-benzyl-*C*-(dibenzyloxyphosphoryl)nitrone **17** (0.059 g, 0.148 mmol) and *N*^1^-allyl-*N*^3^-(3-nitrobenzyl)quinazoline-2,4-dione **18f** (0.050 g, 0.148 mmol), pure *trans*-**16f** (0.042 g, 38%) and a mixture of *cis*-**16f** and *trans*-**16f** (0.031 g, 28%) were obtained by column chromatography (toluene–ethyl acetate 20:1, 5:1, *v*/*v*) and next by HPLC with a mobile phase of water–isopropanol (61:39, *v*/*v*).

*Compound cis*-**16f**. Data noted below correspond to a 96:4 mixture of *cis*-**16f** and *trans*-**16f**. A colorless oil. IR (film, cm^–1^) ν_max_: 3441, 3063, 2924, 1960, 1885, 1658, 1482, 1347, 1235, 1018, 696. NMR signals of *cis*-**16f** were extracted from the spectrum of a 96:4 mixture of *cis*-**16f** and *trans*-**16f**. ^1^H NMR (600 MHz, CDCl_3_): δ = 8.36 (s, 1H), 8.15–8.11 (m, 2H), 7.84 (d, *J* = 7.6 Hz, 1H), 7.49 (t, *J* = 7.9 Hz, 1H), 7.42–7.37 (m, 7H), 7.36–7.32 (m, 3H), 7.30–7.23 (m, 5H), 7.20 (d, *J* = 8.5 Hz, 1H), 7.10 (t, *J* = 7.4 Hz, 1H), 7.03–7.01 (m, 1H), 5.34 (AB, *J*_AB_ = 14.0 Hz, 1H, *H*CHN), 5.29 (AB, *J*_AB_ = 14.0 Hz, 1H, HC*H*N), 5.23–5.18 (m, 2H, CH_2_OP), 5.14–5.09 (m, 2H, CH_2_OP), 4.59–4.55 (m 1H, *H*C5), 4.40 (d, ^2^*J* = 13.7 Hz, 1H, *H*CHPh), 4.09 (dd, ^2^*J* = 14.9 Hz, ^3^*J*_(HC–H5)_ = 8.8 Hz, 1H, HC*H*N), 4.04 (dd, ^2^*J* = 14.9 Hz, ^3^*J*_(HC–H5)_ = 2.5 Hz, 1H, *H*CHN), 3.85 (d, ^2^*J* = 13.7 Hz, 1H, HC*H*Ph), 3.23 (dd, ^3^*J*_(H3–H4α)_ = 9.9 Hz, ^3^*J*_(H3–H4β)_ = 7.3 Hz, ^2^*J*_(H3–P)_ = 2.5 Hz, 1H, *H*C3), 2.77 (dddd, ^2^*J*_(H4α–H4β)_ = 12.0 Hz, ^3^*J*_(H4α–P)_ = 10.0 Hz, ^3^*J*_(H4α–H3)_ = 9.9 Hz, ^3^*J*_(H4α–H5)_ = 9.9 Hz, 1H, *H*αC4), 2.39 (dddd, ^3^*J*_(H4β–P)_ = 19.9 Hz, ^2^*J*_(H4β–H4α)_ = 12.0 Hz, ^3^*J*_(H4β–H3)_ = 7.3 Hz, ^3^*J*_(H4β–H5)_ = 4.0 Hz, 1H, *H*βC4); ^13^C NMR (151 MHz, CDCl_3_): δ = 161.87 (C=O), 151.10 (C=O), 148.33, 140.39, 138.95, 136.33, 136.02 (d, ^3^*J*_(CCOP)_ = 5.7 Hz), 135.95 (d, ^3^*J*_(CCOP)_ = 5.4 Hz), 135.22, 135.16, 129.97, 129.38, 128.78, 128.77, 128.74, 128.28, 128.24, 128.18, 128.16, 127.60, 124.00, 122.91, 122.72, 115.62, 114.77, 75.79 (d, ^3^*J*_(CCCP)_ = 6.6 Hz, C5), 68.40 (d, ^2^*J*_(COP)_ = 6.6 Hz, CH_2_OP), 68.21 (d, ^2^*J*_(COP)_ = 6.7 Hz, CH_2_OP), 62.23 (d, *J* = 4.9 Hz, *C*H_2_Ph), 60.76 (d, ^1^*J*_(CP)_ = 169.8 Hz, C3), 47.69 (CH_2_N), 44.22 (*C*H_2_Ph), 34.92 (C4); ^31^P NMR (243 MHz, CDCl_3_): δ = 23.57. Anal. calcd. for C_40_H_37_N_4_O_8_P × 1.5 H_2_O: C, 63.24; H, 5.31; N, 7.38. Found: C, 63.50; H, 5.09; N, 7.55 (obtained on 96:4 mixture of *cis*-**16f** and *trans*-**16f**).

*Compound trans*-**16f**. A colorless oil. IR (film, cm^–1^) ν_max_: 3441, 3063, 2955, 1959, 1815, 1658, 1482, 1347, 1235, 993, 696. ^1^H NMR (600 MHz, CDCl_3_): δ = 8.37 (s, 1H), 8.25 (d, *J* = 7.9 Hz, 1H), 8.12 (d, *J* = 8.2 Hz, 1H), 7.84 (d, *J* = 7.7 Hz, 1H), 7.65 (t, *J* = 7.9 Hz, 1H), 7.46 (t, *J* = 7.9 Hz, 1H), 7.37–7.29 (m, 12H), 7.27–7.23 (m, 5H), 5.36 (AB, *J*_AB_ = 14.2 Hz, 1H, *H*CHN), 5.32 (AB, *J*_AB_ = 14.2 Hz, 1H, HC*H*N), 5.14–5.05 (m, 4H, 2 × CH_2_OP), 4.45 (dd, ^2^*J* = 15.1 Hz, ^3^*J*_(HC–H5)_ = 3.8 Hz, 1H, HC*H*N), 4.40 (d, ^2^*J* = 13.8 Hz, 1H, *H*CHPh), 4.35– 4.31 (m, 1H, *H*C5), 4.17 (dd, ^2^*J* = 15.1 Hz, ^3^*J*_(HC–H5)_ = 6.2 Hz, 1H, *H*CHN), 3.90 (d, ^2^*J* = 13.8 Hz, 1H, HC*H*Ph), 3.36–3.34 (m, 1H, *H*C3), 2.69 (dddd, ^3^*J*_(H4α–P)_ = 19.1 Hz, ^2^*J*_(H4α–H4β)_ = 13.2 Hz, ^3^*J*_(H4α–H3)_ = 6.8 Hz, ^3^*J*_(H4α–H5)_ = 6.8 Hz, 1H, *H*αC4), 2.36–2.28 (m, 1H, *H*βC4); ^13^C NMR (151 MHz, CDCl_3_): δ = 161.60 (C=O), 151.14 (C=O), 148.34, 140.13, 138.81, 136.20, 136.13 (d, ^3^*J*_(CCOP)_ = 5.7 Hz), 136.01 (d, ^3^*J*_(CCOP)_ = 5.5 Hz), 135.18, 135.13, 129.71, 129.43, 129.00, 128.65, 128.61, 128.55, 128.15, 127.57, 123.93, 123.43, 122.77, 115.34, 115.07, 75.75 (d, ^3^*J*_(CCCP)_ = 5.7 Hz, C5), 68.74 (d, ^2^*J*_(COP)_ = 6.5 Hz, CH_2_OP), 68.03 (d, ^2^*J*_(COP)_ = 6.7 Hz, CH_2_OP), 62.69 (d, *J* = 2.5 Hz, *C*H_2_Ph), 60.95 (d, ^1^*J*_(CP)_ = 170.3 Hz, C3), 45.79 (CH_2_N), 44.38 (*C*H_2_Ph), 35.03 (C4); ^31^P NMR (243 MHz, CDCl_3_): δ = 22.69 Anal. calcd. for C_40_H_37_N_4_O_8_P × 0.75 H_2_O: C, 64.39; H, 5.20; N, 7.51. Found: C, 64.69; H, 5.15; N, 7.22.

Dibenzyl *cis*- and *trans*- (2-benzyl-5-((3-(4-nitrobenzyl)-2,4-dioxo-3,4-dihydroquinazolin-1(2*H*)-yl)methyl)isoxazolidin-3-yl)phosphonate (*cis*-**16g** and *trans*-**16g**). 

According to the general procedure from *N*-benzyl-*C*-(dibenzyloxyphosphoryl)nitrone **17** (0.059 g, 0.148 mmol) and *N*^1^-allyl-*N*^3^-(4-nitrobenzyl)quinazoline-2,4-dione **18g** (0.050 g, 0.148 mmol), pure *trans*-**16g** (0.028 g, 26%) and a mixture of *cis*-**16g** and *trans*-**16g** (0.043 g, 39%) were obtained by column chromatography (toluene–ethyl acetate 20:1, 5:1, *v*/*v*) and next by HPLC with a mobile phase of water–isopropanol (61:39, *v*/*v*).

*Compound cis*-**16g**. Data noted below correspond to an 88:12 mixture of *cis*-**16g** and *trans*-**16g**. A colorless oil. IR (film, cm^–1^) ν_max_: 3442, 3062, 2956, 1954, 1657, 1609, 1482, 1343, 1214, 1023, 803. NMR signals of *cis*-**16g** were extracted from the spectrum of a 88:12 mixture of *cis*-**16g** and *trans*-**16g**. ^1^H NMR (600 MHz, CDCl_3_): δ = 8.18–8.16 (m, 2H), 8.12 (d, *J* = 7.8 Hz, *J* = 1.6 Hz, 1H), 7.64 (t, *J* = 8.8 Hz, 2H), 7.41–7.37 (m, 6H), 7.36–7.32 (m, 4H), 7.30–7.25 (m, 2H), 7.24–7.21 (m, 4H), 7.10 (t, *J* = 7.4 Hz, 1H), 7.05–7.02 (m, 1H), 5.33 (AB, *J*_AB_ = 14.2 Hz, 1H, *H*CHN), 5.29 (AB, *J*_AB_ = 14.2 Hz, 1H, HC*H*N), 5.23–5.17 (m, 2H, CH_2_OP), 5.14–5.08 (m, 2H, CH_2_OP), 4.58–4.55 (m 1H, *H*C5), 4.40 (d, ^2^*J* = 13.7 Hz, 1H, *H*CHPh), 4.11 (dd, ^2^*J* = 14.9 Hz, ^3^*J*_(HC–H5)_ = 9.1 Hz, 1H, HC*H*N), 4.03 (dd, ^2^*J* = 14.9 Hz, ^3^*J*_(HC–H5)_ = 2.3 Hz, 1H, *H*CHN), 3.86 (d, ^2^*J* = 13.7 Hz, 1H, HC*H*Ph), 3.23 (ddd, ^3^*J*_(H3–H4α)_ = 10.0 Hz, ^3^*J*_(H3–H4β)_ = 7.4 Hz, ^2^*J*_(H3–P)_ = 2.7 Hz, 1H, *H*C3), 2.76 (dddd, ^2^*J*_(H4α–H4β)_ = 12.7 Hz, ^3^*J*_(H4α–H3)_ = 10.0 Hz, ^3^*J*_(H4α–H5)_ = 10.0 Hz, ^3^*J*_(H4α–P)_ = 9.3 Hz, 1H, *H*αC4), 2.39 (dddd, ^3^*J*_(H4β–P)_ = 19.1 Hz, ^2^*J*_(H4β–H4α)_ = 12.7 Hz, ^3^*J*_(H4β–H3)_ = 7.4 Hz, ^3^*J*_(H4β–H5)_ = 4.0 Hz, 1H, *H*βC4); ^13^C NMR (151 MHz, CDCl_3_): δ = 161.88 (C=O), 151.07 (C=O), 147.37, 144.23, 140.38, 136.35, 136.01 (d, ^3^*J*_(CCOP)_ = 5.6 Hz), 135.94 (d, ^3^*J*_(CCOP)_ = 5.4 Hz), 135.21, 129.94, 129.70, 128.78, 128.75, 128.60, 128.29, 128.23, 128.17, 128.14, 127.61, 123.70, 122.97, 115.65 114.75, 75.76 (d, ^3^*J*_(CCCP)_ = 5.9 Hz, C5), 68.40 (d, ^2^*J*_(COP)_ = 6.6 Hz, CH_2_OP), 68.22 (d, ^2^*J*_(COP)_ = 7.1 Hz, CH_2_OP), 62.25 (d, *J* = 4.6 Hz, *C*H_2_Ph), 60.76 (d, ^1^*J*_(CP)_ = 170.0 Hz, C3), 47.68 (CH_2_N), 44.28 (*C*H_2_Ph), 34.92 (C4); ^31^P NMR (243 MHz, CDCl_3_): δ = 23.50. Anal. calcd. for C_40_H_37_N_4_O_8_P × 3 H_2_O: C, 61.07; H, 5.51; N, 7.12. Found: C, 60.81; H, 5.45; N, 6.82 (obtained on 88:12 mixture of *cis*-**16g** and *trans*-**16g**).

*Compound trans*-**16g**. A colorless oil. IR (film, cm^–1^) ν_max_: 3454, 3062, 2926, 1954, 1812, 1660, 1612, 1485, 1346, 1216, 1043, 806. ^1^H NMR (600 MHz, CDCl_3_): δ = 8.25 (dd, *J* = 7.9 Hz, *J* = 1.4 Hz, 1H), 8.15 (d, *J* = 10.9 Hz, 2H), 7.67–7.63 (m, 3H), 7.39–7.29 (m, 12H), 7.27–7.23 (m, 5H), 5.33 (AB, *J*_AB_ = 14.3 Hz, 1H, *H*CHN), 5.31 (AB, *J*_AB_ = 14.3 Hz, 1H, HC*H*N), 5.14–5.05 (m, 4H, 2 × C*H*_2_OP), 4.44 (dd, ^2^*J* = 15.1 Hz, ^3^*J*_(HC–H5)_ = 3.8 Hz, 1H, *H*CHN), 4.40 (d, ^2^*J* = 13.8 Hz, 1H, *H*CHPh), 4.35–4.31 (m, 1H, *H*C5), 4.15 (dd, ^2^*J* = 15.1 Hz, ^3^*J*_(HC–H5)_ = 6.5 Hz, 1H, HC*H*N), 3.91 (d, ^2^*J* = 13.8 Hz, 1H, HC*H*Ph), 3.36–3.34 (m, 1H, *H*C3), 2.69 (dddd, ^3^*J*_(H4α–P)_ = 18.5 Hz, ^2^*J*_(H4α–H4β)_ = 12.7 Hz, ^3^*J*_(H4α–H3)_ = 6.4 Hz, ^3^*J*_(H4α–H5)_ = 6.4 Hz, 1H, *H*αC4), 2.35–2.28 (m, 1H, *H*βC4); ^13^C NMR (151 MHz, CDCl_3_): δ = 161.60 (C=O), 151.09 (C=O), 147.41, 144.06, 140.10, 136.25, 136.49 (d, ^3^*J*_(CCOP)_ = 5.6 Hz), 136.10 (d, ^3^*J*_(CCOP)_ = 5.3 Hz), 135.22, 129.67, 128.99, 128.65, 128.62, 128.56, 128.24, 128.16, 128.13, 127.57, 123.73, 123.47, 115.31, 115.07, 75.71 (d, ^3^*J*_(CCCP)_ = 5.7 Hz, C5), 68.73 (d, ^2^*J*_(COP)_ = 6.5 Hz, CH_2_OP), 68.04 (d, ^2^*J*_(COP)_ = 6.7 Hz, CH_2_OP), 62.69 (d, *J* = 3.5 Hz, *C*H_2_Ph), 60.95 (d, ^1^*J*_(CP)_ = 170.2 Hz, C3), 45.88 (CH_2_N), 44.45 (*C*H_2_Ph), 35.11 (C4); ^31^P NMR (243 MHz, CDCl_3_): δ = 22.63. Anal. calcd. for C_40_H_37_N_4_O_8_P × 1.5 H_2_O: C, 63.24; H, 5.31; N, 7.38. Found: C, 63.54; H, 5.30; N, 7.18.

### 3.4. Biological Study In Vitro

#### 3.4.1. Cytotoxicity Assay

The tested compounds were tested against three different human cancer cell lines (breast adenocarcinoma MCF-7 ATCC^®^ HTB-22^TM^, fibrosarcoma HT-1080 ATCC^®^ CCL121^TM^, and prostate adenocarcinoma PC-3 ATCC^®^ CRL-1435^TM)^. For the cell viability experiment, cells were seeded in transparent 96-well plates (FALCON no. 353072, Corning, Durham, NC, USA) in MEM (Gibco no. 31095-029, UK) supplemented with 10% heat-inactivated FBS (Gibco no. 10500-064, UK). The cell seeding density was as follows: MCF-7 12,000 cells/well, HT-1080 7000 cells/well, PC-3 10,000 cells/well. Cells were seeded one day before treatment with the tested compounds and cultured overnight. The confluence on the treatment day was around 30%. The tested compounds were dissolved in DMSO as 10 mM stock solutions and kept under −4 °C. On the day of experimentation, the medium was removed and replaced with a fresh one that contained the following: (1) dimethylsulfoxide (DMSO < 0.1%, vehicle control (Veh)); (2) increasing the concentration of compounds (0.205–50 μM, performed as a 2.5-fold serial dilution for dose–response analysis). Treatment with compounds was carried out for 72 h. Each treatment was replicated three times in a single experiment, with three separate experiments conducted. (n = 9). When significant variability in the results occurred, an additional test was performed on certain compounds. During the incubation, the cells were examined under an inverted microscope to check if the compounds had not precipitated in the culture medium. The inhibitory effect of compounds on the cell was examined using an MTS-based assay (Promega, Madison, WI, USA) following the manufacturer’s protocol. The absorbance was measured at 490 nm using Tecan Spark’s multimode plate reader (Tecan*,* Männedorf, Switzerland). A reference wavelength of 630 nm was used to subtract the background. IC_50_ values were calculated by fitting a non-linear regression to a sigmoidal dose–response curve in GraphPad Prism version 8.0.1.

#### 3.4.2. Safety Studies

In drug development, particular attention should be paid to studying the safety profile of compounds. Promising drug candidates should not cause adverse effects on essential organ systems like the liver, kidney, or healthy cells in general. Systemic toxicity of the compound is the main factor limiting its therapeutic application and efficiency. In our study, a preliminary study on nephrotoxicity and hepatotoxicity was evaluated in human embryonic kidney cells (HEK293, ATCC^®^ CRL1573™) and human hepatoma cells (HepG2, ATCC^®^ HB8065™), respectively. HEK293 is commonly used to evaluate the cytotoxicity of nephrotoxic compounds [42]. HepG2 is a well-established in vitro human cell system to study liver toxicity [43]. We also used healthy human skin fibroblast (HSF) to extend the cytotoxicity profile. All three cell lines were cultured in DMEM (Gibco no. 61965-026, UK) supplemented with 10% FBS (Gibco no. 10500-064, UK). For the experiment, cells were seeded (8 × 10^3^ cells/100μL/well) in transparent 96-well plates (FALCON no. 353072, Corning, Durham, DC, USA) and cultured overnight. The next day, the medium was removed and replaced with a fresh one that contained the following: (1) dimethylsulfoxide (DMSO < 0.1%, vehicle control (Veh)); (2) increasing concentration of the compounds *cis*-**16a**/*trans*-**16a** (97:3), *cis*-**16b**/*trans*-**16b** (90:10), and *trans*-**16b** (0.205 × 10^−6^–50 × 10^−6^ μM, 2.5-fold dilution); (3) doxorubicin (DOX, 0.205 × 10^−6^–50 × 10^−6^ μM, 2.5-fold dilution). DOX was a reference compound that adversely affected the liver, kidney, and healthy cells [44,45]. Treatment with compounds was carried out for 72 h. Cell viability was examined using an MTS-based CellTiter96^®^ AQueous One Solution Cell Proliferation Assay (Promega, Madison, WI, USA) following the manufacturer’s protocol.

#### 3.4.3. Apoptosis Assay

An IncuCyte S3 live cell imaging system (Essen Bioscience, Ann Arbor, MI, USA) was used to kinetically monitor the apoptotic activity of the most active compound trans-13b in the PC3 cell line. Apoptosis was monitored in real time using the Incucyte^®^ Caspase-3/7 for Metabolism Dyes (Cat. No. 4776, Sartorius, Ann Arbor, MI, USA). The principle of this test is based on binding a DNA intercalating dye with the activation motif (DEVD) of caspase-3/7, allowing a quantitative analysis of cells to undergo caspase-3/7-mediated apoptosis in real time. For the experiment, PC3 cells were seeded in a black 96-well plate with transparent bottom (cat. no. 165305, ThermoScientific, Waltham, MA, USA) at 10,000 cells/well density and cultured overnight. The next day, the medium was removed, and the cells were treated with the tested compound. The compound was prepared in a complete culture medium containing apoptosis reagent diluted 1000× for a final assay concentration of 1 μM. The IncuCyte S3 imaging system recorded kinetic measures of the number of caspase-3/7 positive cells. Repeat scanning to record phase and fluorescence images were every 2 h, for up to 28 h. Objective 10× and 800 ms acquisition were applied.

## 4. Conclusions

The *cis* and *trans* compounds **16a**–**g** were efficiently synthesized from *N*-benzyl-*C*-(dibenzyloxyphosphoryl)nitrone **17** and selected *N*^1^-allyl-*N*^3^-benzylquinazoline-2,4-diones **18a**–**g** with good yields (61–69%) and low to moderate diastereoselectivities (d.e. 16–34%). The relative configurations in the (isoxazolidine)phosphonates *cis*-**16a**–**g** and *trans*-**16a**–**g** were established based on the analysis of the 2D NOE experiments performed for *cis*-**16a** and *trans*-**16a**.

Among all the tested compounds, isoxazolidines *trans*-**16a** and *trans*-**16b** and mixtures of isoxazolidines enriched in minor *cis*-isomer, i.e., *cis*-**16a**/*trans*-**16a** (97:3) and *cis*-**16b**/*trans*-**16b** (90:10), exhibited the highest inhibitory properties towards the growth of the prostate cancer cell line (PC-3) with IC_50_’s in the 9.84 ± 3.69–12.67 ± 3.45 μM range, while the mixture of isoxazolidines *cis*-**16d**/*trans*-**16d** (97:3) appeared the most active against the fibrosarcoma cell line (HT-1080) (IC_50_ = 10.36 ± 2.69 μM). For the most active compounds, namely *trans*-**16b** and mixtures of isoxazolidines *cis*-**16a**/*trans*-**16a** (97:3) and *cis*-**16b**/*trans*-**16b** (90:10), an apoptosis induction test and an assessment of toxicity were carried out. Isoxazolidine *trans*-**16b** strongly induced apoptosis at 10 μM in the PC-3 cell line. All the active compounds tested showed excellent safety profiles in three cellular models (HEK293, HepG2, and HSF).

Among the tested isoxazolidines, dibenzyl 5-((3-(2-fluorobenzyl)-3,4-dihydro-2,4-dioxoquinazolin-1(2*H*)-yl)methyl)-2-benzylisoxazolidin-3-yl-3-phosphonate *trans*-**16b** turned out to be the most promising cancer-cytotoxic and “drug-like” compound, which can serve as a new lead structure for both extended biological study and further modification in the search for effective anticancer drugs.

## Figures and Tables

**Table 1:** Cycloaddition of the nitrone **17** and *N*^1^-allyl-*N*^3^-benzylquinazoline-2,4-diones **18a**–**g**.

Entry	Alkene 18 (R)	Ratio of *cis*-16:*trans*-16	Yield (%)
**a**	H	35:65	*trans*-**16a** (27%) ^a^ + *cis*-**16a** and *trans*-**16a** (35%) ^b^
**b**	2-F	33:67	*trans*-**16b** (35%) ^a^ + *cis*-**16b** and *trans*-**16b** (33%) ^b^
**c**	3-F	39:61	*trans*-**16c** (34%) ^a^ + *cis*-**16c** and *trans*-**16c** (35%) ^b^
**d**	4-F	39:61	*trans*-**16d** (39%) ^a^ + *cis*-**16d** and *trans*-**16d** (30%) ^b^
**e**	2-NO_2_	39:61	*trans*-**16e** (17%) ^a^ + *cis*-**16e** and *trans*-**16e** (44%) ^b^
**f**	3-NO_2_	42:58	*trans*-**16f** (38%) ^a^ + *cis*-**16f** and *trans*-**16f** (28%) ^b^
**g**	4-NO_2_	40:60	*trans*-**16g** (26%) ^a^ + *cis*-**16g** and *trans*-**16g** (39%) ^b^

^a^ yield of pure *trans*-isomer. ^b^ yield of a pure mixture of *cis*- and *trans*-isomers; the ^1^H and ^13^P NMR spectra of the respective fractions were analyzed to determine the purity and ratio of isomers.

**Table 2:** Inhibitory effects of the tested compounds against the proliferation of breast cancer cells (MCF-7), fibrosarcoma cells (HT-1080), and prostate cancer cells (PC-3).

Compound	IC_50_ ± SEM [μM] ^a^		
	MCF-7	HT-1080	PC-3
*cis*-**16a**/*trans*-**16a** (97:3)	90.33 ± 4.57	19.94 ± 8.13	12.64 ± 5.56
*trans*-**16a**	96.04 ± 4.66	40.45 ± 5.44	12.67 ± 3.45
*cis*-**16b**/*trans*-**16b** (90:10)	103.69 ± 7.38	27.29 ± 5.43	11.21 ± 1.99
*trans*-**16b**	78.66 ± 2.35	34.56 ± 5.30	9.84 ± 3.69
*cis*-**16c**/*trans*-**16c** (90:10)	237.55 ± 20.72	20.47 ± 1.56	17.64 ± 6.21
*trans*-**16c**	130.35 ± 9.97	42.34 ± 3.48	16.37 ± 4.32
*cis*-**16d**/*trans*-**16d** (96:4)	116.45 ± 5.73	10.36 ± 2.69	16.43 ± 3.69
*trans*-**16d**	88.89 ± 3.86	35.62 ± 3.03	13.93 ± 2.14
*cis*-**16e**/*trans*-**16e** (70:30)	59.08 ± 3.77	59.60 ± 0.36	26.57 ± 4.69
*trans*-**16e**	57.87 ± 8.36	29.80 ± 4.75	26.58 ± 1.09
*cis*-**16f**/*trans*-**16f** (96:4)	91.68 ± 1.47	17.07 ± 5.73	24.80 ± 2.15
*trans*-**16f**	59.40 ± 0.78	23.08 ± 9.22	18.14 ± 0.98
*cis*-**16g**/*trans*-**16g** (88:12)	116.45 ± 9.69	16.45 ± 2.03	21.51 ± 4.63
*trans*-**16g**	142.49 ± 5.11	16.64 ± 3.11	16.68 ± 3.48

^a^ 50% inhibitory concentration or compound required to inhibit tumor cell proliferation by 50%. IC_50_ values were calculated by fitting a non-linear regression to a sigmoidal dose–response curve in GraphPad Prism version 8.0.1.

## Data Availability

Data are contained within the article.

## Supplementary Materials

The following are available online at https://www.mdpi.com/article/10.3390/molecules29133050/s1, Figure S1: ^1^H NMR spectrum for **18e** in CDCl_3_ and expanded spectral regions; Figure S2: ^13^C NMR spectrum for **18e** in CDCl_3_ and expanded spectral regions; Figure S3: ^1^H NMR spectrum for **18f** in CDCl_3_ and expanded spectral regions; Figure S4: ^13^C NMR spectrum for **18f** in CDCl_3_ and expanded spectral regions; Figure S5: ^1^H NMR spectrum for **18g** in CDCl_3_ and expanded spectral regions; Figure S6: ^13^C NMR spectrum for **18g** in CDCl_3_ and expanded spectral regions; Figure S7: ^31^P NMR spectrum of the raw product of the synthesis of isoxazolidines *cis*-**16a**/*trans*-**16a** in CDCl_3_; Figure S8: ^31^P NMR spectrum of the raw product of the synthesis of isoxazolidines *cis*-**16b**/*trans*-**16b** in CDCl_3_; Figure S9: ^31^P NMR spectrum of the raw product of the synthesis of isoxazolidines *cis*-**16c**/*trans*-**16c** in CDCl_3_; Figure S10: ^31^P NMR spectrum of the raw product of the synthesis of isoxazolidines *cis*-**16d**/*trans*-**16d** in CDCl_3_; Figure S11: ^31^P NMR spectrum of the raw product of the synthesis of isoxazolidines *cis*-**16e**/*trans*-**16e** in CDCl_3_; Figure S12: ^31^P NMR spectrum of the raw product of the synthesis of isoxazolidines *cis*-**16f**/*trans*-**16f** in CDCl_3_; Figure S13: ^31^P NMR spectrum of the raw product of the synthesis of isoxazolidines *cis*-**16g**/*trans*-**16g** in CDCl_3_; Figure S14: ^1^H NMR spectrum for mixture of *cis*-**16a**/*trans*-**16a** (97:3) in CDCl_3_ and expanded spectral regions; Figure S15: ^31^P NMR spectrum for mixture of *cis*-**16a**/*trans*-**16a** (97:3) in CDCl_3_; Figure S16: ^13^C NMR spectrum for mixture of *cis*-**16a**/*trans*-**16a** (97:3) in CDCl_3_ and expanded spectral regions; Figure S17: HPLC chromatogram for mixture of *cis*-**16a**/*trans*-**16a** (97:3); Figure S18: ^1^H NMR spectrum for *trans*-**16a** in CDCl_3_ and expanded spectral regions; Figure S19: ^31^P NMR spectrum for *trans*-**16a** in CDCl_3_; Figure S20: ^13^C NMR spectrum for *trans*-**16a** in CDCl_3_ and expanded spectral regions; Figure S21: HPLC chromatogram for *trans*-**16a**; Figure S22: ^1^H NMR spectrum for mixture of *cis*-**16b**/*trans*-**16b** (90:10) in CDCl_3_ and expanded spectral regions; Figure S23: ^31^P NMR spectrum for mixture of *cis*-**16b**/*trans*-**16b** (90:10) in CDCl_3_; Figure S24: ^13^C NMR spectrum for mixture of *cis*-**16b**/*trans*-**16b** (90:10) in CDCl_3_ and expanded spectral regions; Figure S25: HPLC chromatogram for mixture of *cis*-**16b**/*trans*-**16b** (90:10); Figure S26: ^1^H NMR spectrum for *trans*-**16b** in CDCl_3_ and expanded spectral regions; Figure S27: ^31^P NMR spectrum for *trans*-**16b** in CDCl_3_; Figure S28: ^13^C NMR spectrum for *trans*-**16b** in CDCl_3_ and expanded spectral regions; Figure S29: HPLC chromatogram for *trans*-**16b**; Figure S30: ^1^H NMR spectrum for mixture of *cis*-**16c**/*trans*-**16c** (90:10) in CDCl_3_ and expanded spectral regions; Figure S31: ^31^P NMR spectrum for mixture of *cis*-**16c**/*trans*-**16c** (90:10) in CDCl_3_; Figure S32: ^13^C NMR spectrum for mixture of *cis*-**16c**/*trans*-**16c** (90:10) in CDCl_3_ and expanded spectral regions; Figure S33: HPLC chromatogram for mixture of *cis*-**16c**/*trans*-**16c** (90:10); Figure S34: ^1^H NMR spectrum for *trans*-**16c** in CDCl_3_ and expanded spectral regions; Figure S35: ^31^P NMR spectrum for *trans*-**16c** in CDCl_3_; Figure S36: ^13^C NMR spectrum for *trans*-**16c** in CDCl_3_ and expanded spectral regions; Figure S37: HPLC chromatogram for *trans*-**16c**; Figure S38: ^1^H NMR spectrum for mixture of *cis*-**16d**/*trans*-**16d** (96:4) in CDCl_3_ and expanded spectral regions; Figure S39: ^31^P NMR spectrum for mixture of *cis*-**16d**/*trans*-**16d** (96:4) in CDCl_3_; Figure S40: ^13^C NMR spectrum for mixture of *cis*-**16d**/*trans*-**16d** (96:4) in CDCl_3_ and expanded spectral regions; Figure S41: HPLC chromatogram for mixture of *cis*-**16d**/*trans*-**16d** (96:4); Figure S42: ^1^H NMR spectrum for *trans*-**16d** in CDCl_3_ and expanded spectral regions; Figure S43: ^31^P NMR spectrum for *trans*-**16d** in CDCl_3_; Figure S44: ^13^C NMR spectrum for *trans*-**16d** in CDCl_3_ and expanded spectral regions; Figure S45: HPLC chromatogram for *trans*-**16d**; Figure S46: ^1^H NMR spectrum for mixture of *cis*-**16e**/*trans*-**16e** (70:30) in CDCl_3_ and expanded spectral regions; Figure S47: ^31^P NMR spectrum for mixture of *cis*-**16e**/*trans*-**16e** (70:30) in CDCl_3_; Figure S48: ^13^C NMR spectrum for mixture of *cis*-**16e**/*trans*-**16e** (70:30) in CDCl_3_ and expanded spectral regions; Figure S49: HPLC chromatogram for mixture of *cis*-**16e**/*trans*-**16e** (70:30); Figure S50: ^1^H NMR spectrum for *trans*-**16e** in CDCl_3_ and expanded spectral regions; Figure S51: ^31^P NMR spectrum for *trans*-**16e** in CDCl_3_; Figure S52: ^13^C NMR spectrum for *trans*-**16e** in CDCl_3_ and expanded spectral regions; Figure S53: HPLC chromatogram for *trans*-**16e**; Figure S54: ^1^H NMR spectrum for mixture of *cis*-**16f**/*trans*-**16f** (96:4) in CDCl_3_ and expanded spectral regions; Figure S55: ^31^P NMR spectrum for mixture of *cis*-**16f**/*trans*-**16f** (96:4) in CDCl_3_; Figure S56: ^13^C NMR spectrum for mixture of *cis*-**16f**/*trans*-**16f** (96:4) in CDCl_3_ and expanded spectral regions; Figure S57: HPLC chromatogram for mixture of *cis*-**16f**/*trans*-**16f** (96:4); Figure S58: ^1^H NMR spectrum for *trans*-**16f** in CDCl_3_ and expanded spectral regions; Figure S59: ^31^P NMR spectrum for *trans*-**16f** in CDCl_3_; Figure S60: ^13^C NMR spectrum for *trans*-**16f** in CDCl_3_ and expanded spectral regions; Figure S61: HPLC chromatogram for *trans*-**16f**; Figure S62: ^1^H NMR spectrum for mixture of *cis*-**16g**/*trans*-**16g** (88:12) in CDCl_3_ and expanded spectral regions; Figure S63: ^31^P NMR spectrum for mixture of *cis*-**16g**/*trans*-**16g** (88:12) in CDCl_3_; Figure S64: ^13^C NMR spectrum for mixture of *cis*-**16g**/*trans*-**16g** (88:12) in CDCl_3_ and expanded spectral regions; Figure S65: HPLC chromatogram for mixture of *cis*-**16g**/*trans*-**16g** (88:12); Figure S66: ^1^H NMR spectrum for *trans*-**16g** in CDCl_3_ and expanded spectral regions; Figure S67: ^31^P NMR spectrum for *trans*-**16g** in CDCl_3_; Figure S68: ^13^C NMR spectrum for *trans*-**16g** in CDCl_3_ and expanded spectral regions; Figure S69: HPLC chromatogram for *trans*-**16g**; Figure S70: ^1^H–^1^H COSY spectrum for mixture of *cis*-**16a**/*trans*-**16a** (97:3) in CDCl_3_; Figure S71: NOESY spectrum for mixture of *cis*-**16a**/*trans*-**16a** (97:3) in CDCl_3_; Figure S72: ^1^H–^1^H COSY spectrum for *trans*-**16a** in CDCl_3_; Figure S73: NOESY spectrum for *trans*-**16a** in CDCl_3_; Table S1: ADMET properties in silico for compound *trans*-**16a**; Table S2: ADMET properties in silico for compound *cis*-**16a**; Table S3: ADMET properties in silico for compound *trans*-**16b**; Table S4: ADMET properties in silico for compound *cis*-**16b**; Table S5: ADMET properties in silico for compound *trans*-**16c**; Table S6: ADMET properties in silico for compound *cis*-**16c**; Table S7: ADMET properties in silico for compound *trans*-**16d**; Table S8: ADMET properties in silico for compound *cis*-**16d**; Table S9: ADMET properties in silico for compound *trans*-**16e**; Table S10: ADMET properties in silico for compound *cis*-**16e**; Table S11: ADMET properties in silico for compound *trans*-**16f**; Table S12: ADMET properties in silico for compound *cis*-**16f**; Table S13 ADMET properties in silico for compound *trans*-**16g**; Table S14: ADMET properties in silico for compound *cis*-**16g**; Table S15: ADMET properties in silico for Doxorubicin.
