# Supplementary material for: New 3-(Dibenzyloxyphosphoryl)isoxazolidine Conjugates of N1-Benzylated Quinazoline-2,4-diones as Potential Cytotoxic Agents against Cancer Cell Lines
Source: Molecules. 2024 Jun 27;29(13):3050. doi: 10.3390/molecules29133050 (PMC11243672; doi:10.3390/molecules29133050)
Supplement: Supplementary file 1 [file molecules-29-03050-s001.zip › molecules-3035651-supplementary.pdf]

New 3-(Dibenzyloxyphosphoryl)isoxazolidine Conjugates of N1-Benzylated Quinazoline-2,4-diones as Potential Cytotoxic Agents against Cancer Cell Lines

Magdalena Łysakowska [1], Iwona E. Głowacka [1], Ewelina Honkisz-Orzechowska [2], Jadwiga Handzlik [2], and Dorota G. Piotrowska [1,*]

[1.] Bioorganic Chemistry Laboratory, Faculty of Pharmacy, Medical University of Lodz, 90-151 Lodz, Muszynskiego 1, Poland; magdalena.lysakowska@umed.lodz.pl (M.Ł.); iwona.glowacka@umed.lodz.pl (I.E.G.)

[2.] Department of Technology and Biotechnology of Drugs, Faculty of Pharmacy, Jagiellonian University, Medical College, Medyczna 9, 30-688 Krakow, Poland; ewelina.honkisz@uj.edu.pl (E.H.-O.); j.handzlik@uj.edu.pl (J.H.)

* Correspondence: dorota.piotrowska@umed.lodz.pl (D.G.P.)

Contents

NMR spectra of N-allylquinazoline-2,4-diones **18e-g** and NMR spectra and chromatograms for compounds *trans*-**16a-16g**, and mixture *cis*/*trans* **16e-16g** as well as Tables with ADMET properties *in silico* for **16a-16g** and reference doxorubicin tested using pkCSM (https://biosig.lab.uq.edu.au/pkcsm/prediction):

Figure S1: ^{1}H NMR Spectrum for **18e** in $CDCl_3$ and expanded spectral regions

Figure S2: ^{13}C NMR Spectrum for **18e** in $CDCl_3$ and expanded spectral regions

Figure S3: ^{1}H NMR Spectrum for **18f** in $CDCl_3$ and expanded spectral regions

Figure S4: ^{13}C NMR Spectrum for **18f** in $CDCl_3$ and expanded spectral regions

Figure S5: ^{1}H NMR Spectrum for **18g** in $CDCl_3$ and expanded spectral regions

Figure S6: ^{13}C NMR Spectrum for **18g** in $CDCl_3$ and expanded spectral regions

Figure S7: ^{31}P NMR Spectrum of the raw product of the synthesis of isoxazolidines *cis*-**16a**/*trans*-**16a** in $CDCl_3$

Figure S8: ^{31}P NMR Spectrum of the raw product of the synthesis of isoxazolidines *cis*-**16b**/*trans*-**16b** in $CDCl_3$

Figure S9: ^{31}P NMR Spectrum of the raw product of the synthesis of isoxazolidines *cis*-**16c**/*trans*-**16c** in $CDCl_3$

Figure S10: ^{31}P NMR Spectrum of the raw product of the synthesis of isoxazolidines *cis*-**16d**/*trans*-**16d** in $CDCl_3$

Figure S11: ^{31}P NMR Spectrum of the raw product of the synthesis of isoxazolidines *cis*-**16e**/*trans*-**16e** in $CDCl_3$

Figure S12: ^{31}P NMR Spectrum of the raw product of the synthesis of isoxazolidines *cis*-**16f**/*trans*-**16f** in $CDCl_3$

Figure S13: ^{31}P NMR Spectrum of the raw product of the synthesis of isoxazolidines *cis*-**16g**/*trans*-**16g** in $CDCl_3$

Figure S14: ^{1}H NMR Spectrum for mixture of *cis*-**16a**/*trans*-**16a** (97:3) in $CDCl_3$ and expanded spectral regions

Figure S15: ^{31}P NMR Spectrum for mixture of *cis*-**16a**/*trans*-**16a** (97:3) in $CDCl_3$

Figure S16: ^{13}C NMR Spectrum for mixture of *cis*-**16a**/*trans*-**16a** (97:3) in $CDCl_3$ and expanded spectral regions

Figure S17: HPLC chromatogram for mixture of *cis*-**16a**/*trans*-**16a** (97:3)

Figure S18: ^{1}H NMR Spectrum for *trans*-**16a** in $CDCl_3$

Figure S19: ^{31}P NMR Spectrum for *trans*-**16a** in $CDCl_3$

Figure S20: ^{13}C NMR Spectrum for *trans*-**16a** in $CDCl_3$ and expanded spectral regions

Figure S21: HPLC chromatogram for *trans*-**16a**

Figure S22: ^{1}H NMR Spectrum for mixture of *cis*-**16b**/*trans*-**16b** (90:10) in $CDCl_3$ and expanded spectral regions

Figure S23: ^{31}P NMR Spectrum for mixture of *cis*-**16b**/*trans*-**16b** (90:10) in $CDCl_3$

Figure S24: ^{13}C NMR Spectrum for mixture of *cis*-**16b**/*trans*-**16b** (90:10) in $CDCl_3$ and expanded spectral regions

Figure S25: HPLC chromatogram for mixture of *cis*-**16b**/*trans*-**16b** (90:10)

Figure S26: ^{1}H NMR Spectrum for *trans*-**16b** in $CDCl_3$ and expanded spectral regions

Figure S27: ^{31}P NMR Spectrum for *trans*-**16b**in $CDCl_3$

Figure S28: ^{13}C NMR Spectrum for *trans*-**16b** in $CDCl_3$ and expanded spectral regions

Figure S29: HPLC chromatogram for *trans*-**16b**

Figure S30: ^{1}H NMR Spectrum for mixture of *cis*-**16c**/*trans*-**16c** (90:10) in $CDCl_3$ and expanded spectral regions

Figure S31: ^{31}P NMR Spectrum for mixture of *cis*-**16c**/*trans*-**16c** (90:10) in $CDCl_3$

Figure S32: ^{13}C NMR Spectrum for mixture of *cis*-**16c**/*trans*-**16c** (90:10) in $CDCl_3$ and expanded spectral regions

Figure S33: HPLC chromatogram for mixture of *cis*-**16c**/*trans*-**16c** (90:10)

Figure S34: ^{1}H NMR Spectrum for *trans*-**16c** in $CDCl_3$ and expanded spectral regions

Figure S35: ^{31}P NMR Spectrum for *trans*-**16c** in $CDCl_3$

Figure S36: ^{13}C NMR Spectrum for *trans*-**16c** in $CDCl_3$ and expanded spectral regions

Figure S37: HPLC chromatogram for *trans*-**16c**

Figure S38: ^{1}H NMR Spectrum for mixture of *cis*-**16d**/*trans*-**16d** (96:4) in $CDCl_3$ and expanded spectral regions

Figure S39: ^{31}P NMR Spectrum for mixture of *cis*-**16d**/*trans*-**16d** (96:4) in $CDCl_3$

Figure S40: ^{13}C NMR Spectrum for mixture of *cis*-**16d**/*trans*-**16d** (96:4) in $CDCl_3$ and expanded spectral regions

Figure S41: HPLC chromatogram for mixture of *cis*-**16d**/*trans*-**16d** (96:4)

Figure S42: ^{1}H NMR Spectrum for *trans*-**16d** in $CDCl_3$ and expanded spectral regions

Figure S43: ^{31}P NMR Spectrum for *trans*-**16d** in $CDCl_3$

Figure S44: ^{13}C NMR Spectrum for *trans*-**16d** in $CDCl_3$ and expanded spectral regions

Figure S45: HPLC chromatogram for *trans*-**16d**

Figure S46: ^{1}H NMR Spectrum for mixture of *cis*-**16e**/*trans*-**16e** (70:30) in $CDCl_3$ and expanded spectral regions

Figure S47: ^{31}P NMR Spectrum for mixture of *cis*-**16e**/*trans*-**16e** (70:30) in $CDCl_3$

Figure S48: ^{13}C NMR Spectrum for mixture of *cis*-**16e**/*trans*-**16e** (70:30) in $CDCl_3$ and expanded spectral regions

Figure S49: HPLC chromatogram for mixture of *cis*-**16e**/*trans*-**16e** (70:30)

Figure S50: ^{1}H NMR Spectrum for *trans*-**16e** in $CDCl_3$ and expanded spectral regions

Figure S51: ^{31}P NMR Spectrum for *trans*-**16e** in $CDCl_3$

Figure S52: ^{13}C NMR Spectrum for *trans*-**16e** in $CDCl_3$ and expanded spectral regions

Figure S53: HPLC chromatogram for *trans*-**16e**

Figure S54: ^{1}H NMR Spectrum for mixture of *cis*-**16f**/*trans*-**16f** (96:4) in $CDCl_3$ and expanded spectral regions

Figure S55: ^{31}P NMR Spectrum for mixture of *cis*-**16f**/*trans*-**16f** (96:4) in $CDCl_3$

Figure S56: ^{13}C NMR Spectrum for mixture of *cis*-**16f**/*trans*-**16f** (96:4) in $CDCl_3$ and expanded spectral regions

Figure S57: HPLC chromatogram for mixture of *cis*-**16f**/*trans*-**16f** (96:4)

Figure S58: ^{1}H NMR Spectrum for *trans*-**16f** in $CDCl_3$ and expanded spectral regions

Figure S59: ^{31}P NMR Spectrum for *trans*-**16f** in $CDCl_3$

Figure S60: ^{13}C NMR Spectrum for *trans*-**16f** in $CDCl_3$ and expanded spectral regions

Figure S61: HPLC chromatogram for *trans*-**16f**

Figure S62: ^{1}H NMR Spectrum for mixture of *cis*-**16g**/*trans*-**16g** (88:12) in $CDCl_3$ and expanded spectral regions

Figure S63: ^{31}P NMR Spectrum for mixture of *cis*-**16g**/*trans*-**16g** (88:12) in $CDCl_3$

Figure S64: ^{13}C NMR Spectrum for mixture of *cis*-**16g**/*trans*-**16g** (88:12) in $CDCl_3$ and expanded spectral regions

Figure S65: HPLC chromatogram for mixture of *cis*-**16g**/*trans*-**16g** (88:12)

Figure S66: ^{1}H NMR Spectrum for *trans*-**16g** in $CDCl_3$ and expanded spectral regions

Figure S67: ^{31}P NMR Spectrum for *trans*-**16g** in $CDCl_3$

Figure S68: ^{13}C NMR Spectrum for *trans*-**16g** in $CDCl_3$ and expanded spectral regions

Figure S69: HPLC chromatogram for *trans*-**16g**

Figure S70: ^{1}H–^{1}H COSY Spectrum for mixture of *cis*-**16a**/*trans*-**16a** (97:3) in $CDCl_3$

Figure S71: NOESY Spectrum for mixture of *cis*-**16a**/*trans*-**16a** (97:3) in $CDCl_3$

Figure S72: ^{1}H–^{1}H COSY Spectrum for mixture of *trans*-**16a** in $CDCl_3$

Figure S73: NOESY Spectrum for mixture of *trans*-**16a** in $CDCl_3$

Table S1: ADMET properties in silico for compound *trans*-**16a**

Table S2: ADMET properties in silico for compound *cis*-**16a**

Table S3: ADMET properties in silico for compound *trans*-**16b**

Table S4: ADMET properties in silico for compound *cis*-**16b**

Table S5: ADMET properties in silico for compound *trans*-**16c**

Table S6: ADMET properties in silico for compound *cis*-**16c**

Table S7: ADMET properties in silico for compound *trans*-**16d**

Table S8: ADMET properties in silico for compound *cis*-**16d**

Table S9: ADMET properties in silico for compound *trans*-**16e**

Table S10: ADMET properties in silico for compound *cis*-**16e**

Table S11: ADMET properties in silico for compound *trans*-**16f**

Table S12: ADMET properties in silico for compound *cis*-**16f**

Table S13 ADMET properties in silico for compound *trans*-**16g**

Table S14: ADMET properties in silico for compound *cis*-**16g**

Table S15: ADMET properties in silico for Doxorubicin

Figure S1: ^{1}H NMR Spectrum for **18e** in $CDCl_3$ and expanded spectral regions

SpinWorks 4: no title

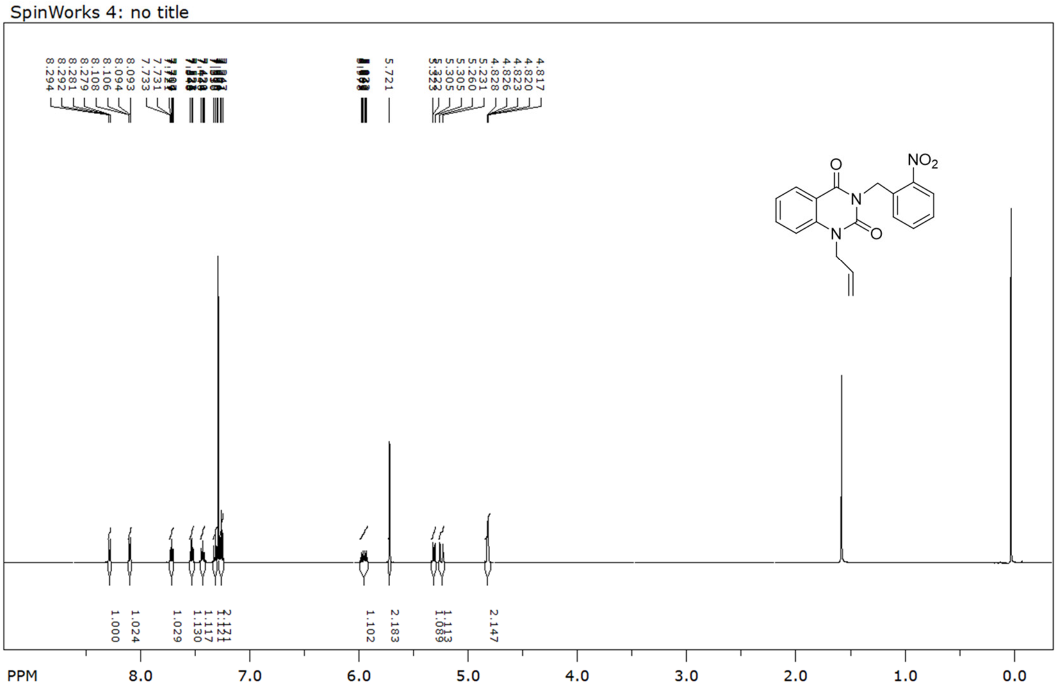

file: ... Łodzi\Desktop\nmr\mfl-2870\10\fid expt: <zg30>
transmitter freq.: 600.263707 MHz
time domain size: 65536 points
width: 12335.53 Hz = 20.5502 ppm = 0.188225 Hz/pt
number of scans: 16

freq. of 0 ppm: 600.260000 MHz
processed size: 32768 complex points
LB: 0.300 GF: 0.0000

SpinWorks 4: no title

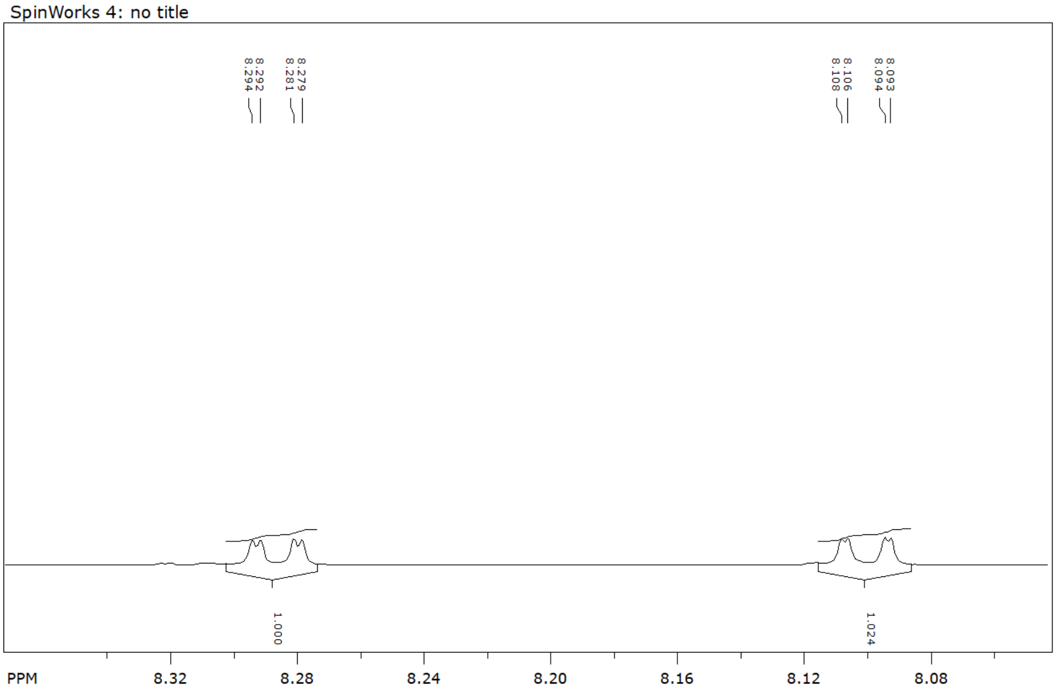

file: ... Łodzi\Desktop\nmr\mfl-2870\10\fid expt: <zg30>
transmitter freq.: 600.263707 MHz
time domain size: 65536 points
width: 12335.53 Hz = 20.5502 ppm = 0.188225 Hz/pt
number of scans: 16

freq. of 0 ppm: 600.260000 MHz
processed size: 32768 complex points
LB: 0.300 GF: 0.0000

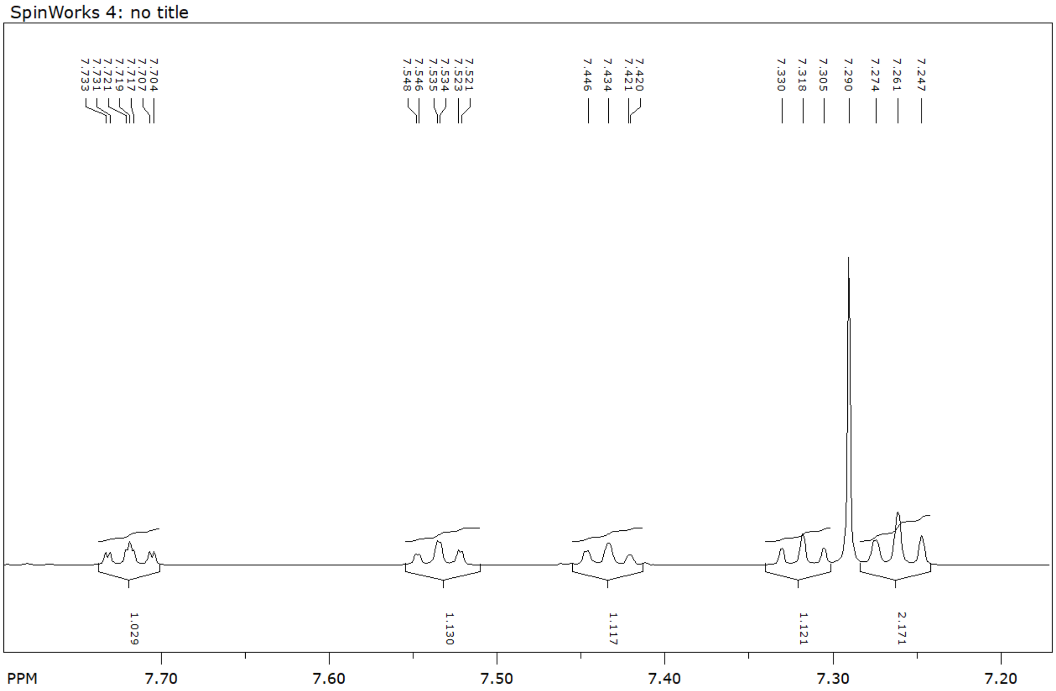

file: ... Łodzi\Desktop\nmr\mfl-2870\10\fid expt: <zg30>
transmitter freq.: 600.263707 MHz
time domain size: 65536 points
width: 12335.53 Hz = 20.5502 ppm = 0.188225 Hz/pt
number of scans: 16

freq. of 0 ppm: 600.260000 MHz
processed size: 32768 complex points
LB: 0.300 GF: 0.0000

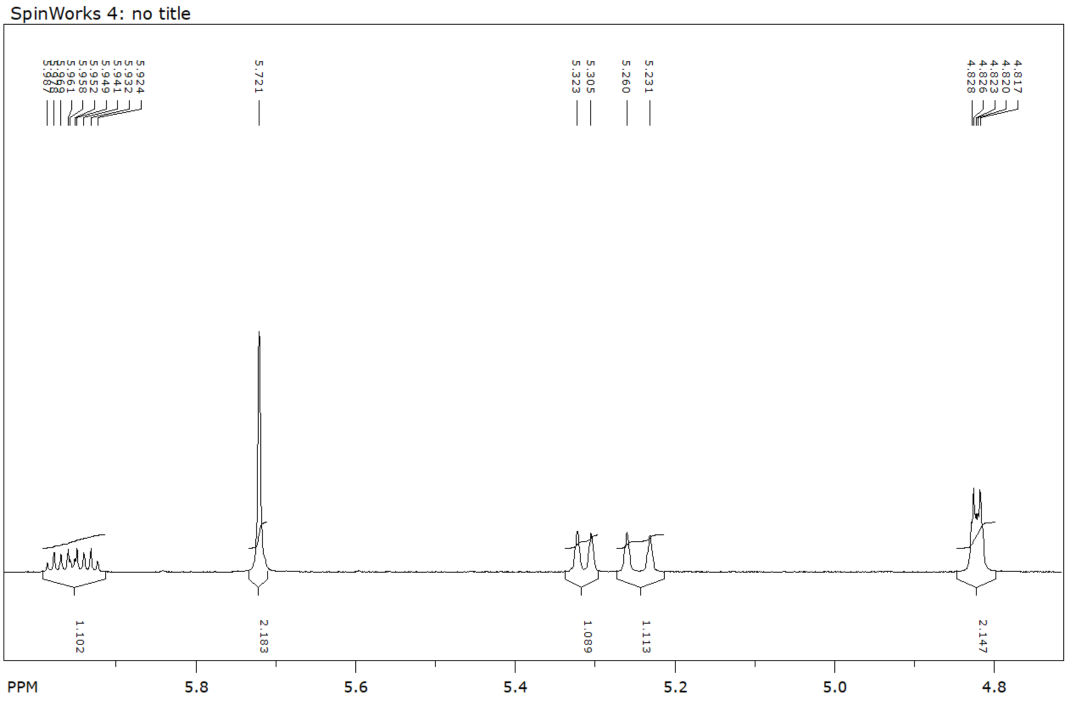

file: ... Łodzi\Desktop\nmr\mfl-2870\10\fid expt: <zg30>
transmitter freq.: 600.263707 MHz
time domain size: 65536 points
width: 12335.53 Hz = 20.5502 ppm = 0.188225 Hz/pt
number of scans: 16

freq. of 0 ppm: 600.260000 MHz
processed size: 32768 complex points
LB: 0.300 GF: 0.0000

Figure S2: ^{13}C NMR Spectrum for **18e** in $CDCl_3$ and expanded spectral regions

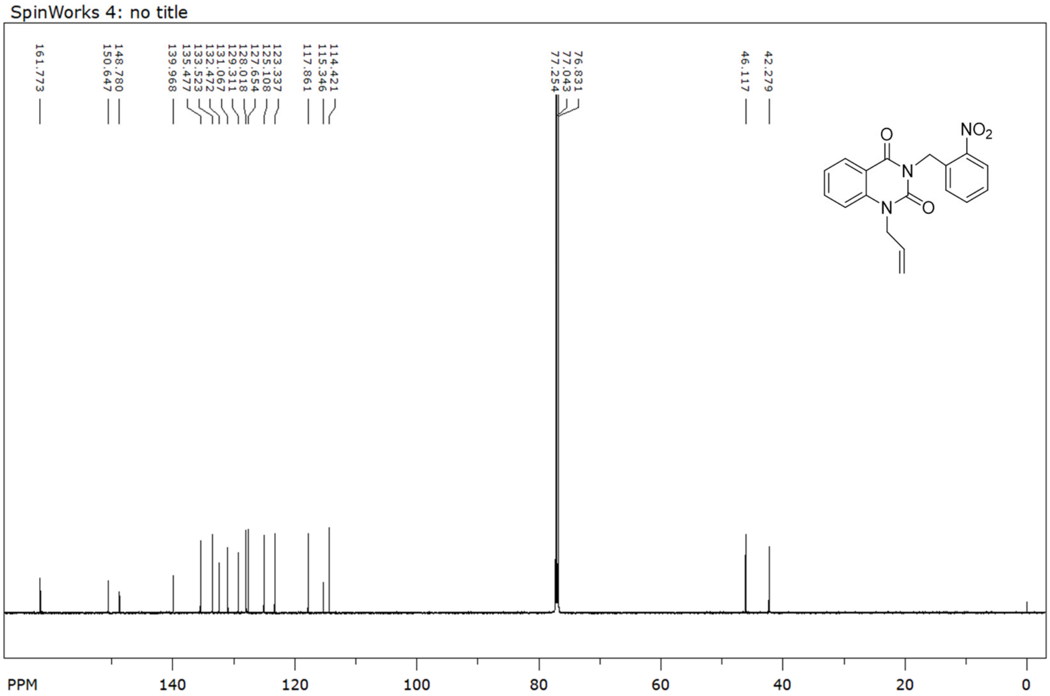

file: ... Łodzi\Desktop\nmr\mfl-2866\10\fid expt: <zgpg30>
transmitter freq.: 150.950591 MHz
time domain size: 65536 points
width: 36057.69 Hz = 238.8708 ppm = 0.550197 Hz/pt
number of scans: 7168

freq. of 0 ppm: 150.935497 MHz
processed size: 32768 complex points
LB: 1.000 GF: 0.0000

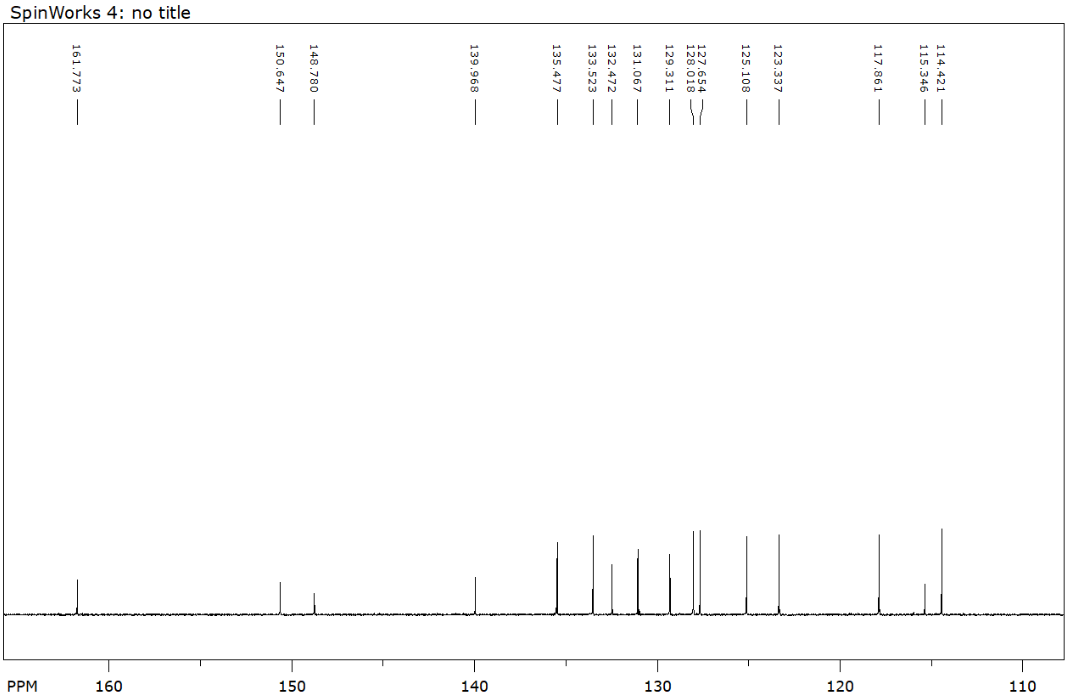

file: ... Łodzi\Desktop\nmr\mfl-2866\10\fid expt: <zgpg30>
transmitter freq.: 150.950591 MHz
time domain size: 65536 points
width: 36057.69 Hz = 238.8708 ppm = 0.550197 Hz/pt
number of scans: 7168

freq. of 0 ppm: 150.935497 MHz
processed size: 32768 complex points
LB: 1.000 GF: 0.0000

Figure S3: ^{1}H NMR Spectrum for **18f** in $CDCl_3$ and expanded spectral regions

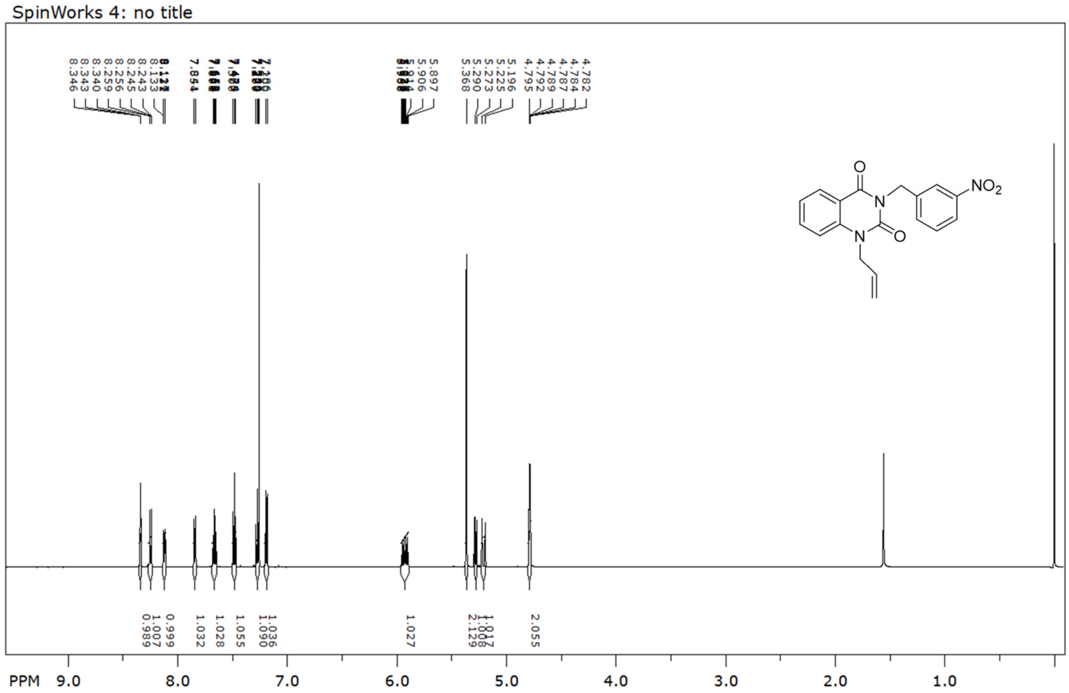

file: ... Łodzi\Desktop\nmr\mfl-2880\10\fid expt: <zg30>
transmitter freq.: 600.263707 MHz
time domain size: 65536 points
width: 12335.53 Hz = 20.5502 ppm = 0.188225 Hz/pt
number of scans: 16

freq. of 0 ppm: 600.260018 MHz
processed size: 32768 complex points
LB: 0.300 GF: 0.0000

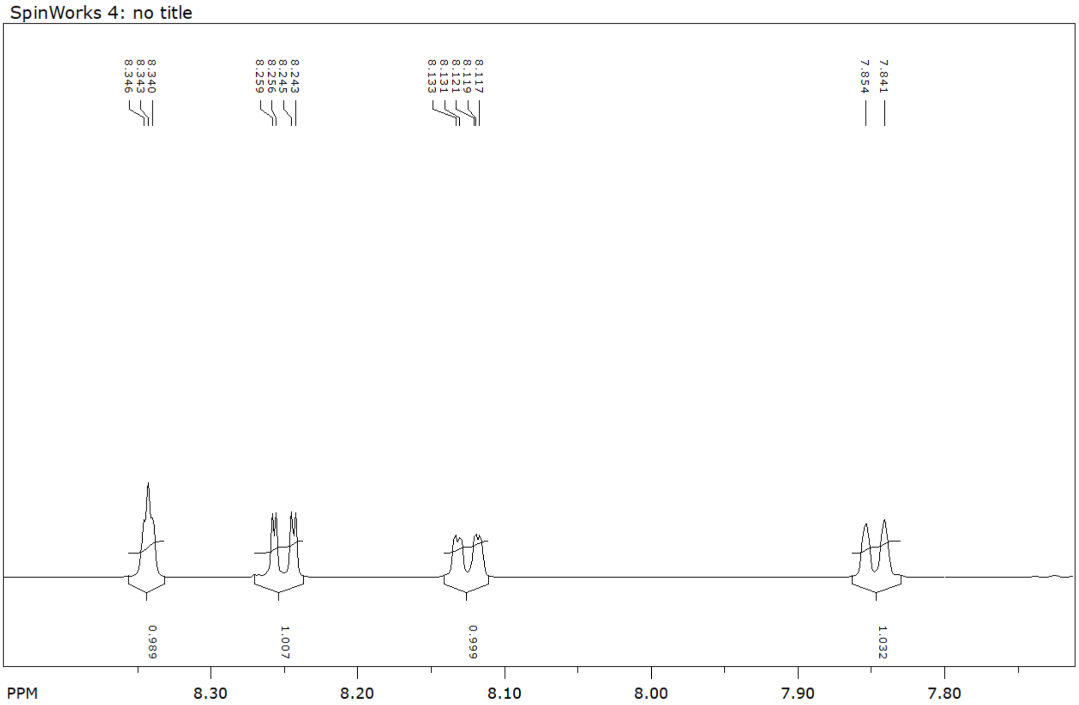

file: ... Łodzi\Desktop\nmr\mfl-2880\10\fid expt: <zg30>
transmitter freq.: 600.263707 MHz
time domain size: 65536 points
width: 12335.53 Hz = 20.5502 ppm = 0.188225 Hz/pt
number of scans: 16

freq. of 0 ppm: 600.260018 MHz
processed size: 32768 complex points
LB: 0.300 GF: 0.0000

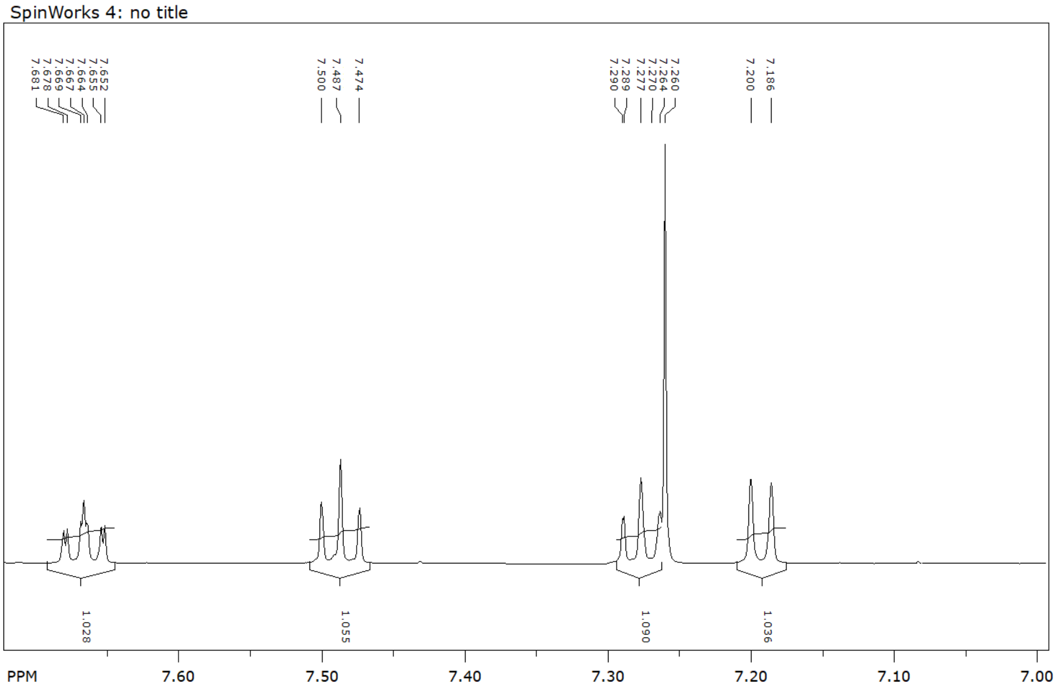

file: ... Łodzi\Desktop\nmr\mfl-2880\10\fid expt: <zg30>
transmitter freq.: 600.263707 MHz
time domain size: 65536 points
width: 12335.53 Hz = 20.5502 ppm = 0.188225 Hz/pt
number of scans: 16

freq. of 0 ppm: 600.260018 MHz
processed size: 32768 complex points
LB: 0.300 GF: 0.0000

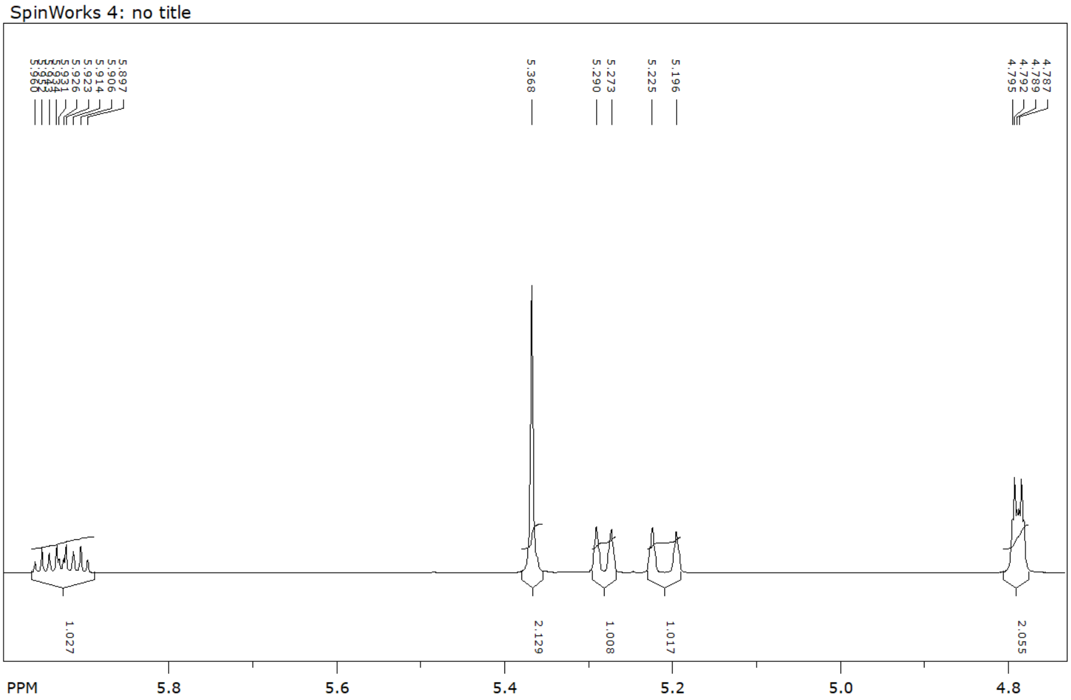

file: ... Łodzi\Desktop\nmr\mfl-2880\10\fid expt: <zg30>
transmitter freq.: 600.263707 MHz
time domain size: 65536 points
width: 12335.53 Hz = 20.5502 ppm = 0.188225 Hz/pt
number of scans: 16

freq. of 0 ppm: 600.260018 MHz
processed size: 32768 complex points
LB: 0.300 GF: 0.0000

Figure S4: ^{13}C NMR Spectrum for **18f** in $CDCl_3$ and expanded spectral regions

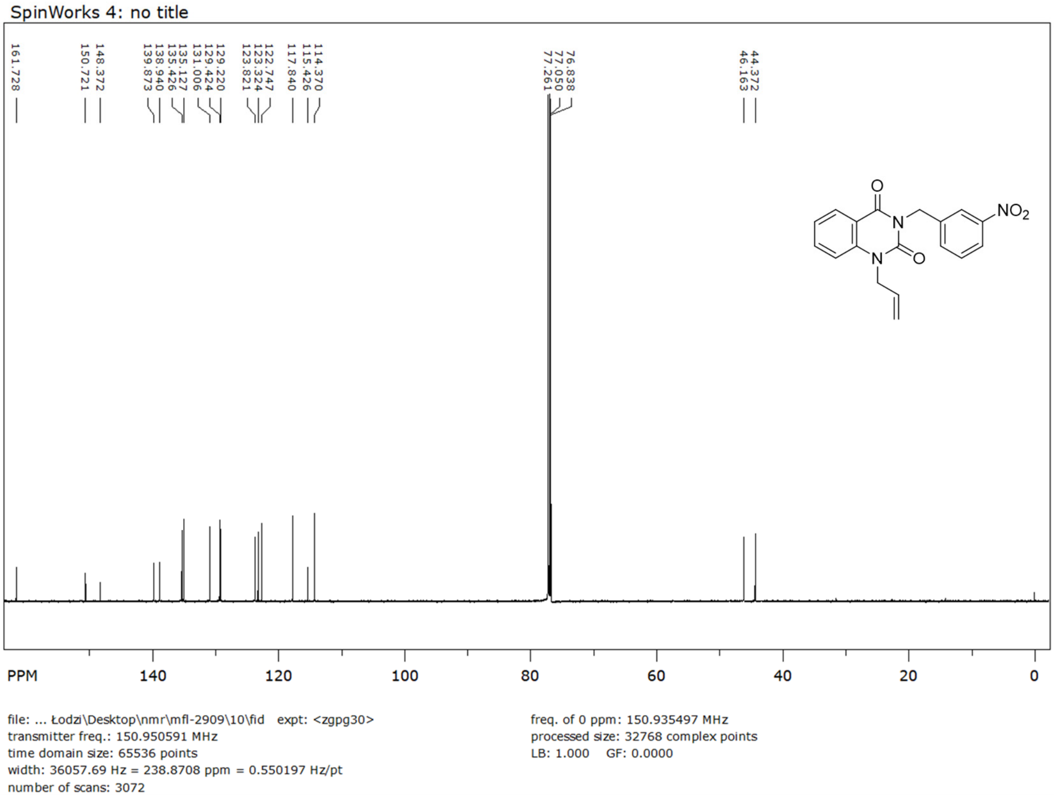

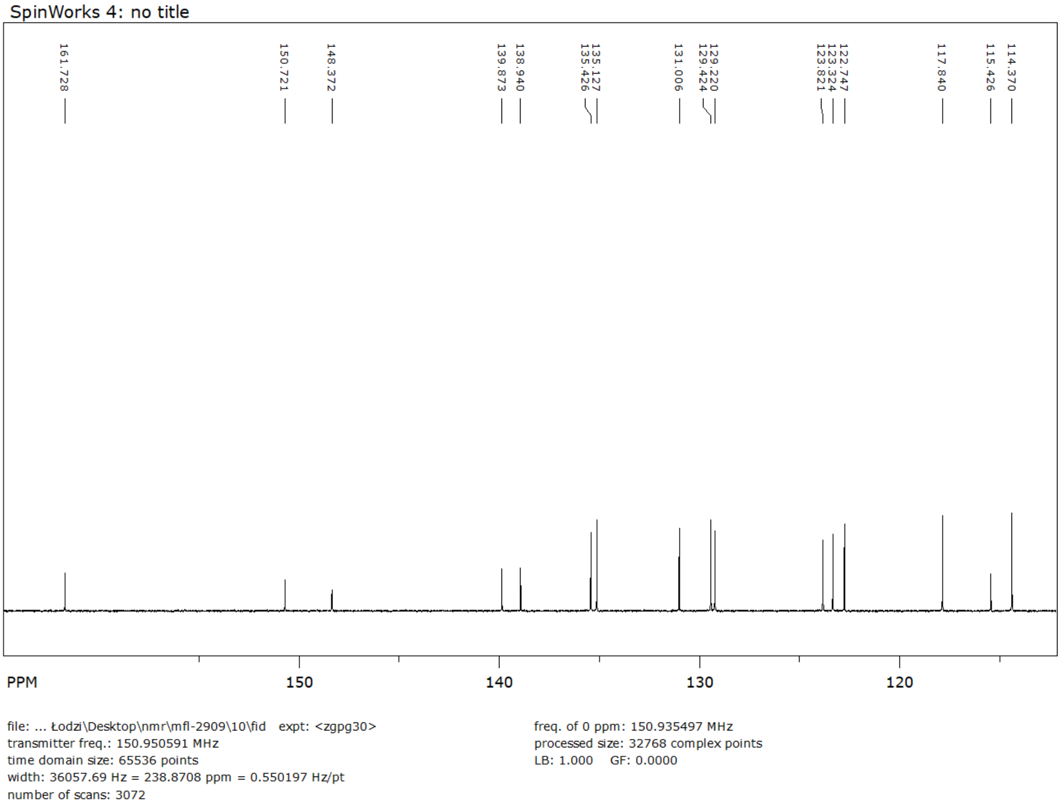

Figure S5: ^{1}H NMR Spectrum for **18g** in $CDCl_3$ and expanded spectral regions

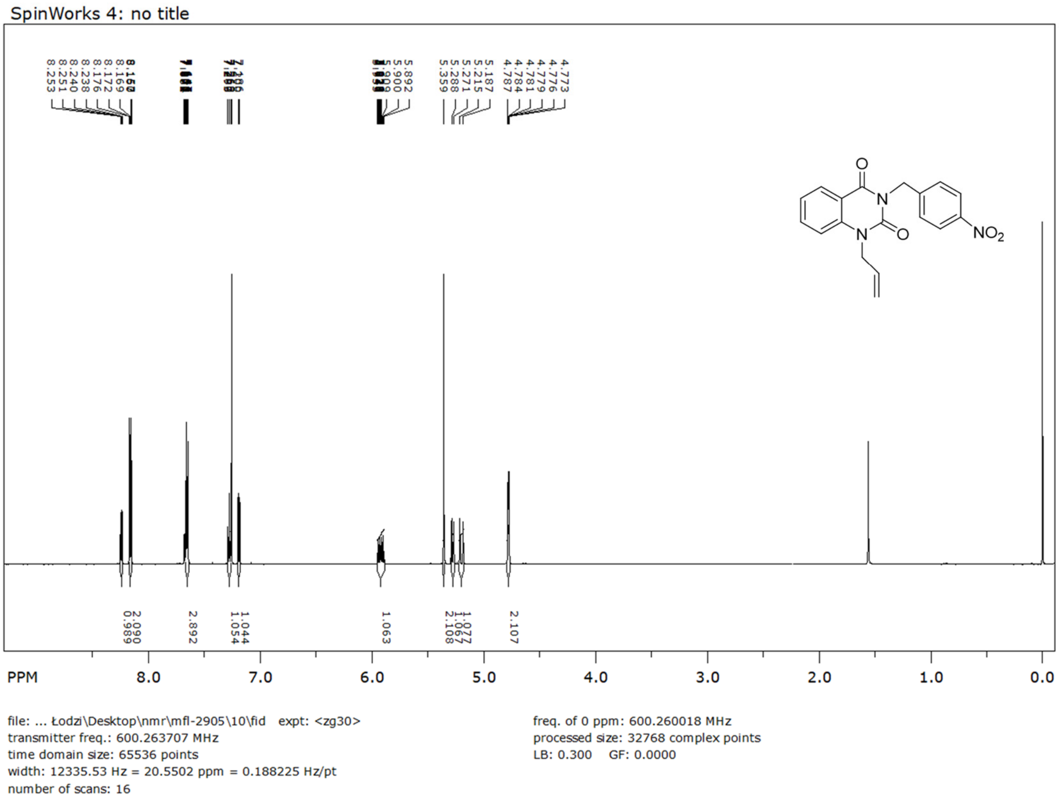

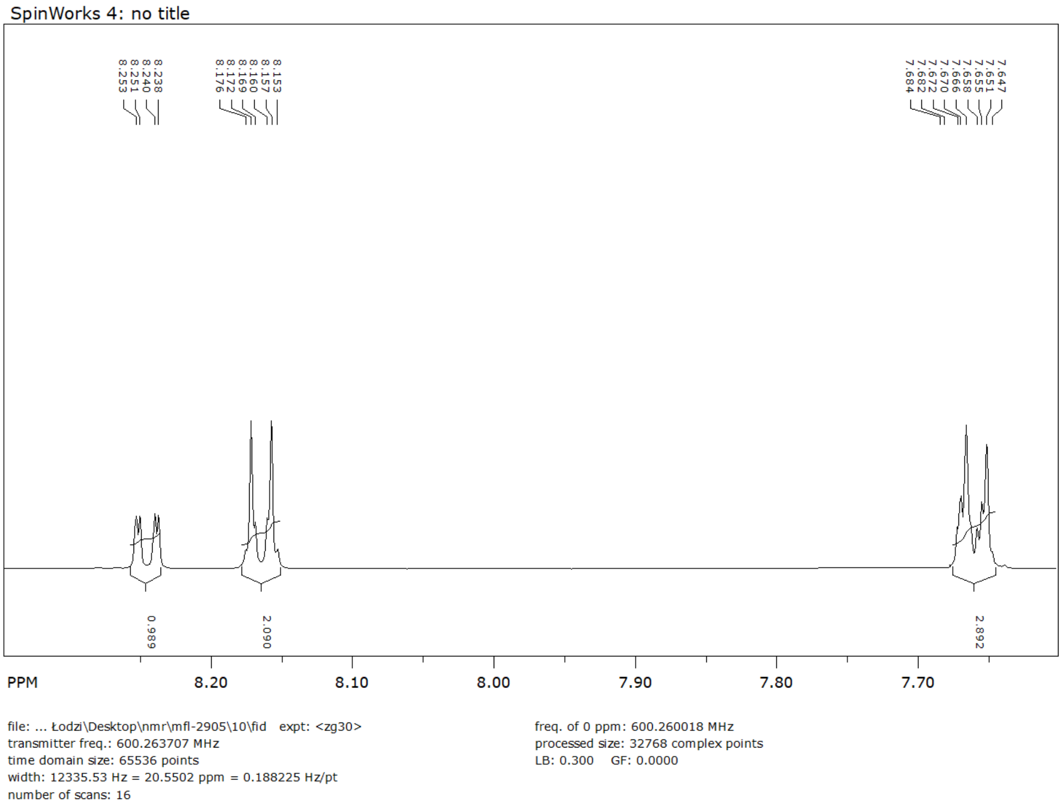

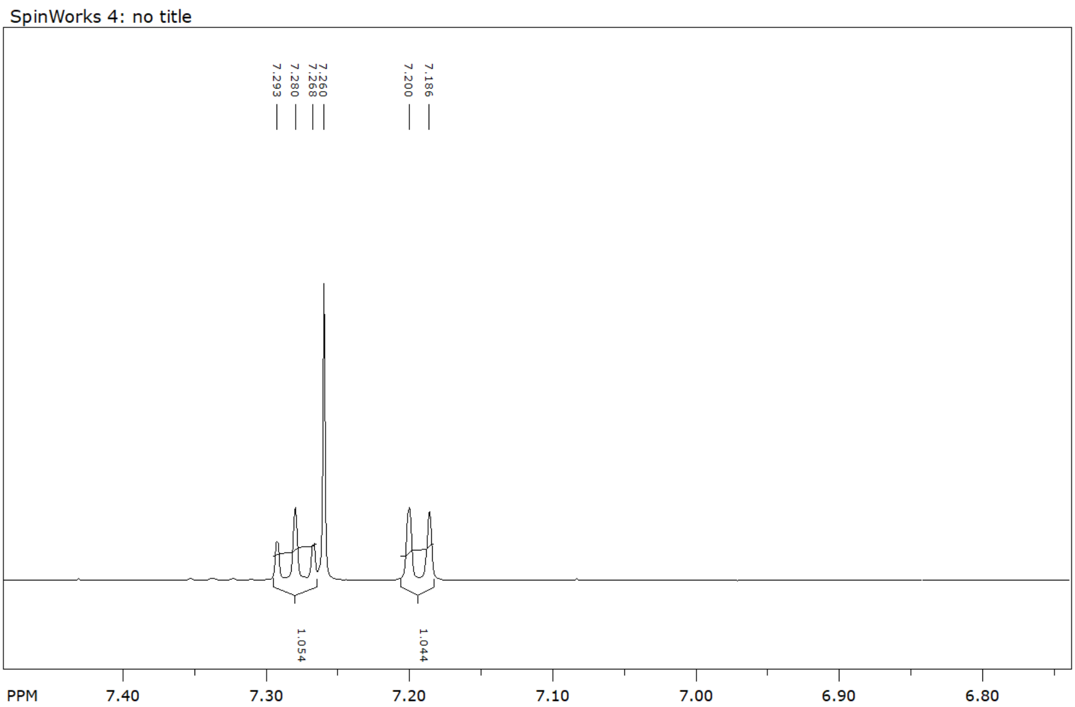

file: ... Łodzi\Desktop\nmr\mfl-2905\10\fid expt: <zg30>
transmitter freq.: 600.263707 MHz
time domain size: 65536 points
width: 12335.53 Hz = 20.5502 ppm = 0.188225 Hz/pt
number of scans: 16

freq. of 0 ppm: 600.260018 MHz
processed size: 32768 complex points
LB: 0.300 GF: 0.0000

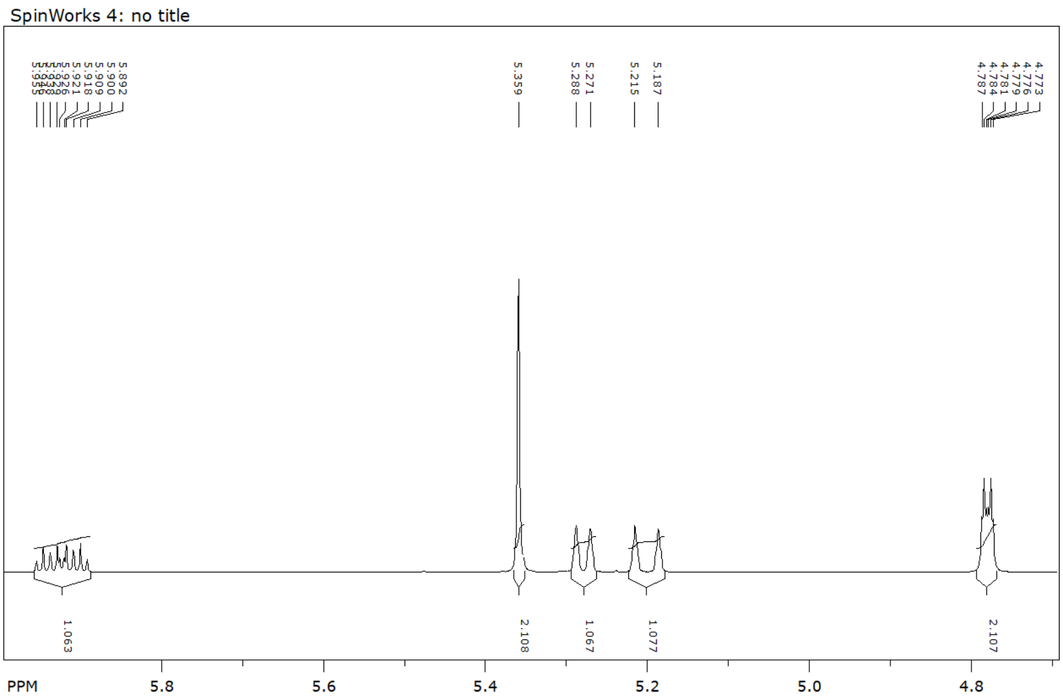

file: ... Łodzi\Desktop\nmr\mfl-2905\10\fid expt: <zg30>
transmitter freq.: 600.263707 MHz
time domain size: 65536 points
width: 12335.53 Hz = 20.5502 ppm = 0.188225 Hz/pt
number of scans: 16

freq. of 0 ppm: 600.260018 MHz
processed size: 32768 complex points
LB: 0.300 GF: 0.0000

Figure S6: ^{13}C NMR Spectrum for **18g** in $CDCl_3$ and expanded spectral regions

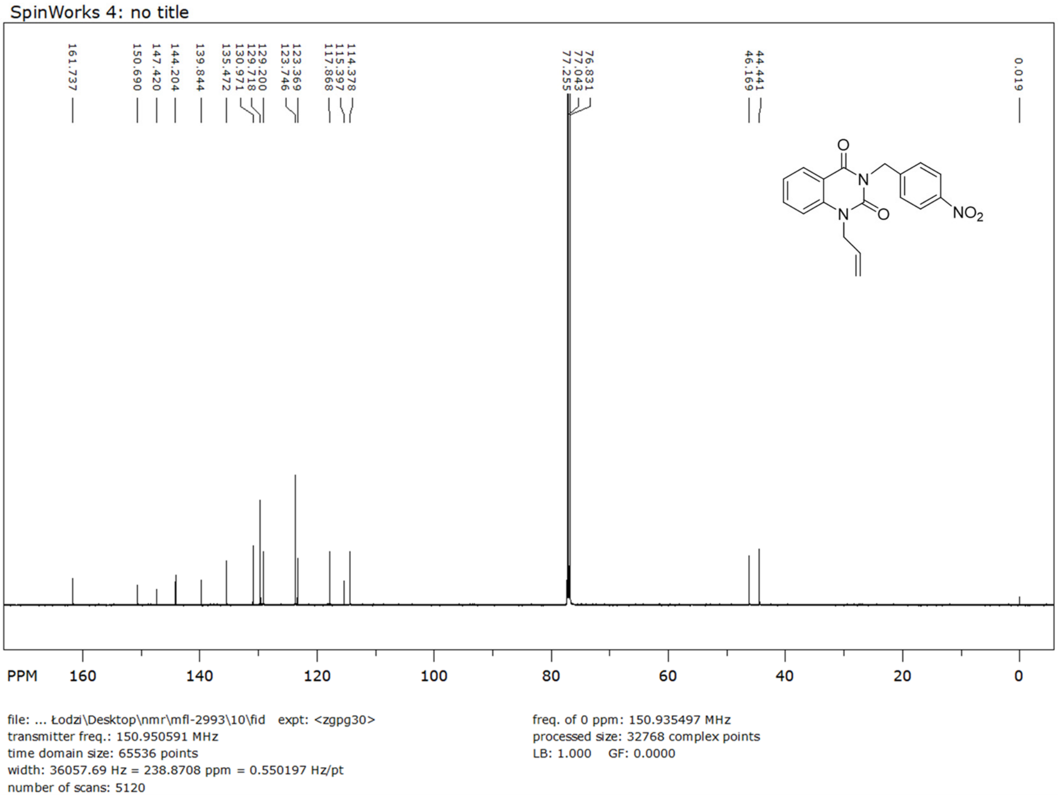

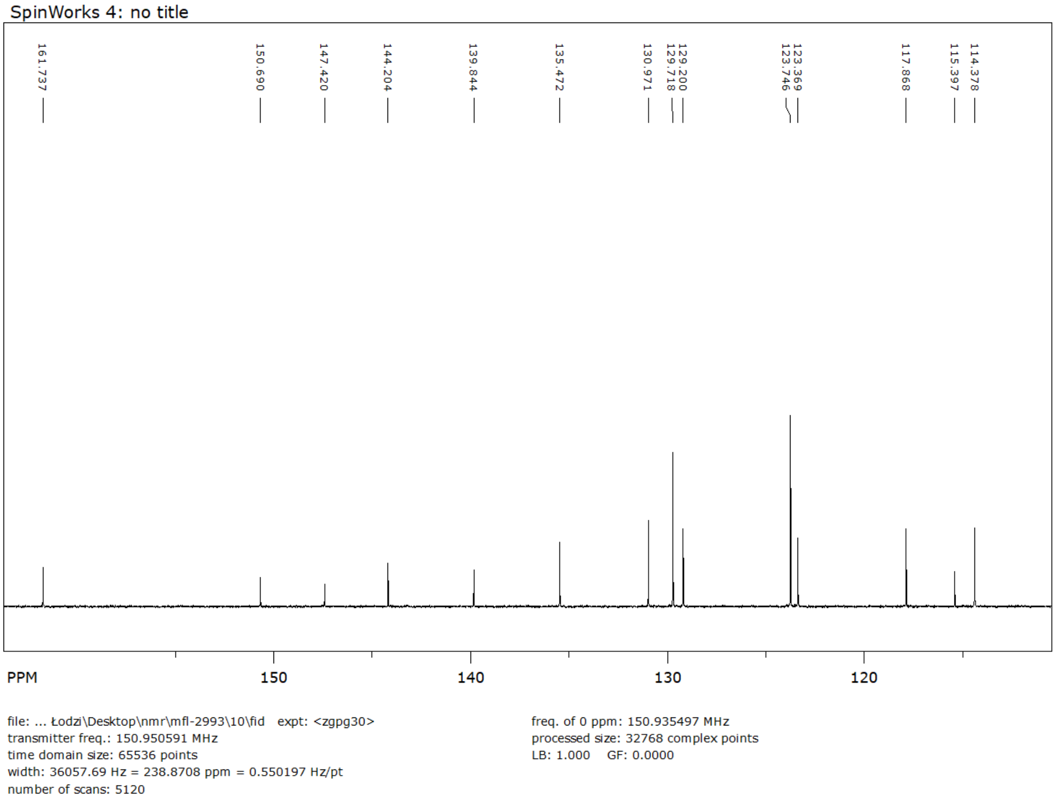

Figure S7: ^{31}P NMR Spectrum of the raw product of the synthesis of isoxazolidines *cis*-**16a**/*trans*-**16a** in $CDCl_3$

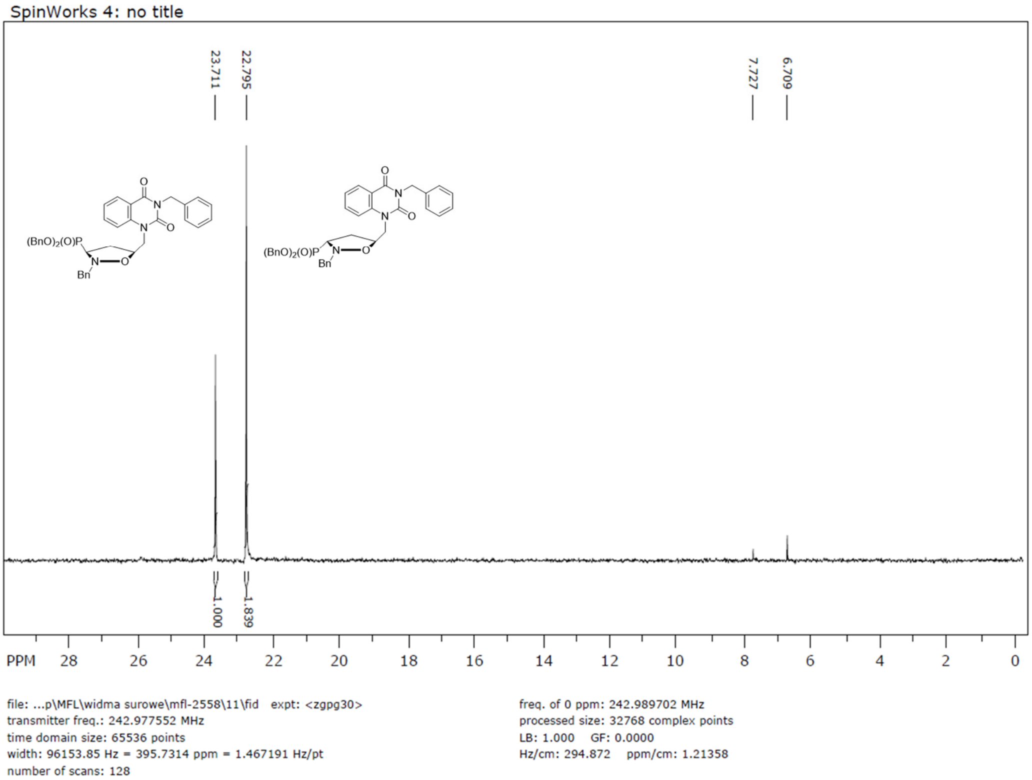

Figure S8: ^{31}P NMR Spectrum of the raw product of the synthesis of isoxazolidines *cis*-**16b**/*trans*-**16b** in $CDCl_3$

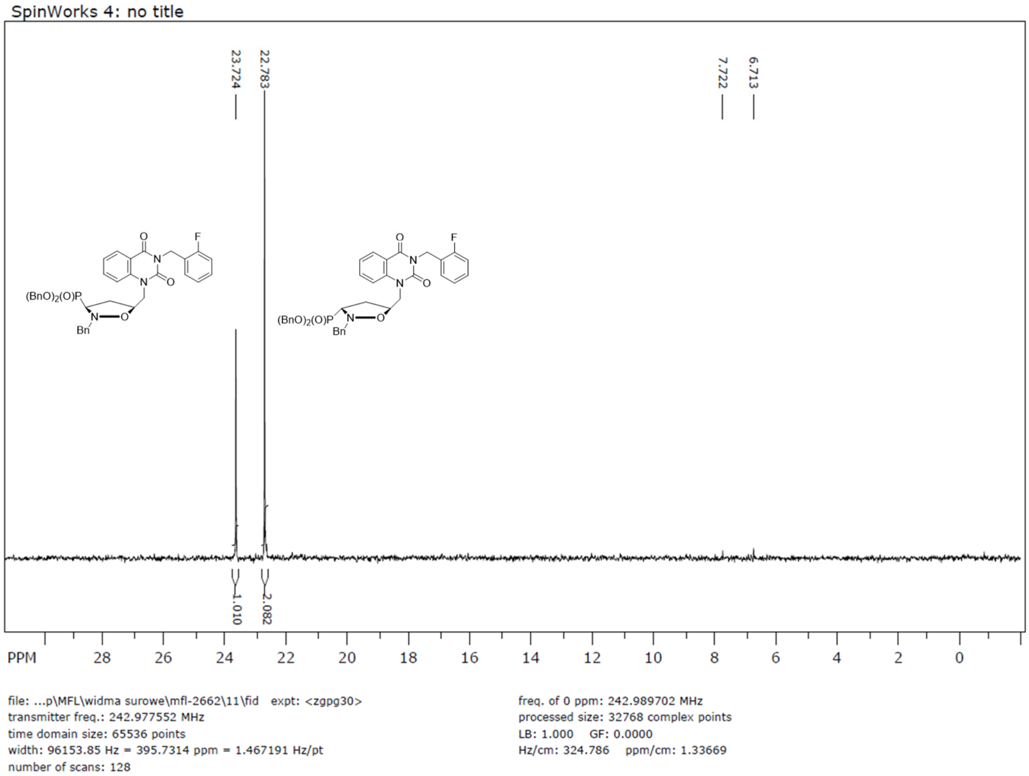

Figure S9: ^{31}P NMR Spectrum of the raw product of the synthesis of isoxazolidines *cis*-**16c**/*trans*-**16c** in $CDCl_3$

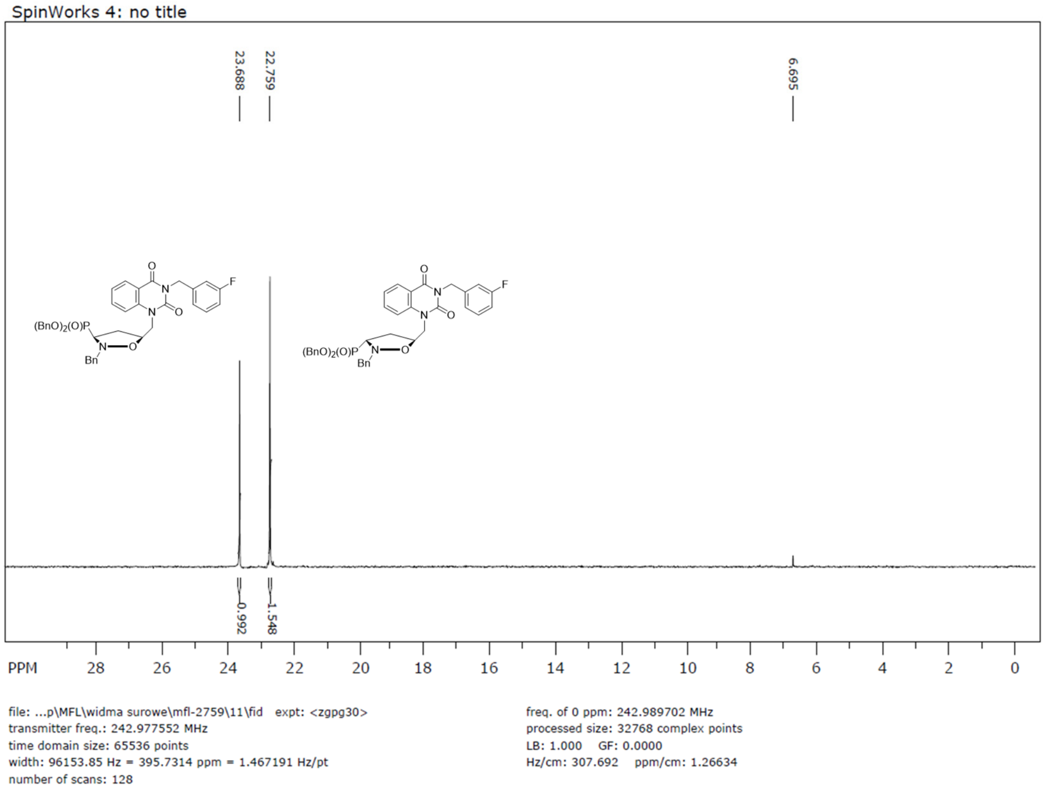

Figure S10: ^{31}P NMR Spectrum of the raw product of the synthesis of isoxazolidines *cis*-**16d**/*trans*-**16d** in $CDCl_3$

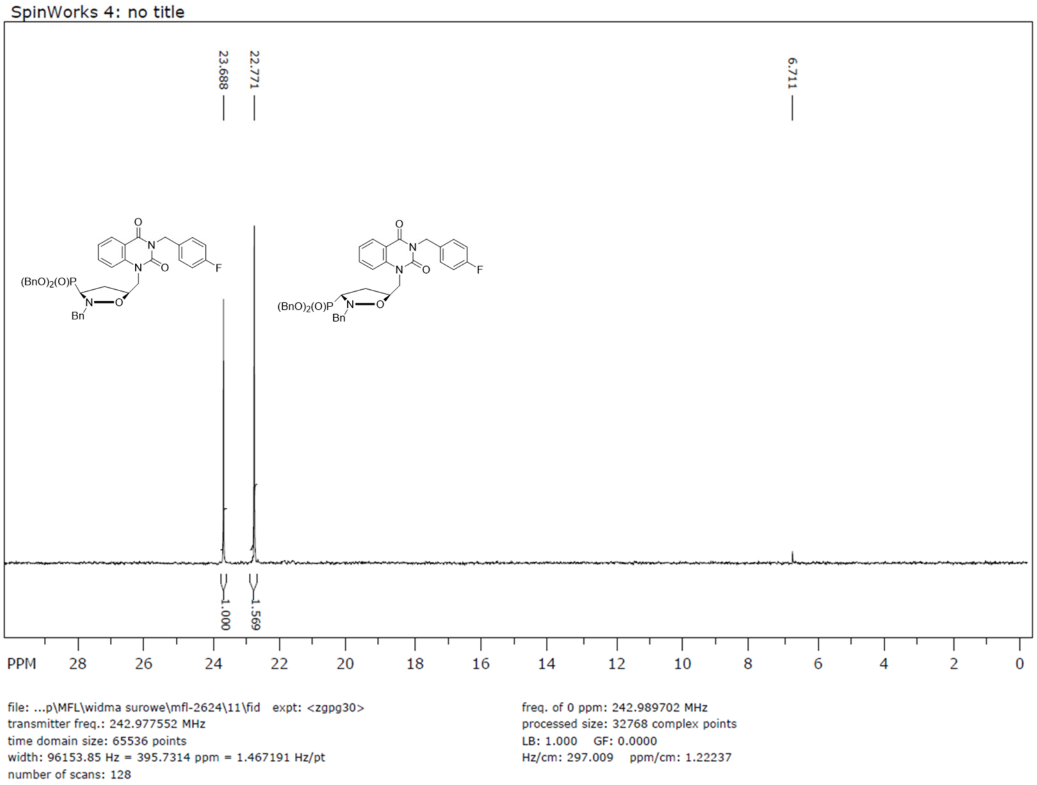

Figure S11: ^{31}P NMR Spectrum of the raw product of the synthesis of isoxazolidines *cis*-**16e**/*trans*-**16e** in $CDCl_3$

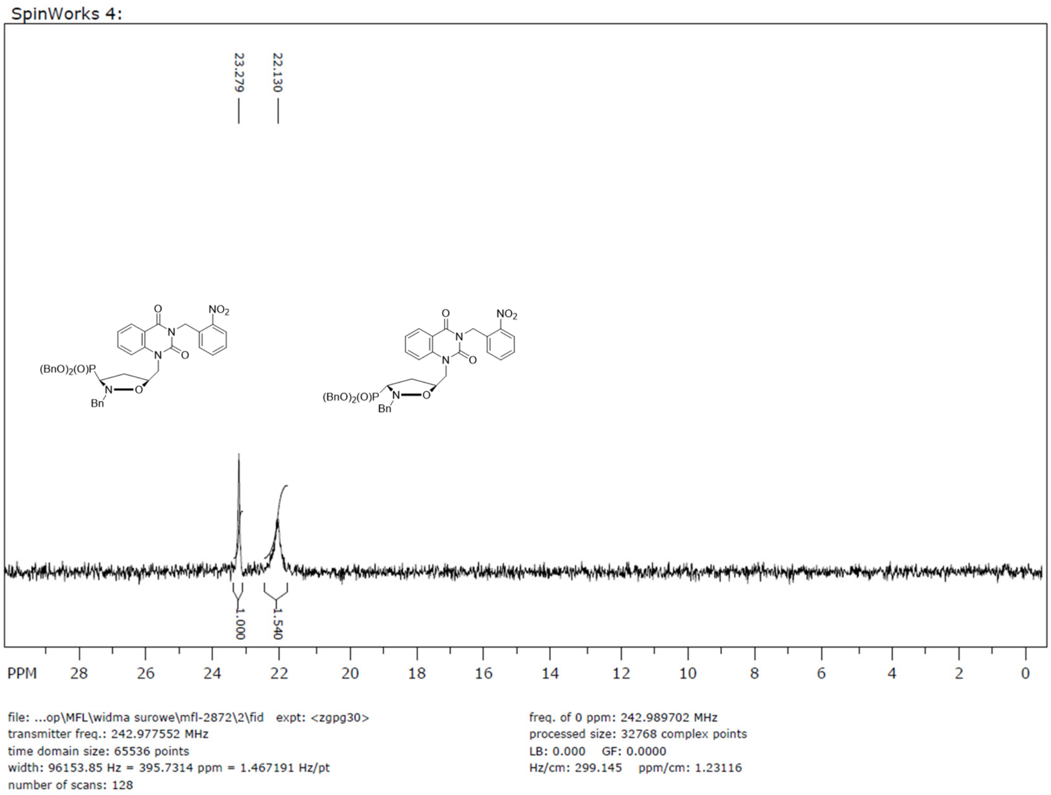

Figure S12: ^{31}P NMR Spectrum of the raw product of the synthesis of isoxazolidines *cis*-**16f**/*trans*-**16f** in $CDCl_3$

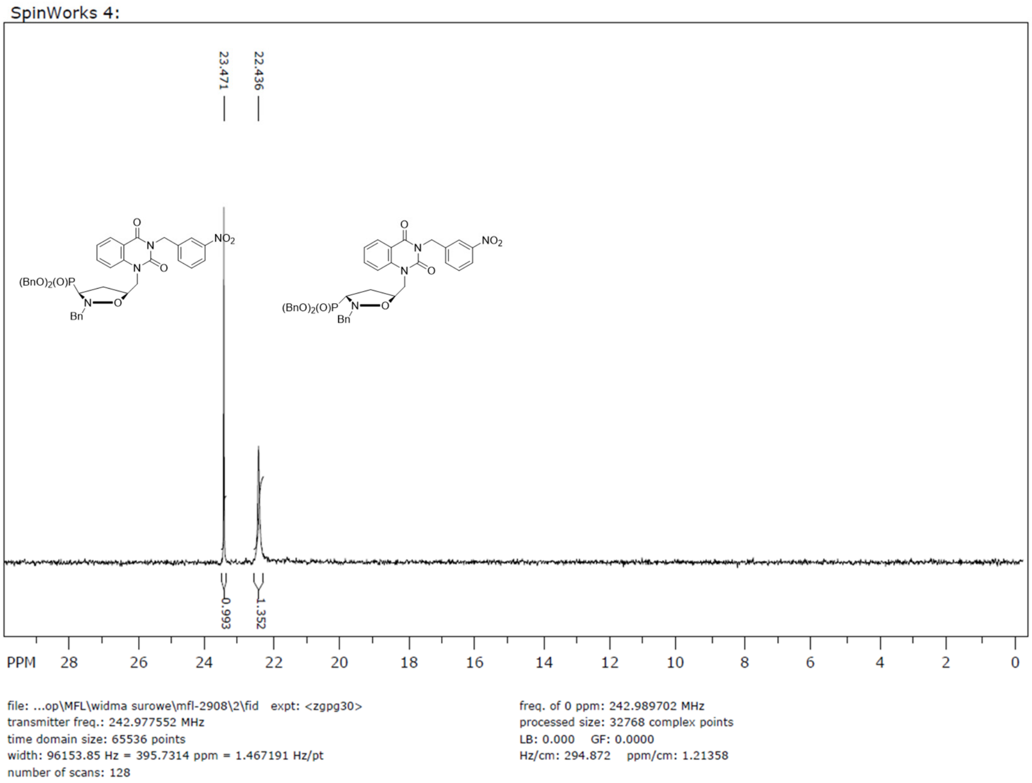

Figure S13: ^{31}P NMR Spectrum of the raw product of the synthesis of isoxazolidines *cis*-**16g**/*trans*-**16g** in $CDCl_3$

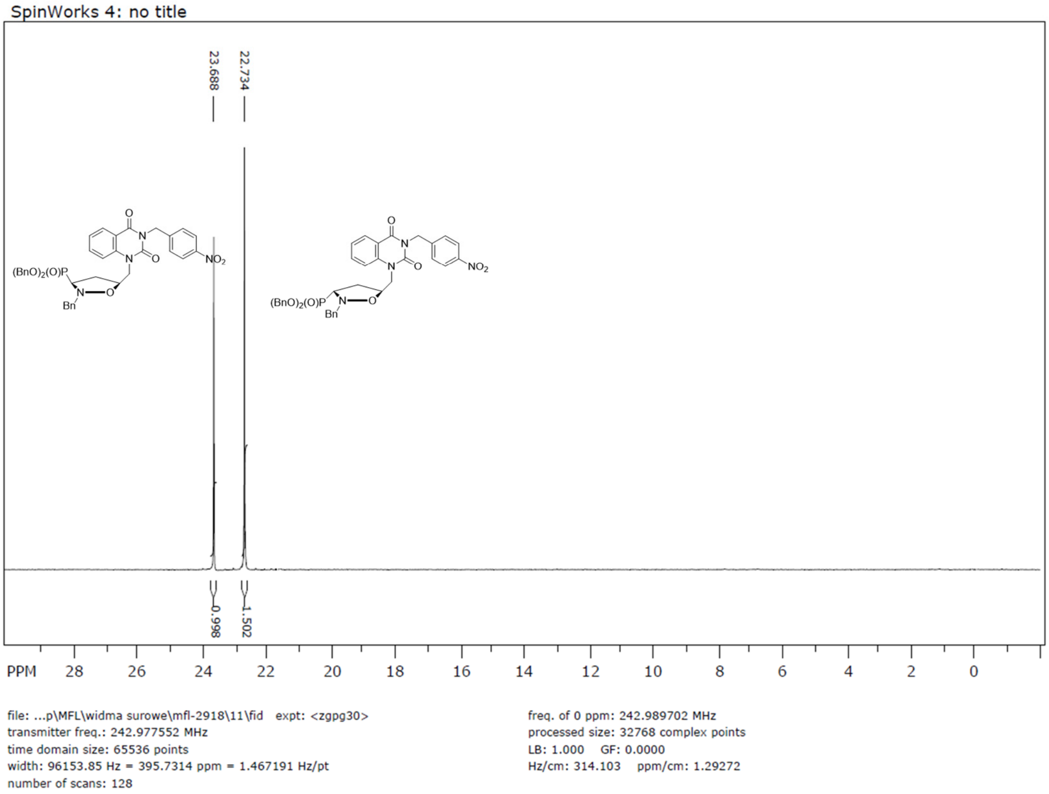

Figure S14: ^{1}H NMR Spectrum for mixture of *cis*-**16a**/*trans*-**16a** (97:3) in $CDCl_3$ and expanded spectral regions

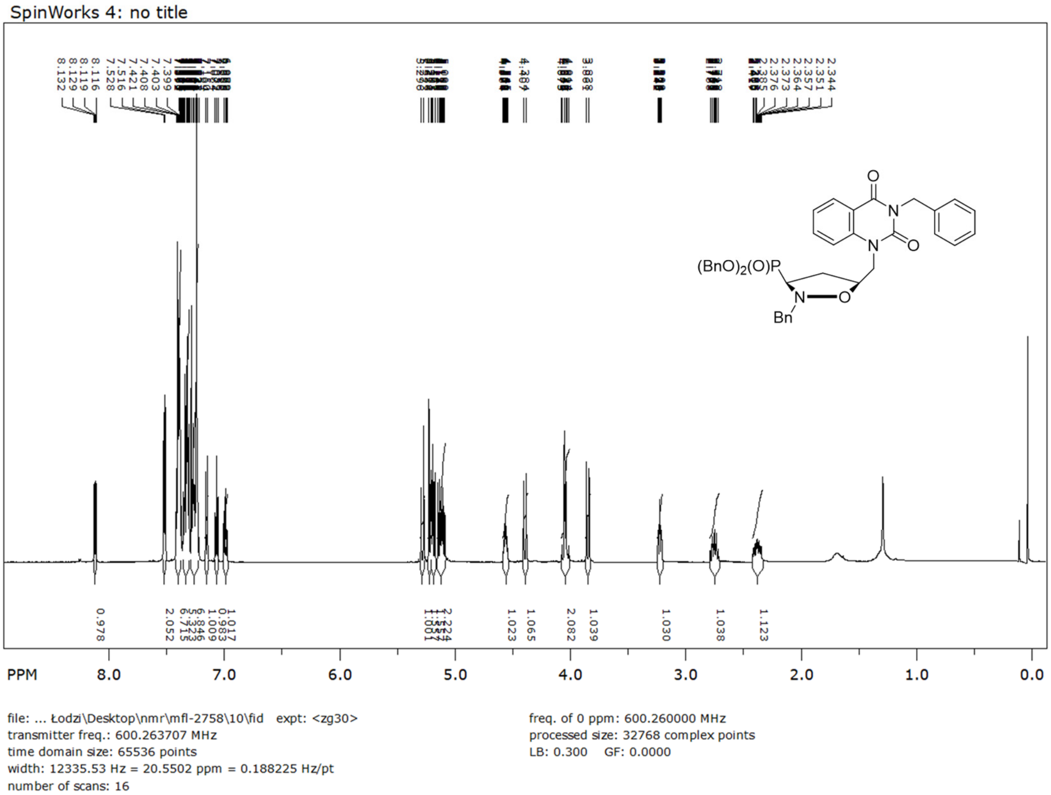

SpinWorks 4: no title

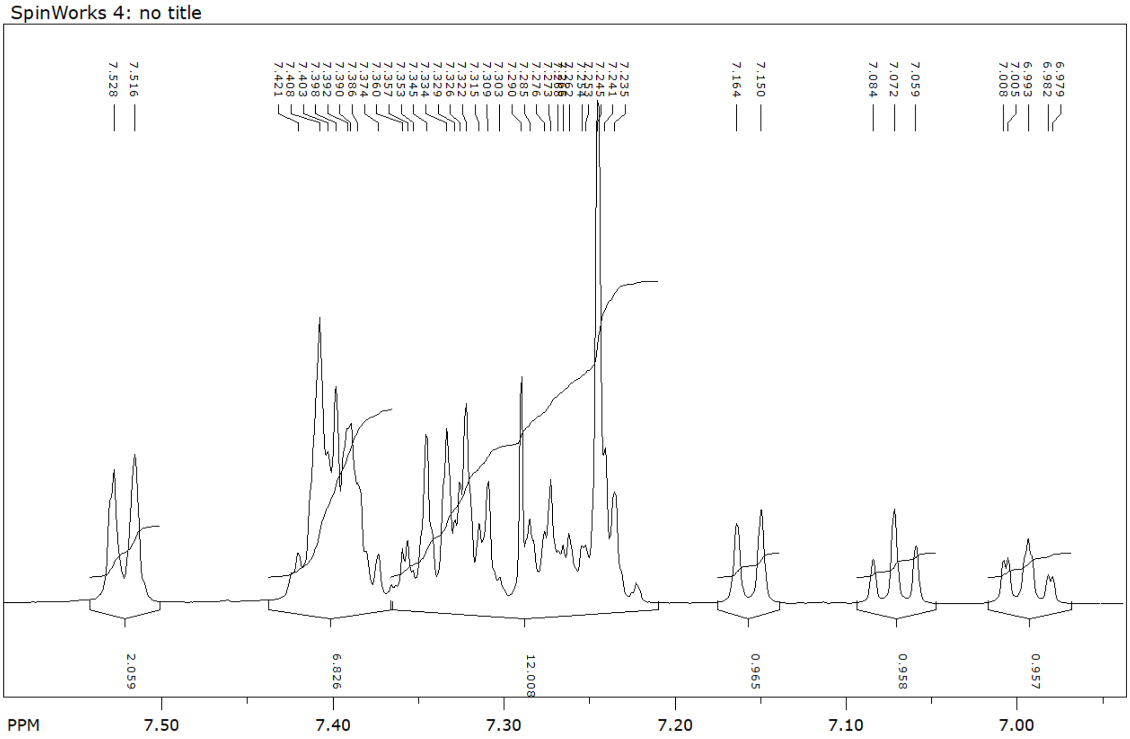

file: D:\mfl-2758\10\fid expt: <zg30>
transmitter freq.: 600.263707 MHz
time domain size: 65536 points
width: 12335.53 Hz = 20.5502 ppm = 0.188225 Hz/pt
number of scans: 16

freq. of 0 ppm: 600.260000 MHz
processed size: 32768 complex points
LB: 0.300 GF: 0.0000

SpinWorks 4: no title

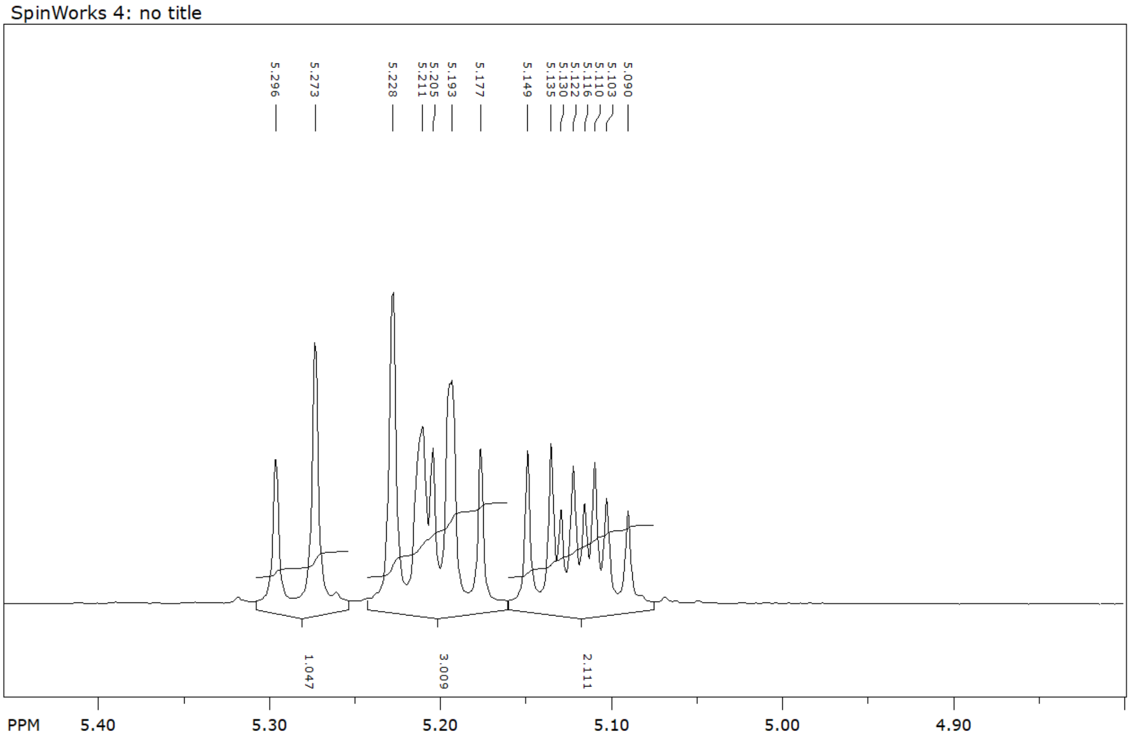

file: D:\mfl-2758\10\fid expt: <zg30>
transmitter freq.: 600.263707 MHz
time domain size: 65536 points
width: 12335.53 Hz = 20.5502 ppm = 0.188225 Hz/pt
number of scans: 16

freq. of 0 ppm: 600.260000 MHz
processed size: 32768 complex points
LB: 0.300 GF: 0.0000

SpinWorks 4: no title

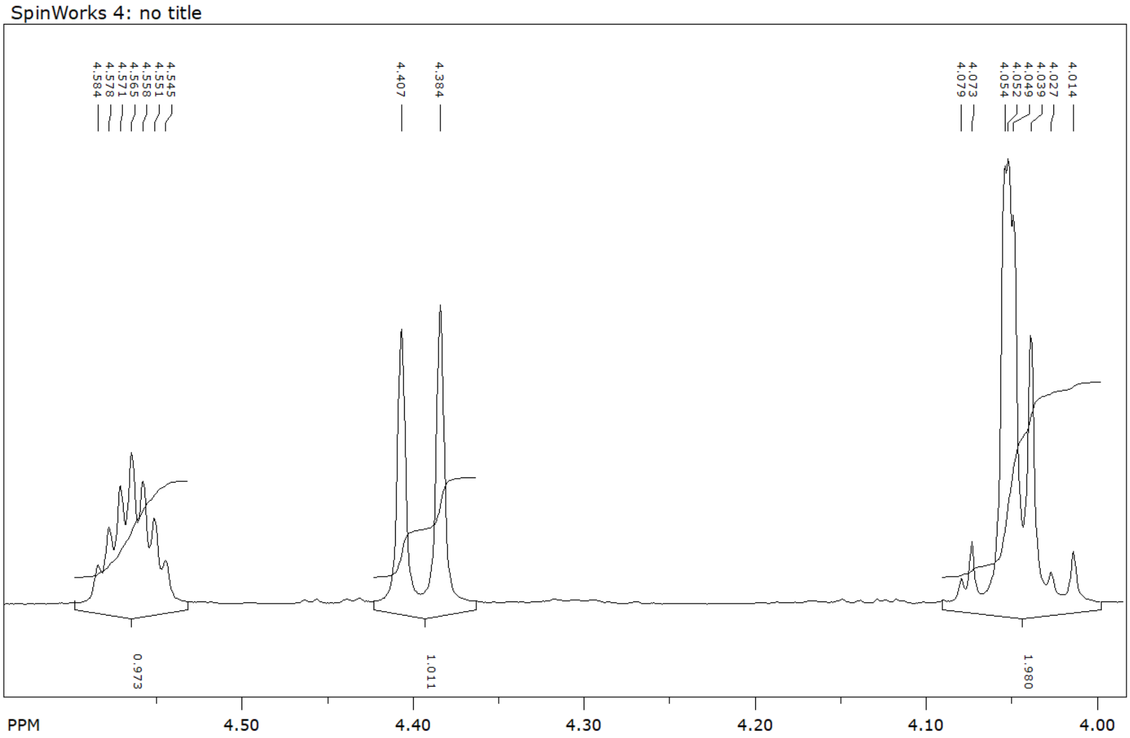

file: D:\mfl-2758\10\fid expt: <zg30>
transmitter freq.: 600.263707 MHz
time domain size: 65536 points
width: 12335.53 Hz = 20.5502 ppm = 0.188225 Hz/pt
number of scans: 16

freq. of 0 ppm: 600.260000 MHz
processed size: 32768 complex points
LB: 0.300 GF: 0.0000

SpinWorks 4: no title

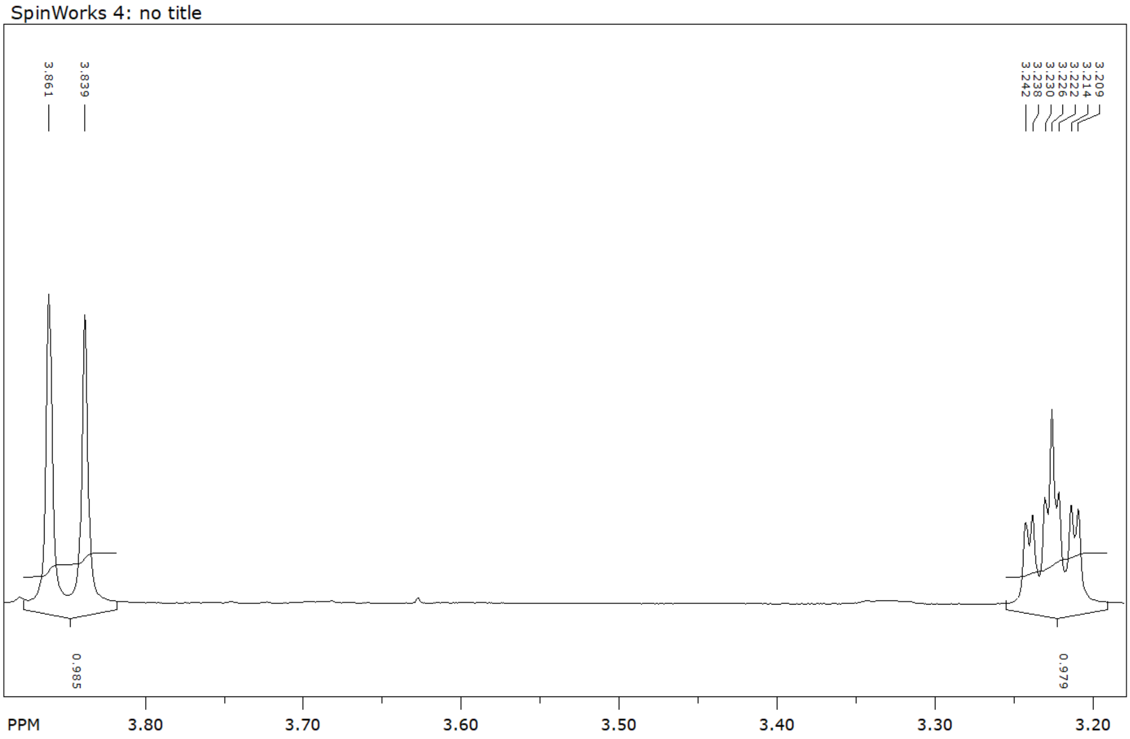

file: D:\mfl-2758\10\fid expt: <zg30>
transmitter freq.: 600.263707 MHz
time domain size: 65536 points
width: 12335.53 Hz = 20.5502 ppm = 0.188225 Hz/pt
number of scans: 16

freq. of 0 ppm: 600.260000 MHz
processed size: 32768 complex points
LB: 0.300 GF: 0.0000

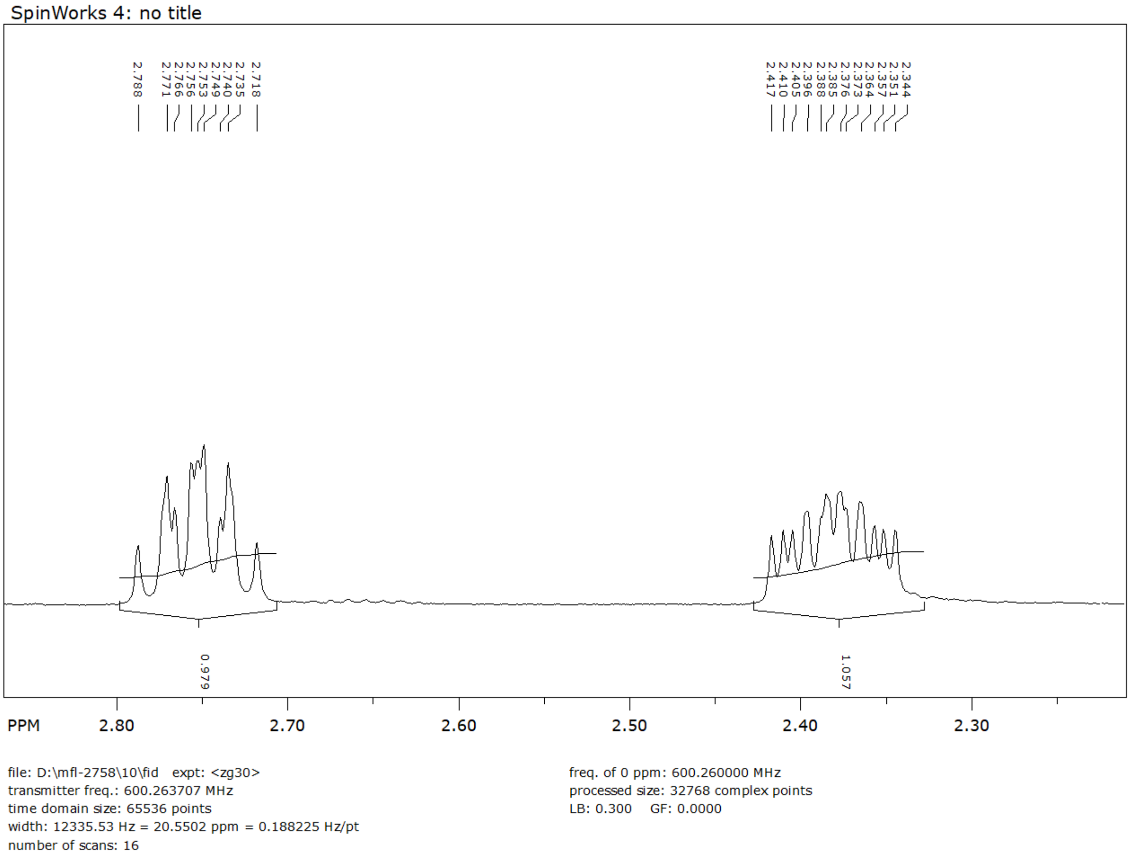

Figure S15: ^{31}P NMR Spectrum for mixture of *cis*-**16a**/*trans*-**16a** (97:3) in $CDCl_3$

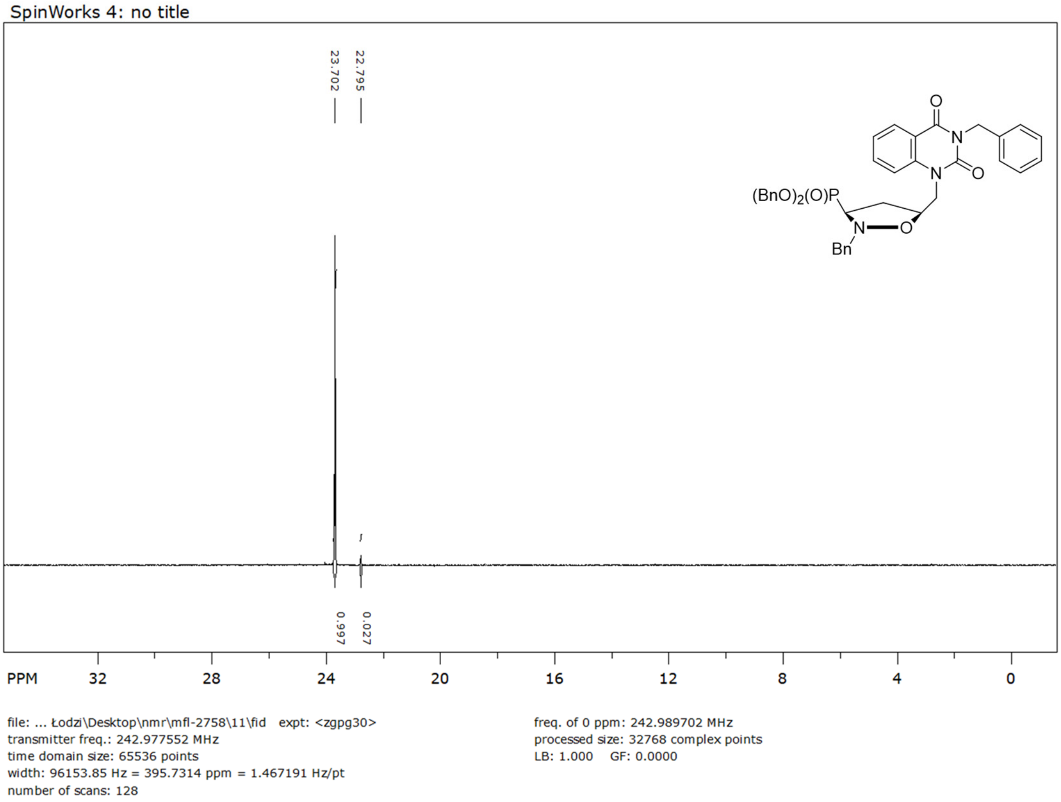

Figure S16: ^{13}C NMR Spectrum for mixture of *cis*-**16a**/*trans*-**16a** (97:3) in $CDCl_3$ and expanded spectral regions

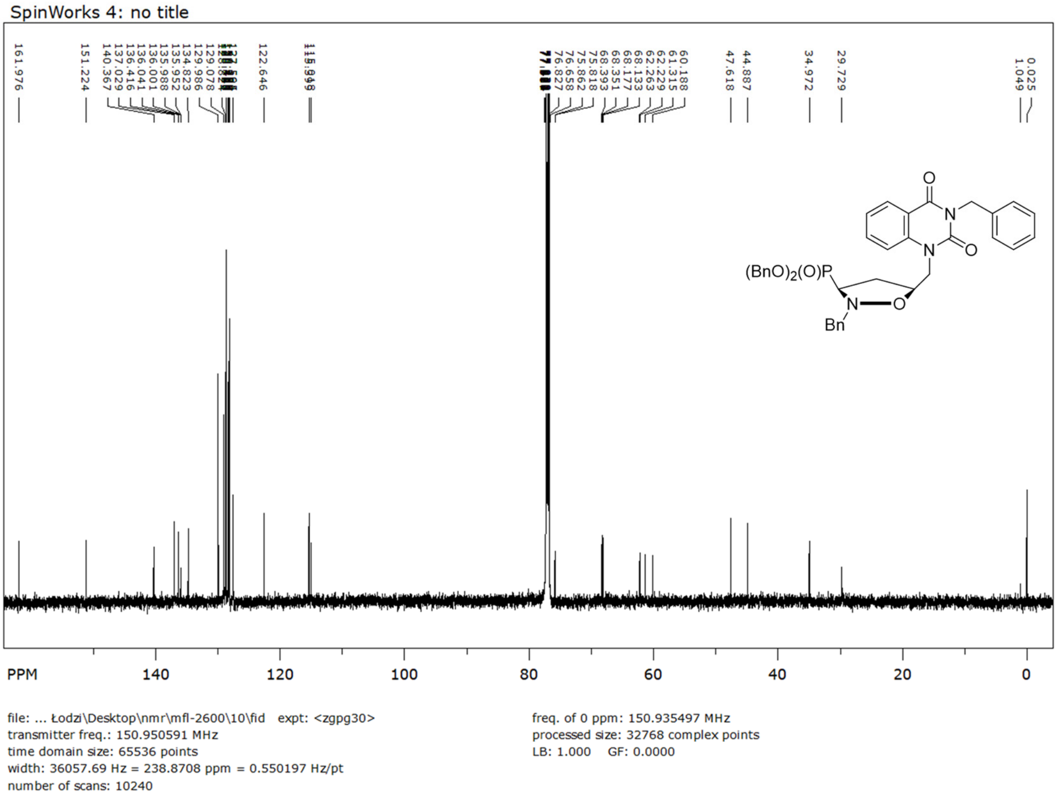

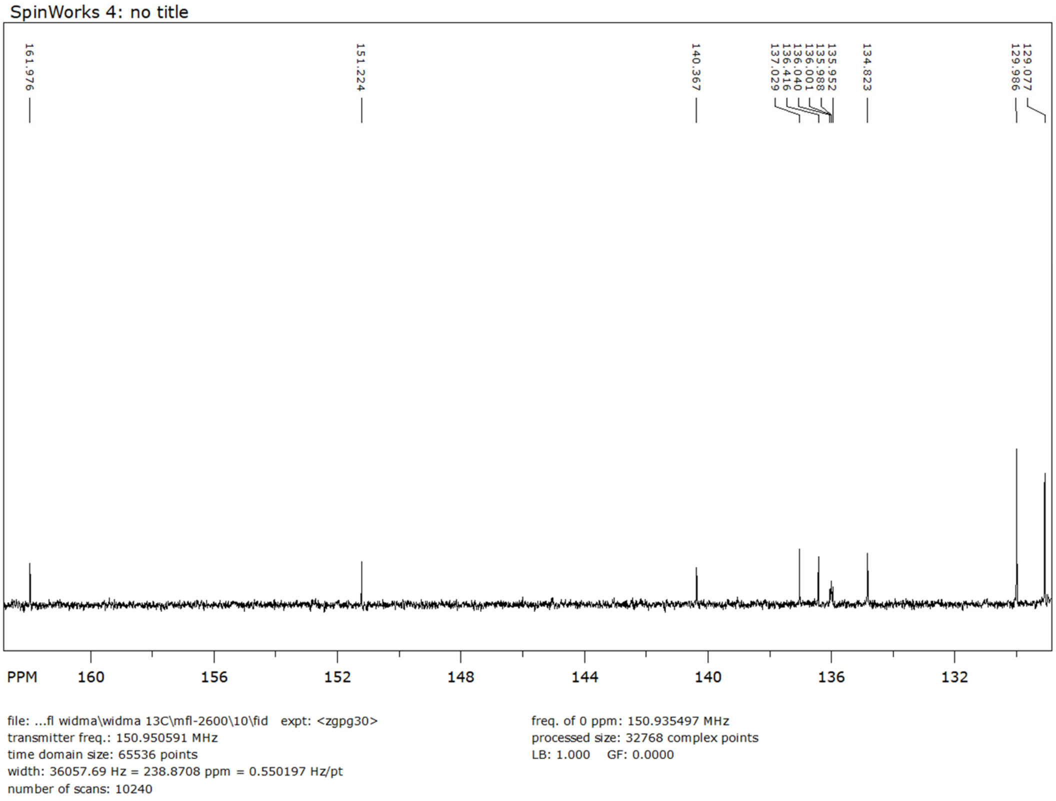

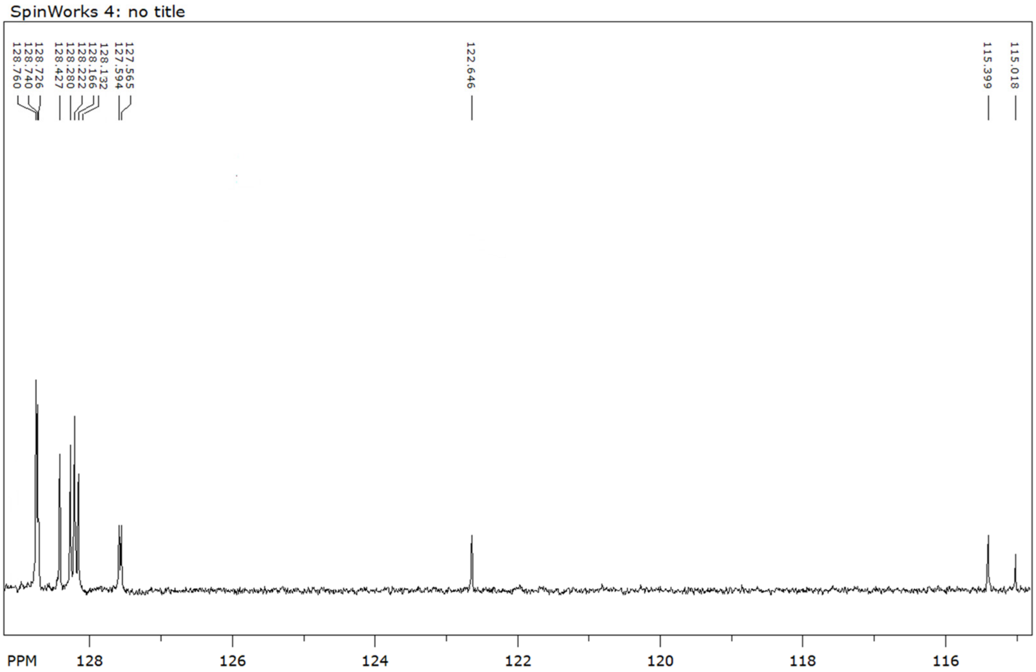

file: ...fl widma\widma 13C\mfl-2600\10\fid expt: <zgpg30>
transmitter freq.: 150.950591 MHz
time domain size: 65536 points
width: 36057.69 Hz = 238.8708 ppm = 0.550197 Hz/pt
number of scans: 10240

freq. of 0 ppm: 150.935497 MHz
processed size: 32768 complex points
LB: 1.000 GF: 0.0000

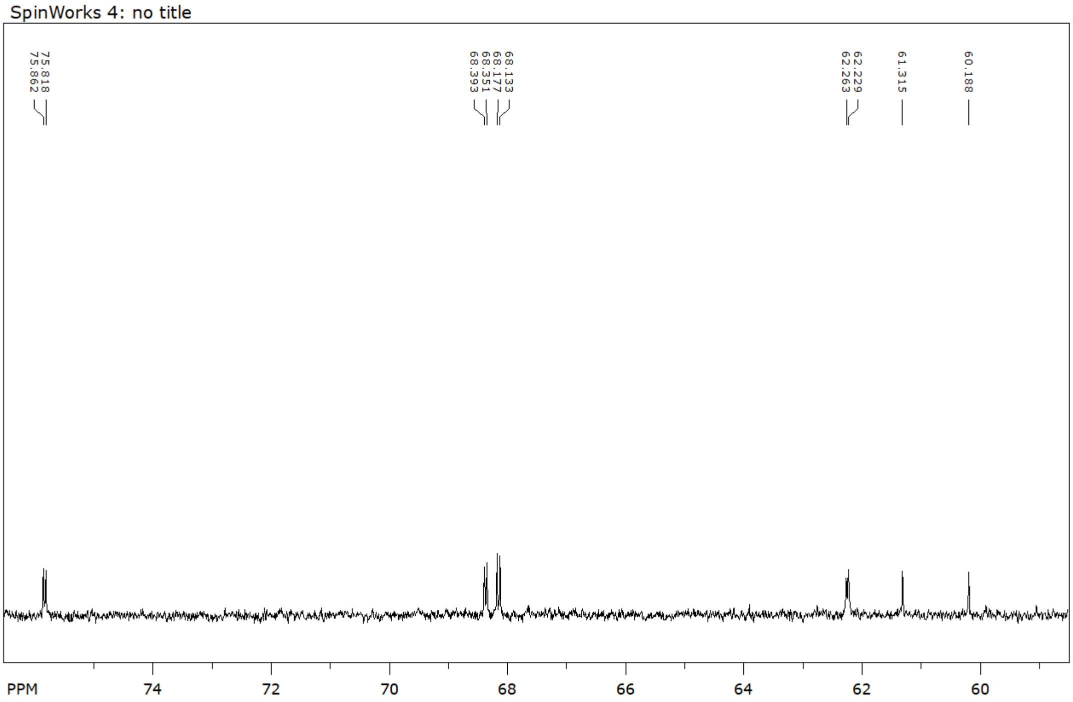

file: ... Łodzi\Desktop\nmr\mfl-2600\10\fid expt: <zgpg30>
transmitter freq.: 150.950591 MHz
time domain size: 65536 points
width: 36057.69 Hz = 238.8708 ppm = 0.550197 Hz/pt
number of scans: 10240

freq. of 0 ppm: 150.935497 MHz
processed size: 32768 complex points
LB: 1.000 GF: 0.0000

Figure S17: HPLC chromatogram for mixture of *cis*-**16a**/*trans*-**16a** (97:3)

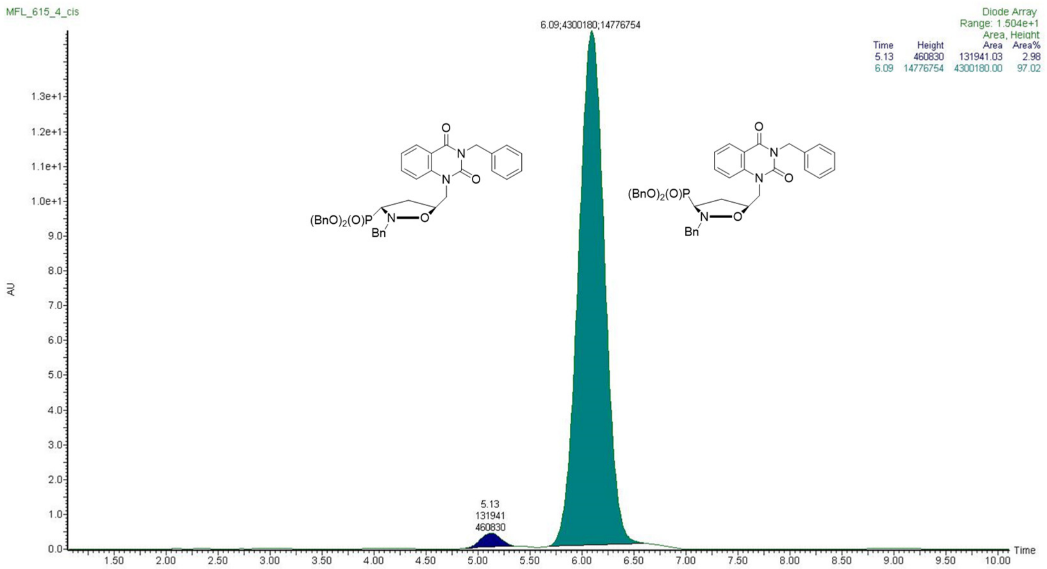

Figure S18: ^{1}H NMR Spectrum for *trans*-**16a** in CDCl$_3$ and expanded spectral regions

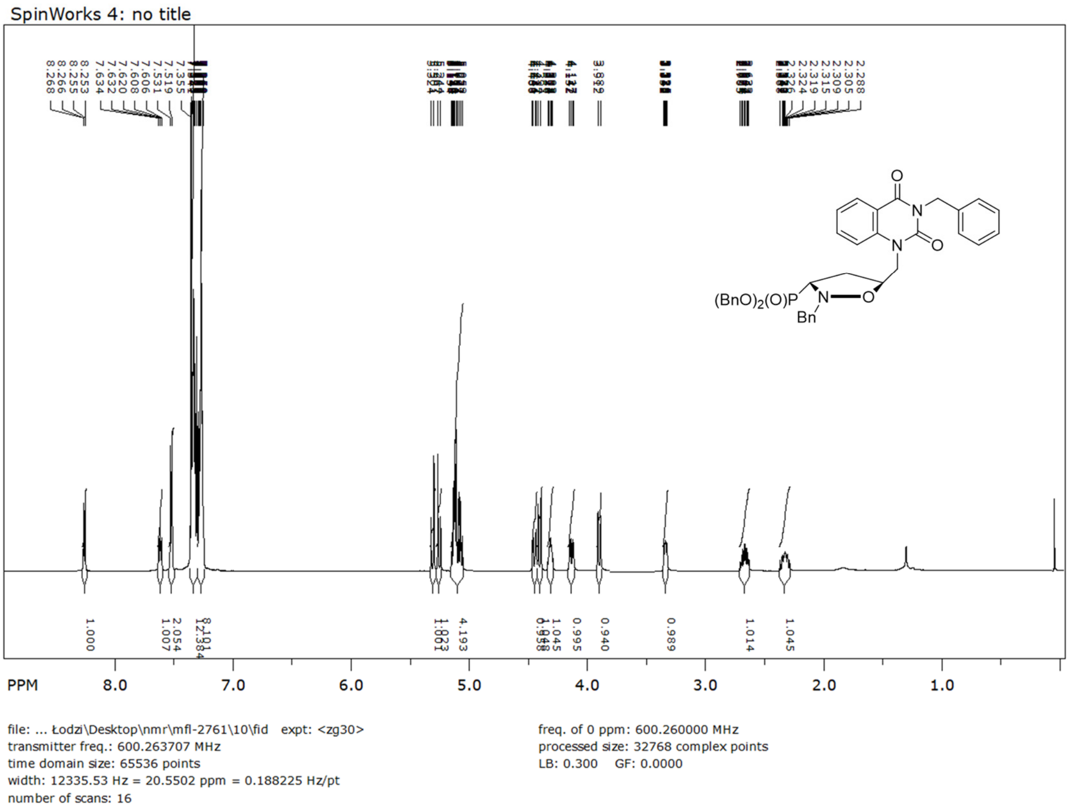

SpinWorks 4: no title

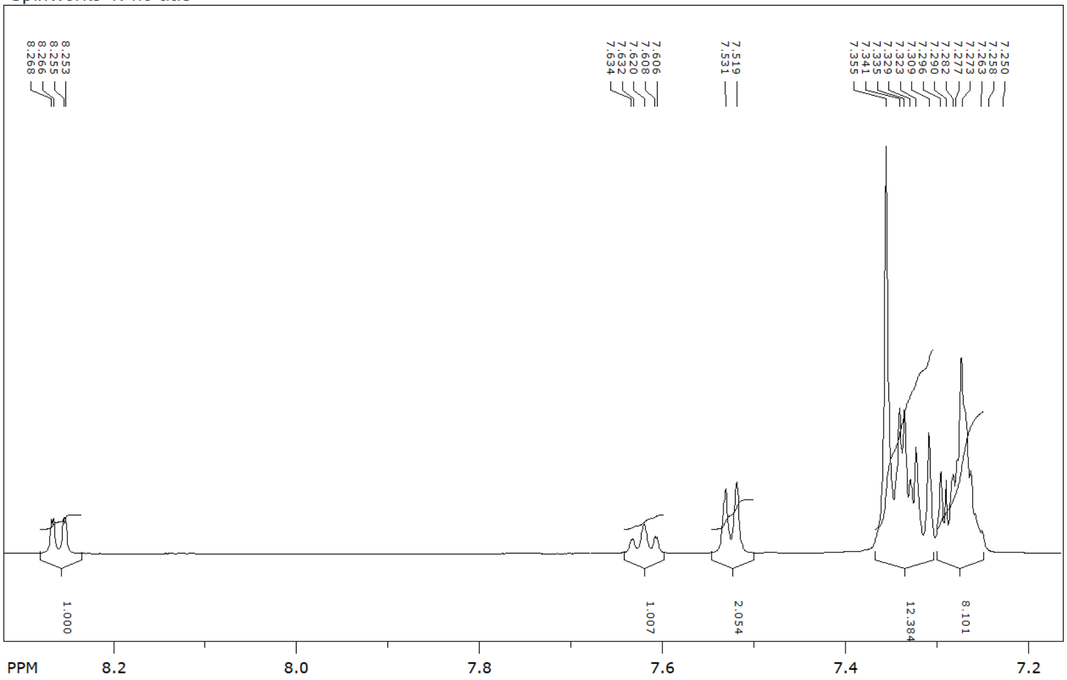

file: ... Łodzi\Desktop\nmr\mfl-2761\10\fid expt: <zg30>
transmitter freq.: 600.263707 MHz
time domain size: 65536 points
width: 12335.53 Hz = 20.5502 ppm = 0.188225 Hz/pt
number of scans: 16

freq. of 0 ppm: 600.260000 MHz
processed size: 32768 complex points
LB: 0.300 GF: 0.0000

SpinWorks 4: no title

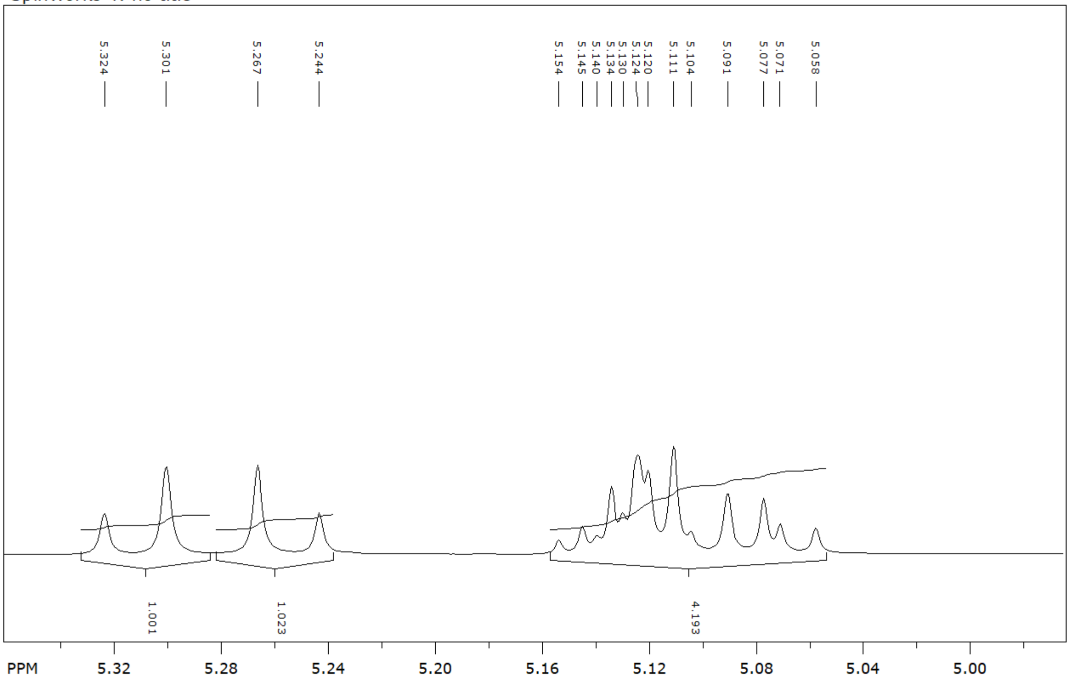

file: ... Łodzi\Desktop\nmr\mfl-2761\10\fid expt: <zg30>
transmitter freq.: 600.263707 MHz
time domain size: 65536 points
width: 12335.53 Hz = 20.5502 ppm = 0.188225 Hz/pt
number of scans: 16

freq. of 0 ppm: 600.260000 MHz
processed size: 32768 complex points
LB: 0.300 GF: 0.0000

SpinWorks 4: no title

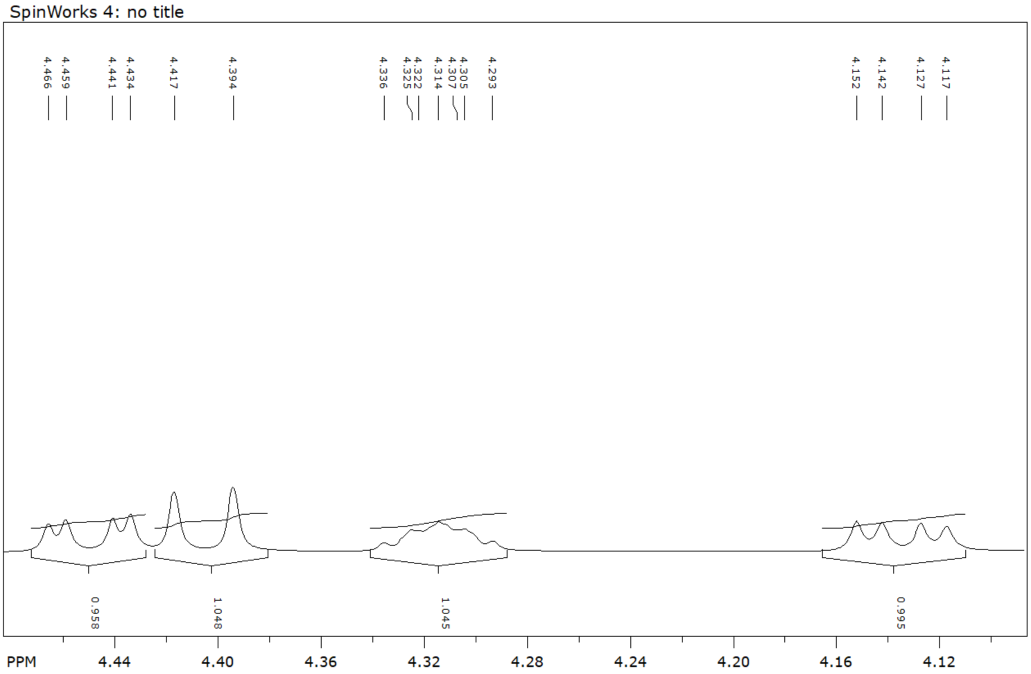

file: ... Łodzi\Desktop\nmr\mfl-2761\10\fid expt: <zg30>
transmitter freq.: 600.263707 MHz
time domain size: 65536 points
width: 12335.53 Hz = 20.5502 ppm = 0.188225 Hz/pt
number of scans: 16

freq. of 0 ppm: 600.260000 MHz
processed size: 32768 complex points
LB: 0.300 GF: 0.0000

SpinWorks 4: no title

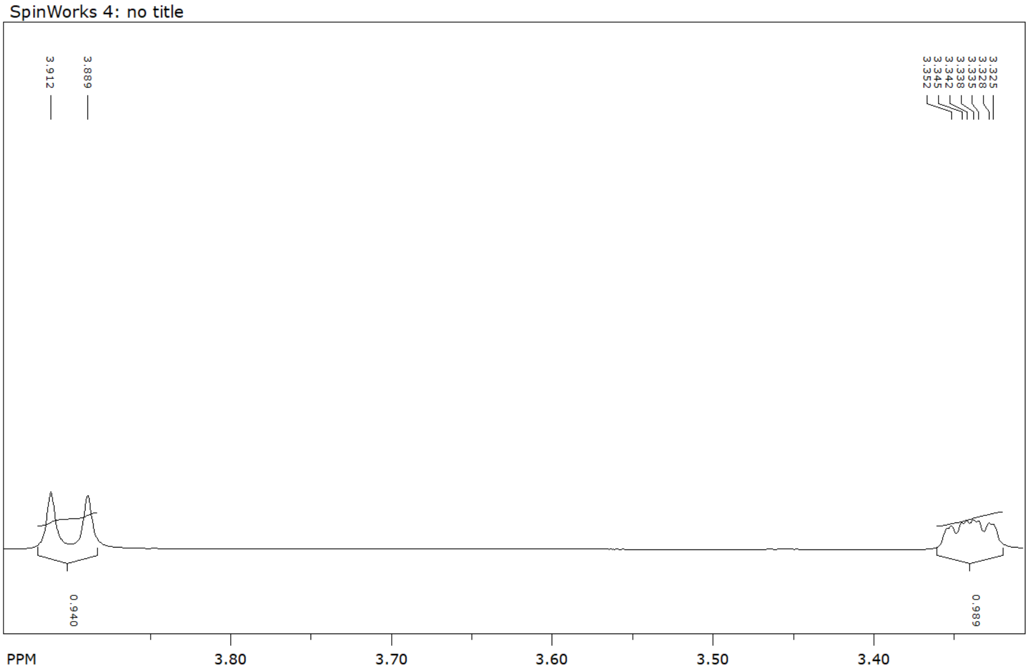

file: ... Łodzi\Desktop\nmr\mfl-2761\10\fid expt: <zg30>
transmitter freq.: 600.263707 MHz
time domain size: 65536 points
width: 12335.53 Hz = 20.5502 ppm = 0.188225 Hz/pt
number of scans: 16

freq. of 0 ppm: 600.260000 MHz
processed size: 32768 complex points
LB: 0.300 GF: 0.0000

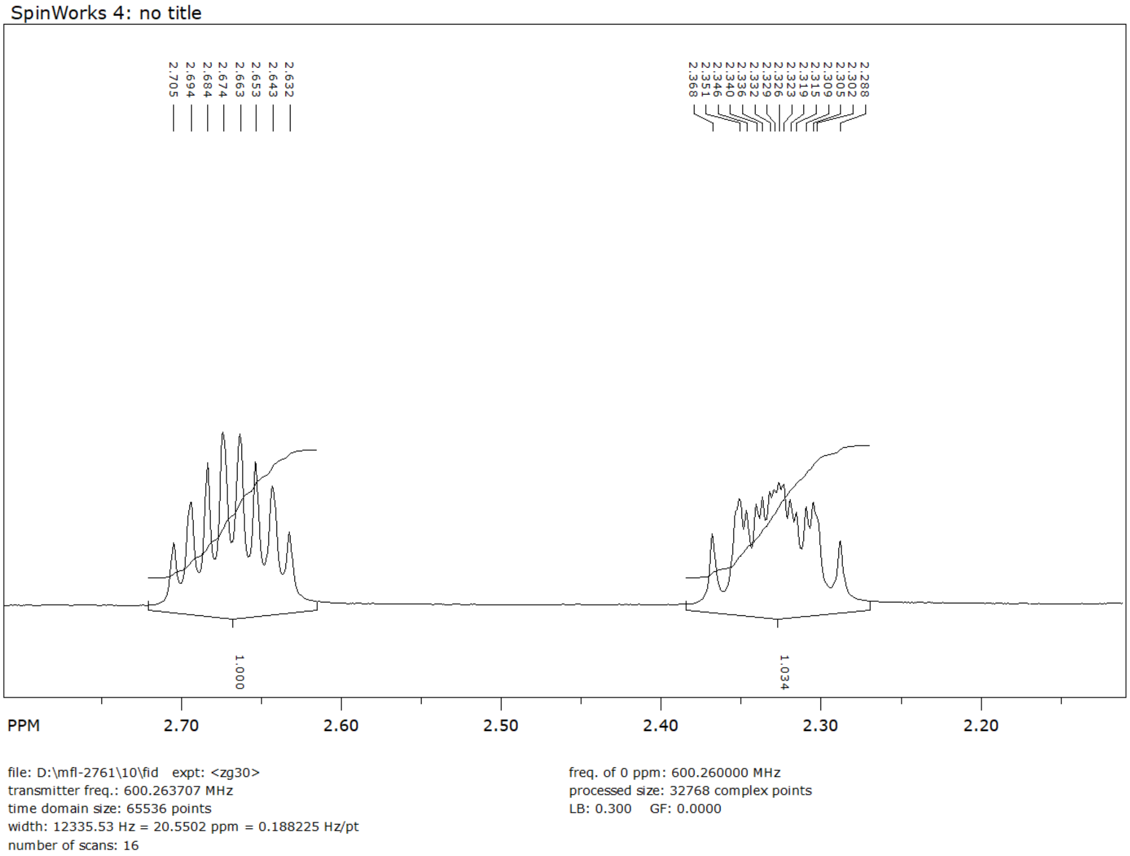

Figure S19: ^{31}P NMR Spectrum for *trans*-**16a** in $CDCl_3$

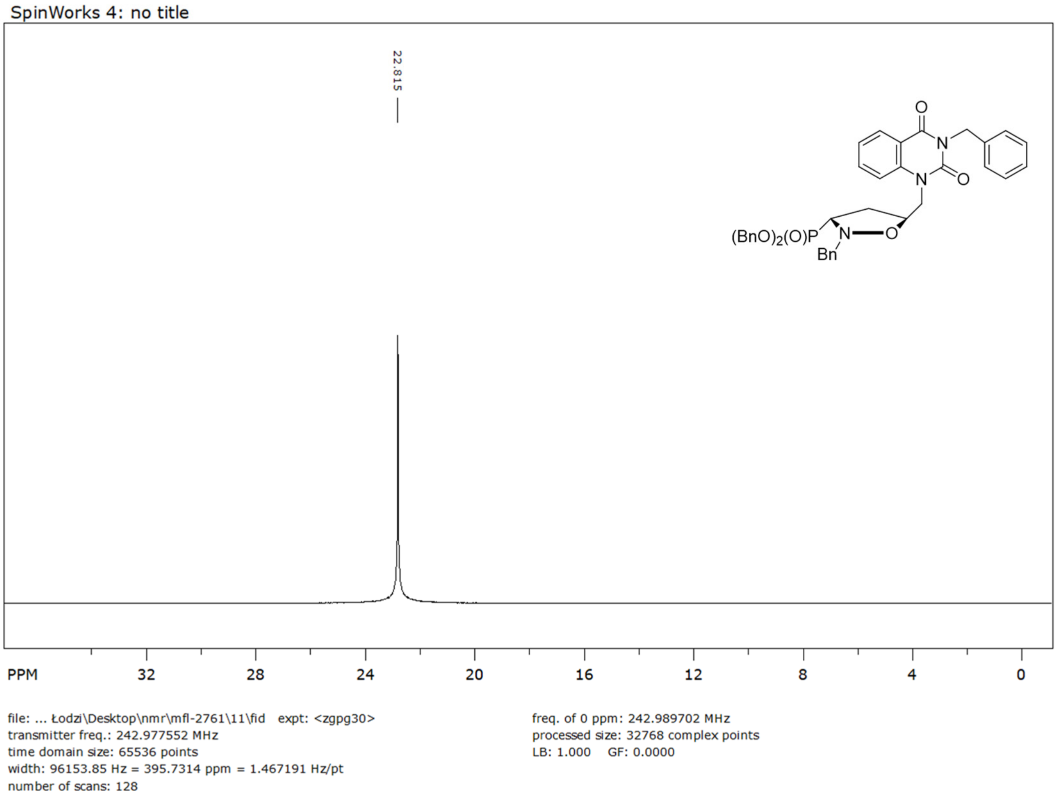

Figure S20: ^{13}C NMR Spectrum for *trans*-**16a** in $CDCl_3$ and expanded spectral regions

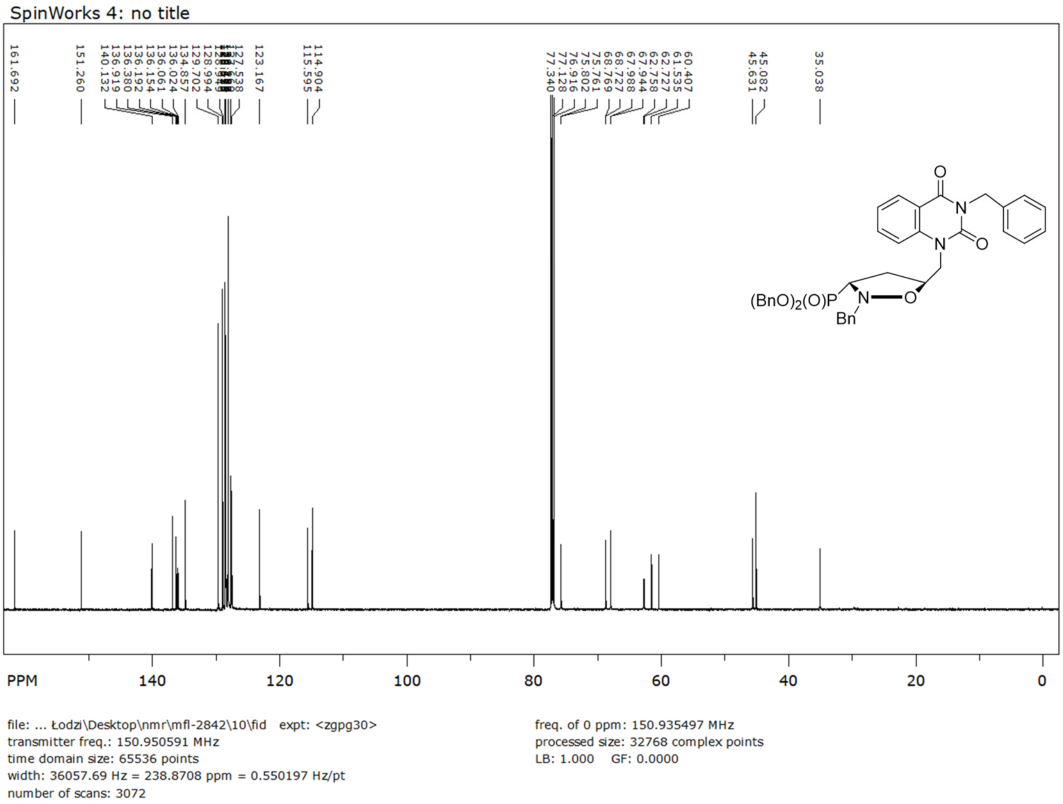

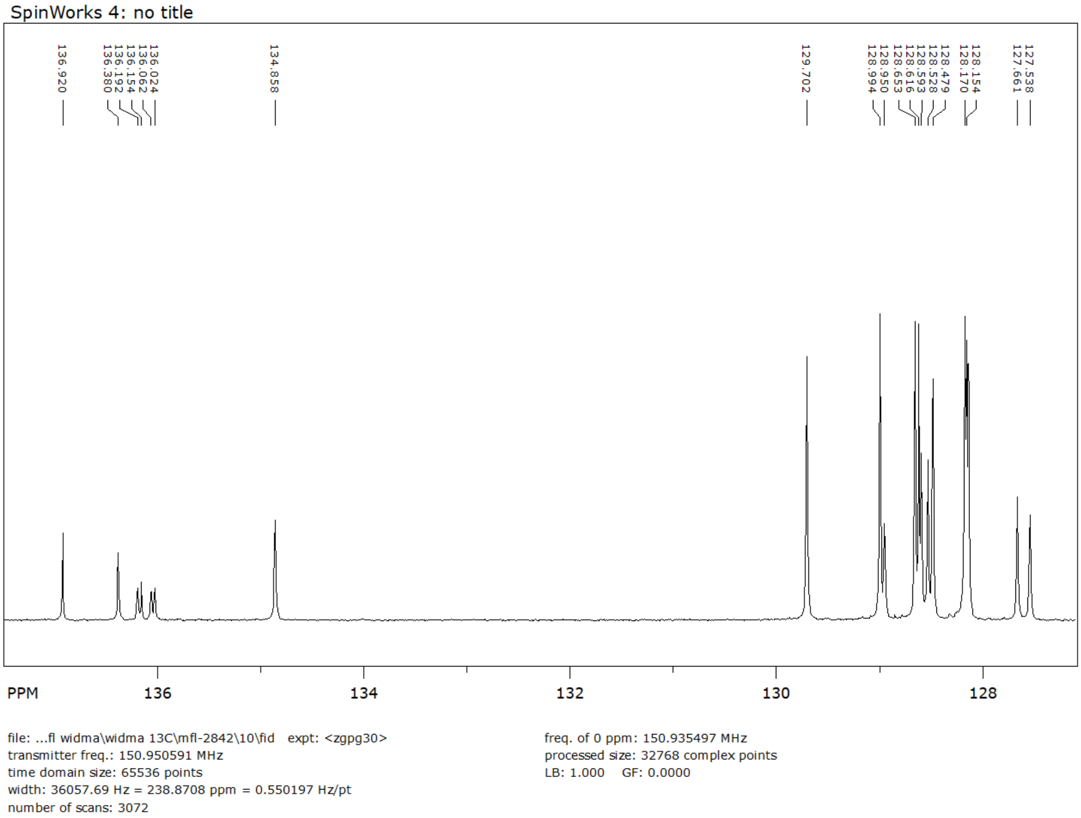

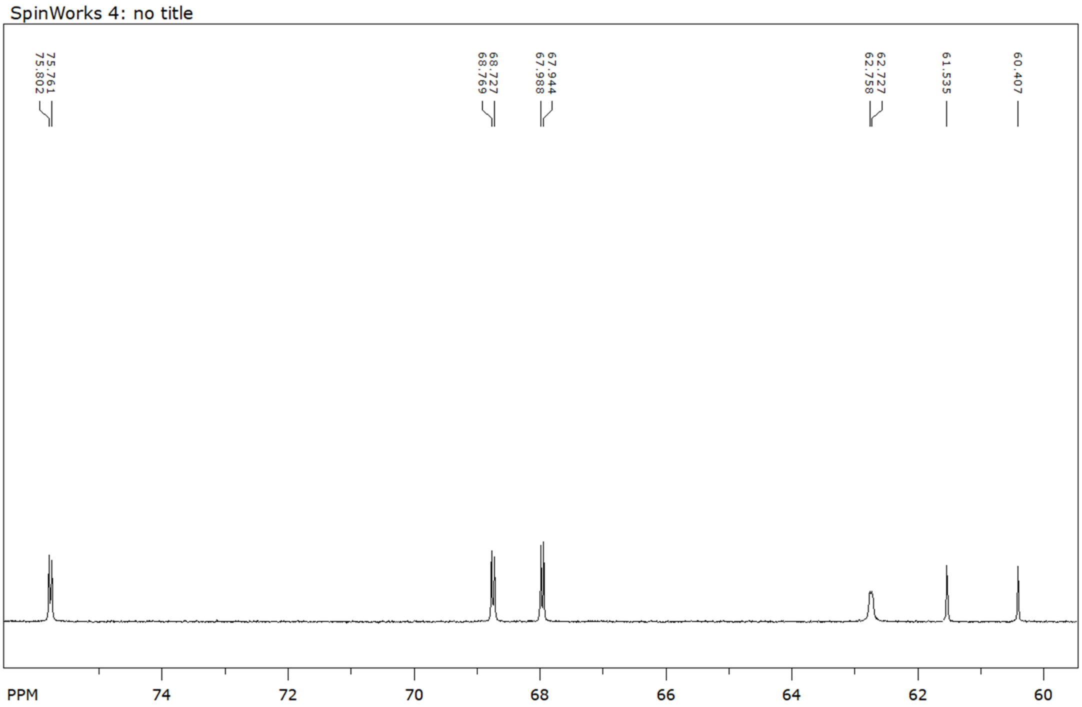

file: ...fl widma\widma 13C\mfl-2842\10\fid expt: <zgpg30>
transmitter freq.: 150.950591 MHz
time domain size: 65536 points
width: 36057.69 Hz = 238.8708 ppm = 0.550197 Hz/pt
number of scans: 3072

freq. of 0 ppm: 150.935497 MHz
processed size: 32768 complex points
LB: 1.000 GF: 0.0000

Figure S21: HPLC chromatogram for *trans*-**16a**

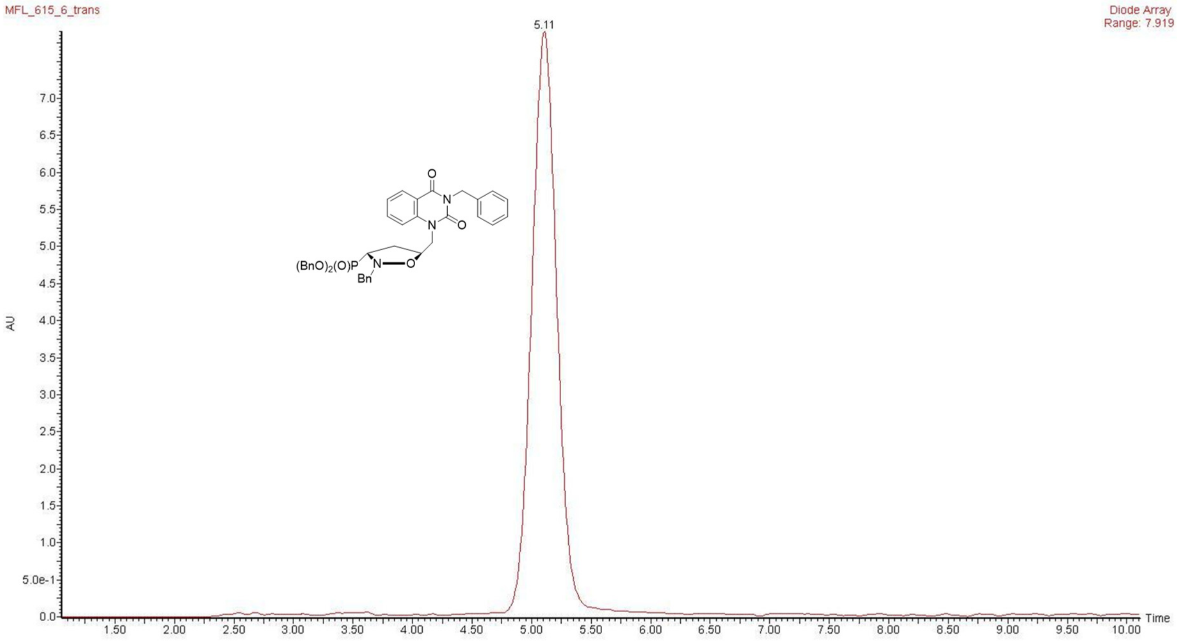

Figure S22: ^{1}H NMR Spectrum for mixture of *cis*-**16b**/*trans*-**16b** (90:10) in $CDCl_3$ and expanded spectral regions

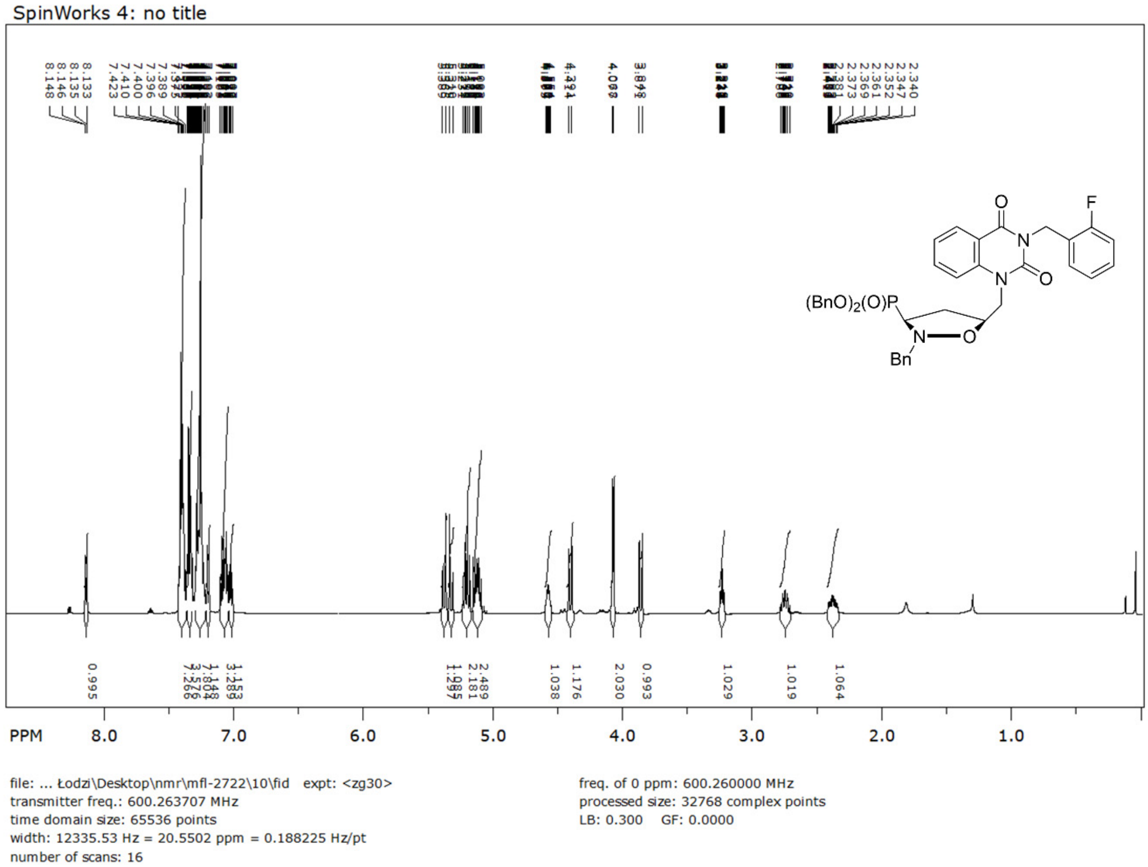

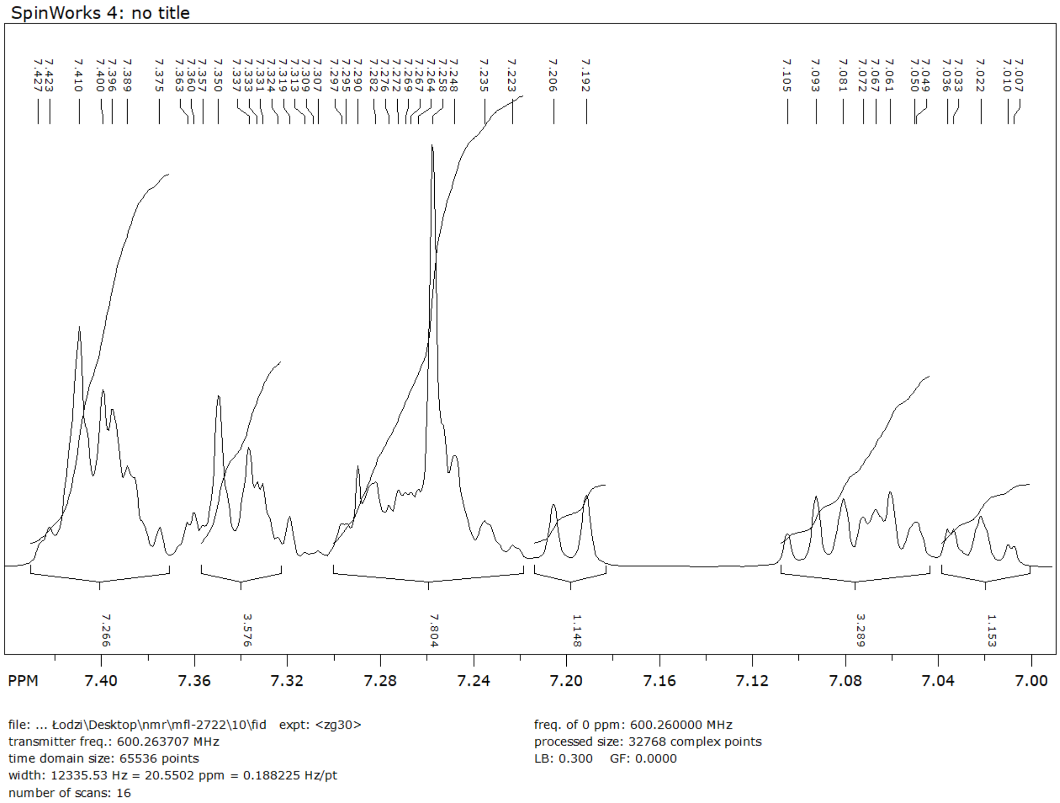

SpinWorks 4: no title

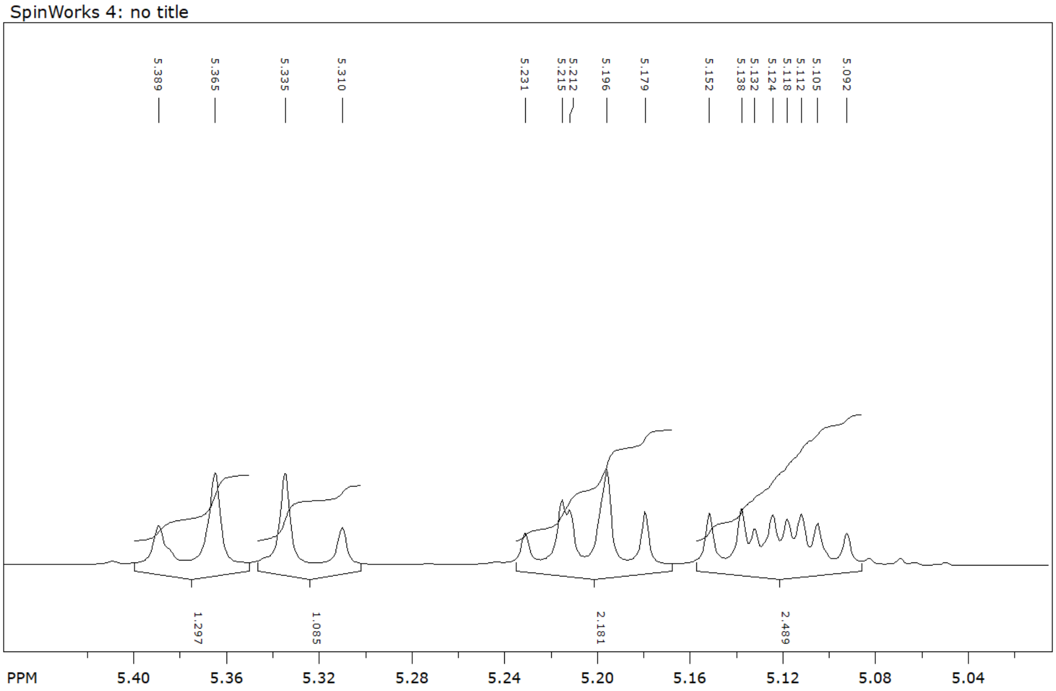

file: ... Łodzi\Desktop\nmr\mfl-2722\10\fid expt: <zg30>
transmitter freq.: 600.263707 MHz
time domain size: 65536 points
width: 12335.53 Hz = 20.5502 ppm = 0.188225 Hz/pt
number of scans: 16

freq. of 0 ppm: 600.260000 MHz
processed size: 32768 complex points
LB: 0.300 GF: 0.0000

SpinWorks 4: no title

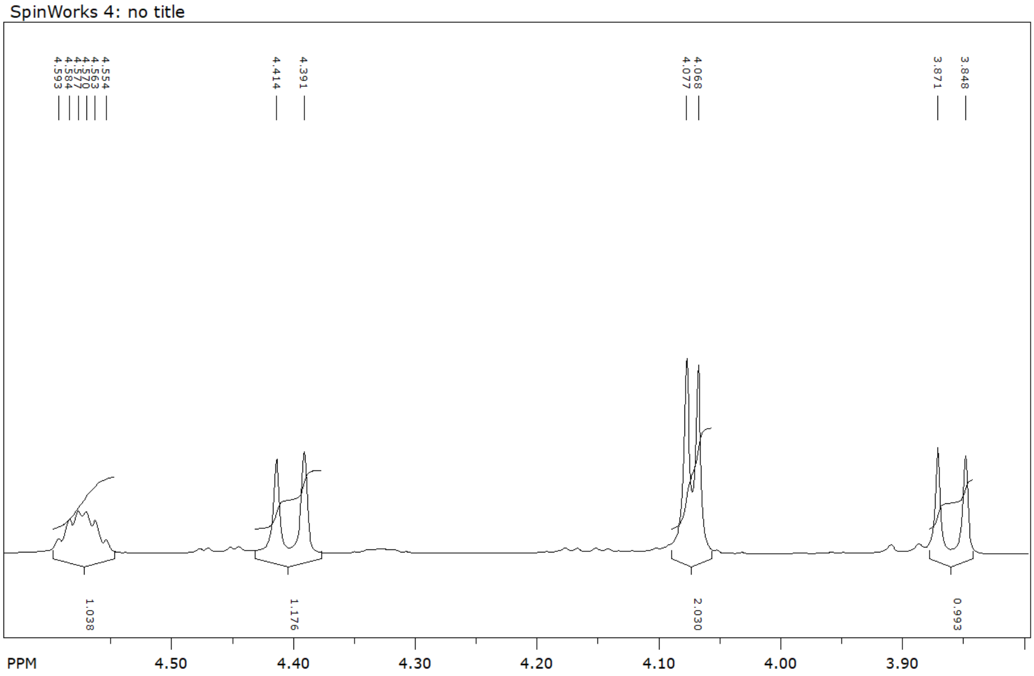

file: ... Łodzi\Desktop\nmr\mfl-2722\10\fid expt: <zg30>
transmitter freq.: 600.263707 MHz
time domain size: 65536 points
width: 12335.53 Hz = 20.5502 ppm = 0.188225 Hz/pt
number of scans: 16

freq. of 0 ppm: 600.260000 MHz
processed size: 32768 complex points
LB: 0.300 GF: 0.0000

SpinWorks 4: no title

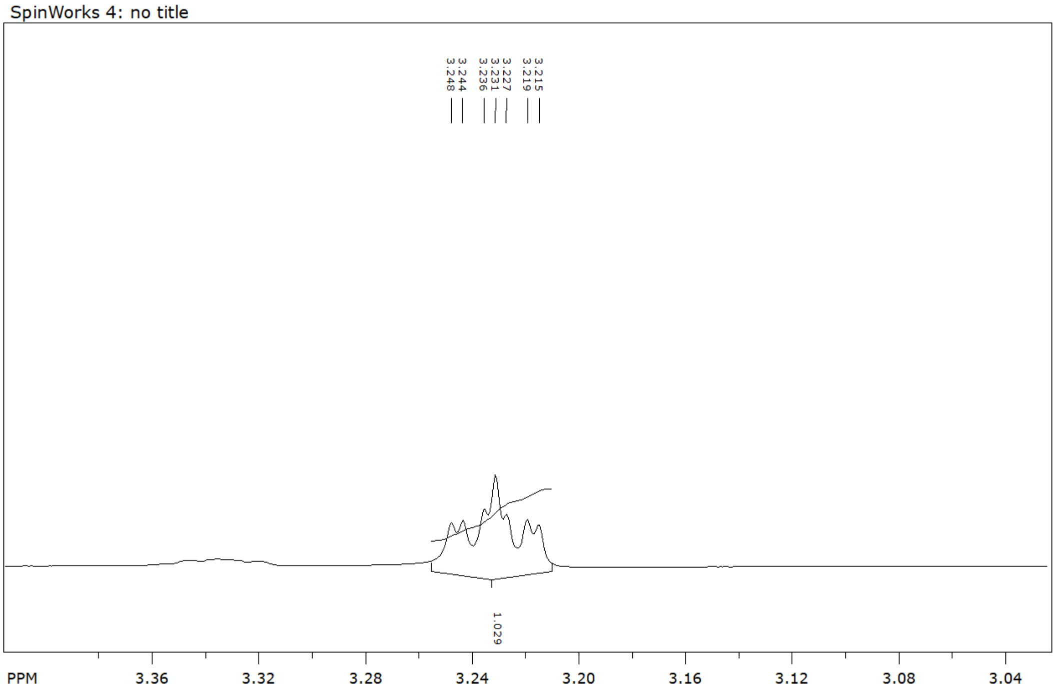

file: ... Łodzi\Desktop\nmr\mfl-2722\10\fid   expt: <zg30>
transmitter freq.: 600.263707 MHz
time domain size: 65536 points
width: 12335.53 Hz = 20.5502 ppm = 0.188225 Hz/pt
number of scans: 16

freq. of 0 ppm: 600.260000 MHz
processed size: 32768 complex points
LB: 0.300   GF: 0.0000

SpinWorks 4: no title

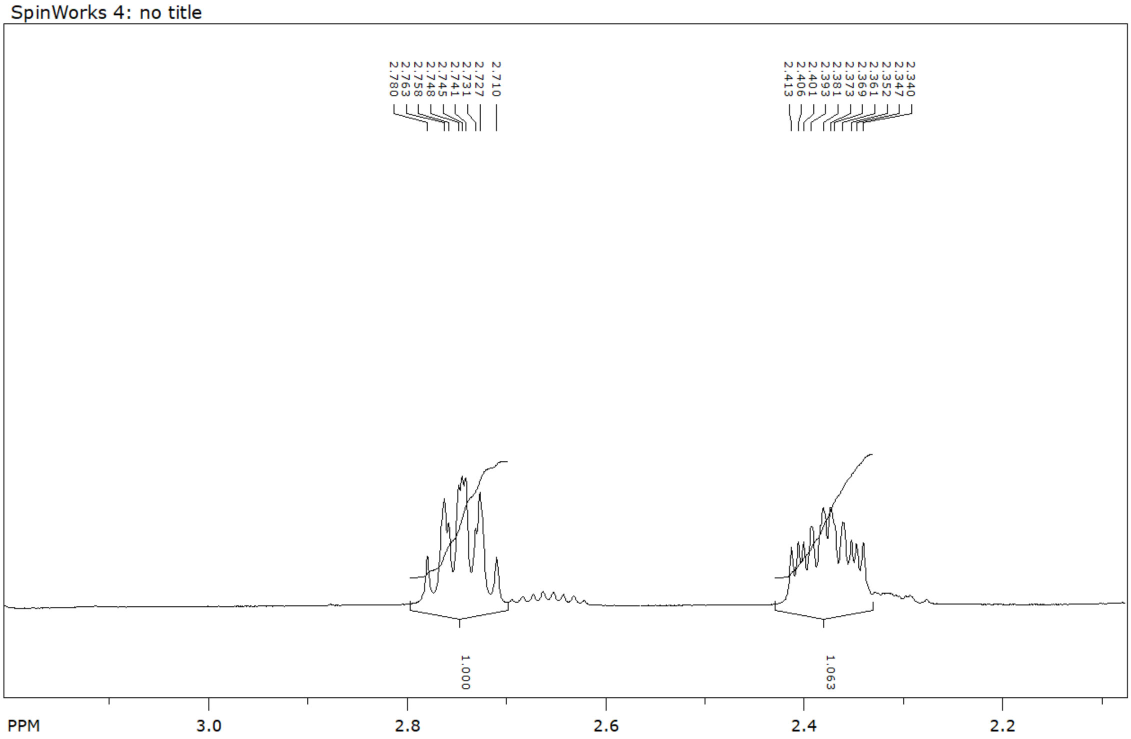

file: D:\mfl-2722\10\fid   expt: <zg30>
transmitter freq.: 600.263707 MHz
time domain size: 65536 points
width: 12335.53 Hz = 20.5502 ppm = 0.188225 Hz/pt
number of scans: 16

freq. of 0 ppm: 600.260000 MHz
processed size: 32768 complex points
LB: 0.300   GF: 0.0000

Figure S23: ^{31}P NMR Spectrum for mixture of *cis*-**16b**/*trans*-**16b** (90:10) in $CDCl_3$

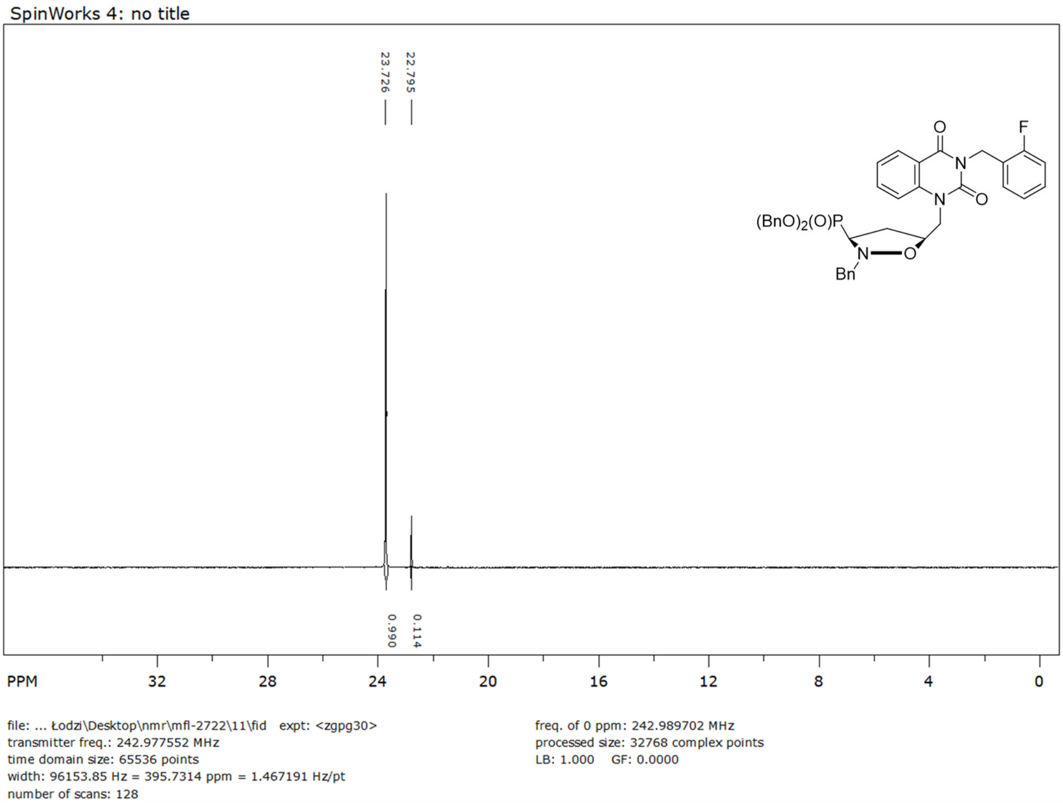

Figure S24: ^{13}C NMR Spectrum for mixture of *cis*-**16b**/*trans*-**16b** (90:10) in $CDCl_3$ and expanded spectral regions

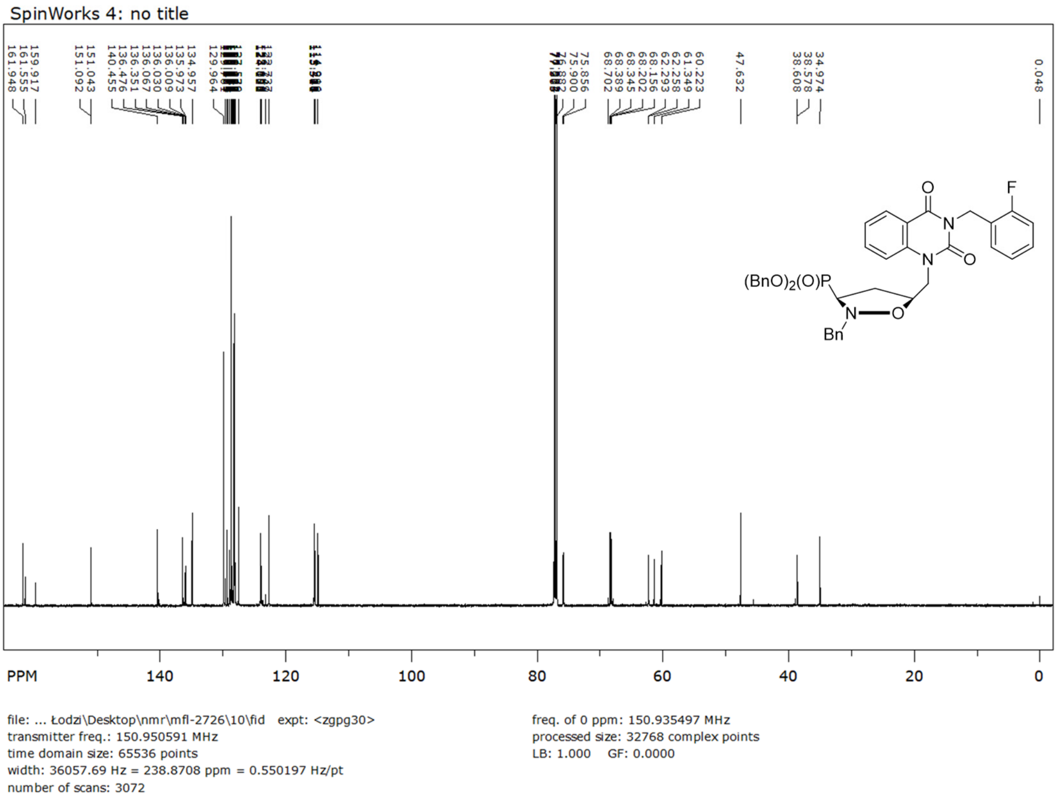

SpinWorks 4: no title

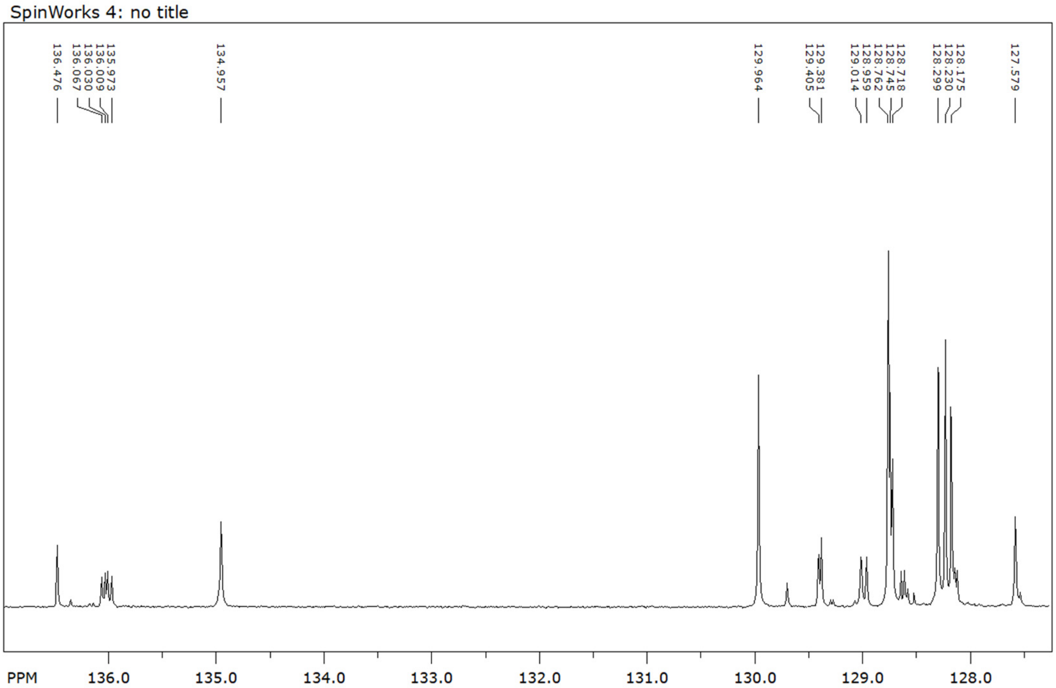

file: ...fl widma\widma 13C\mfl-2726\10\fid expt: <zgpg30>
transmitter freq.: 150.950591 MHz
time domain size: 65536 points
width: 36057.69 Hz = 238.8708 ppm = 0.550197 Hz/pt
number of scans: 3072

freq. of 0 ppm: 150.935497 MHz
processed size: 32768 complex points
LB: 1.000 GF: 0.0000

SpinWorks 4: no title

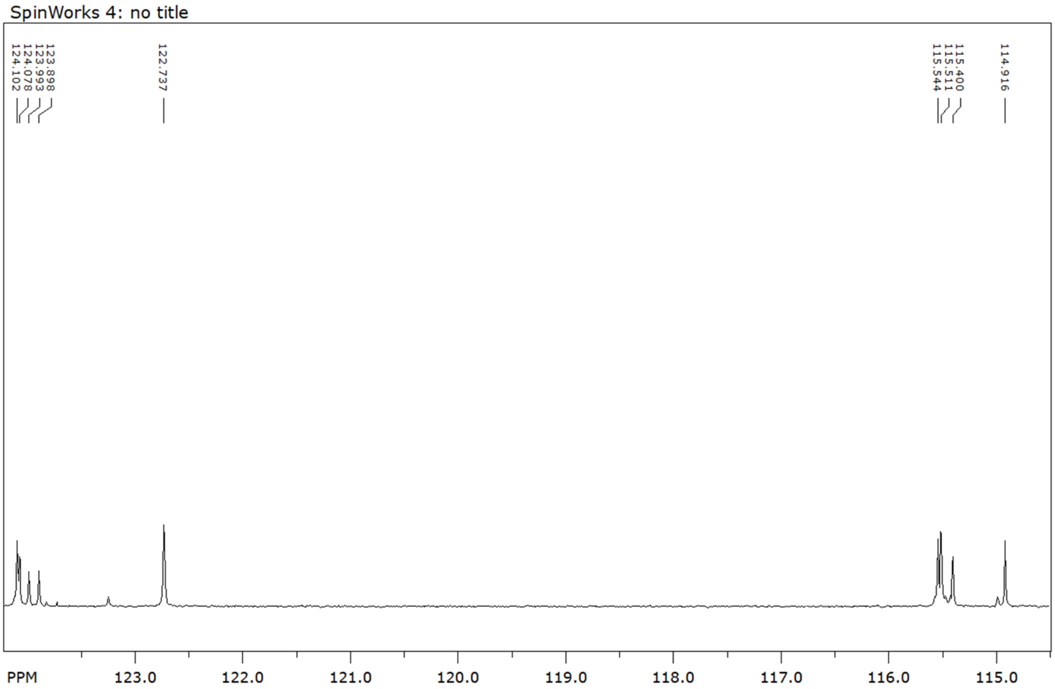

file: ...fl widma\widma 13C\mfl-2726\10\fid expt: <zgpg30>
transmitter freq.: 150.950591 MHz
time domain size: 65536 points
width: 36057.69 Hz = 238.8708 ppm = 0.550197 Hz/pt
number of scans: 3072

freq. of 0 ppm: 150.935497 MHz
processed size: 32768 complex points
LB: 1.000 GF: 0.0000

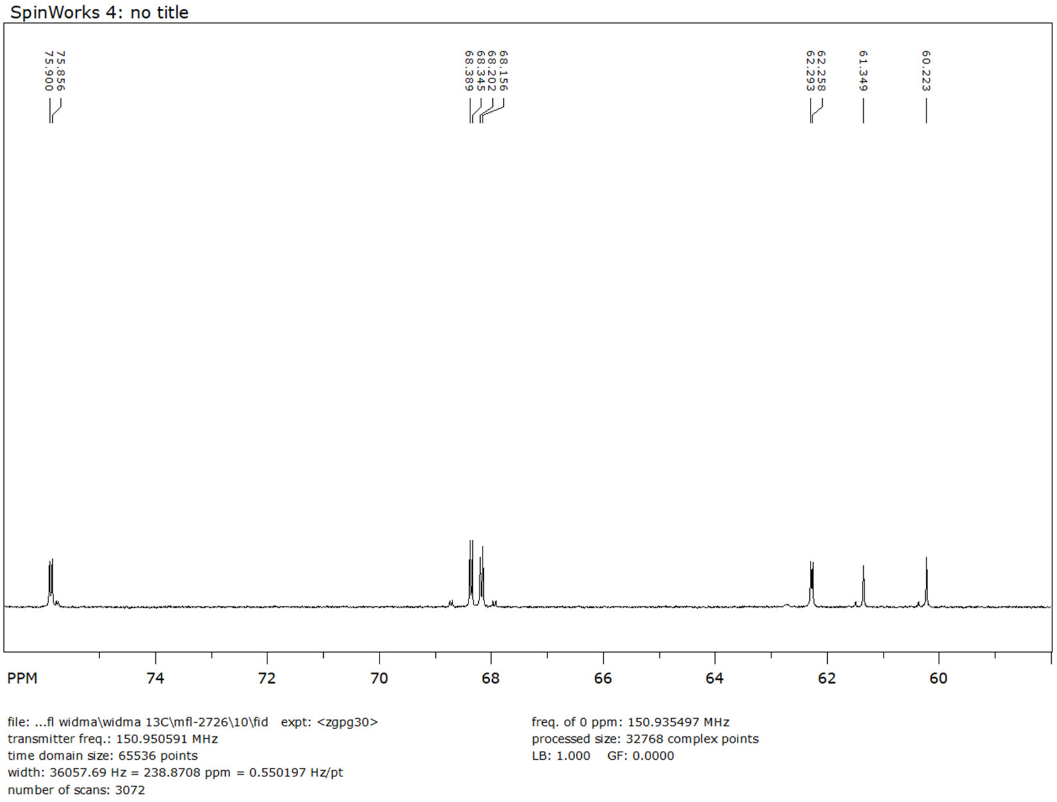

Figure S25: HPLC chromatogram for mixture of *cis*-**16b**/*trans*-**16b** (90:10)

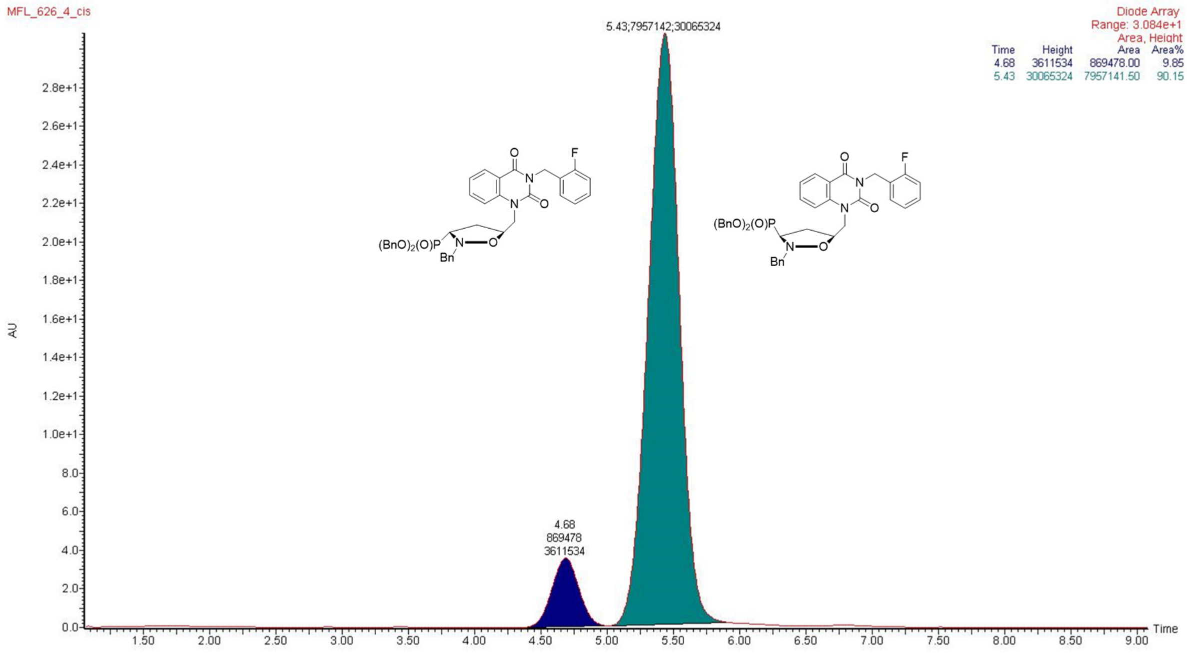

Figure S26: ^{1}H NMR Spectrum for *trans*-**16b** in $CDCl_3$ and expanded spectral regions

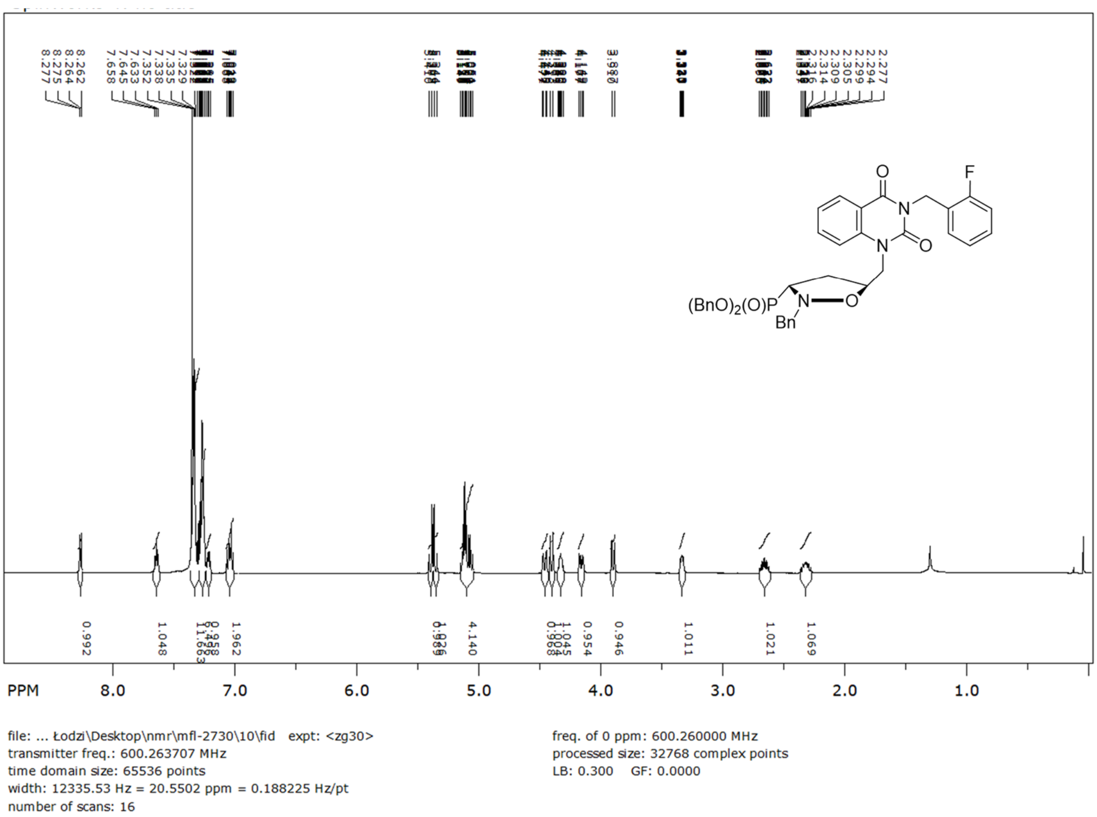

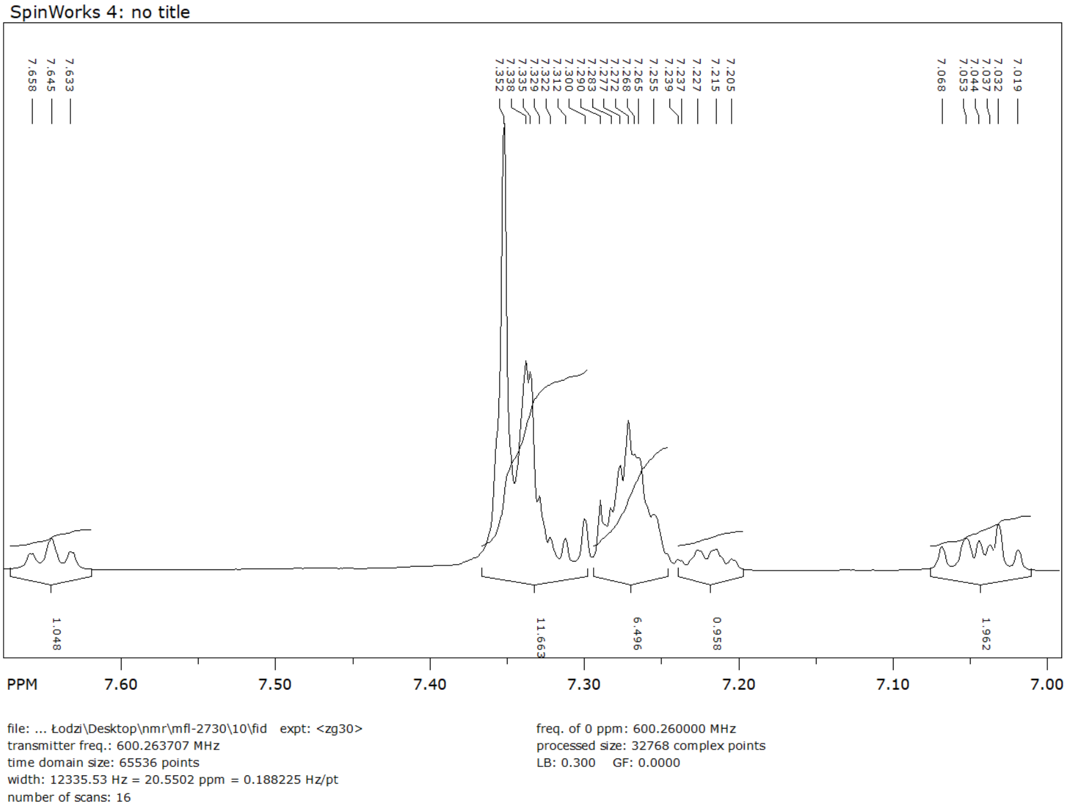

SpinWorks 4: no title

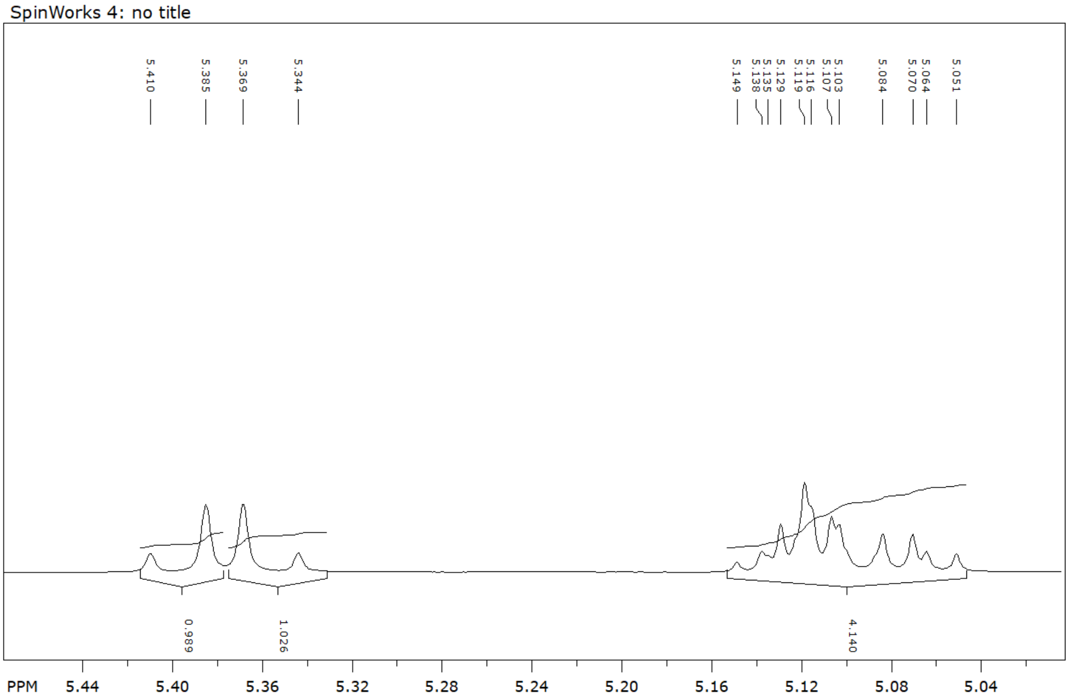

file: ... Łodzi\Desktop\nmr\mfl-2730\10\fid expt: <zg30>
transmitter freq.: 600.263707 MHz
time domain size: 65536 points
width: 12335.53 Hz = 20.5502 ppm = 0.188225 Hz/pt
number of scans: 16

freq. of 0 ppm: 600.260000 MHz
processed size: 32768 complex points
LB: 0.300 GF: 0.0000

SpinWorks 4: no title

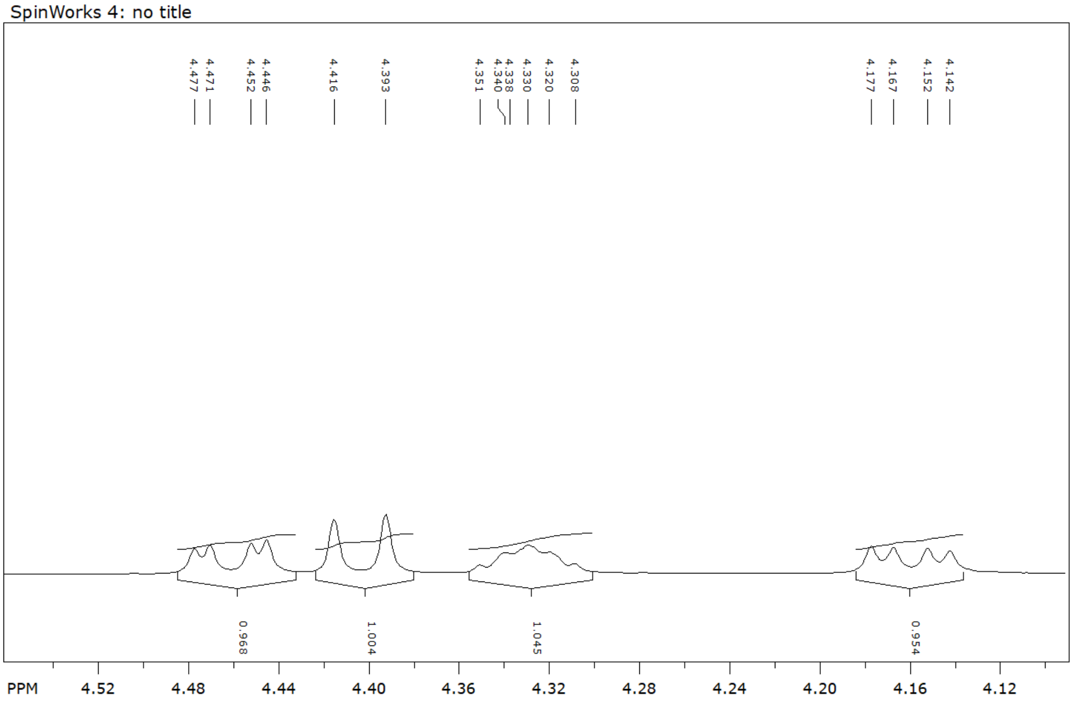

file: ... Łodzi\Desktop\nmr\mfl-2730\10\fid expt: <zg30>
transmitter freq.: 600.263707 MHz
time domain size: 65536 points
width: 12335.53 Hz = 20.5502 ppm = 0.188225 Hz/pt
number of scans: 16

freq. of 0 ppm: 600.260000 MHz
processed size: 32768 complex points
LB: 0.300 GF: 0.0000

SpinWorks 4: no title

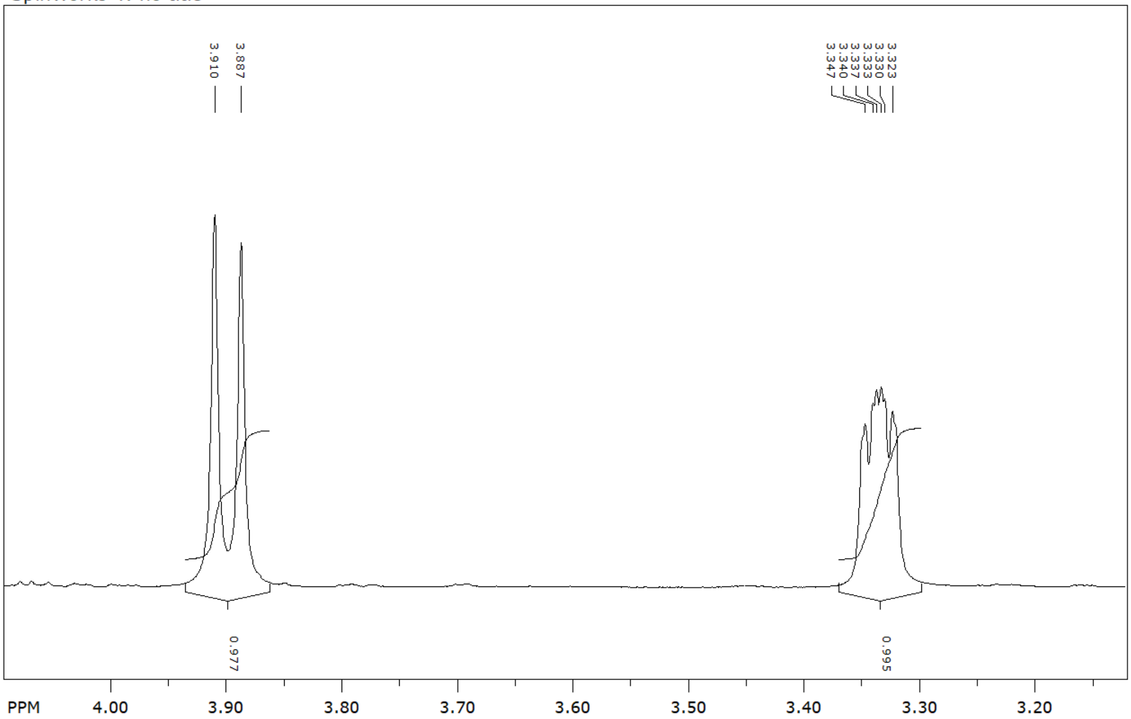

file: D:\mfl-2730\10\fid expt: <zg30>
transmitter freq.: 600.263707 MHz
time domain size: 65536 points
width: 12335.53 Hz = 20.5502 ppm = 0.188225 Hz/pt
number of scans: 16

freq. of 0 ppm: 600.260000 MHz
processed size: 32768 complex points
LB: 0.300 GF: 0.0000

SpinWorks 4: no title

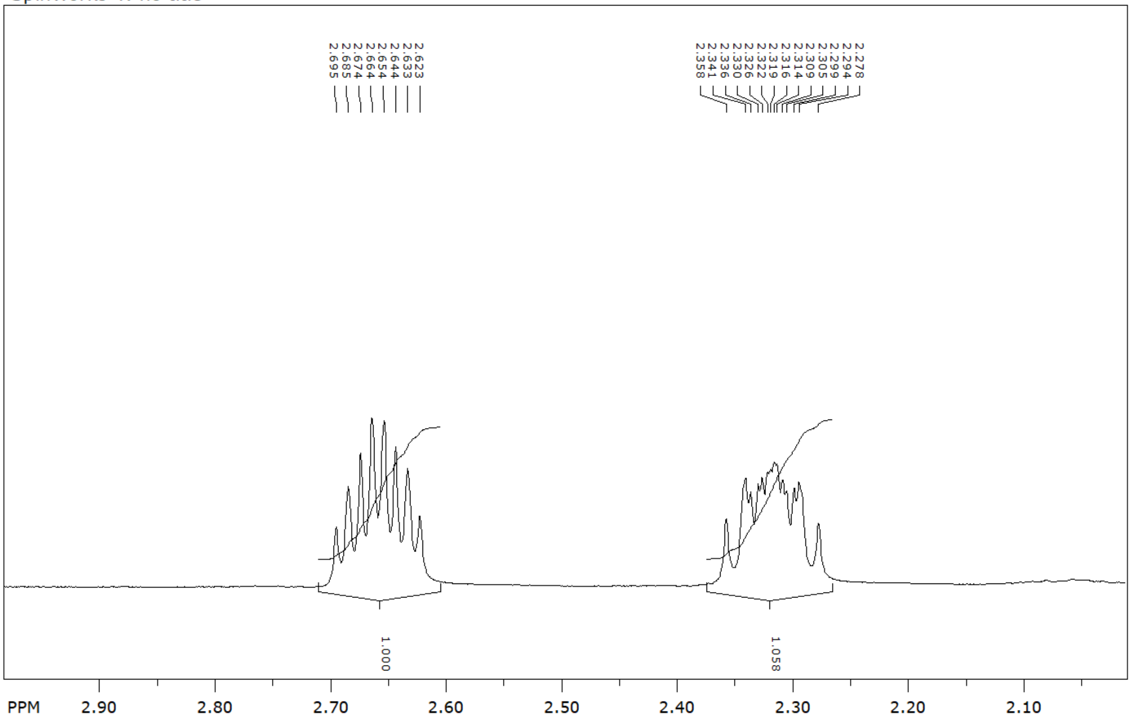

file: D:\mfl-2730\10\fid expt: <zg30>
transmitter freq.: 600.263707 MHz
time domain size: 65536 points
width: 12335.53 Hz = 20.5502 ppm = 0.188225 Hz/pt
number of scans: 16

freq. of 0 ppm: 600.260000 MHz
processed size: 32768 complex points
LB: 0.300 GF: 0.0000

Figure S27: ^{31}P NMR Spectrum for *trans*-**16b**in $CDCl_3$

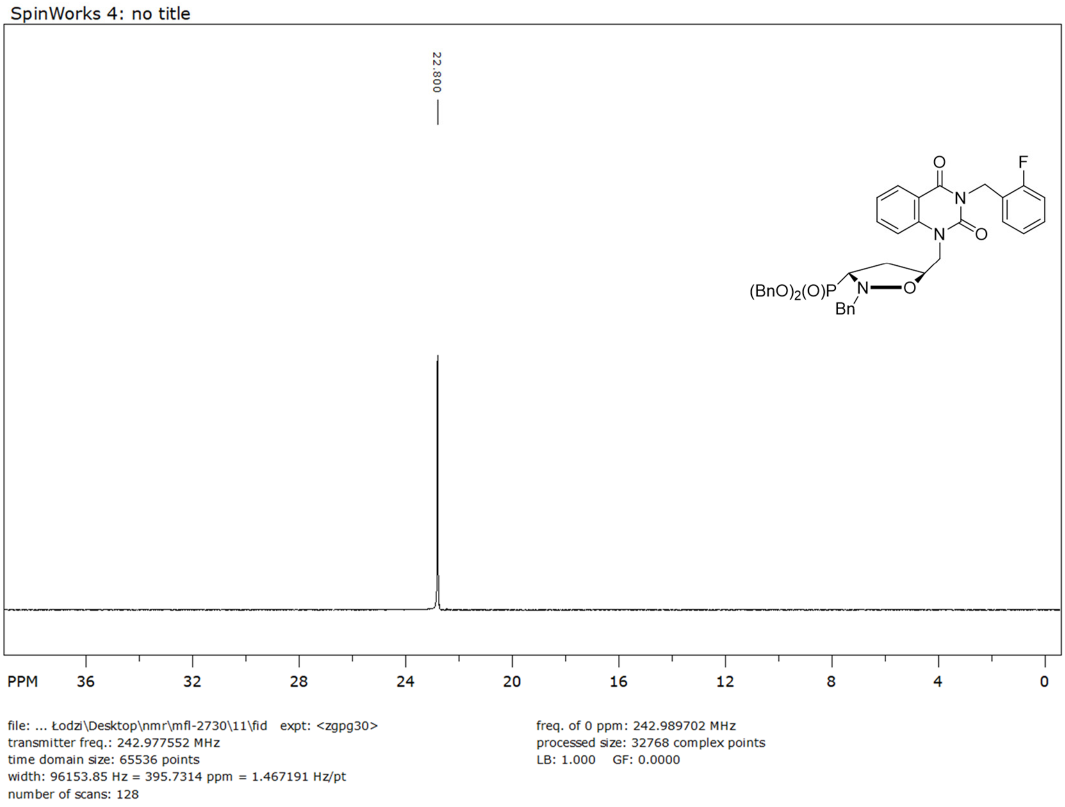

Figure S28: ^{13}C NMR Spectrum for *trans*-**16b** in $CDCl_3$ and expanded spectral regions

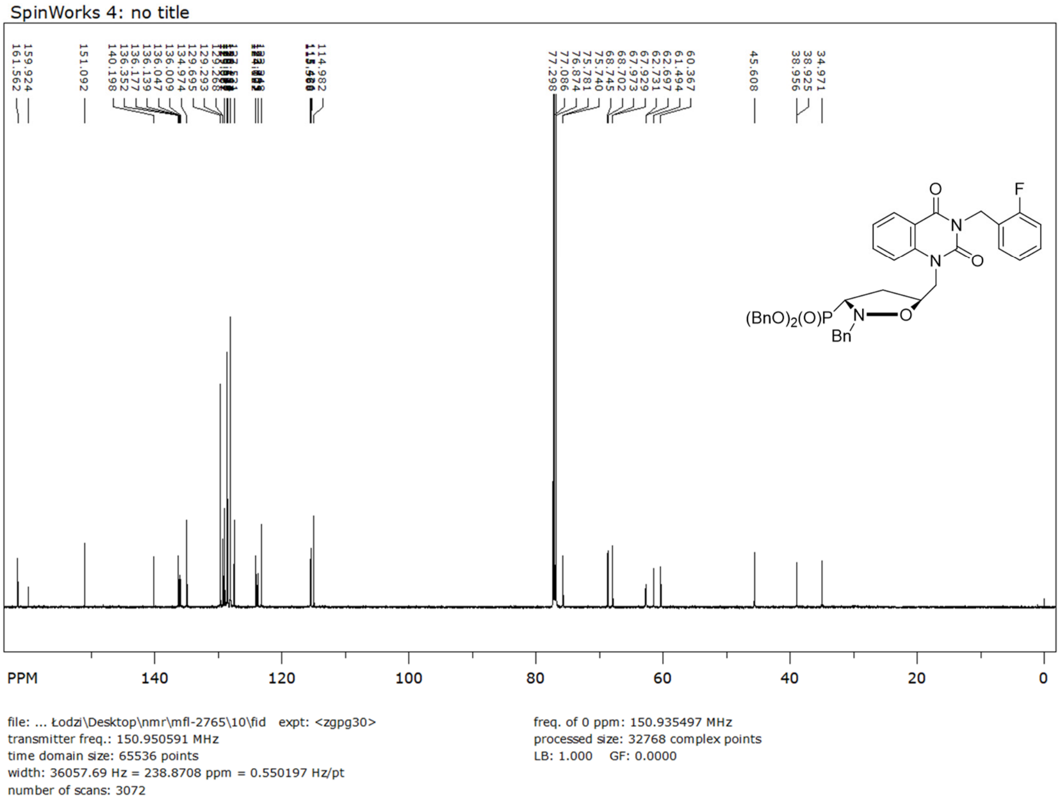

SpinWorks 4: no title

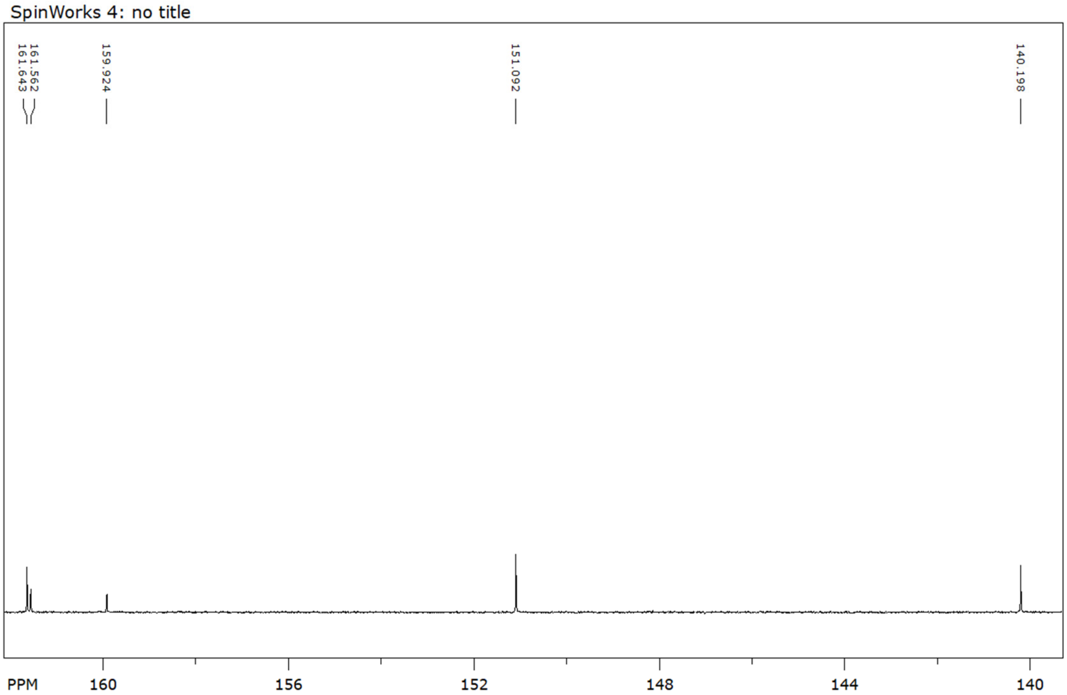

file: ...fl widma\widma 13C\mfl-2765\10\fid   expt: <zgpg30>
transmitter freq.: 150.950591 MHz
time domain size: 65536 points
width: 36057.69 Hz = 238.8708 ppm = 0.550197 Hz/pt
number of scans: 3072

freq. of 0 ppm: 150.935497 MHz
processed size: 32768 complex points
LB: 1.000   GF: 0.0000

SpinWorks 4: no title

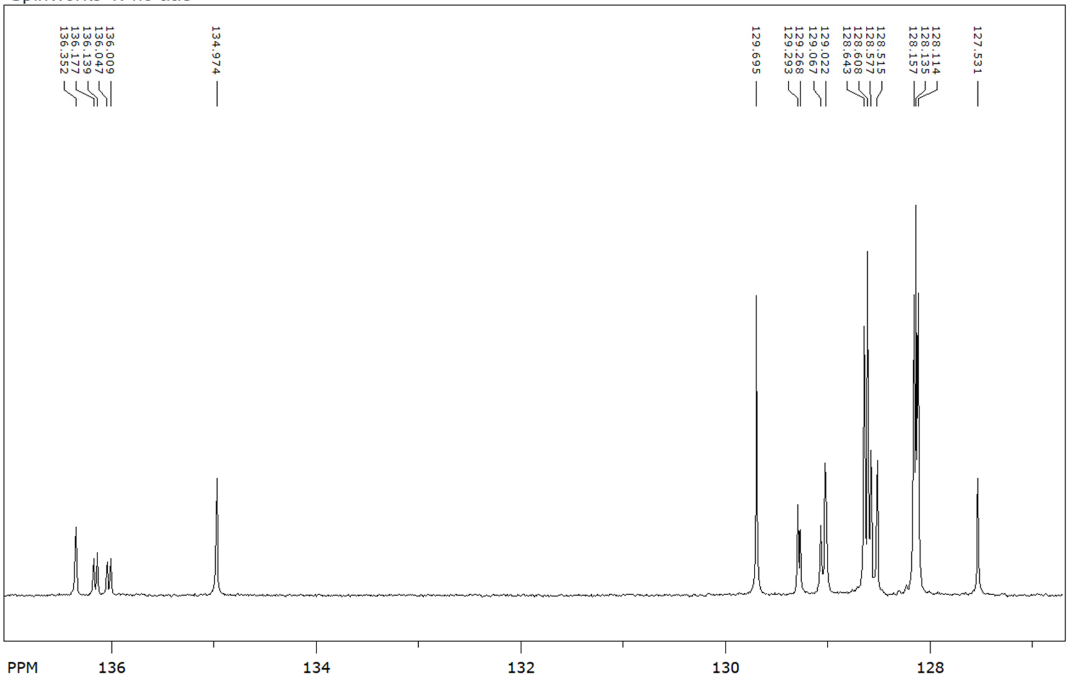

file: ...fl widma\widma 13C\mfl-2765\10\fid   expt: <zgpg30>
transmitter freq.: 150.950591 MHz
time domain size: 65536 points
width: 36057.69 Hz = 238.8708 ppm = 0.550197 Hz/pt
number of scans: 3072

freq. of 0 ppm: 150.935497 MHz
processed size: 32768 complex points
LB: 1.000   GF: 0.0000

SpinWorks 4: no title

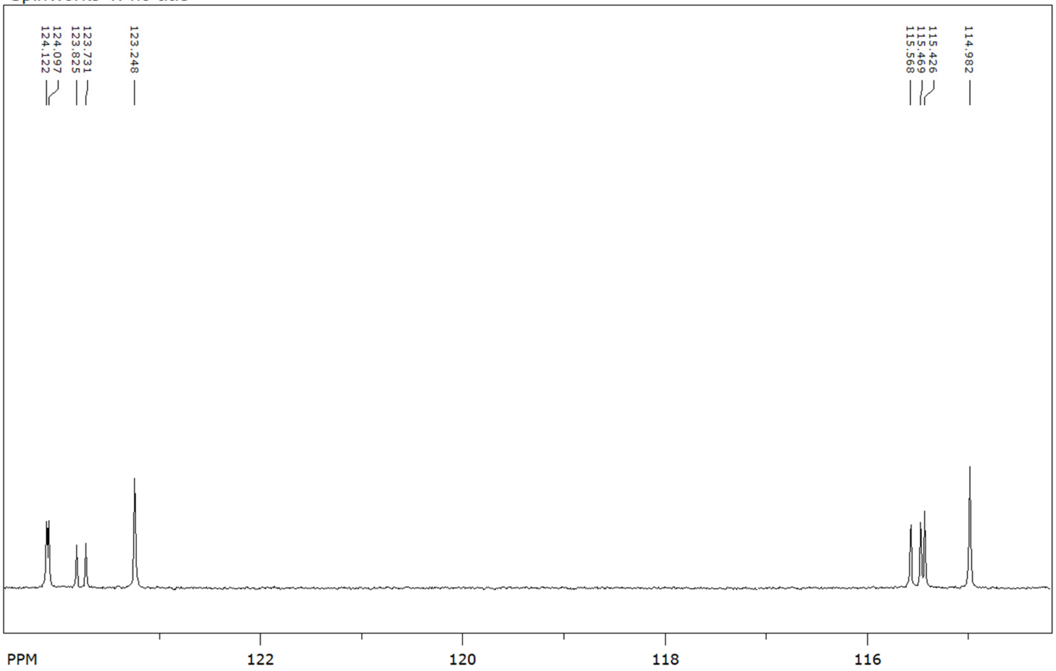

file: ...fl widma\widma 13C\mfl-2765\10\fid expt: <zgpg30>
transmitter freq.: 150.950591 MHz
time domain size: 65536 points
width: 36057.69 Hz = 238.8708 ppm = 0.550197 Hz/pt
number of scans: 3072

freq. of 0 ppm: 150.935497 MHz
processed size: 32768 complex points
LB: 1.000 GF: 0.0000

SpinWorks 4: no title

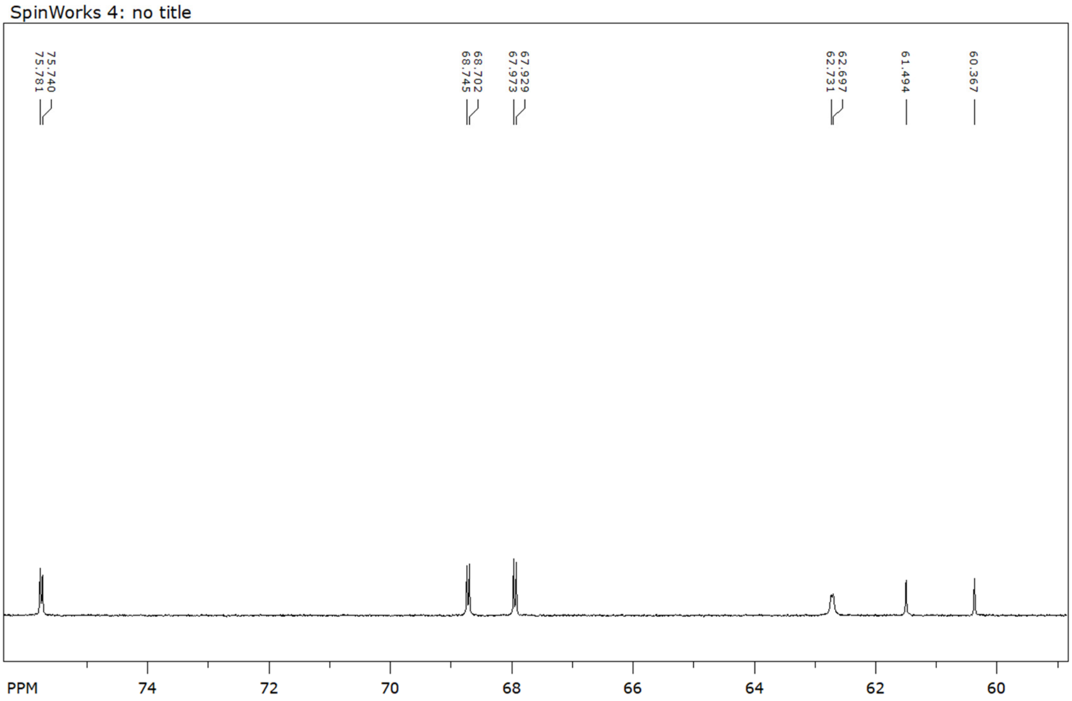

file: ...fl widma\widma 13C\mfl-2765\10\fid expt: <zgpg30>
transmitter freq.: 150.950591 MHz
time domain size: 65536 points
width: 36057.69 Hz = 238.8708 ppm = 0.550197 Hz/pt
number of scans: 3072

freq. of 0 ppm: 150.935497 MHz
processed size: 32768 complex points
LB: 1.000 GF: 0.0000

Figure S29: HPLC chromatogram for *trans*-**16b**

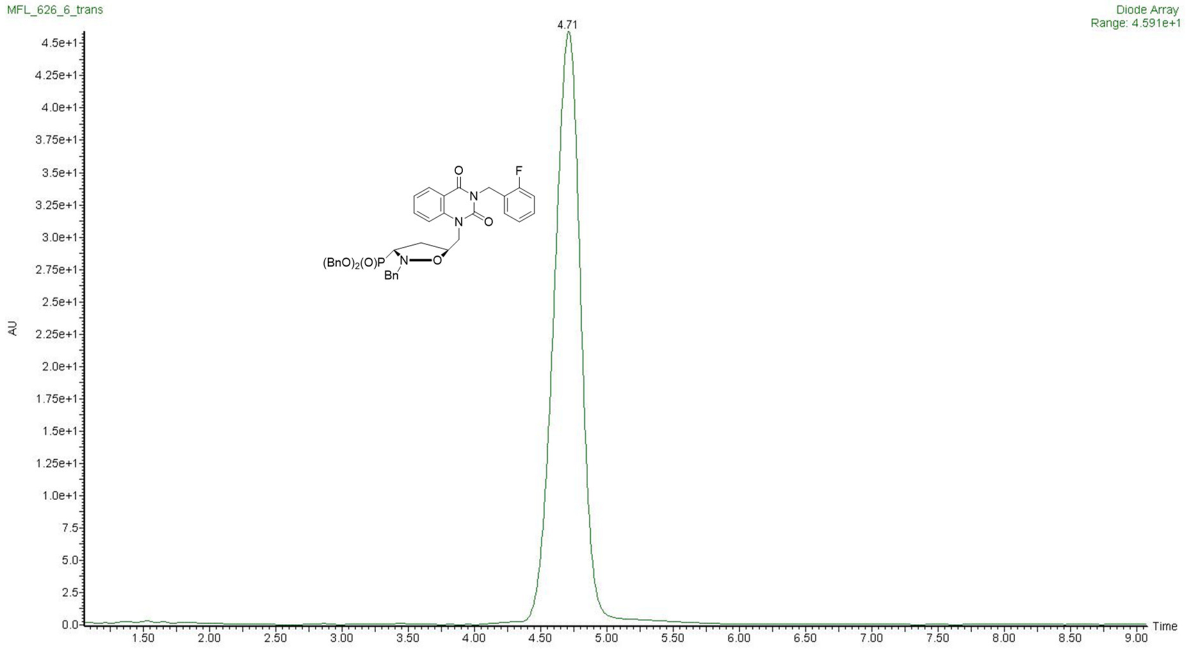

Figure S30: ^{1}H NMR Spectrum for mixture of *cis*-**16c**/*trans*-**16c** (90:10) in $CDCl_3$ and expanded spectral regions

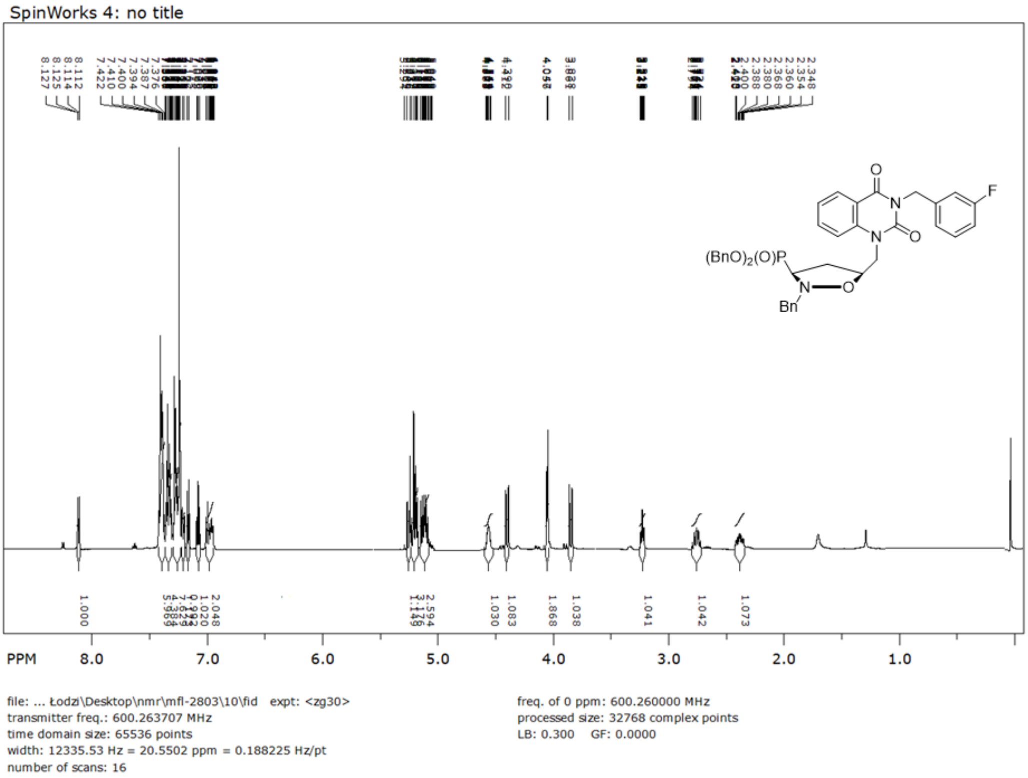

SpinWorks 4: no title

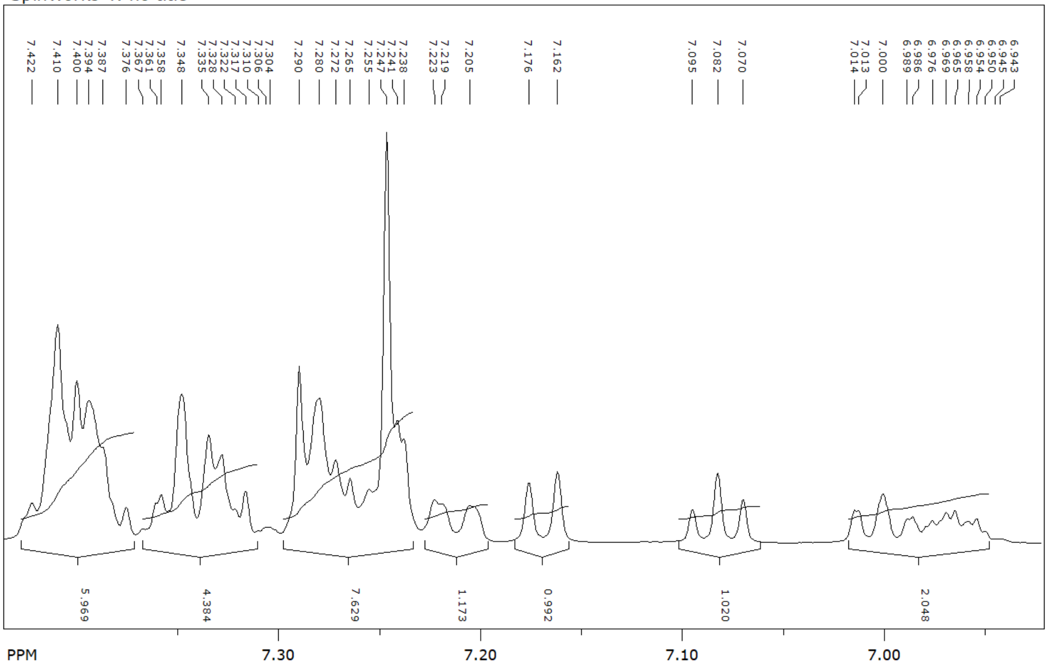

file: ... Łodzi\Desktop\nmr\mfl-2803\10\fid expt: <zg30>
transmitter freq.: 600.263707 MHz
time domain size: 65536 points
width: 12335.53 Hz = 20.5502 ppm = 0.188225 Hz/pt
number of scans: 16

freq. of 0 ppm: 600.260000 MHz
processed size: 32768 complex points
LB: 0.300 GF: 0.0000

SpinWorks 4: no title

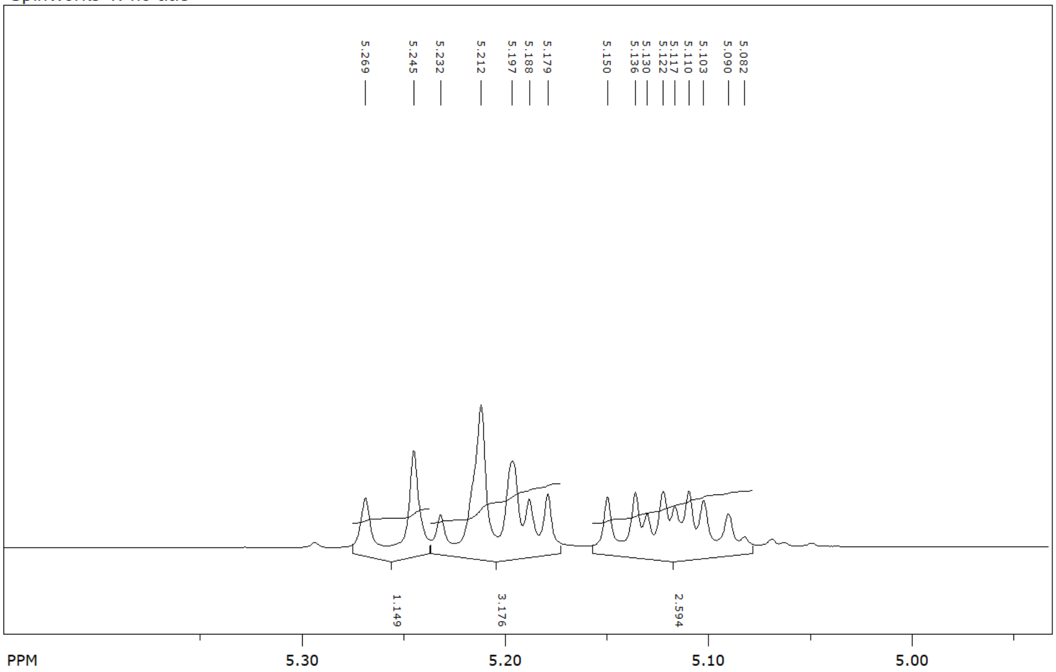

file: ... Łodzi\Desktop\nmr\mfl-2803\10\fid expt: <zg30>
transmitter freq.: 600.263707 MHz
time domain size: 65536 points
width: 12335.53 Hz = 20.5502 ppm = 0.188225 Hz/pt
number of scans: 16

freq. of 0 ppm: 600.260000 MHz
processed size: 32768 complex points
LB: 0.300 GF: 0.0000

SpinWorks 4: no title

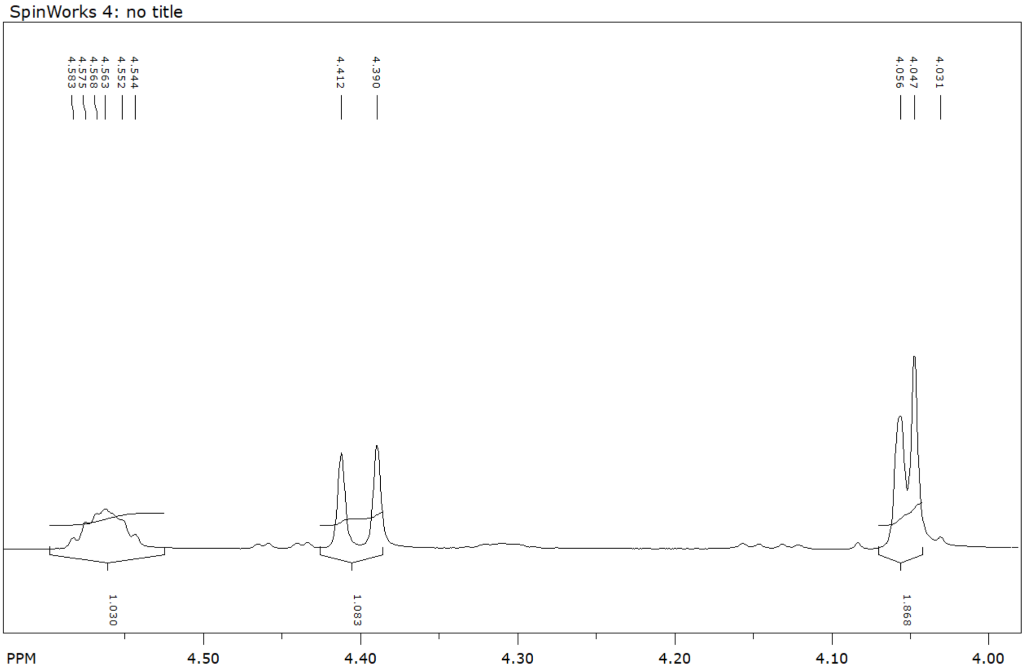

file: ... Łodzi\Desktop\nmr\mfl-2803\10\fid expt: <zg30>
transmitter freq.: 600.263707 MHz
time domain size: 65536 points
width: 12335.53 Hz = 20.5502 ppm = 0.188225 Hz/pt
number of scans: 16

freq. of 0 ppm: 600.260000 MHz
processed size: 32768 complex points
LB: 0.300 GF: 0.0000

SpinWorks 4: no title

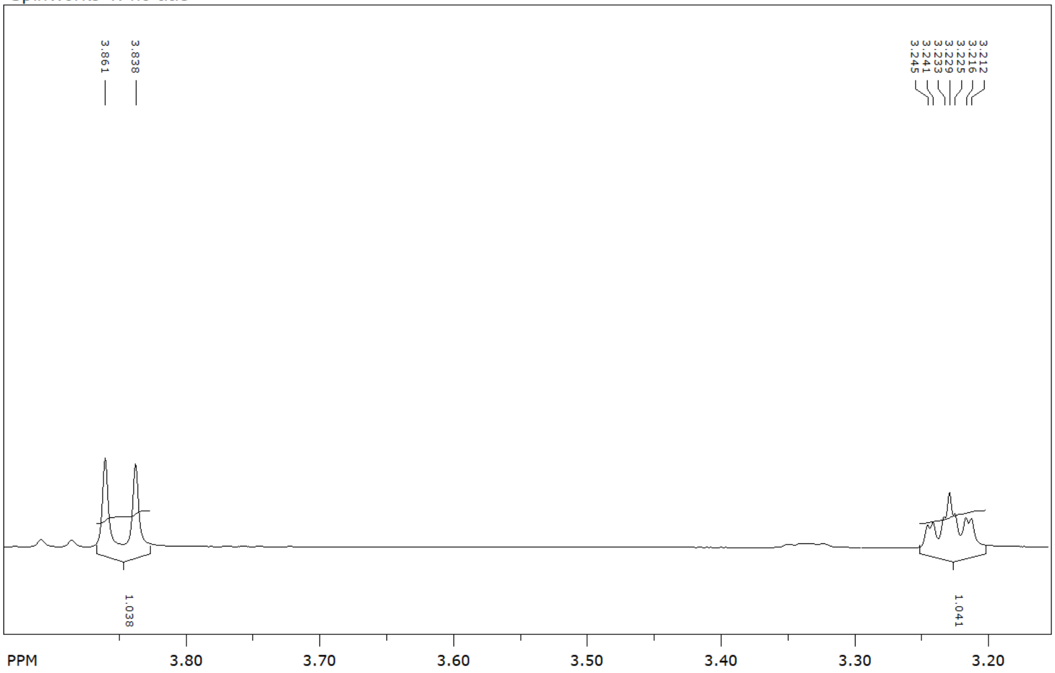

file: ... Łodzi\Desktop\nmr\mfl-2803\10\fid expt: <zg30>
transmitter freq.: 600.263707 MHz
time domain size: 65536 points
width: 12335.53 Hz = 20.5502 ppm = 0.188225 Hz/pt
number of scans: 16

freq. of 0 ppm: 600.260000 MHz
processed size: 32768 complex points
LB: 0.300 GF: 0.0000

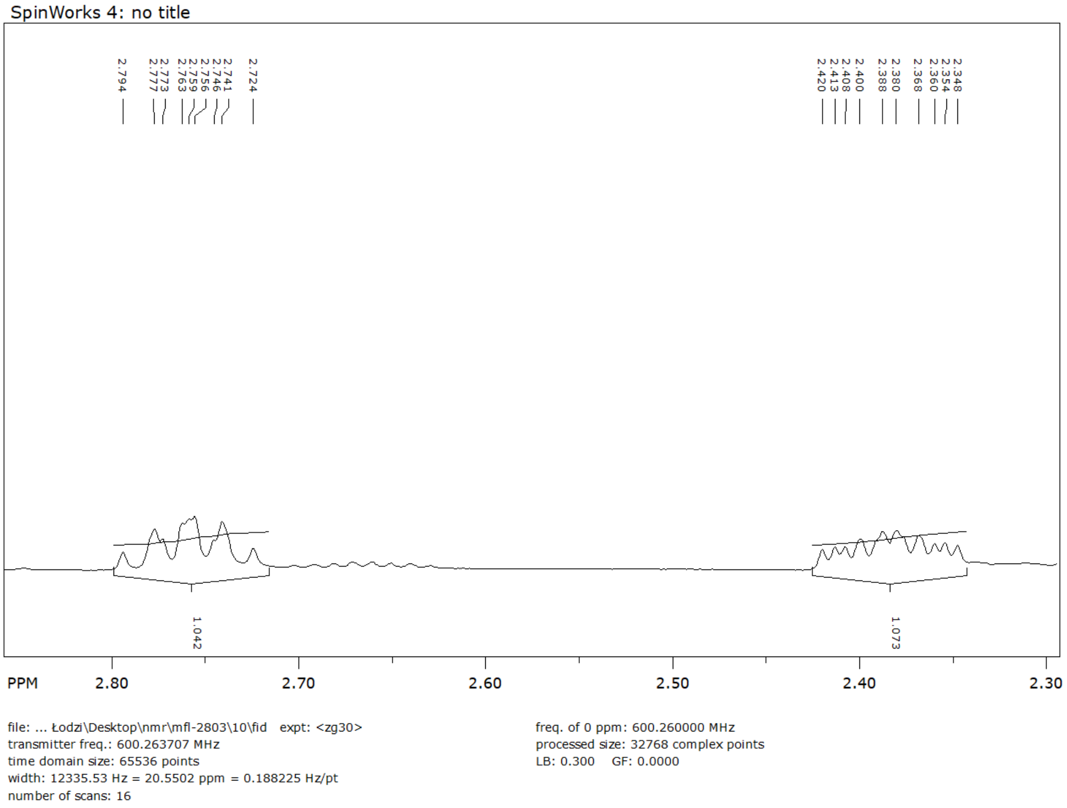

Figure S31: ^{31}P NMR Spectrum for mixture of *cis*-**16c**/*trans*-**16c** (90:10) in $CDCl_3$ and expanded spectral regions

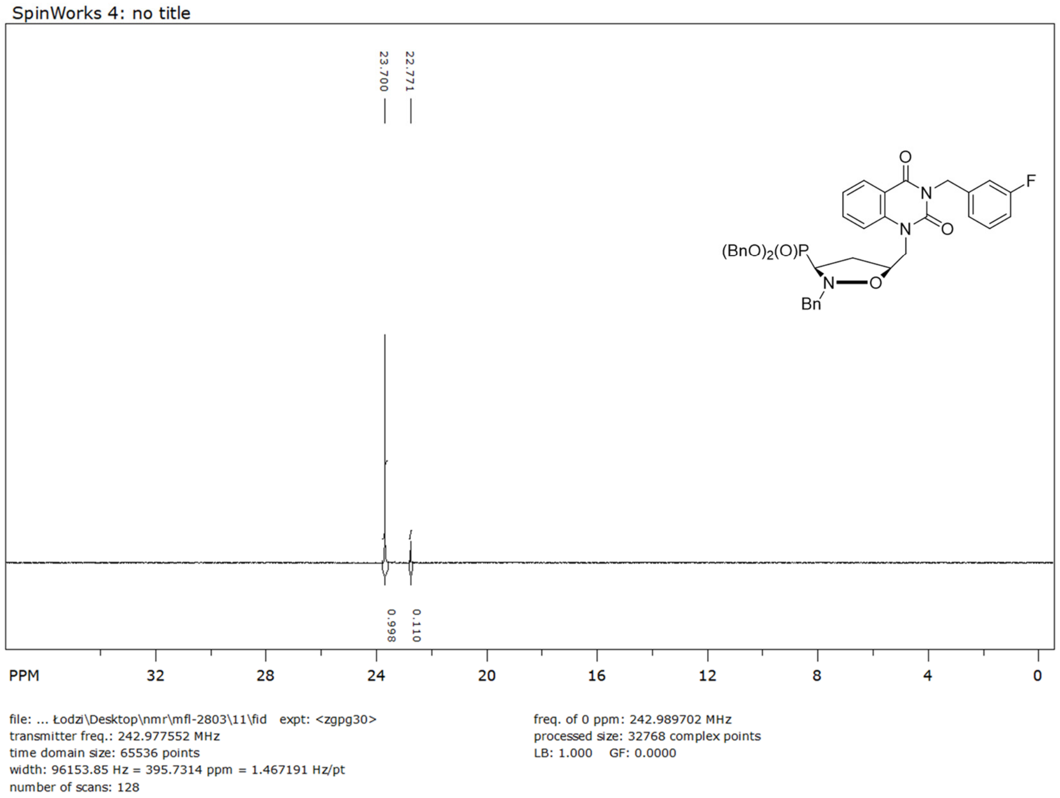

Figure S32: ^{13}C NMR Spectrum for mixture of *cis*-**16c**/*trans*-**16c** (90:10) in $CDCl_3$ and expanded spectral regions

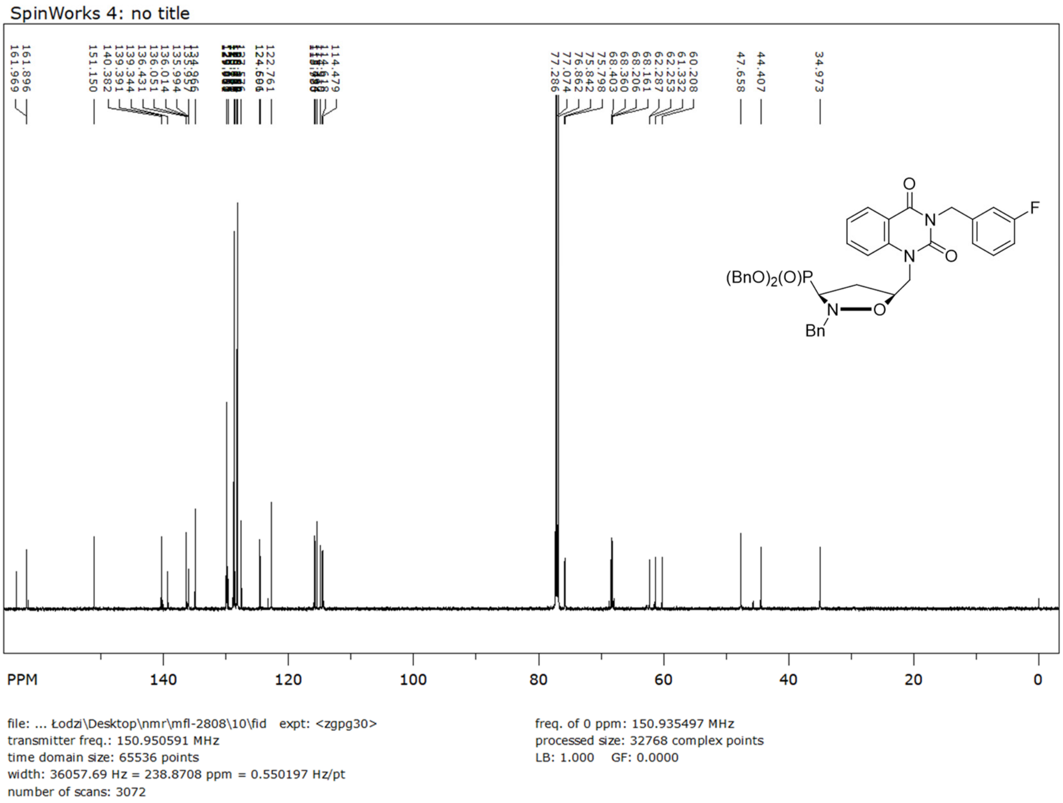

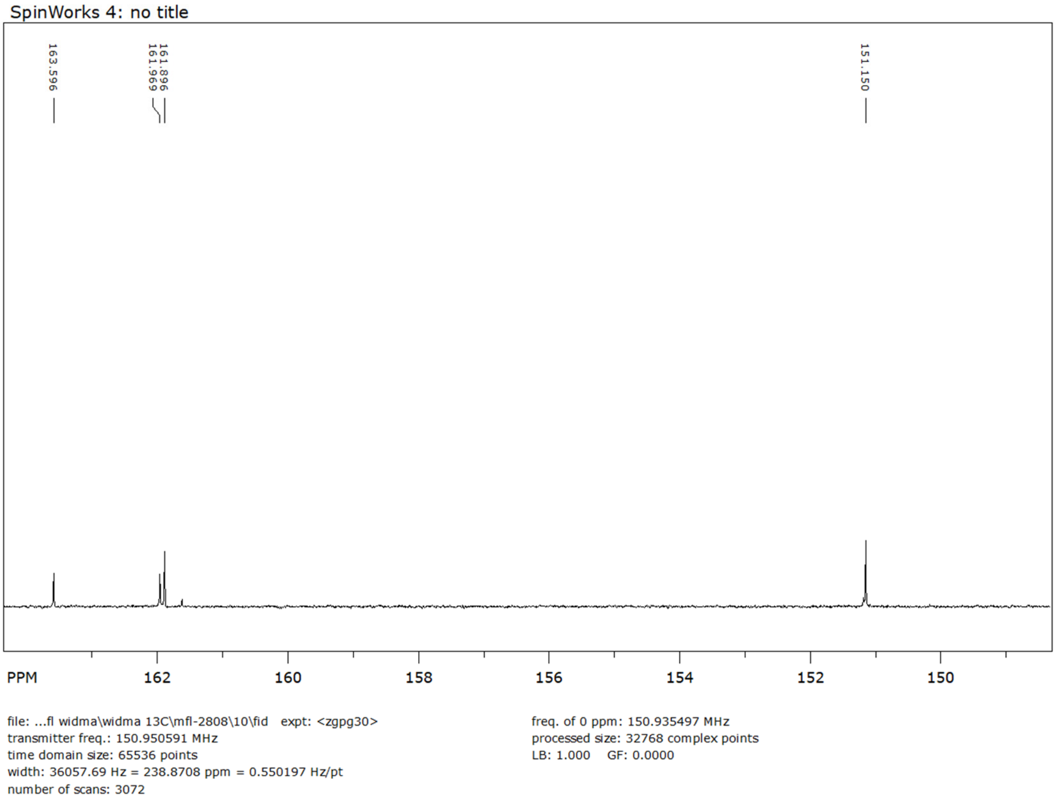

SpinWorks 4: no title

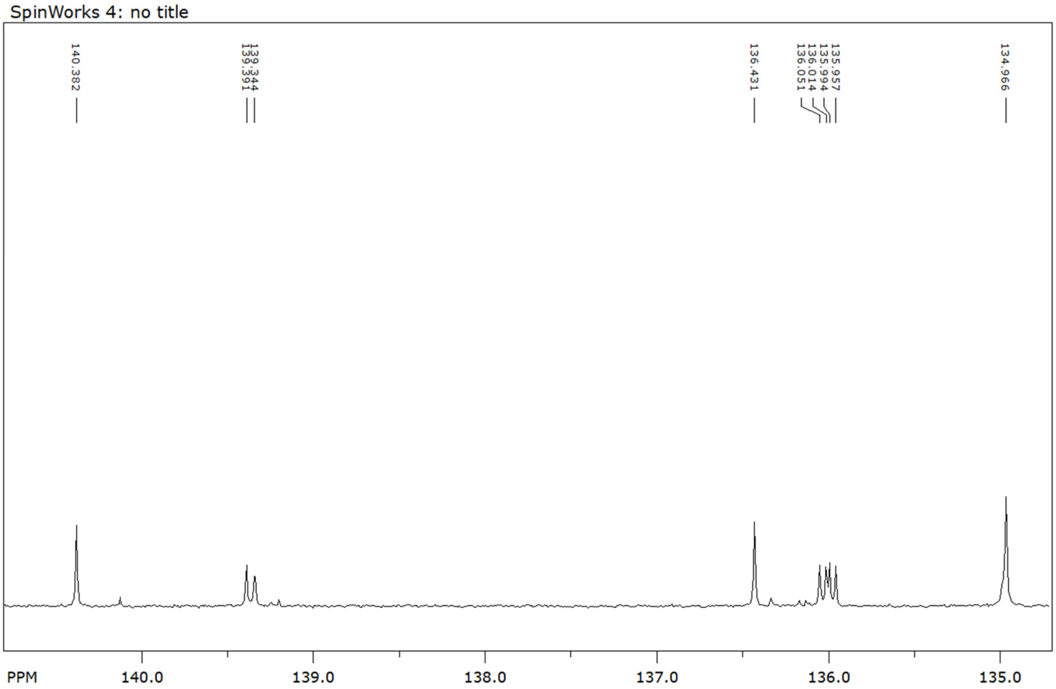

file: ...fl widma\widma 13C\mfl-2808\10\fid expt: <zgpg30>
transmitter freq.: 150.950591 MHz
time domain size: 65536 points
width: 36057.69 Hz = 238.8708 ppm = 0.550197 Hz/pt
number of scans: 3072

freq. of 0 ppm: 150.935497 MHz
processed size: 32768 complex points
LB: 1.000 GF: 0.0000

SpinWorks 4: no title

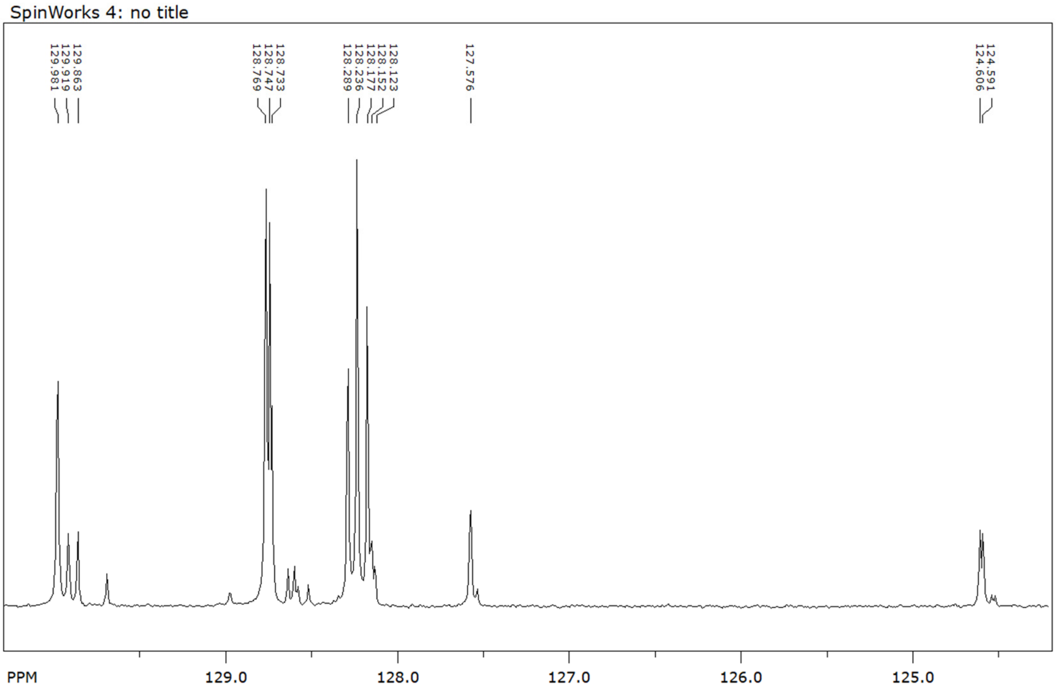

file: ...fl widma\widma 13C\mfl-2808\10\fid expt: <zgpg30>
transmitter freq.: 150.950591 MHz
time domain size: 65536 points
width: 36057.69 Hz = 238.8708 ppm = 0.550197 Hz/pt
number of scans: 3072

freq. of 0 ppm: 150.935497 MHz
processed size: 32768 complex points
LB: 1.000 GF: 0.0000

SpinWorks 4: no title

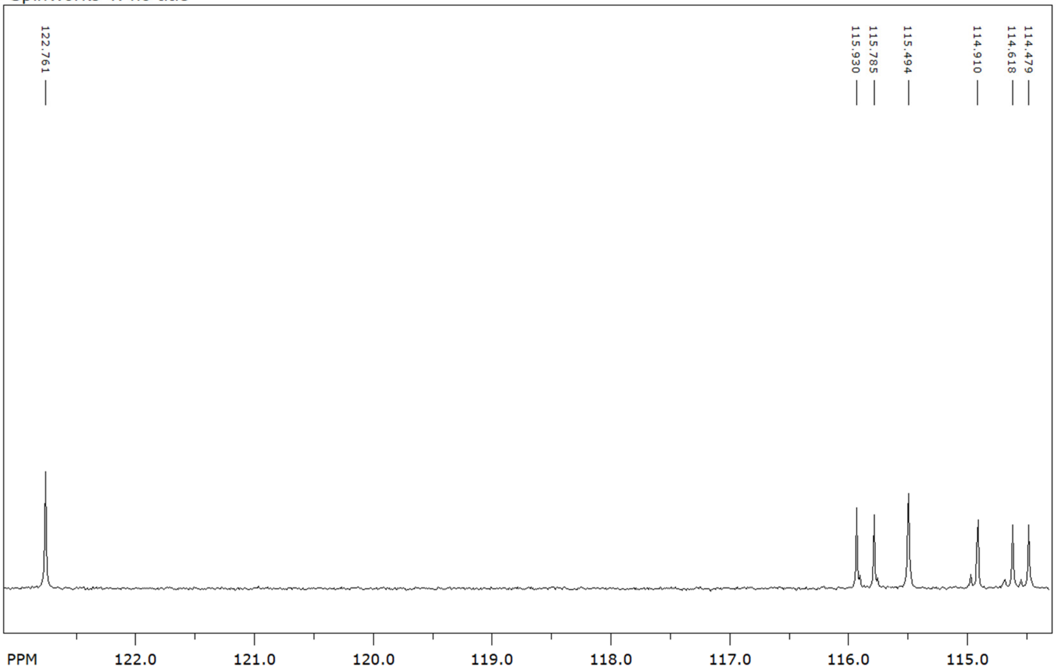

file: ...fl widma\widma 13C\mfl-2808\10\fid expt: <zgpg30>
transmitter freq.: 150.950591 MHz
time domain size: 65536 points
width: 36057.69 Hz = 238.8708 ppm = 0.550197 Hz/pt
number of scans: 3072

freq. of 0 ppm: 150.935497 MHz
processed size: 32768 complex points
LB: 1.000 GF: 0.0000

SpinWorks 4: no title

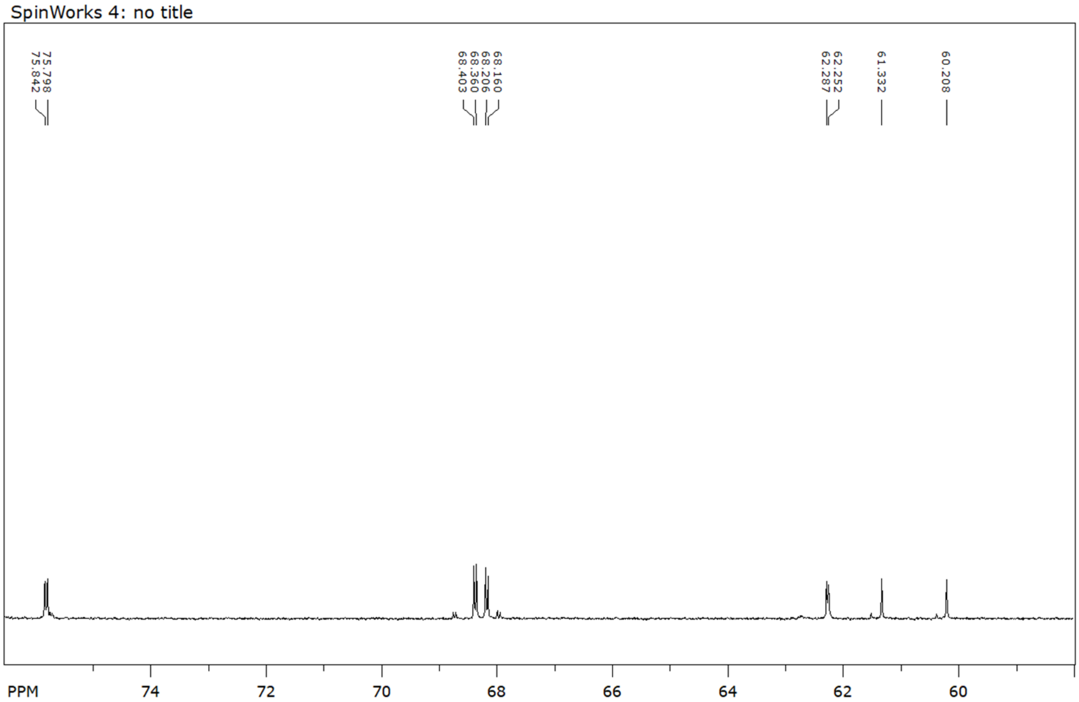

file: ...fl widma\widma 13C\mfl-2808\10\fid expt: <zgpg30>
transmitter freq.: 150.950591 MHz
time domain size: 65536 points
width: 36057.69 Hz = 238.8708 ppm = 0.550197 Hz/pt
number of scans: 3072

freq. of 0 ppm: 150.935497 MHz
processed size: 32768 complex points
LB: 1.000 GF: 0.0000

Figure S33: HPLC chromatogram for mixture of *cis*-**16c**/*trans*-**16c** (90:10)

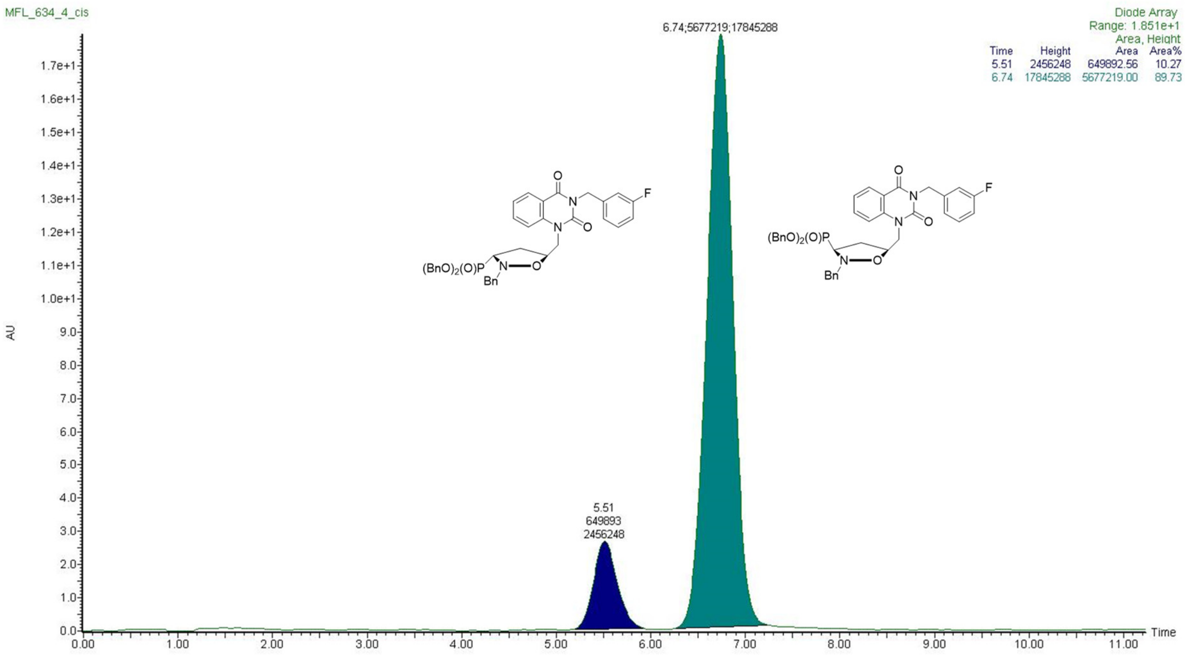

Figure S34: ^{1}H NMR Spectrum for *trans*-**16c** in $CDCl_3$ and expanded spectral regions

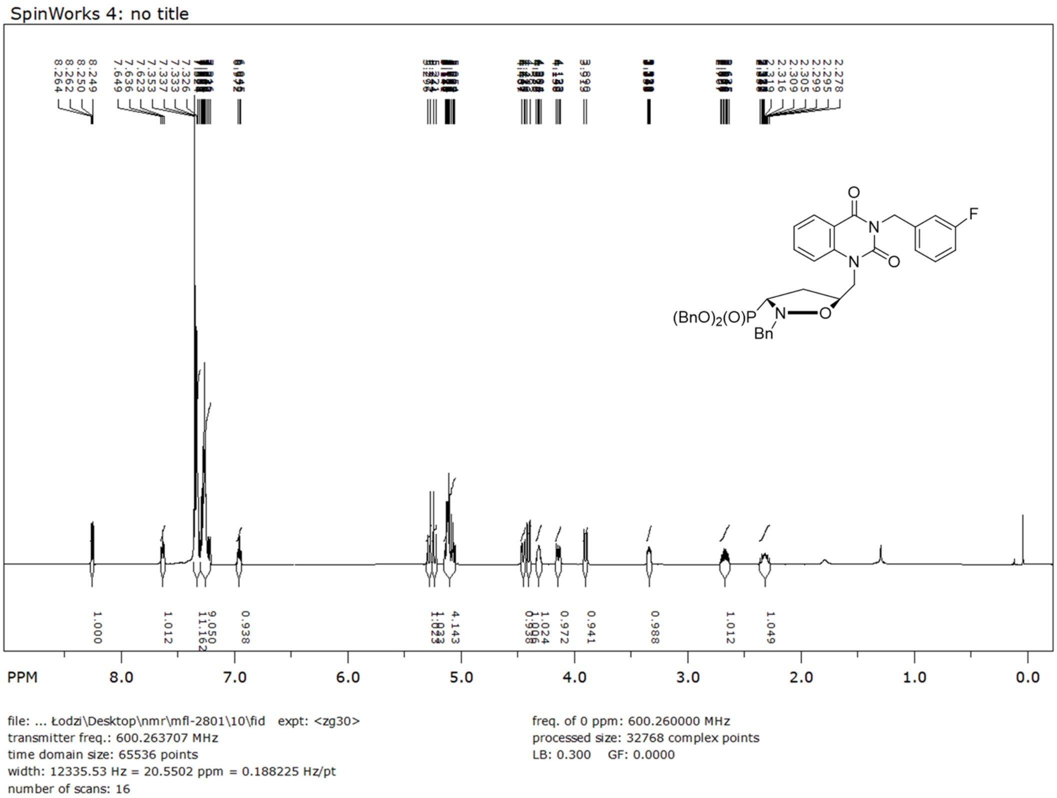

SpinWorks 4: no title

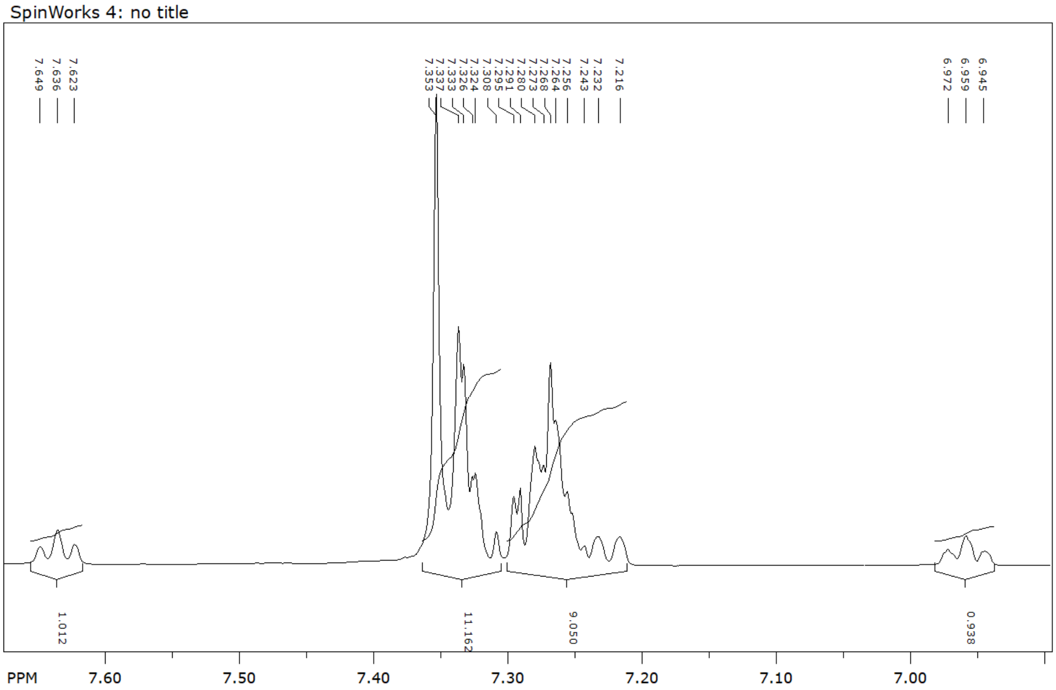

file: ... Łodzi\Desktop\nmr\mfl-2801\10\fid expt: <zg30>
transmitter freq.: 600.263707 MHz
time domain size: 65536 points
width: 12335.53 Hz = 20.5502 ppm = 0.188225 Hz/pt
number of scans: 16

freq. of 0 ppm: 600.260000 MHz
processed size: 32768 complex points
LB: 0.300 GF: 0.0000

SpinWorks 4: no title

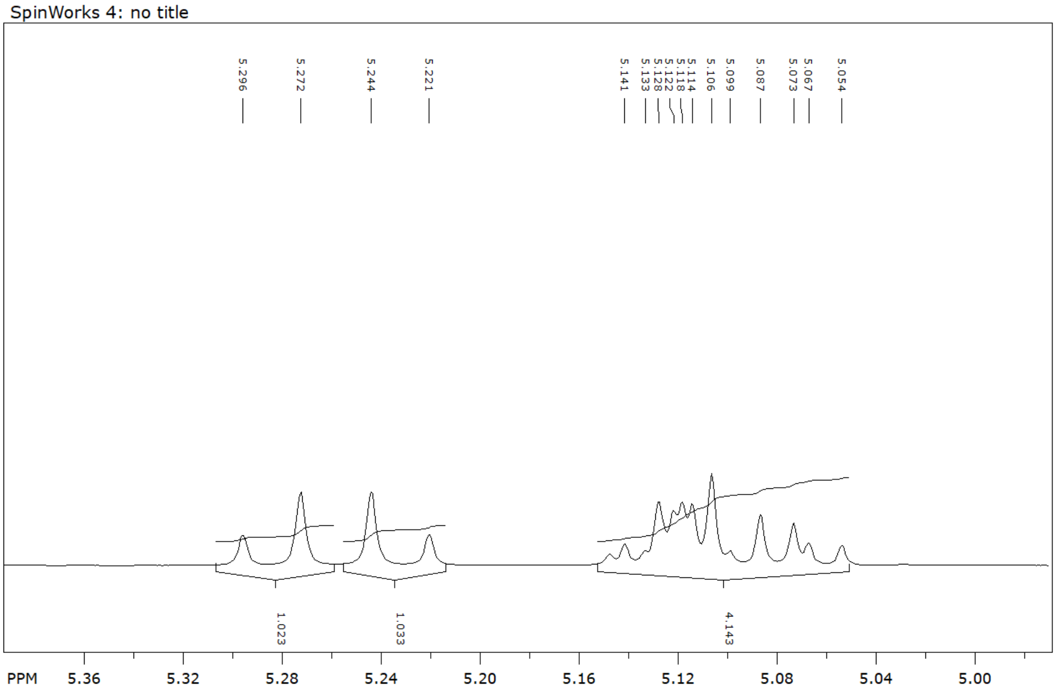

file: ... Łodzi\Desktop\nmr\mfl-2801\10\fid expt: <zg30>
transmitter freq.: 600.263707 MHz
time domain size: 65536 points
width: 12335.53 Hz = 20.5502 ppm = 0.188225 Hz/pt
number of scans: 16

freq. of 0 ppm: 600.260000 MHz
processed size: 32768 complex points
LB: 0.300 GF: 0.0000

SpinWorks 4: no title

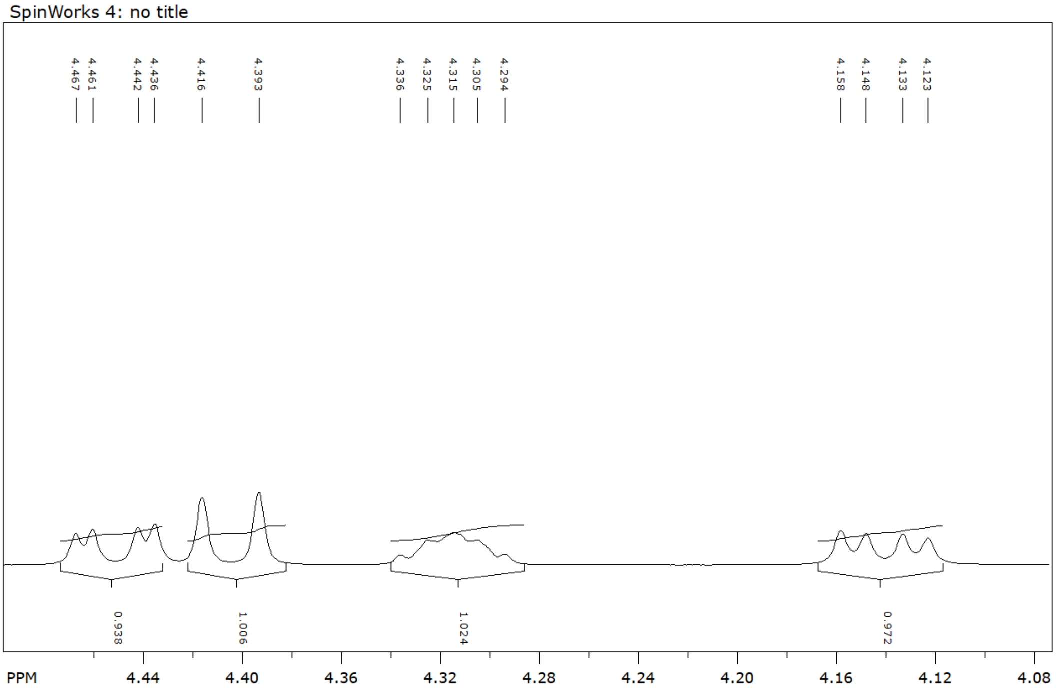

file: ... Łodzi\Desktop\nmr\mfl-2801\10\fid   expt: <zg30>
transmitter freq.: 600.263707 MHz
time domain size: 65536 points
width: 12335.53 Hz = 20.5502 ppm = 0.188225 Hz/pt
number of scans: 16

freq. of 0 ppm: 600.260000 MHz
processed size: 32768 complex points
LB: 0.300   GF: 0.0000

SpinWorks 4: no title

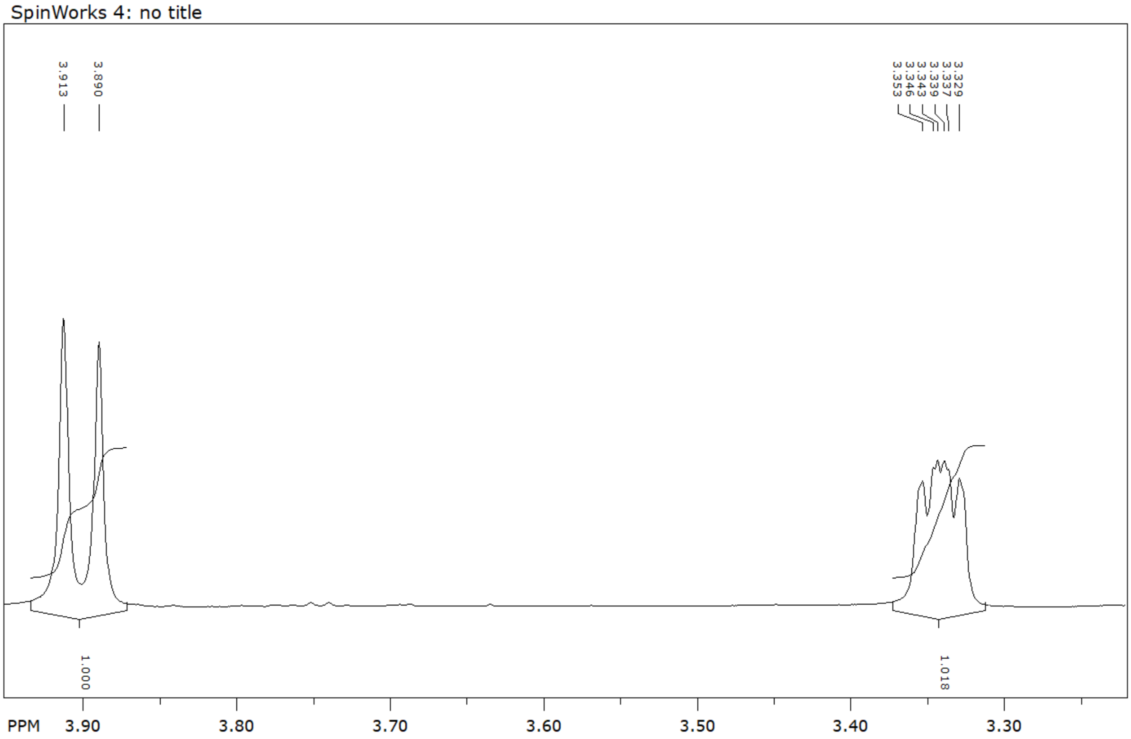

file: D:\mfl-2801\10\fid   expt: <zg30>
transmitter freq.: 600.263707 MHz
time domain size: 65536 points
width: 12335.53 Hz = 20.5502 ppm = 0.188225 Hz/pt
number of scans: 16

freq. of 0 ppm: 600.260000 MHz
processed size: 32768 complex points
LB: 0.300   GF: 0.0000

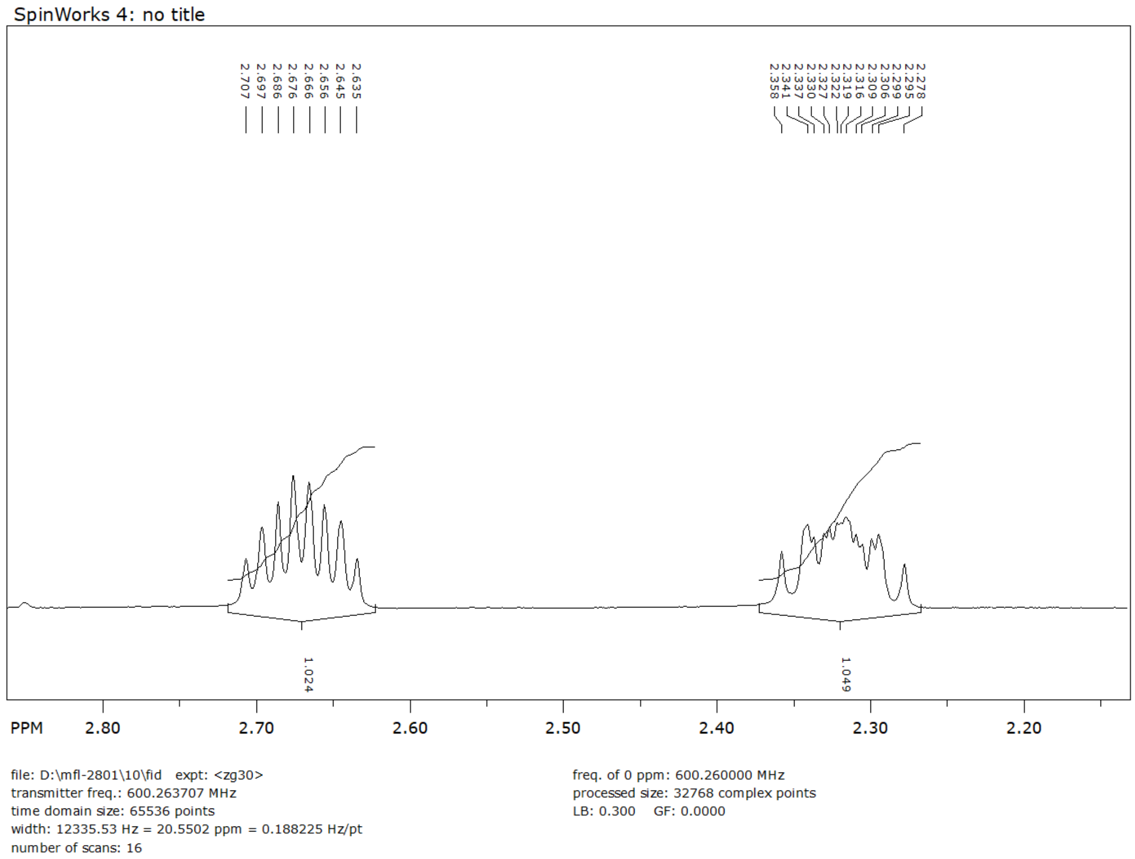

Figure S35: ^{31}P NMR Spectrum for *trans*-**16c** in $CDCl_3$

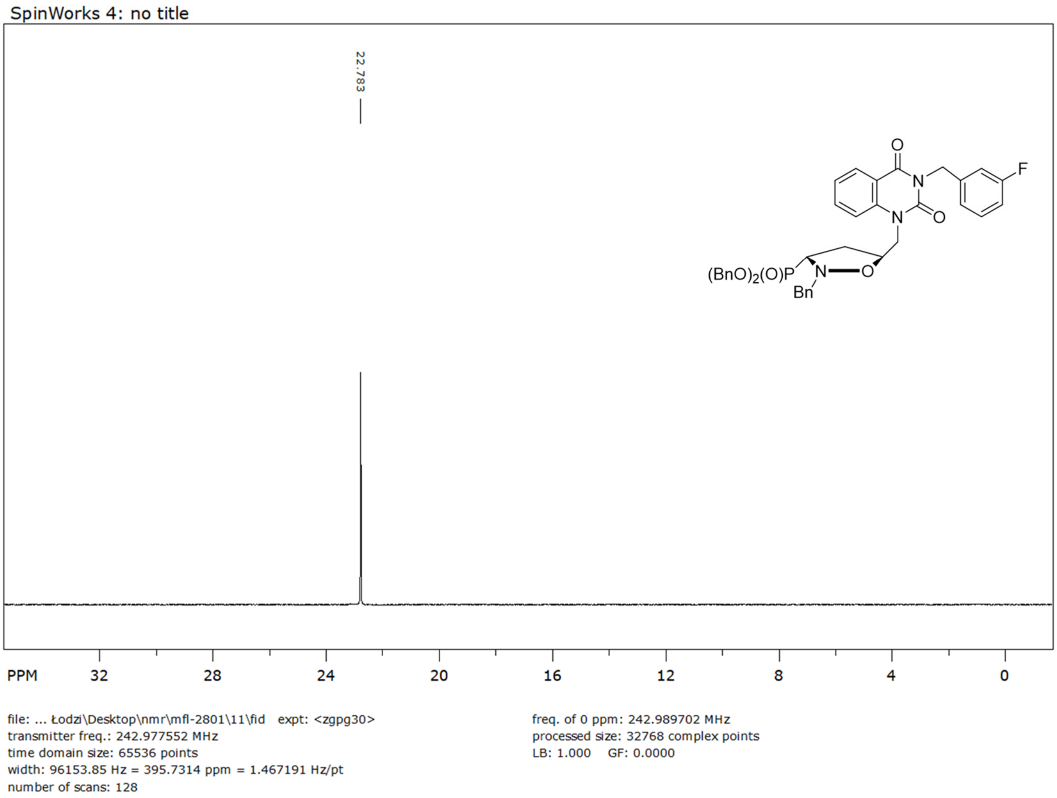

Figure S36: ^{13}C NMR Spectrum for *trans*-**16c** in $CDCl_3$ and expanded spectral regions

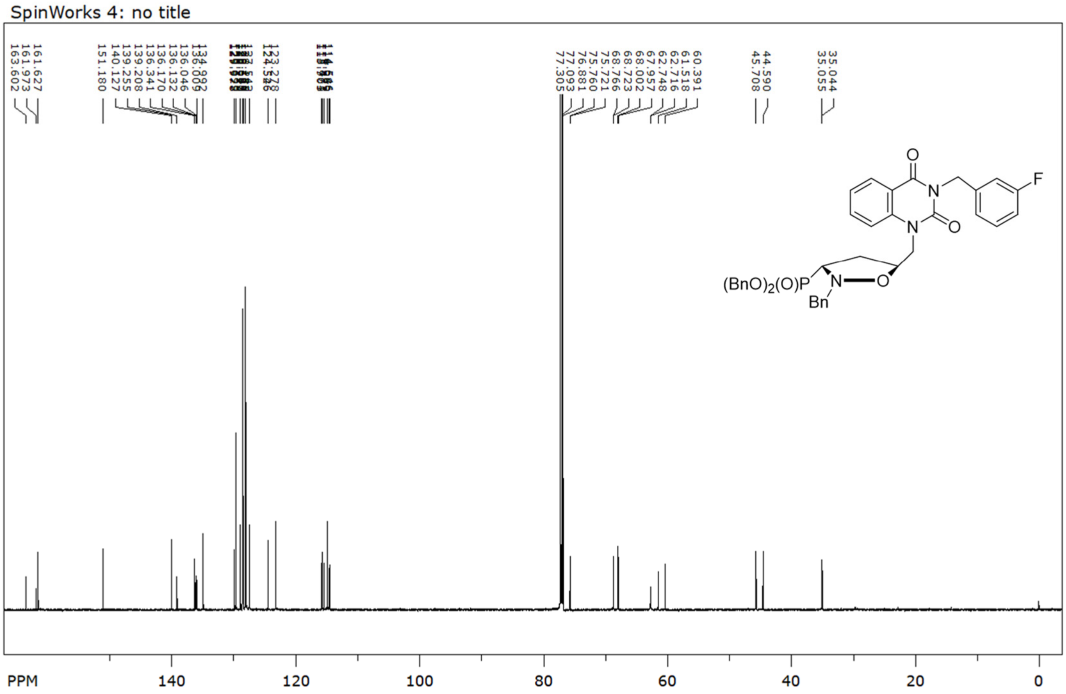

file: ... Łodzi\Desktop\nmr\mfl-2807\10\fid expt: <zgpg30>
transmitter freq.: 150.950591 MHz
time domain size: 65536 points
width: 36057.69 Hz = 238.8708 ppm = 0.550197 Hz/pt
number of scans: 3072

freq. of 0 ppm: 150.935497 MHz
processed size: 32768 complex points
LB: 1.000 GF: 0.0000

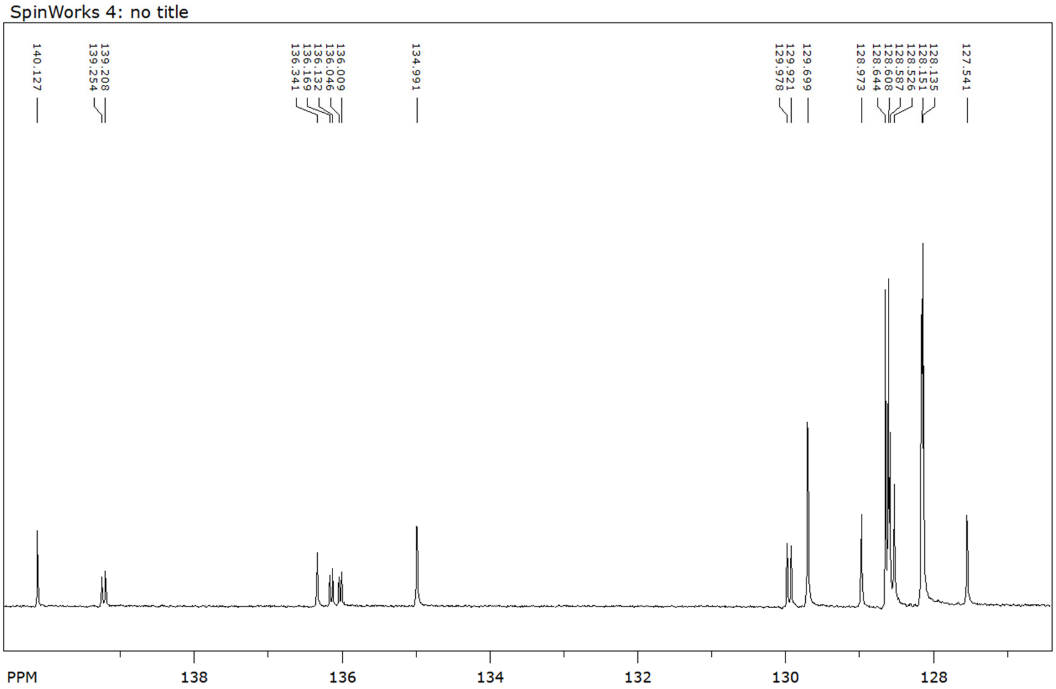

file: ...fl widma\widma 13C\mfl-2807\10\fid expt: <zgpg30>
transmitter freq.: 150.950591 MHz
time domain size: 65536 points
width: 36057.69 Hz = 238.8708 ppm = 0.550197 Hz/pt
number of scans: 3072

freq. of 0 ppm: 150.935497 MHz
processed size: 32768 complex points
LB: 1.000 GF: 0.0000

SpinWorks 4: no title

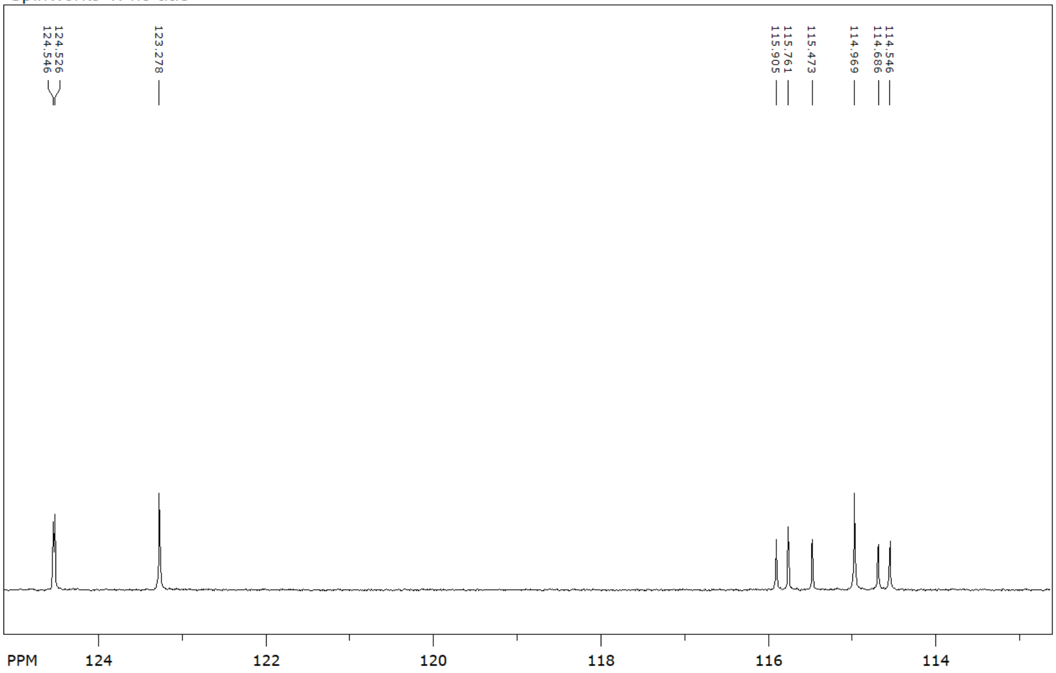

file: ... Łodzi\Desktop\nmr\mfl-2807\10\fid expt: <zgpg30>
transmitter freq.: 150.950591 MHz
time domain size: 65536 points
width: 36057.69 Hz = 238.8708 ppm = 0.550197 Hz/pt
number of scans: 3072

freq. of 0 ppm: 150.935497 MHz
processed size: 32768 complex points
LB: 1.000 GF: 0.0000

SpinWorks 4: no title

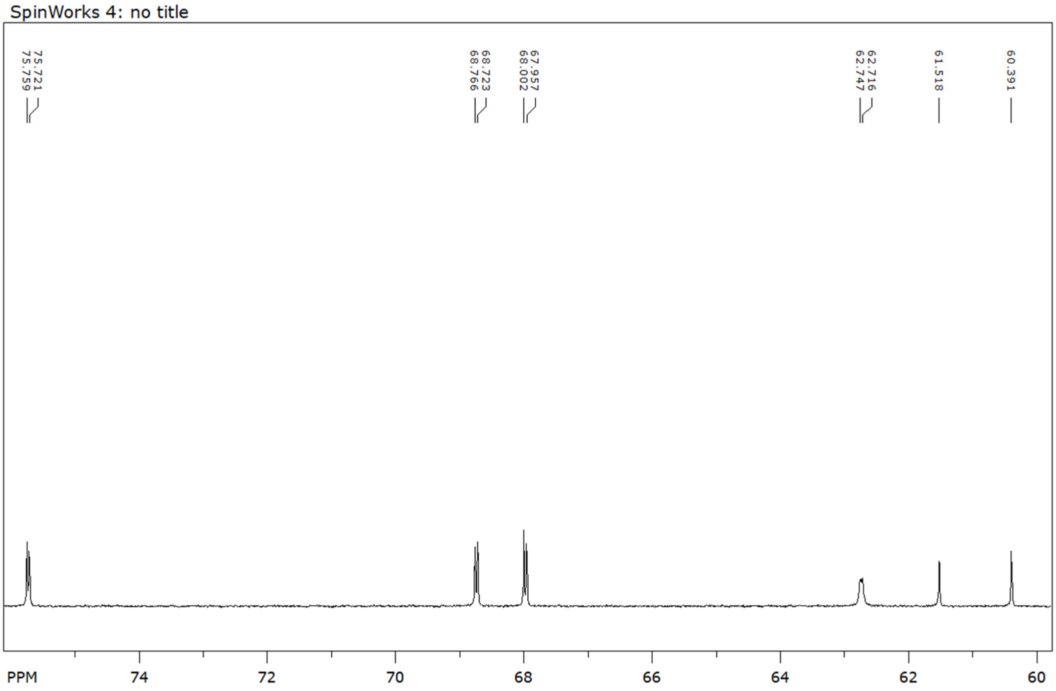

file: ...fl widma\widma 13C\mfl-2807\10\fid expt: <zgpg30>
transmitter freq.: 150.950591 MHz
time domain size: 65536 points
width: 36057.69 Hz = 238.8708 ppm = 0.550197 Hz/pt
number of scans: 3072

freq. of 0 ppm: 150.935497 MHz
processed size: 32768 complex points
LB: 1.000 GF: 0.0000

Figure S37: HPLC chromatogram for *trans*-**16c**

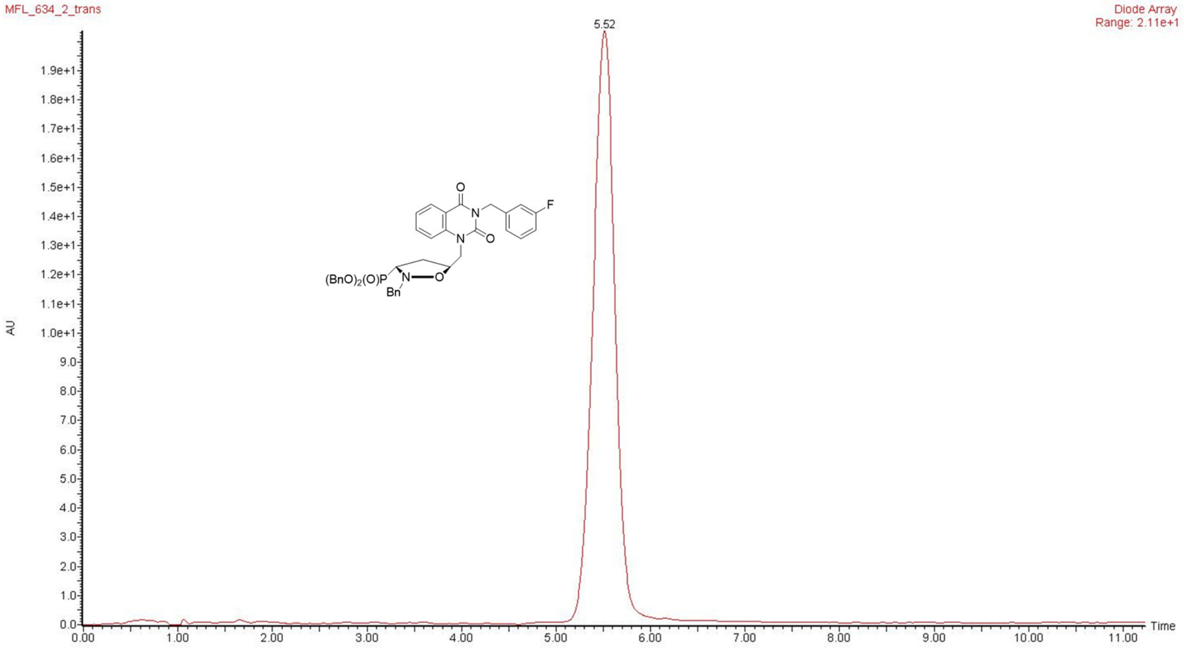

Figure S38: ^{1}H NMR Spectrum for mixture of *cis*-**16d**/*trans*-**16d** (96:4) in $CDCl_3$ and expanded spectral regions

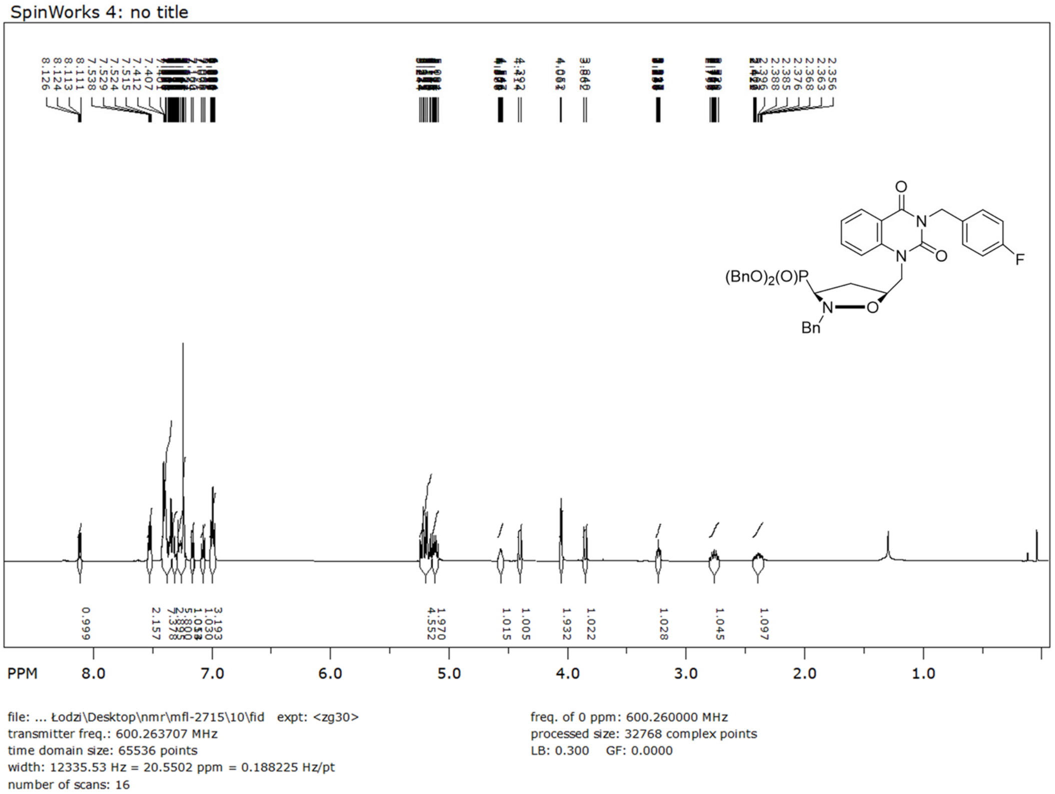

SpinWorks 4: no title

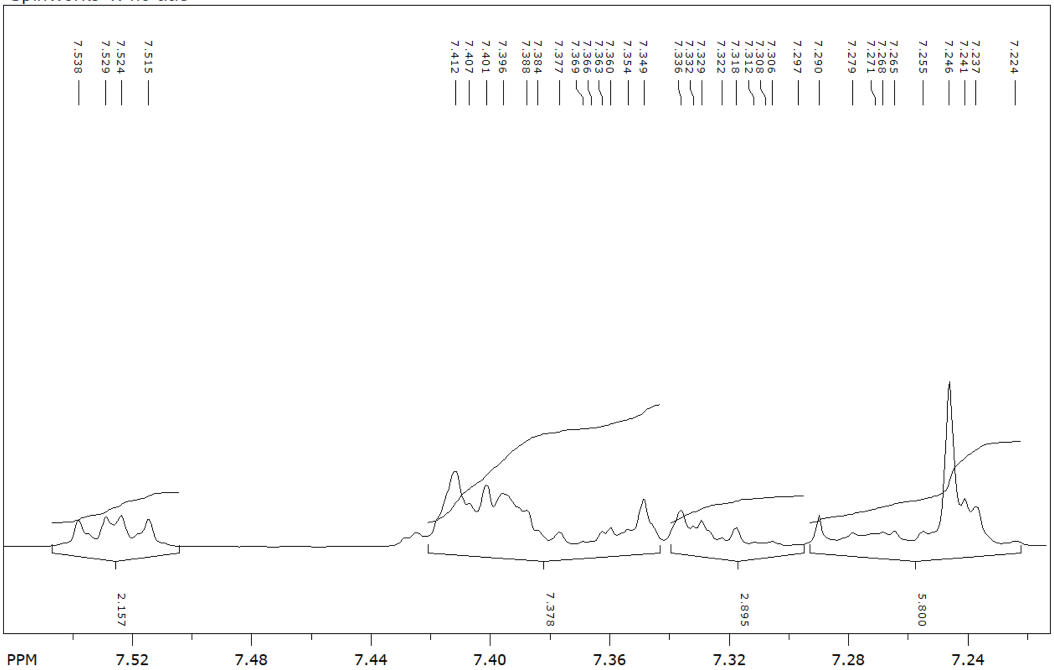

file: ... Łodzi\Desktop\nmr\mfl-2715\10\fid expt: <zg30>
transmitter freq.: 600.263707 MHz
time domain size: 65536 points
width: 12335.53 Hz = 20.5502 ppm = 0.188225 Hz/pt
number of scans: 16

freq. of 0 ppm: 600.260000 MHz
processed size: 32768 complex points
LB: 0.300 GF: 0.0000

SpinWorks 4: no title

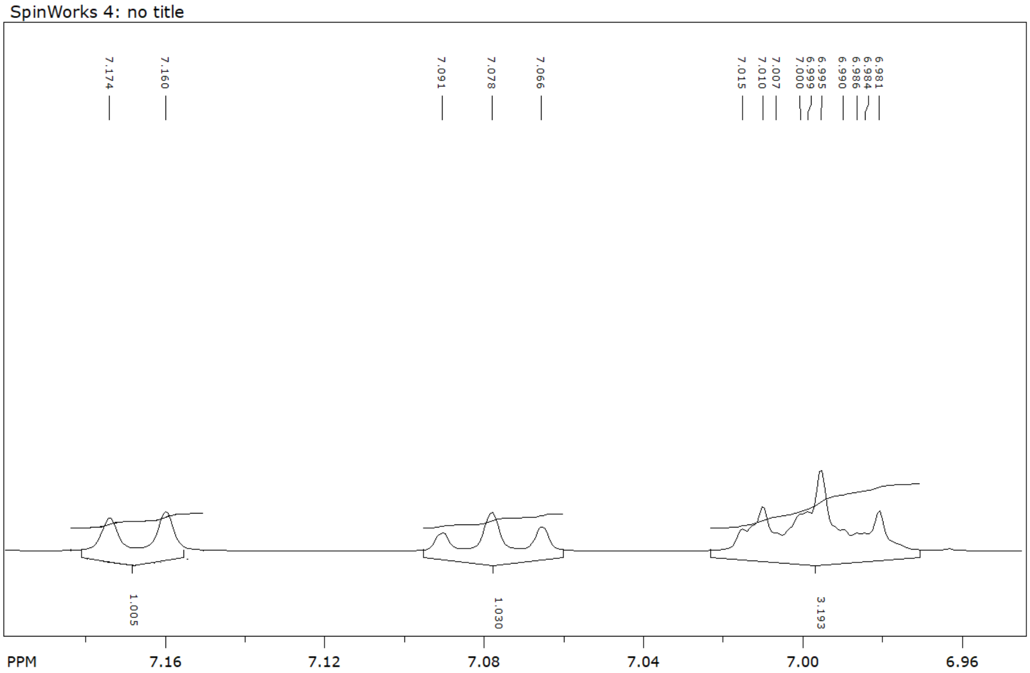

file: ... Łodzi\Desktop\nmr\mfl-2715\10\fid expt: <zg30>
transmitter freq.: 600.263707 MHz
time domain size: 65536 points
width: 12335.53 Hz = 20.5502 ppm = 0.188225 Hz/pt
number of scans: 16

freq. of 0 ppm: 600.260000 MHz
processed size: 32768 complex points
LB: 0.300 GF: 0.0000

SpinWorks 4: no title

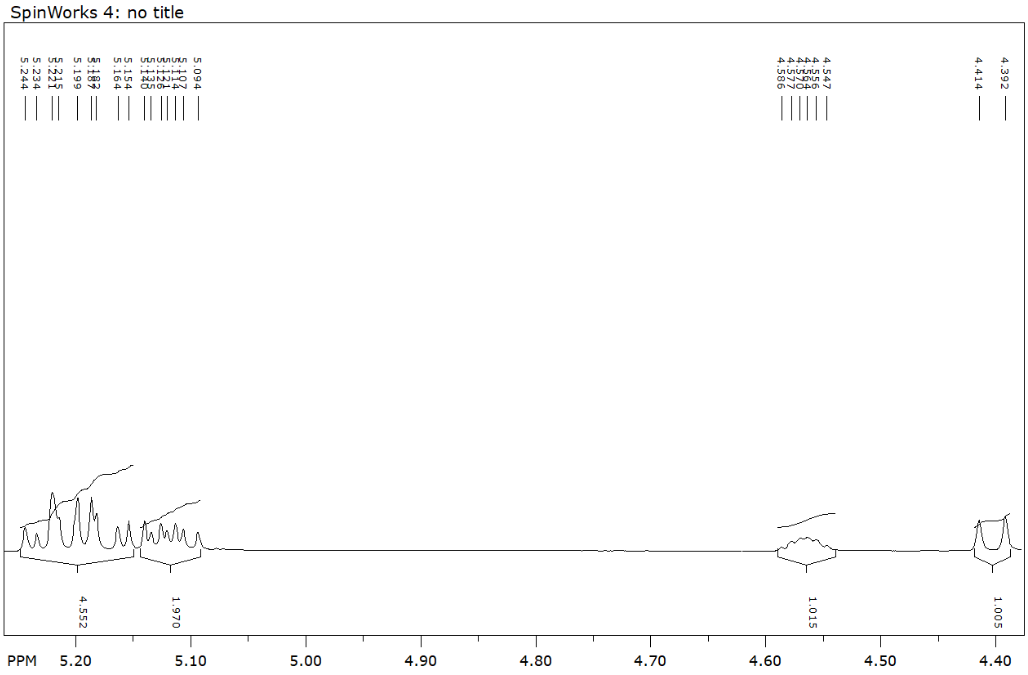

file: ... Łodzi\Desktop\nmr\mfl-2715\10\fid   expt: <zg30>
transmitter freq.: 600.263707 MHz
time domain size: 65536 points
width: 12335.53 Hz = 20.5502 ppm = 0.188225 Hz/pt
number of scans: 16

freq. of 0 ppm: 600.260000 MHz
processed size: 32768 complex points
LB: 0.300   GF: 0.0000

SpinWorks 4: no title

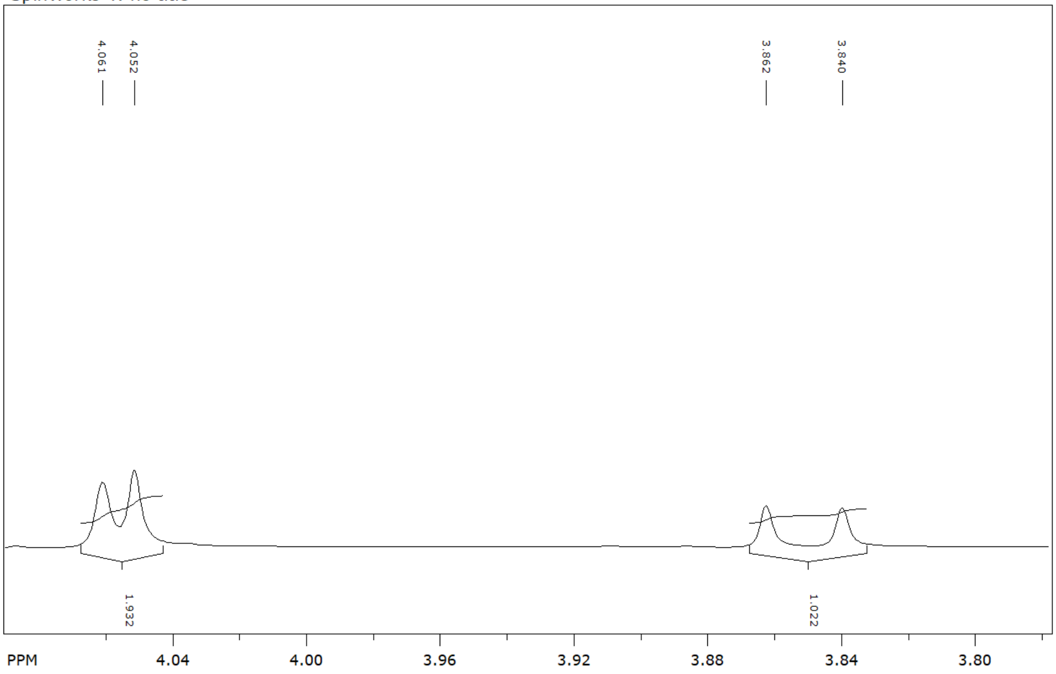

file: ... Łodzi\Desktop\nmr\mfl-2715\10\fid   expt: <zg30>
transmitter freq.: 600.263707 MHz
time domain size: 65536 points
width: 12335.53 Hz = 20.5502 ppm = 0.188225 Hz/pt
number of scans: 16

freq. of 0 ppm: 600.260000 MHz
processed size: 32768 complex points
LB: 0.300   GF: 0.0000

SpinWorks 4: no title

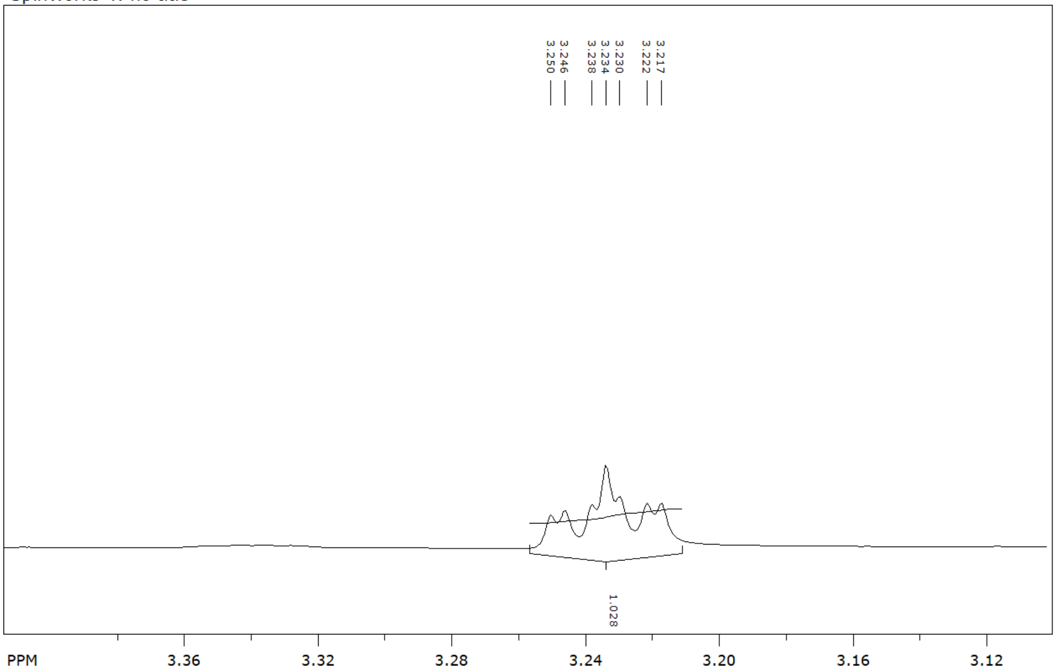

file: ... Łodzi\Desktop\nmr\mfl-2715\10\fid expt: <zg30>
transmitter freq.: 600.263707 MHz
time domain size: 65536 points
width: 12335.53 Hz = 20.5502 ppm = 0.188225 Hz/pt
number of scans: 16

freq. of 0 ppm: 600.260000 MHz
processed size: 32768 complex points
LB: 0.300 GF: 0.0000

SpinWorks 4: no title

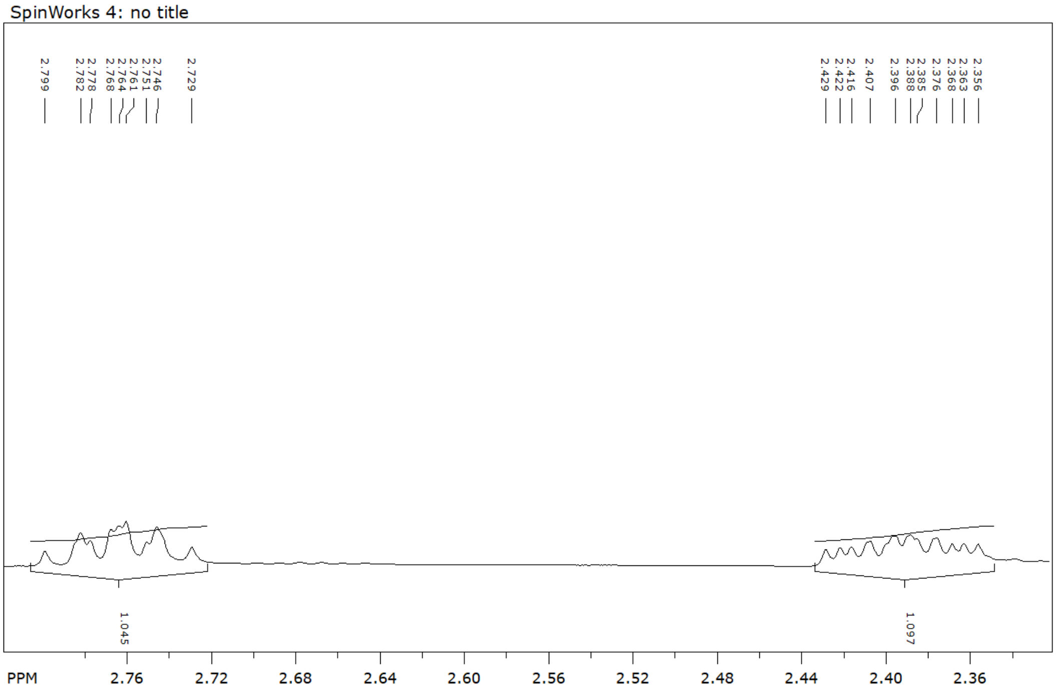

file: ... Łodzi\Desktop\nmr\mfl-2715\10\fid expt: <zg30>
transmitter freq.: 600.263707 MHz
time domain size: 65536 points
width: 12335.53 Hz = 20.5502 ppm = 0.188225 Hz/pt
number of scans: 16

freq. of 0 ppm: 600.260000 MHz
processed size: 32768 complex points
LB: 0.300 GF: 0.0000

Figure S39: ^{31}P NMR Spectrum for mixture of *cis*-**16d**/*trans*-**16d** (96:4) in $CDCl_3$

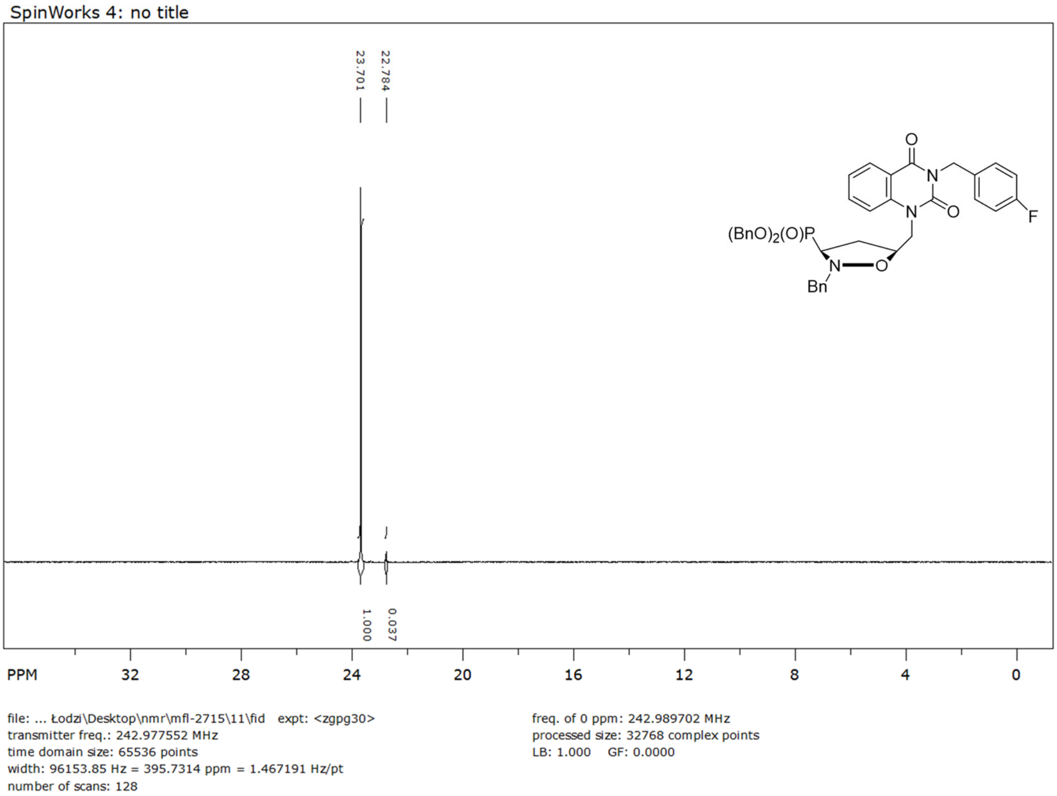

Figure S40: ^{13}C NMR Spectrum for mixture of *cis*-**16d**/*trans*-**16d** (96:4) in $CDCl_3$ and expanded spectral regions

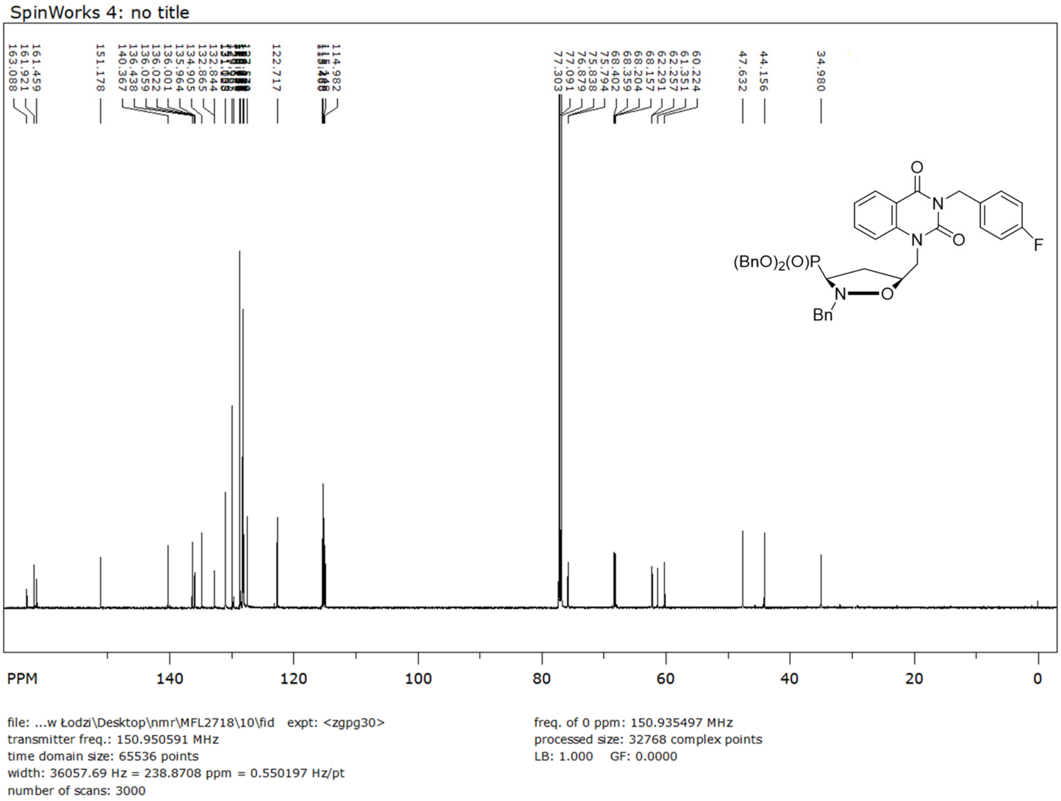

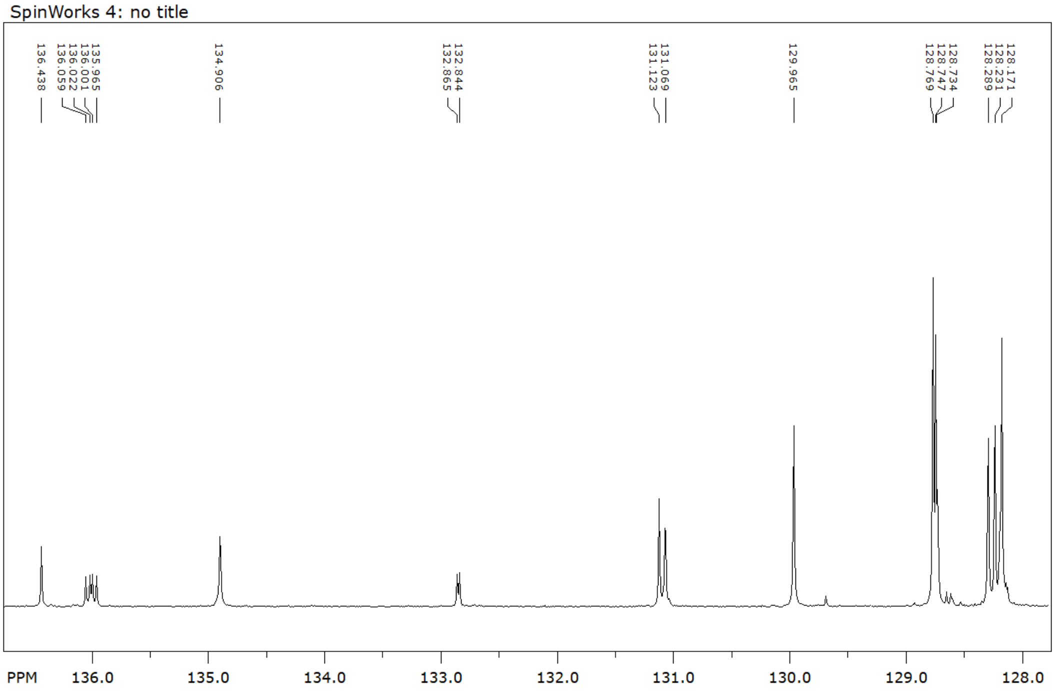

file: ...mfl widma\widma 13C\MFL2718\10\fid expt: <zgpg30>
transmitter freq.: 150.950591 MHz
time domain size: 65536 points
width: 36057.69 Hz = 238.8708 ppm = 0.550197 Hz/pt
number of scans: 3000

freq. of 0 ppm: 150.935497 MHz
processed size: 32768 complex points
LB: 1.000 GF: 0.0000

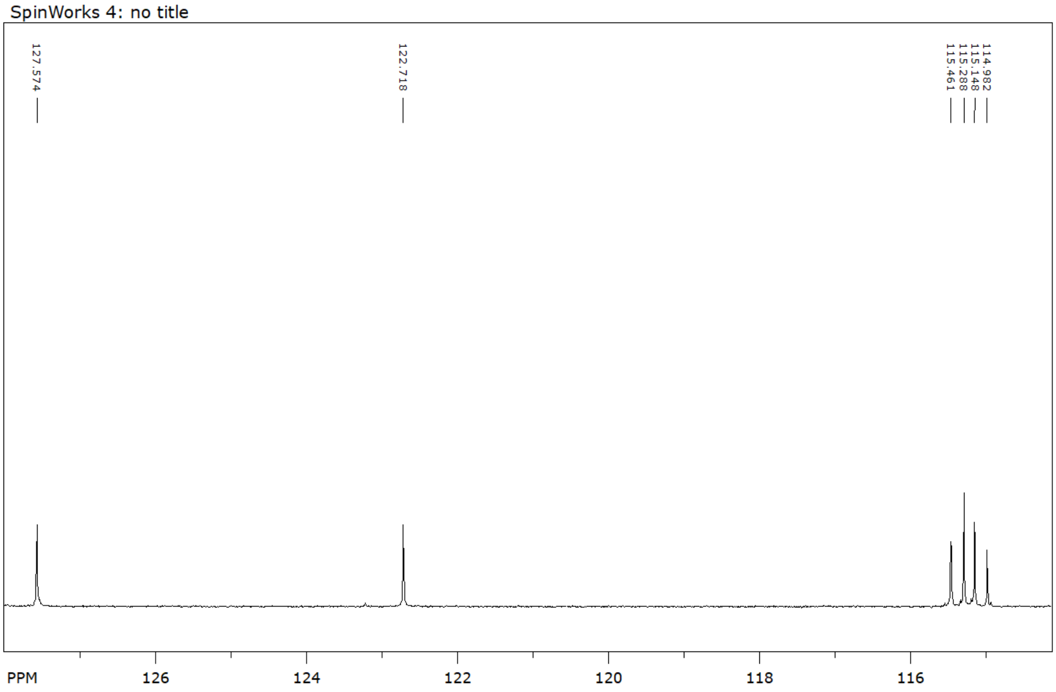

file: ...mfl widma\widma 13C\MFL2718\10\fid expt: <zgpg30>
transmitter freq.: 150.950591 MHz
time domain size: 65536 points
width: 36057.69 Hz = 238.8708 ppm = 0.550197 Hz/pt
number of scans: 3000

freq. of 0 ppm: 150.935497 MHz
processed size: 32768 complex points
LB: 1.000 GF: 0.0000

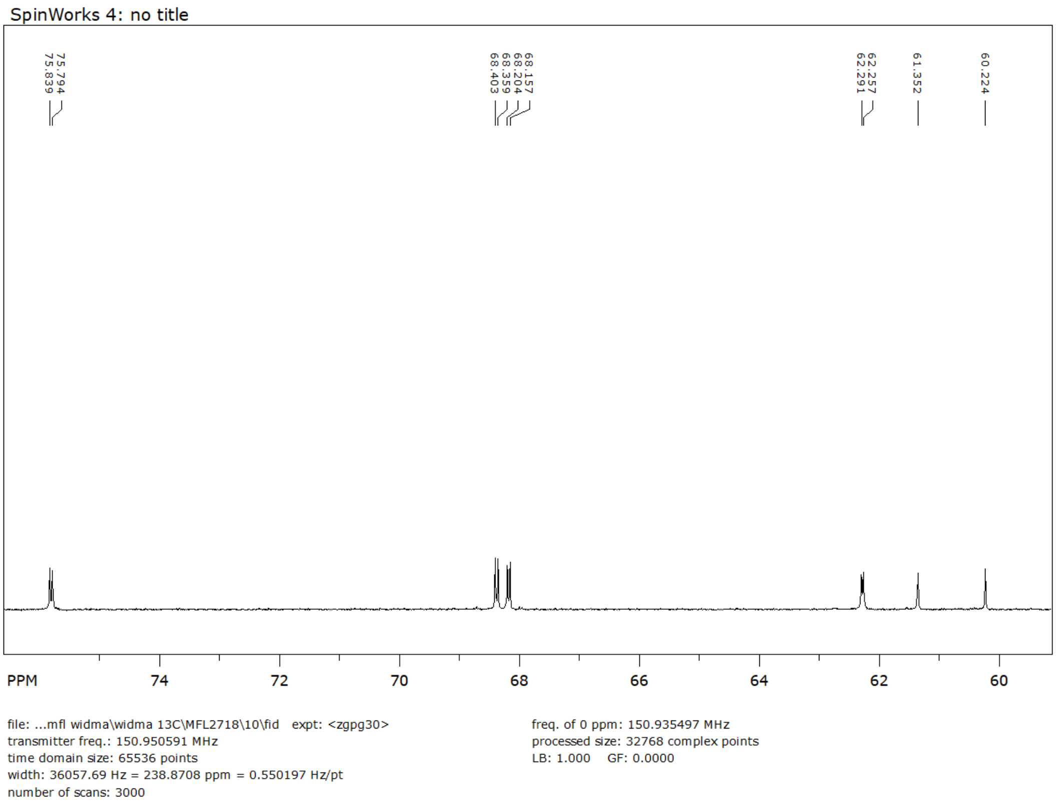

Figure S41: HPLC chromatogram for mixture of *cis*-**16d**/*trans*-**16d** (96:4)

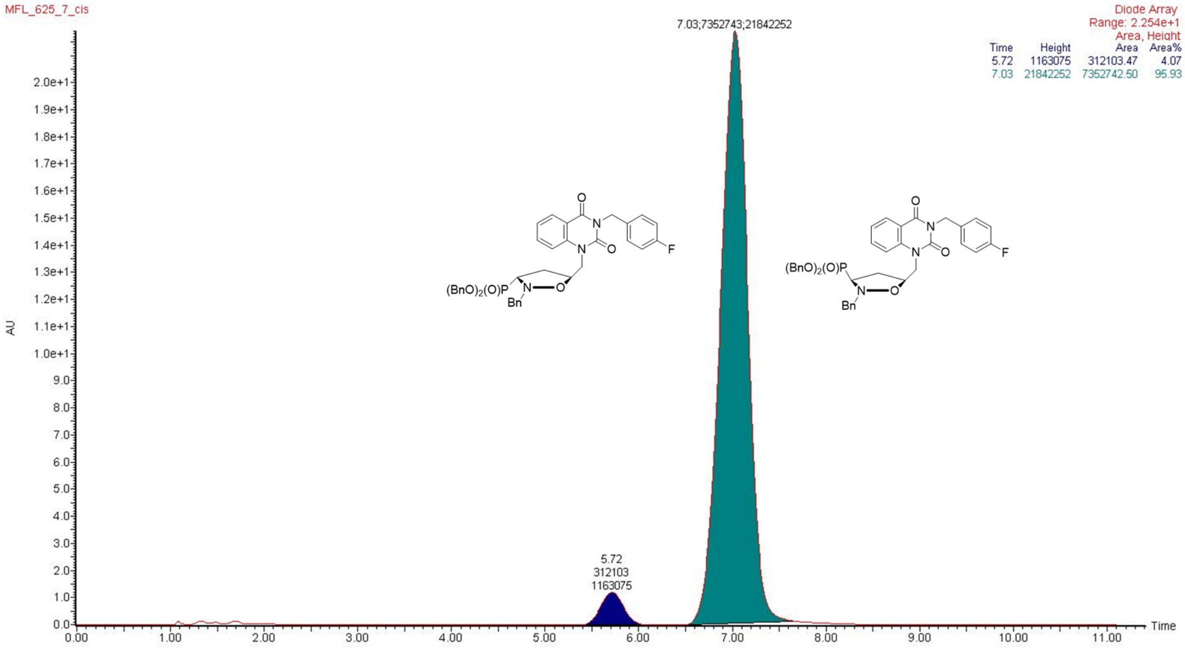

Figure S42: ^{1}H NMR Spectrum for *trans*-**16d** in CDCl3 and expanded spectral regions

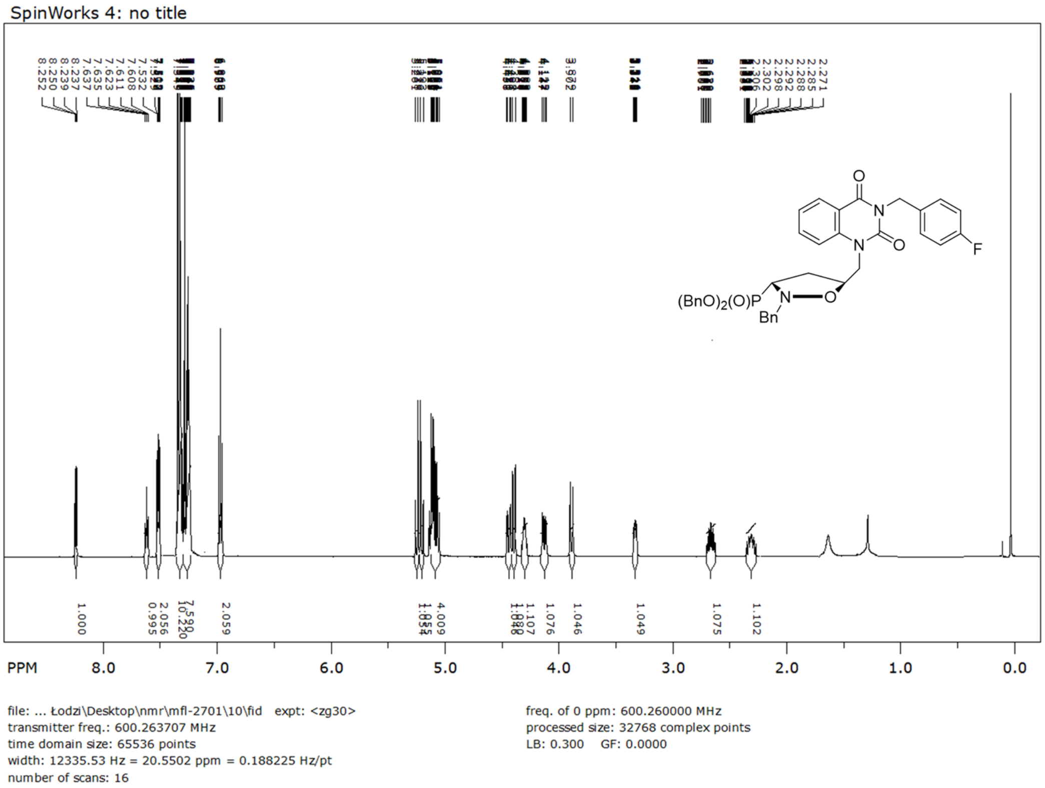

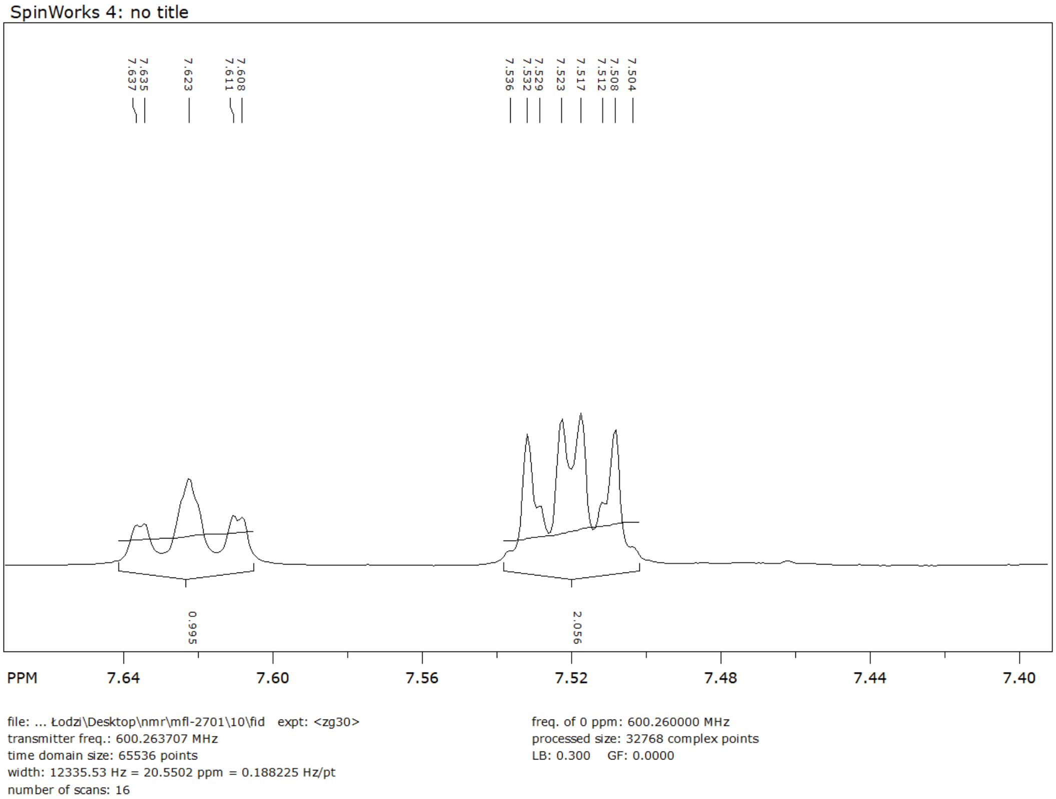

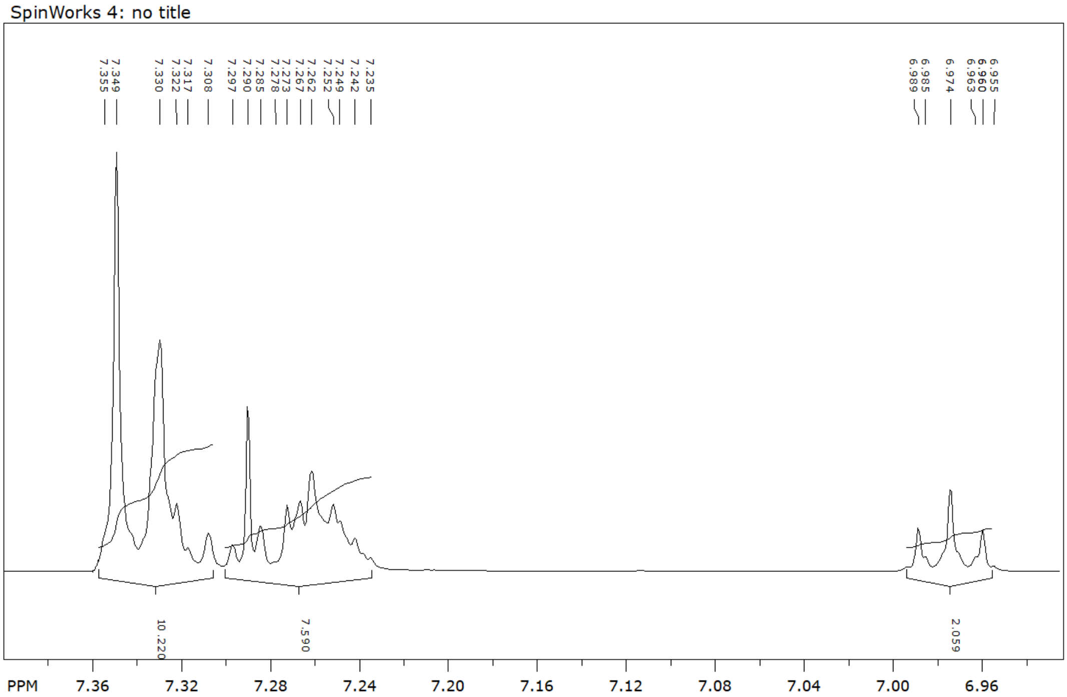

file: ... Łodzi\Desktop\nmr\mfl-2701\10\fid expt: <zg30>
transmitter freq.: 600.263707 MHz
time domain size: 65536 points
width: 12335.53 Hz = 20.5502 ppm = 0.188225 Hz/pt
number of scans: 16

freq. of 0 ppm: 600.260000 MHz
processed size: 32768 complex points
LB: 0.300 GF: 0.0000

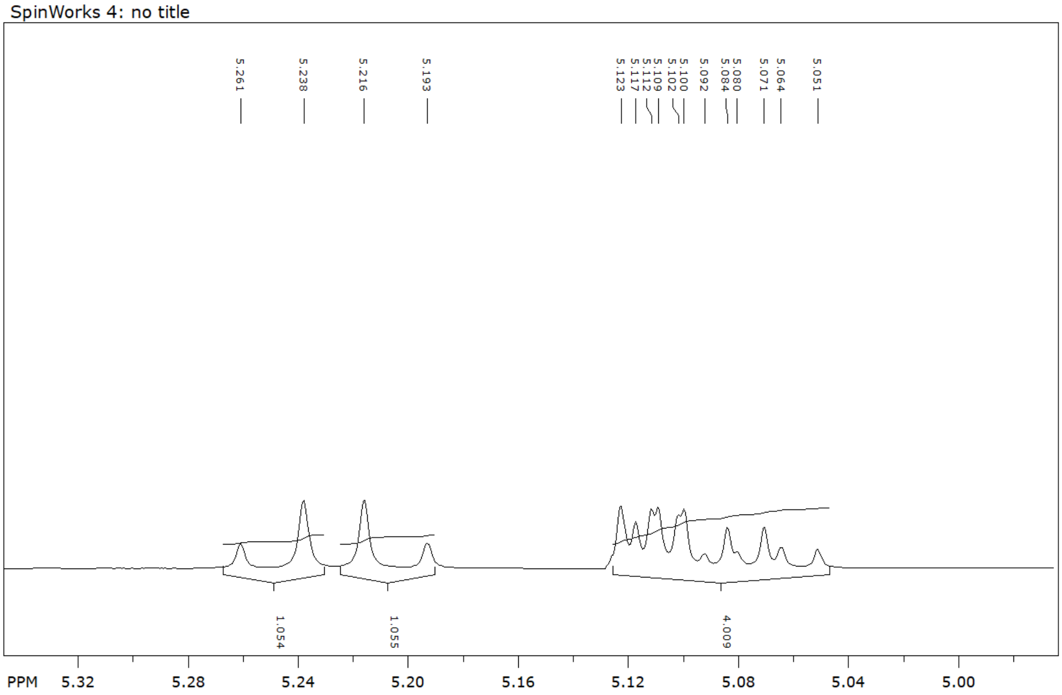

file: ... Łodzi\Desktop\nmr\mfl-2701\10\fid expt: <zg30>
transmitter freq.: 600.263707 MHz
time domain size: 65536 points
width: 12335.53 Hz = 20.5502 ppm = 0.188225 Hz/pt
number of scans: 16

freq. of 0 ppm: 600.260000 MHz
processed size: 32768 complex points
LB: 0.300 GF: 0.0000

SpinWorks 4: no title

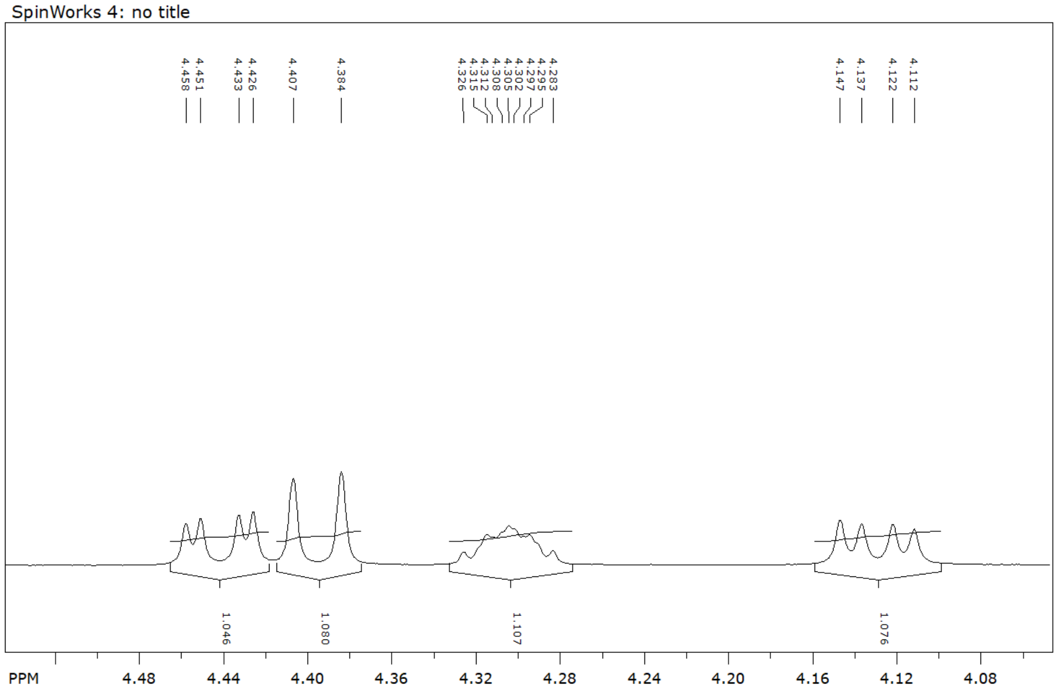

file: ... Łodzi\Desktop\nmr\mfl-2701\10\fid expt: <zg30>
transmitter freq.: 600.263707 MHz
time domain size: 65536 points
width: 12335.53 Hz = 20.5502 ppm = 0.188225 Hz/pt
number of scans: 16

freq. of 0 ppm: 600.260000 MHz
processed size: 32768 complex points
LB: 0.300 GF: 0.0000

SpinWorks 4: no title

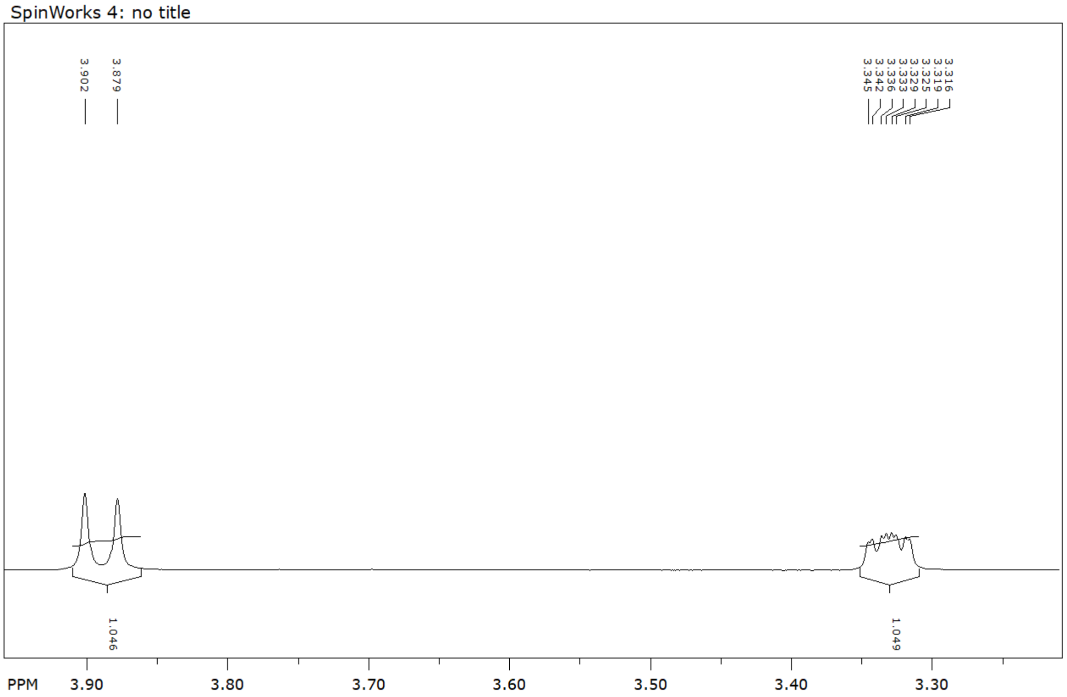

file: ... Łodzi\Desktop\nmr\mfl-2701\10\fid expt: <zg30>
transmitter freq.: 600.263707 MHz
time domain size: 65536 points
width: 12335.53 Hz = 20.5502 ppm = 0.188225 Hz/pt
number of scans: 16

freq. of 0 ppm: 600.260000 MHz
processed size: 32768 complex points
LB: 0.300 GF: 0.0000

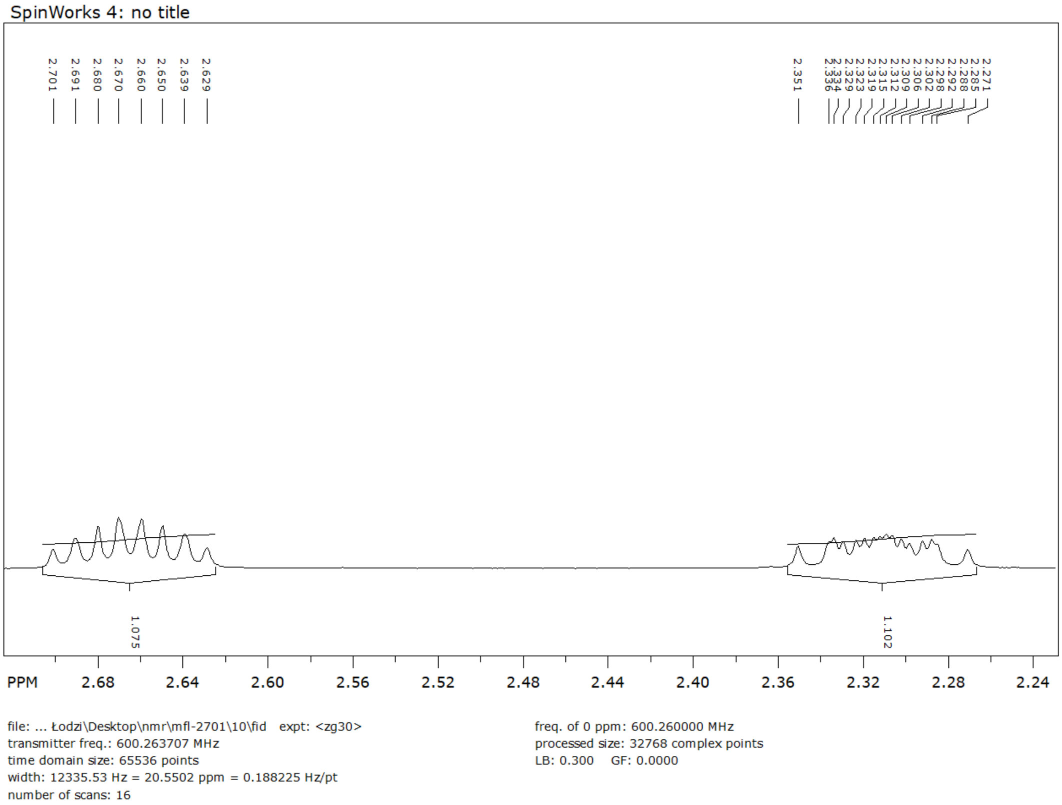

Figure S43: ^{31}P NMR Spectrum for *trans*-**16d** in $CDCl_3$

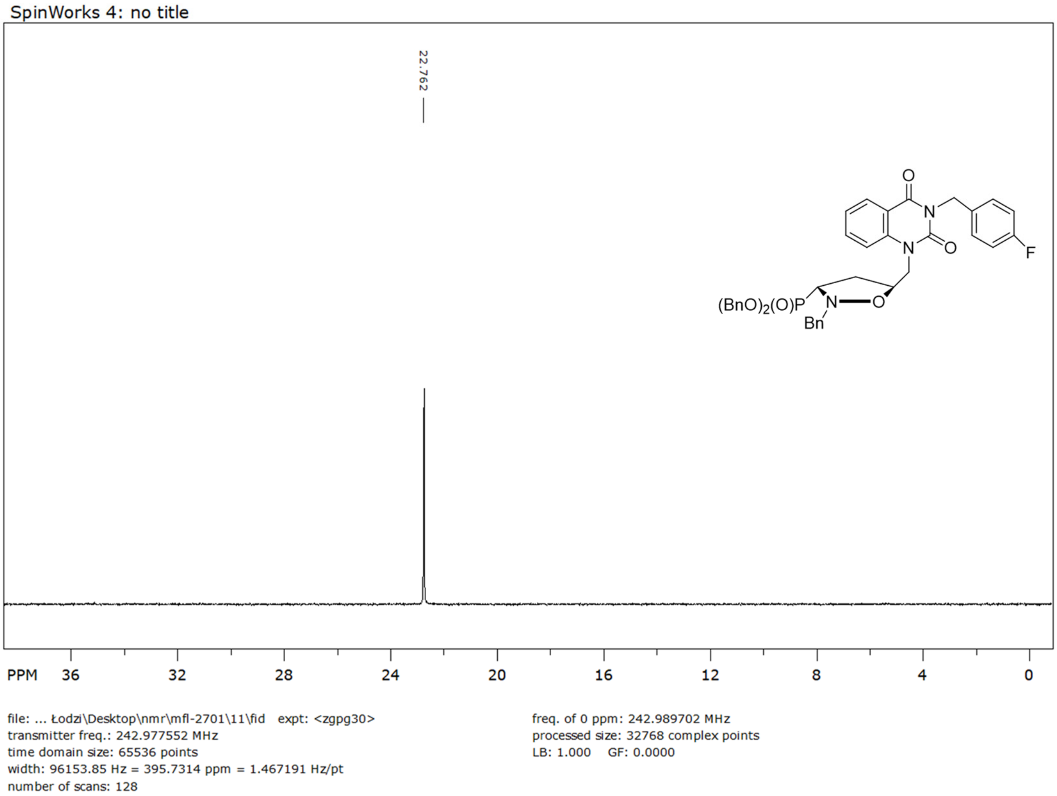

Figure S44: ^{13}C NMR Spectrum for *trans*-**16d** in $CDCl_3$ and expanded spectral regions

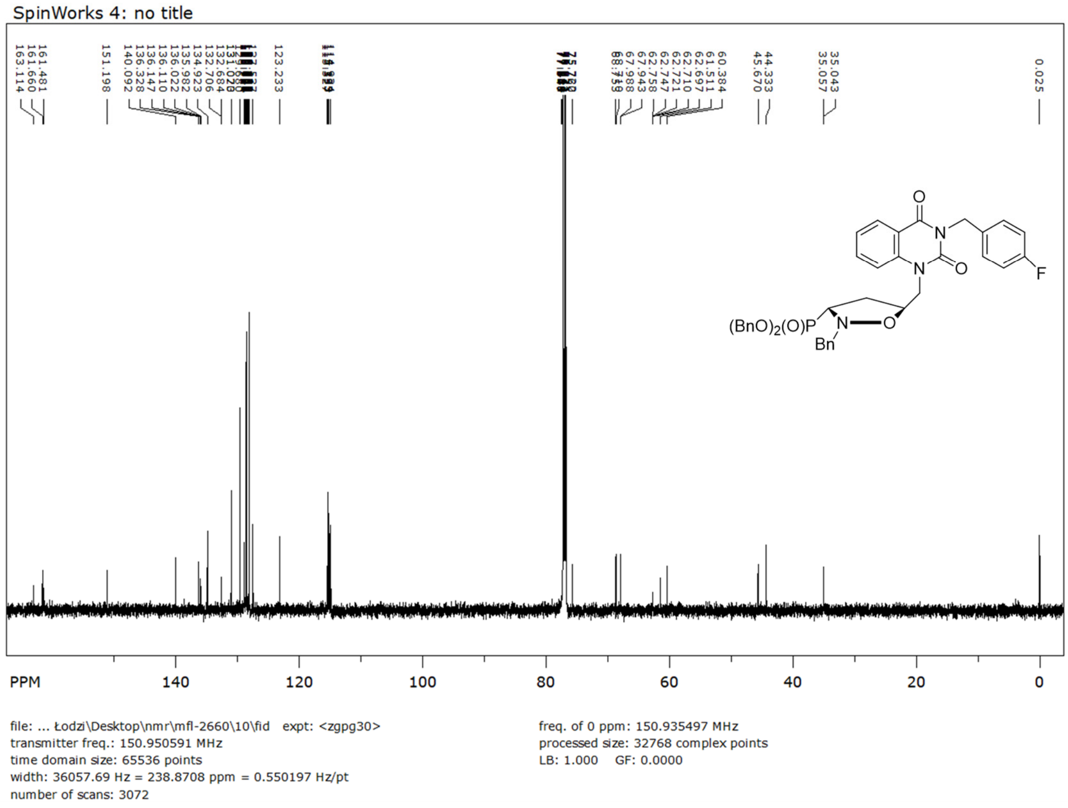

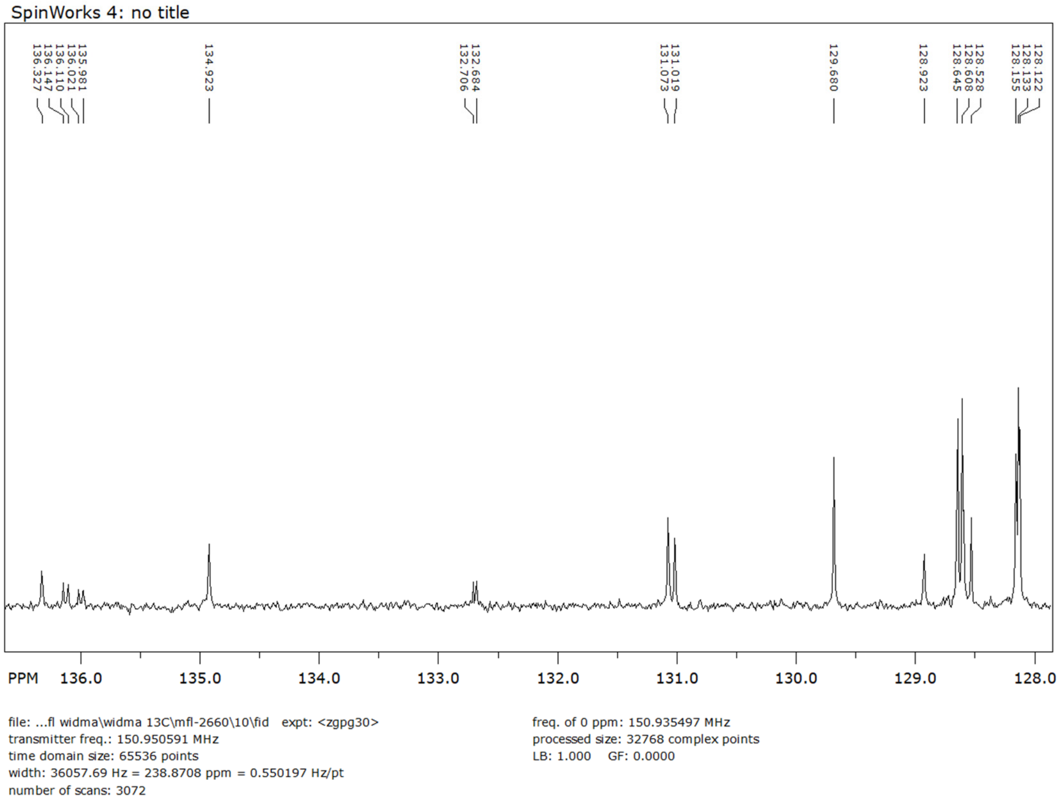

SpinWorks 4: no title

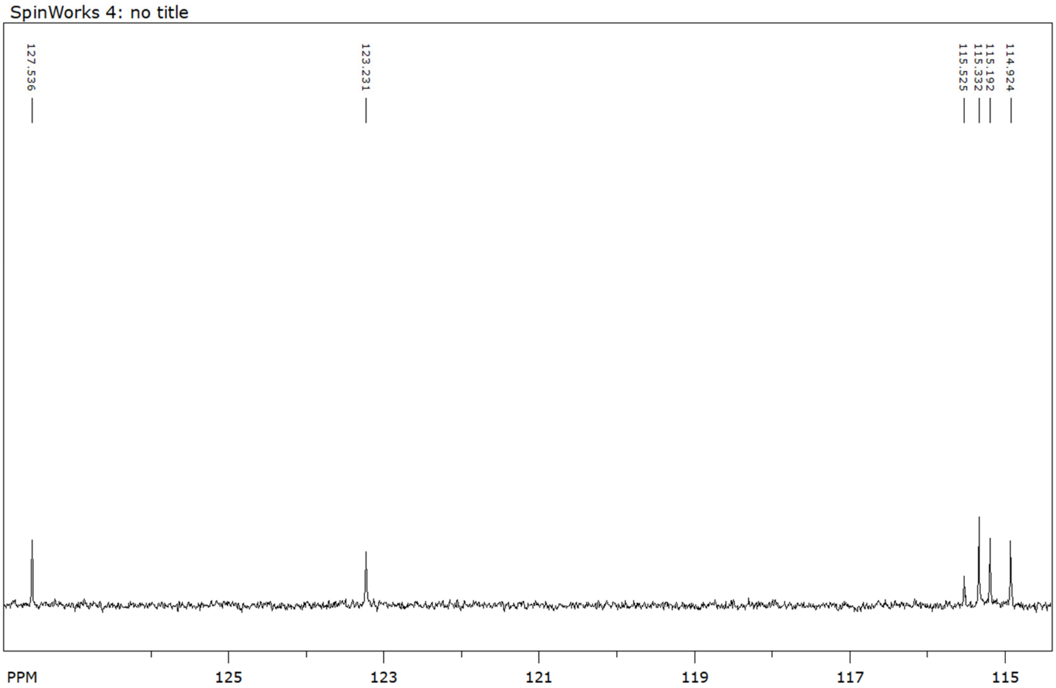

file: ...fl widma\widma 13C\mfl-2660\10\fid expt: <zgpg30>
transmitter freq.: 150.950591 MHz
time domain size: 65536 points
width: 36057.69 Hz = 238.8708 ppm = 0.550197 Hz/pt
number of scans: 3072

freq. of 0 ppm: 150.935497 MHz
processed size: 32768 complex points
LB: 1.000 GF: 0.0000

SpinWorks 4: no title

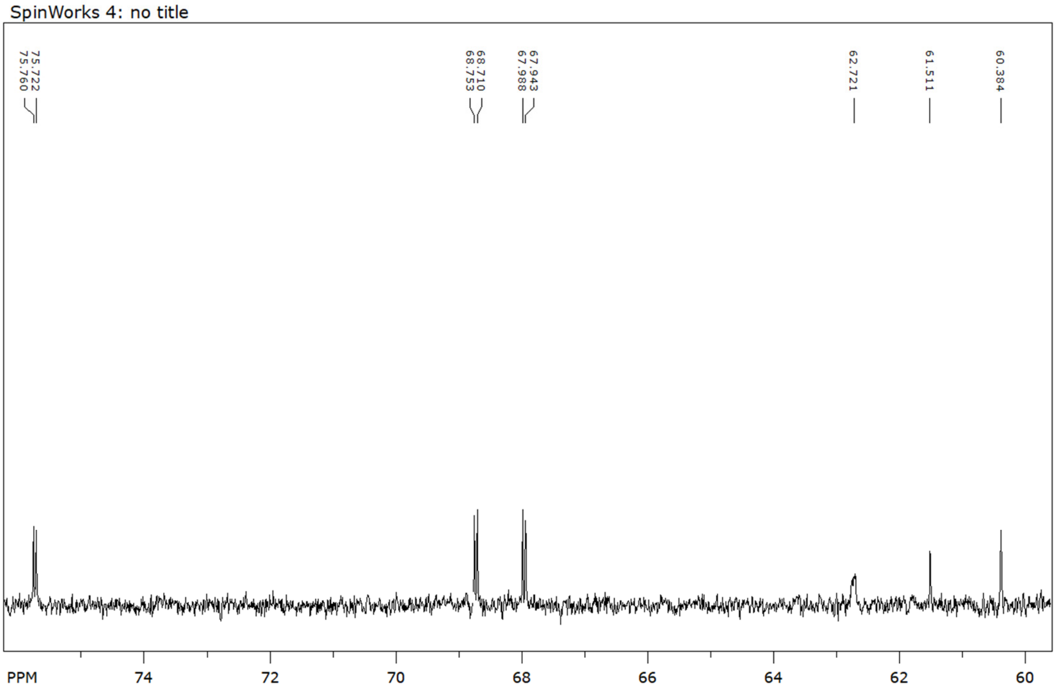

file: ...fl widma\widma 13C\mfl-2660\10\fid expt: <zgpg30>
transmitter freq.: 150.950591 MHz
time domain size: 65536 points
width: 36057.69 Hz = 238.8708 ppm = 0.550197 Hz/pt
number of scans: 3072

freq. of 0 ppm: 150.935497 MHz
processed size: 32768 complex points
LB: 1.000 GF: 0.0000

Figure S45: HPLC chromatogram for *trans*-**16d**

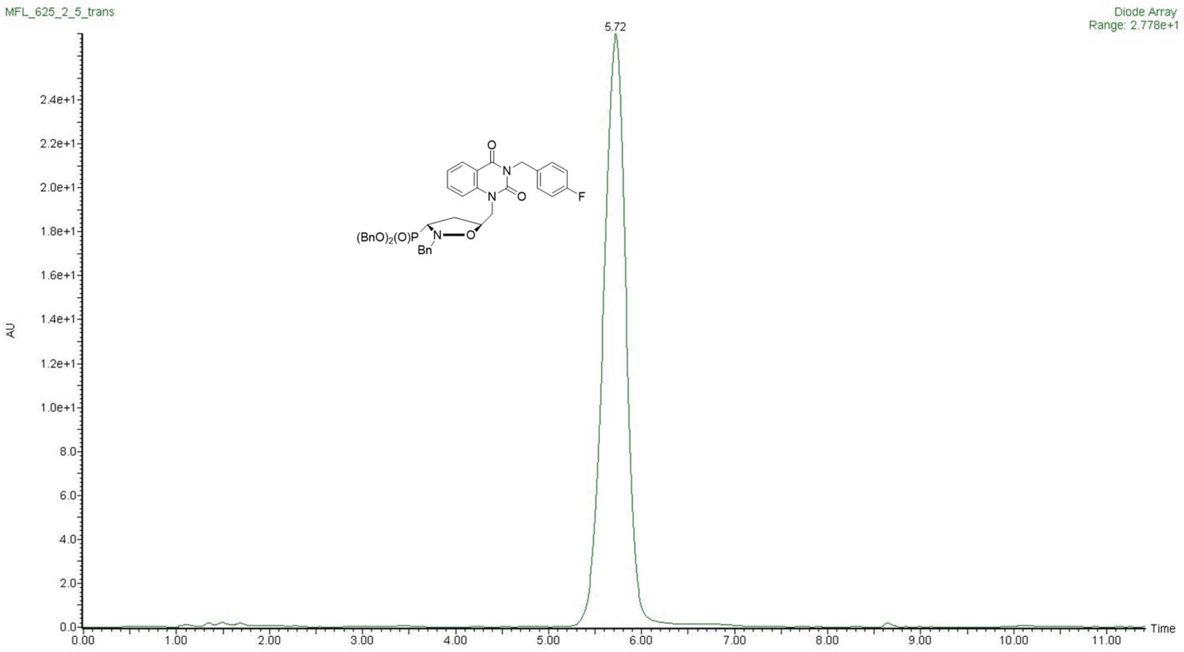

Figure S46: ^{1}H NMR Spectrum for mixture of *cis*-**16e**/*trans*-**16e** (70:30) in $CDCl_3$ and expanded spectral regions

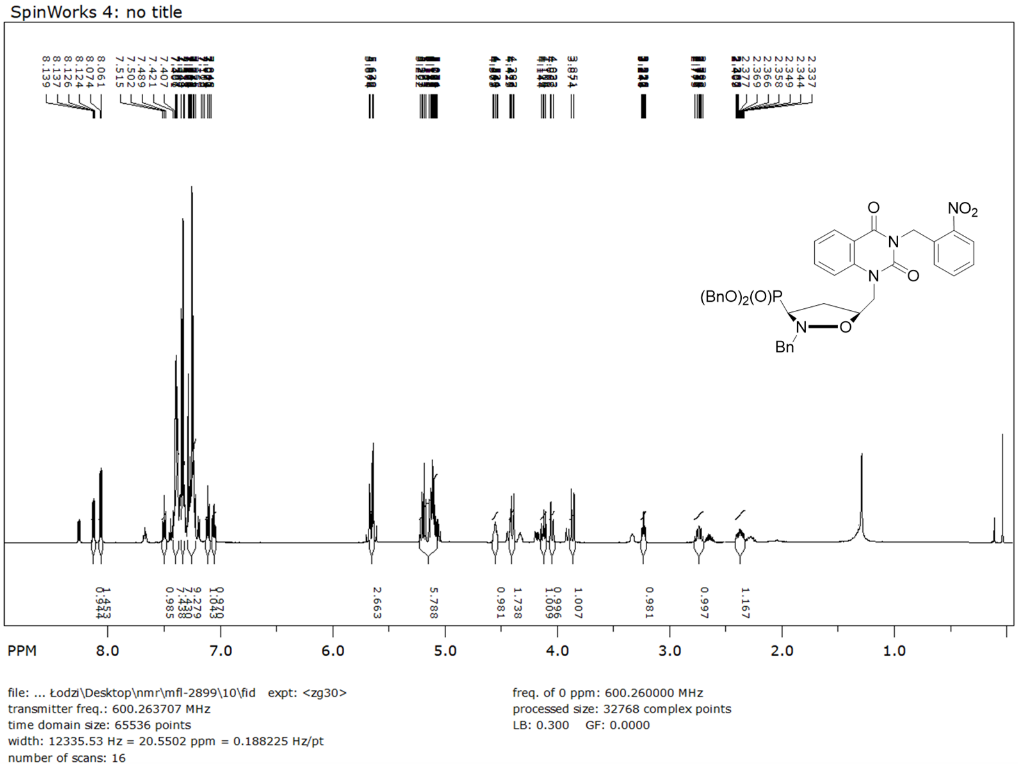

SpinWorks 4: no title

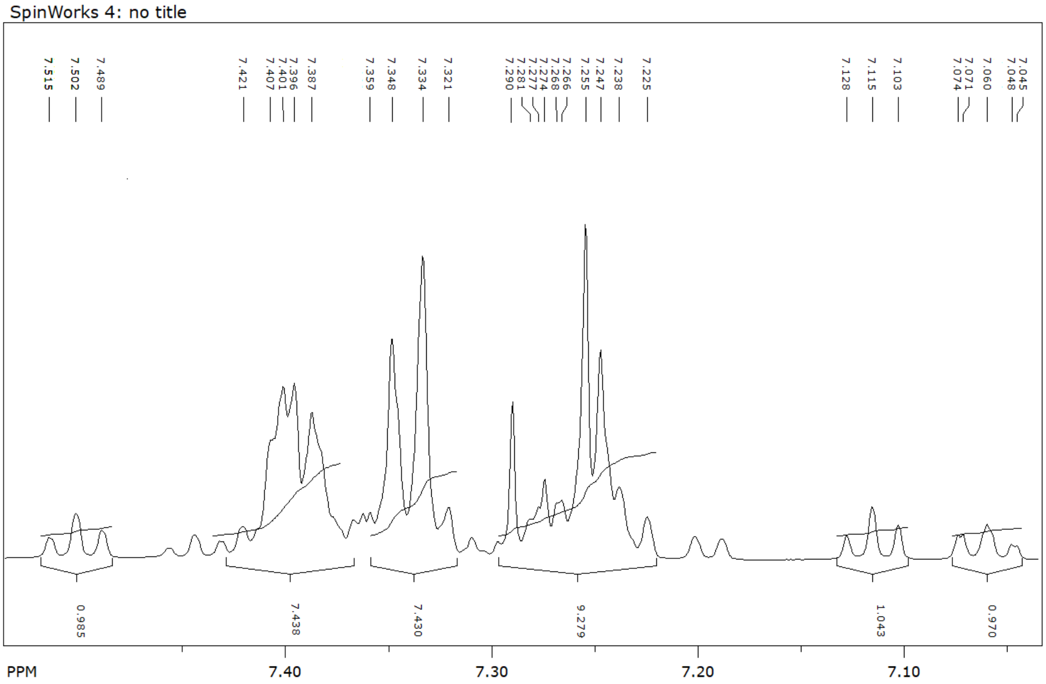

file: ... Łodzi\Desktop\nmr\mfl-2899\10\fid expt: <zg30>
transmitter freq.: 600.263707 MHz
time domain size: 65536 points
width: 12335.53 Hz = 20.5502 ppm = 0.188225 Hz/pt
number of scans: 16

freq. of 0 ppm: 600.260000 MHz
processed size: 32768 complex points
LB: 0.300 GF: 0.0000

SpinWorks 4: no title

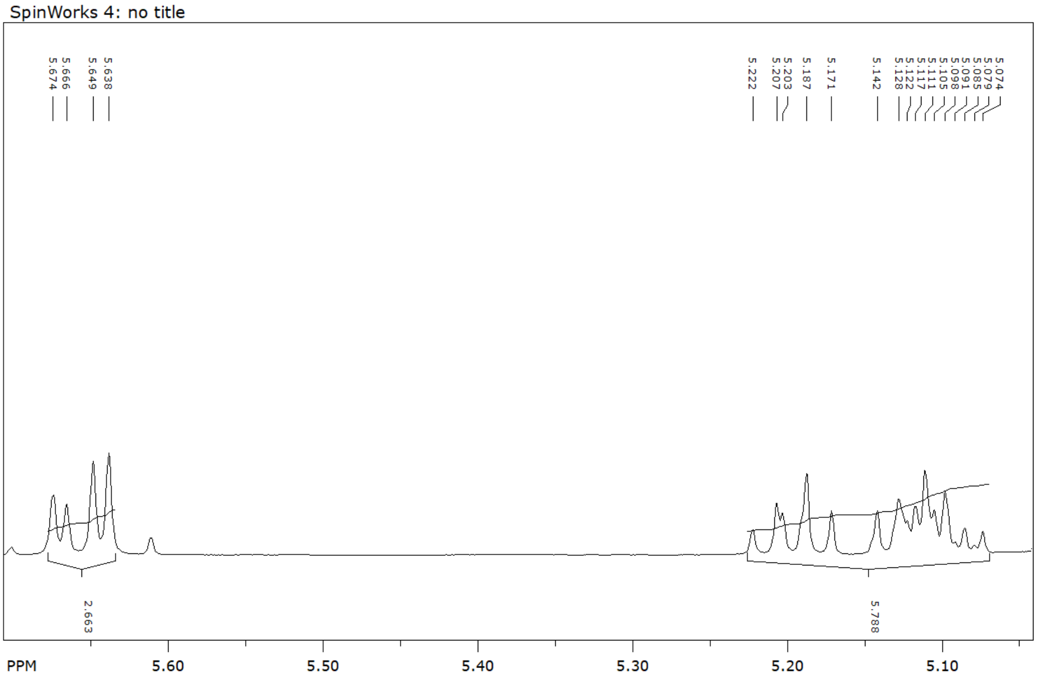

file: ... Łodzi\Desktop\nmr\mfl-2899\10\fid expt: <zg30>
transmitter freq.: 600.263707 MHz
time domain size: 65536 points
width: 12335.53 Hz = 20.5502 ppm = 0.188225 Hz/pt
number of scans: 16

freq. of 0 ppm: 600.260000 MHz
processed size: 32768 complex points
LB: 0.300 GF: 0.0000

SpinWorks 4: no title

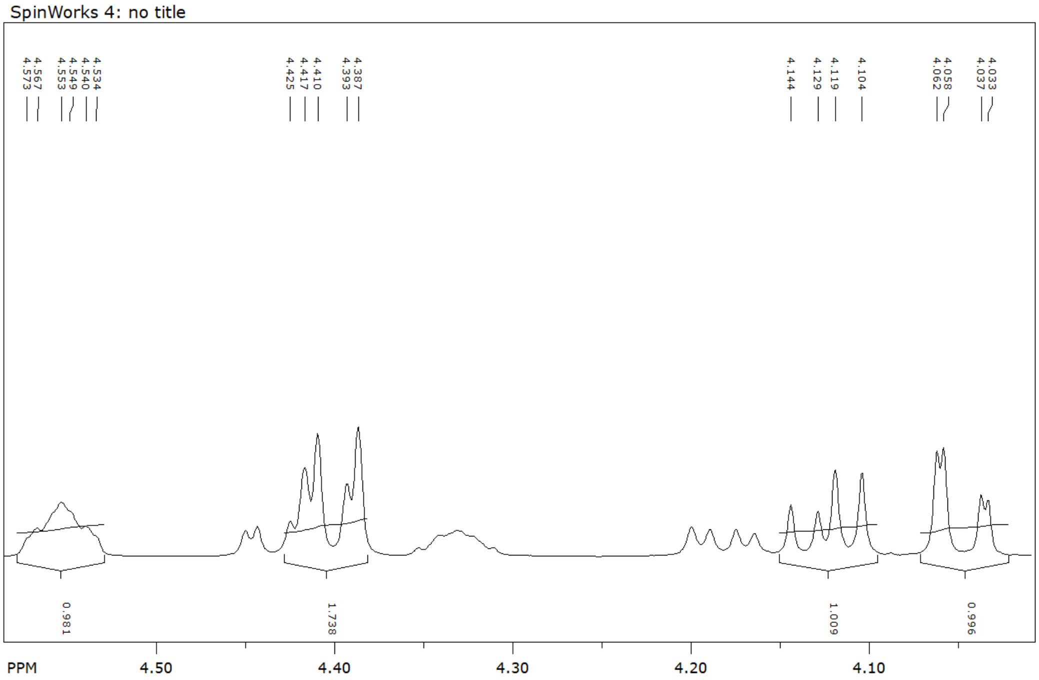

file: ... Łodzi\Desktop\nmr\mfl-2899\10\fid expt: <zg30>
transmitter freq.: 600.263707 MHz
time domain size: 65536 points
width: 12335.53 Hz = 20.5502 ppm = 0.188225 Hz/pt
number of scans: 16

freq. of 0 ppm: 600.260000 MHz
processed size: 32768 complex points
LB: 0.300 GF: 0.0000

SpinWorks 4: no title

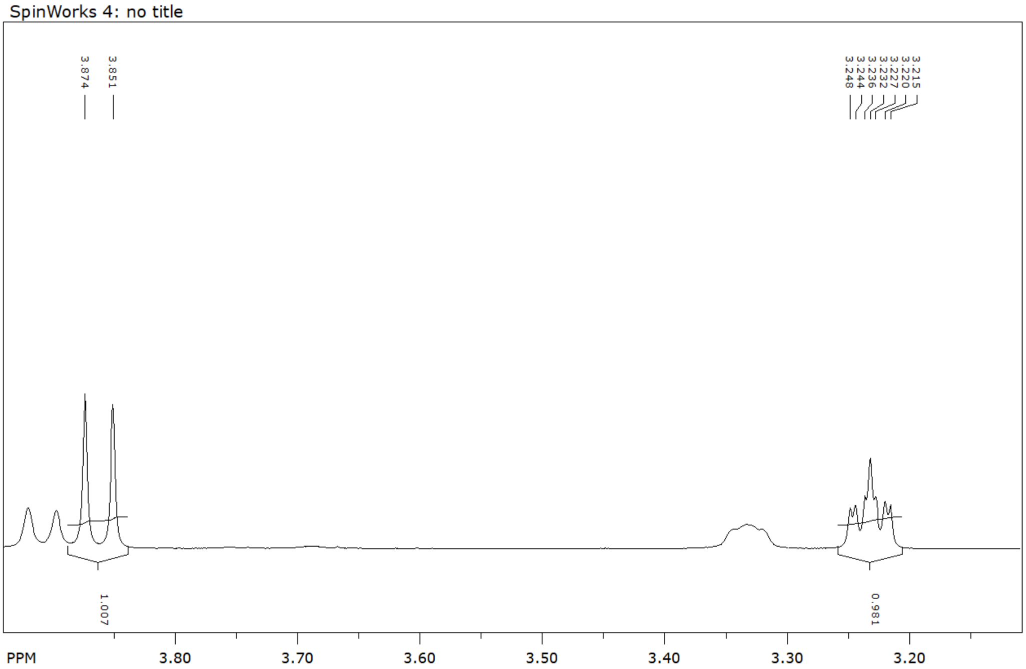

file: ... Łodzi\Desktop\nmr\mfl-2899\10\fid expt: <zg30>
transmitter freq.: 600.263707 MHz
time domain size: 65536 points
width: 12335.53 Hz = 20.5502 ppm = 0.188225 Hz/pt
number of scans: 16

freq. of 0 ppm: 600.260000 MHz
processed size: 32768 complex points
LB: 0.300 GF: 0.0000

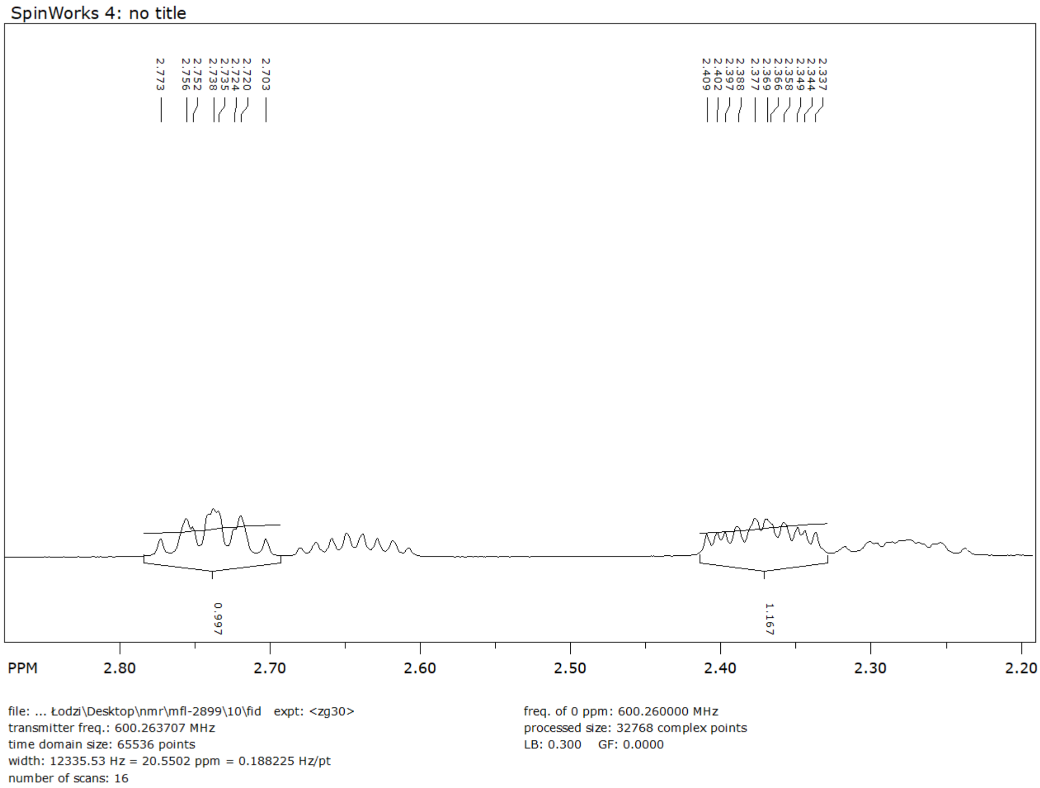

Figure S47: ^{31}P NMR Spectrum for mixture of *cis*-**16e**/*trans*-**16e** (70:30) in $CDCl_3$

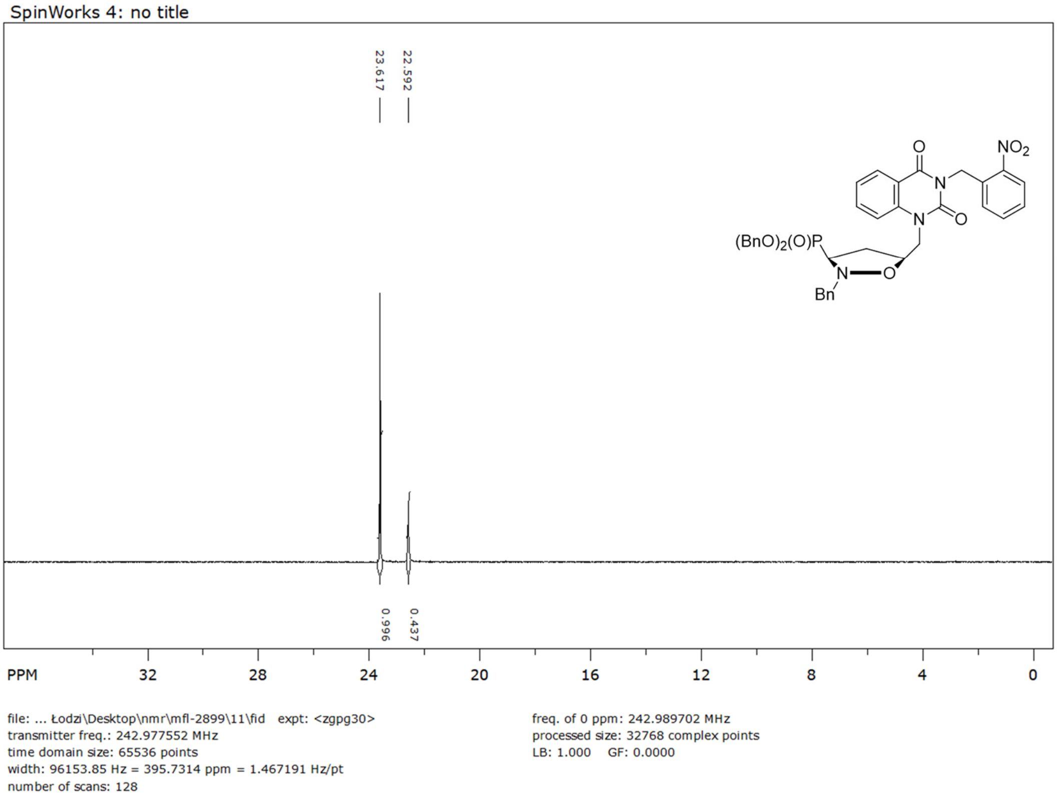

Figure S48: ^{13}C NMR Spectrum for mixture of *cis*-**16e**/*trans*-**16e** (70:30) in CDCl$_3$ and expanded spectral regions

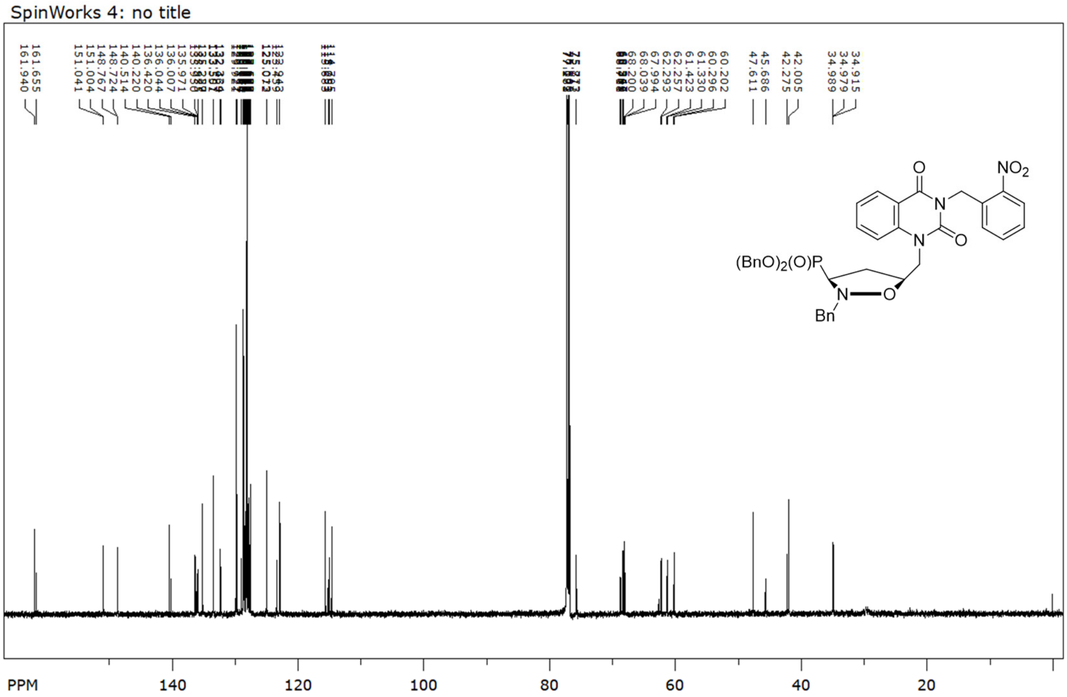

file: ... Łodzi\Desktop\nmr\mfl-2903\10\fid expt: <zgpg30>
transmitter freq.: 150.950591 MHz
time domain size: 65536 points
width: 36057.69 Hz = 238.8708 ppm = 0.550197 Hz/pt
number of scans: 3072

freq. of 0 ppm: 150.935497 MHz
processed size: 32768 complex points
LB: 1.000 GF: 0.0000

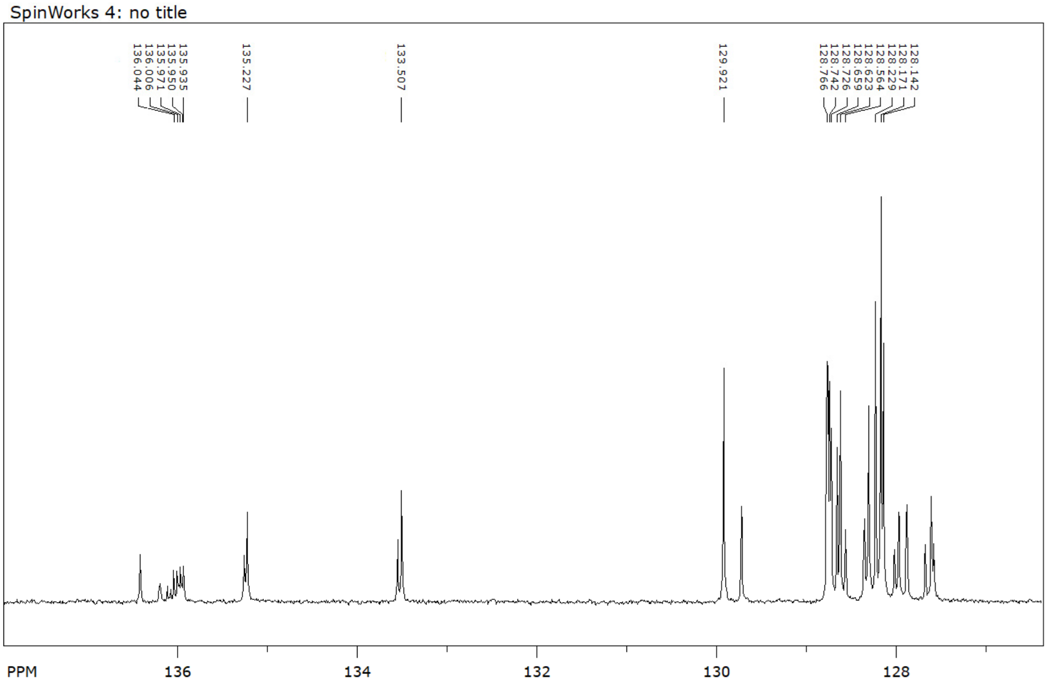

file: ...fl widma\widma 13C\mfl-2903\10\fid expt: <zgpg30>
transmitter freq.: 150.950591 MHz
time domain size: 65536 points
width: 36057.69 Hz = 238.8708 ppm = 0.550197 Hz/pt
number of scans: 3072

freq. of 0 ppm: 150.935497 MHz
processed size: 32768 complex points
LB: 1.000 GF: 0.0000

SpinWorks 4: no title

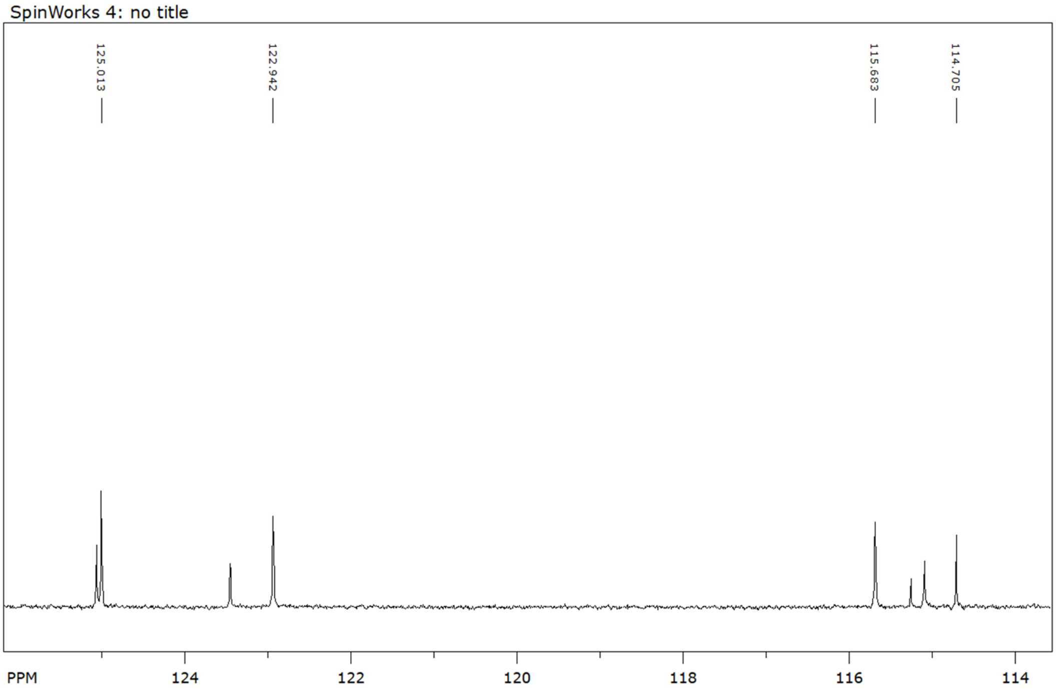

file: ...fl widma\widma 13C\mfl-2903\10\fid   expt: <zgpg30>
transmitter freq.: 150.950591 MHz
time domain size: 65536 points
width: 36057.69 Hz = 238.8708 ppm = 0.550197 Hz/pt
number of scans: 3072

freq. of 0 ppm: 150.935497 MHz
processed size: 32768 complex points
LB: 1.000   GF: 0.0000

SpinWorks 4: no title

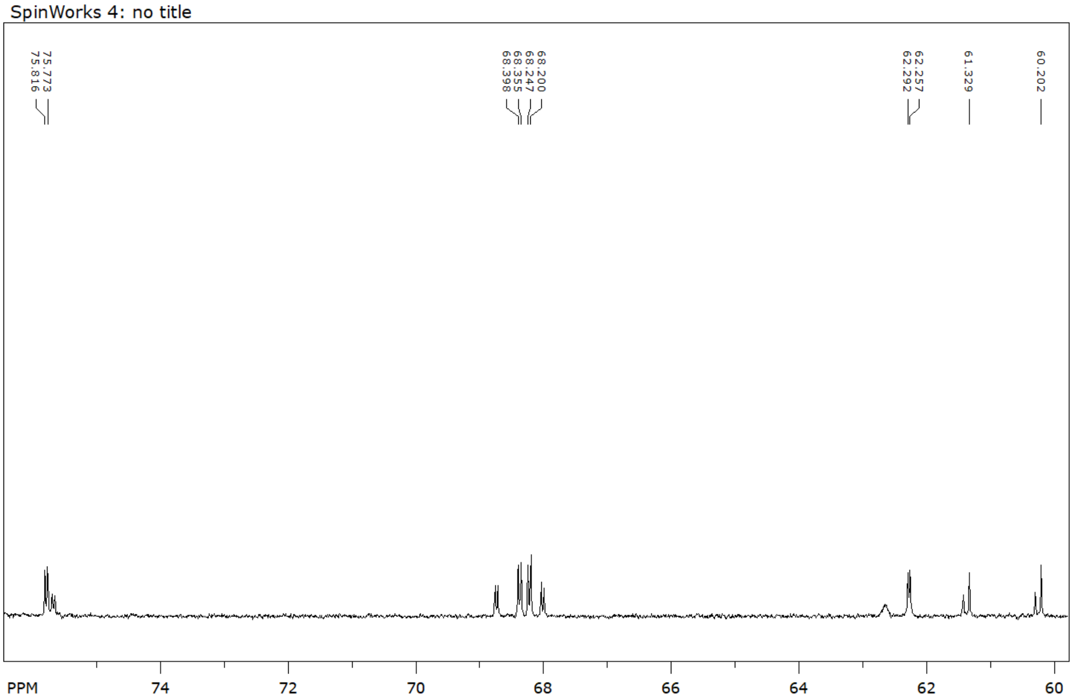

file: ... Łodzi\Desktop\nmr\mfl-2903\10\fid   expt: <zgpg30>
transmitter freq.: 150.950591 MHz
time domain size: 65536 points
width: 36057.69 Hz = 238.8708 ppm = 0.550197 Hz/pt
number of scans: 3072

freq. of 0 ppm: 150.935497 MHz
processed size: 32768 complex points
LB: 1.000   GF: 0.0000

Figure S49: HPLC chromatogram for mixture of *cis*-**16e**/*trans*-**16e** (70:30)

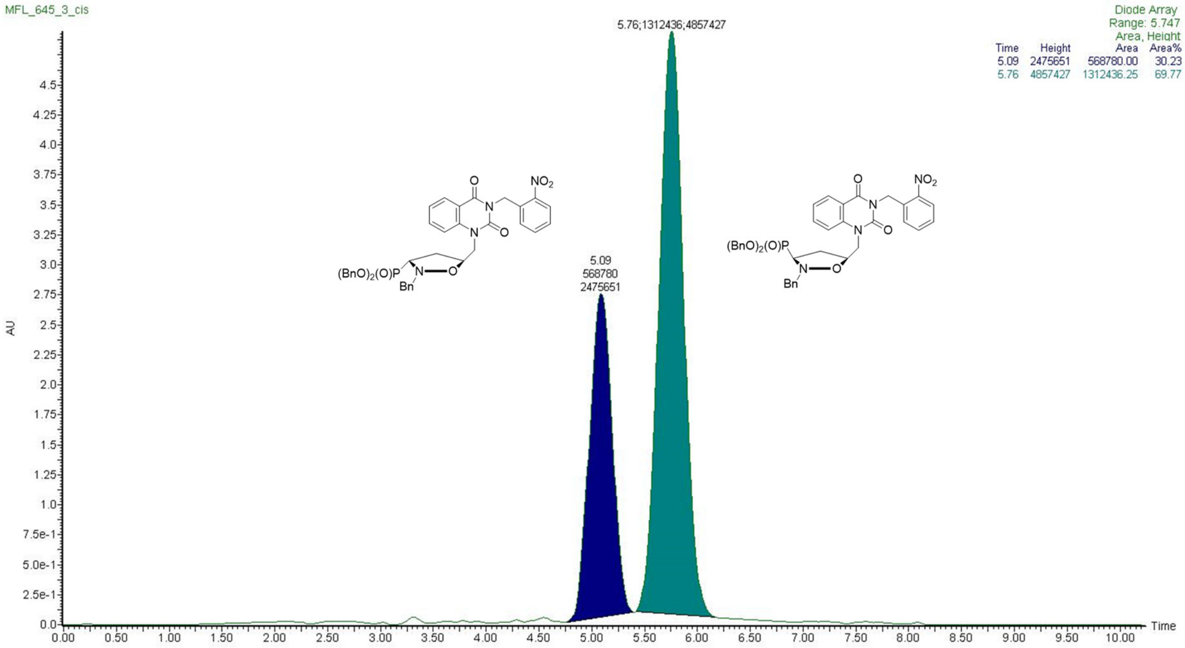

Figure S50: ^{1}H NMR Spectrum for *trans*-**16e** in $CDCl_3$ and expanded spectral regions

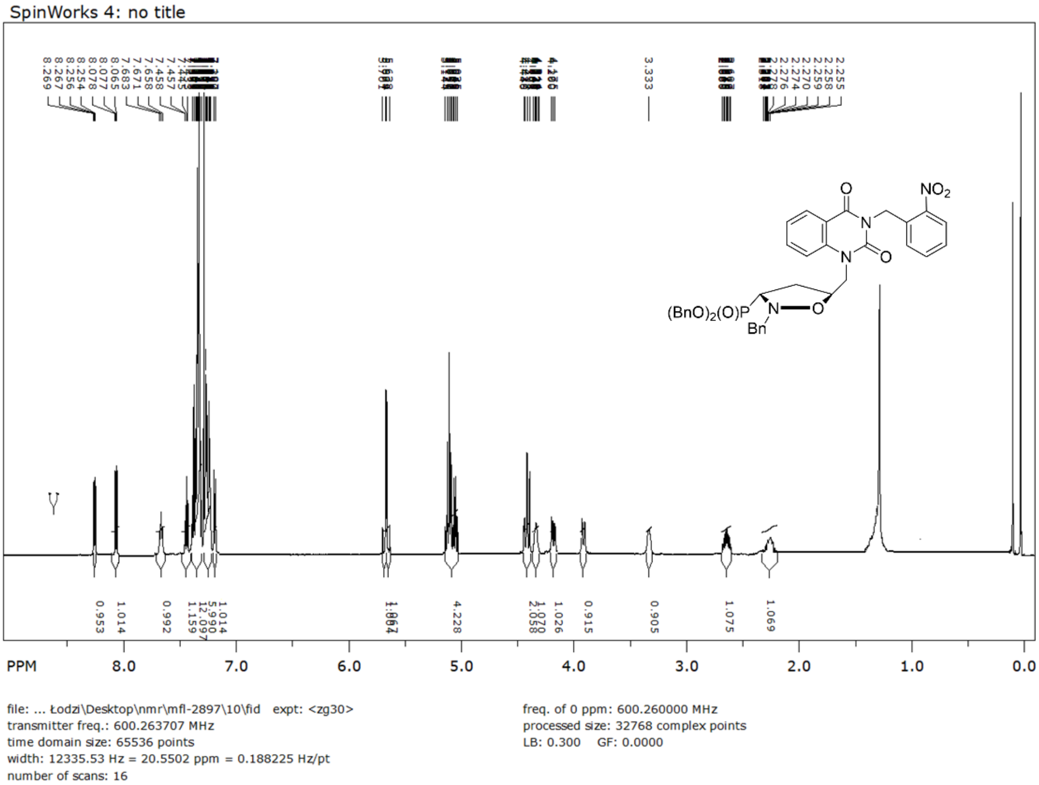

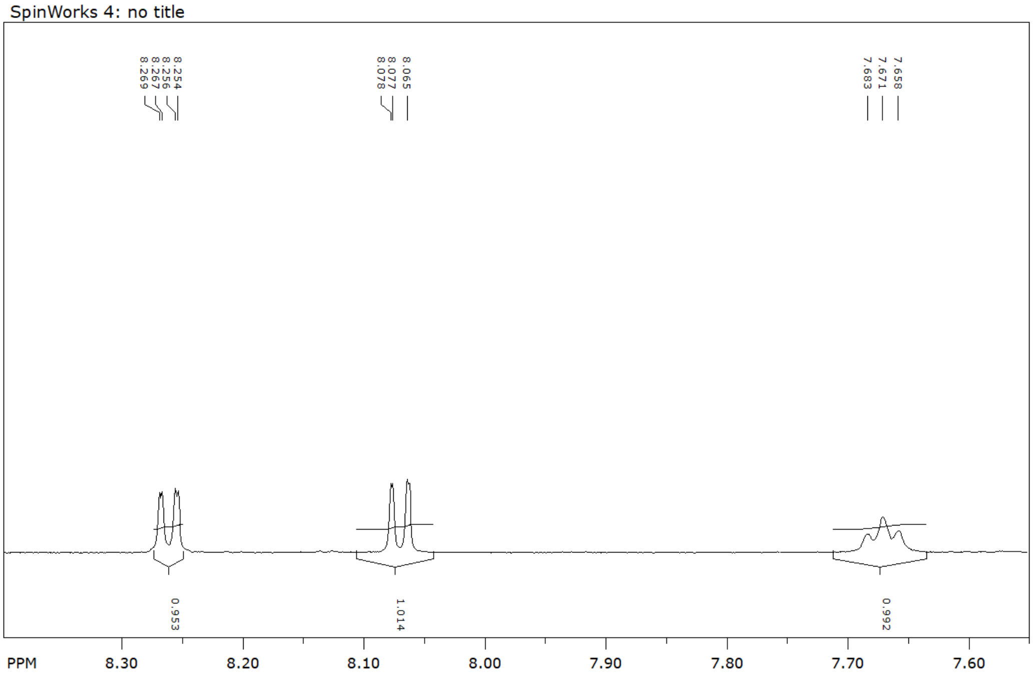

file: ... Łodzi\Desktop\nmr\mfl-2897\10\fid expt: <zg30>
transmitter freq.: 600.263707 MHz
time domain size: 65536 points
width: 12335.53 Hz = 20.5502 ppm = 0.188225 Hz/pt
number of scans: 16

freq. of 0 ppm: 600.260000 MHz
processed size: 32768 complex points
LB: 0.300 GF: 0.0000

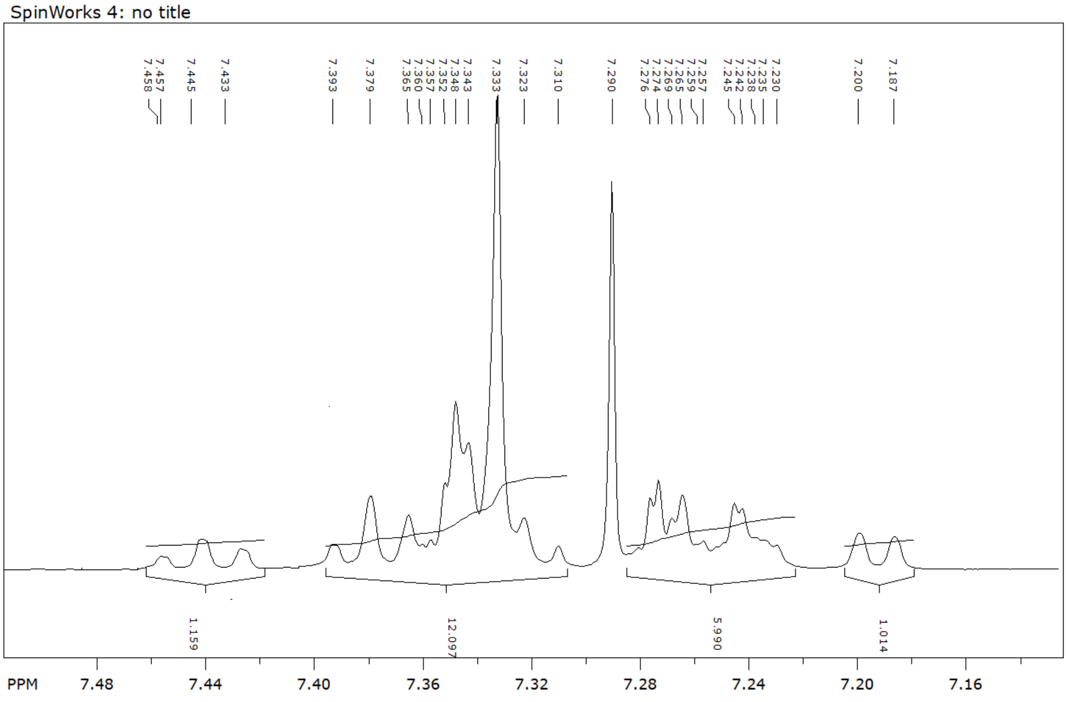

file: ... Łodzi\Desktop\nmr\mfl-2897\10\fid expt: <zg30>
transmitter freq.: 600.263707 MHz
time domain size: 65536 points
width: 12335.53 Hz = 20.5502 ppm = 0.188225 Hz/pt
number of scans: 16

freq. of 0 ppm: 600.260000 MHz
processed size: 32768 complex points
LB: 0.300 GF: 0.0000

SpinWorks 4: no title

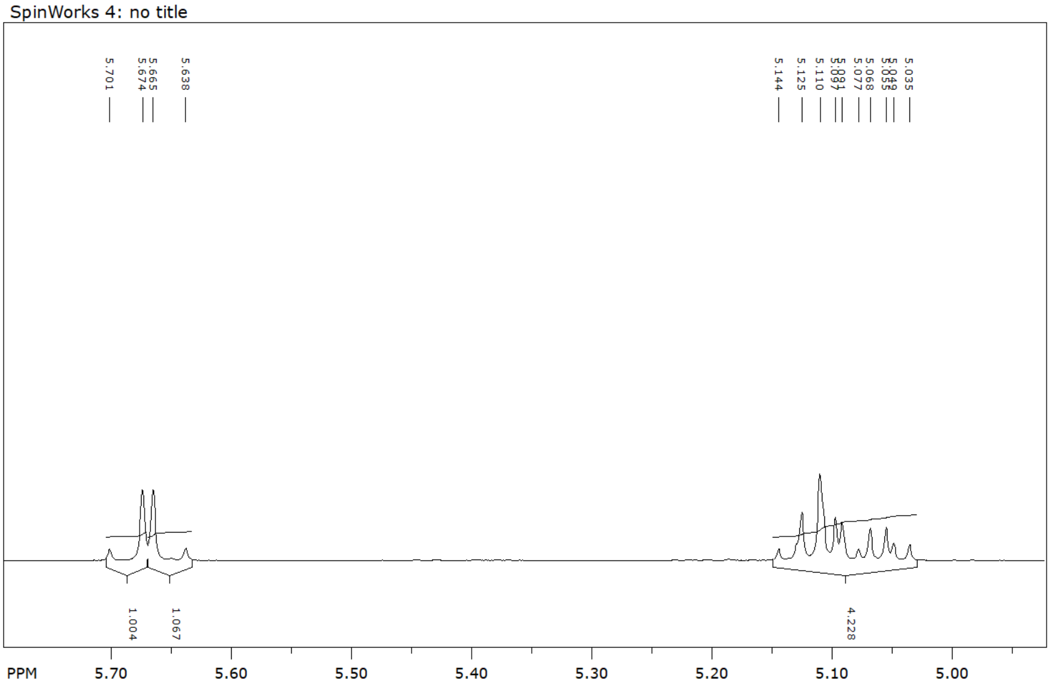

file: ... Łodzi\Desktop\nmr\mfl-2897\10\fid   expt: <zg30>
transmitter freq.: 600.263707 MHz
time domain size: 65536 points
width: 12335.53 Hz = 20.5502 ppm = 0.188225 Hz/pt
number of scans: 16

freq. of 0 ppm: 600.260000 MHz
processed size: 32768 complex points
LB: 0.300   GF: 0.0000

SpinWorks 4: no title

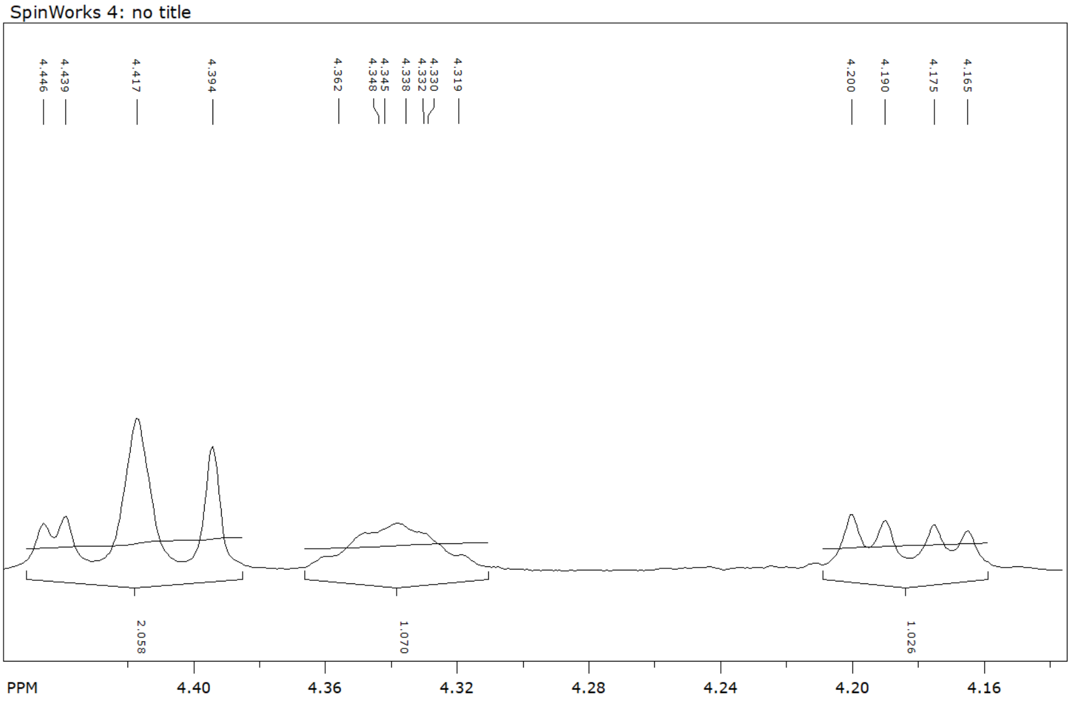

file: ... Łodzi\Desktop\nmr\mfl-2897\10\fid   expt: <zg30>
transmitter freq.: 600.263707 MHz
time domain size: 65536 points
width: 12335.53 Hz = 20.5502 ppm = 0.188225 Hz/pt
number of scans: 16

freq. of 0 ppm: 600.260000 MHz
processed size: 32768 complex points
LB: 0.300   GF: 0.0000

SpinWorks 4: no title

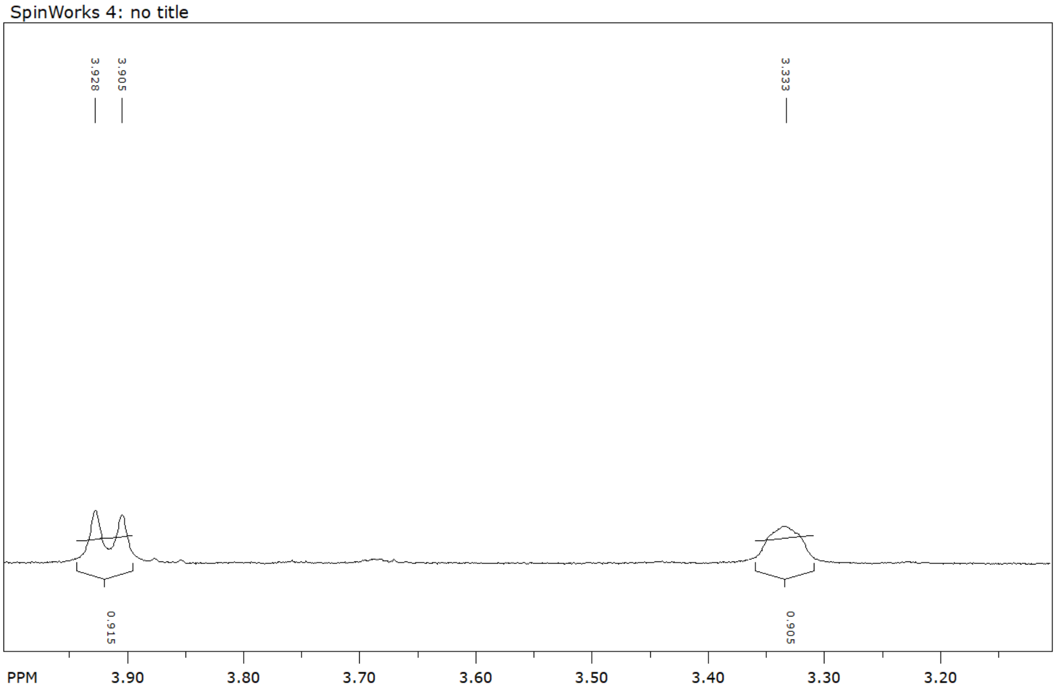

file: ... Łodzi\Desktop\nmr\mfl-2897\10\fid expt: <zg30>
transmitter freq.: 600.263707 MHz
time domain size: 65536 points
width: 12335.53 Hz = 20.5502 ppm = 0.188225 Hz/pt
number of scans: 16

freq. of 0 ppm: 600.260000 MHz
processed size: 32768 complex points
LB: 0.300 GF: 0.0000

SpinWorks 4: no title

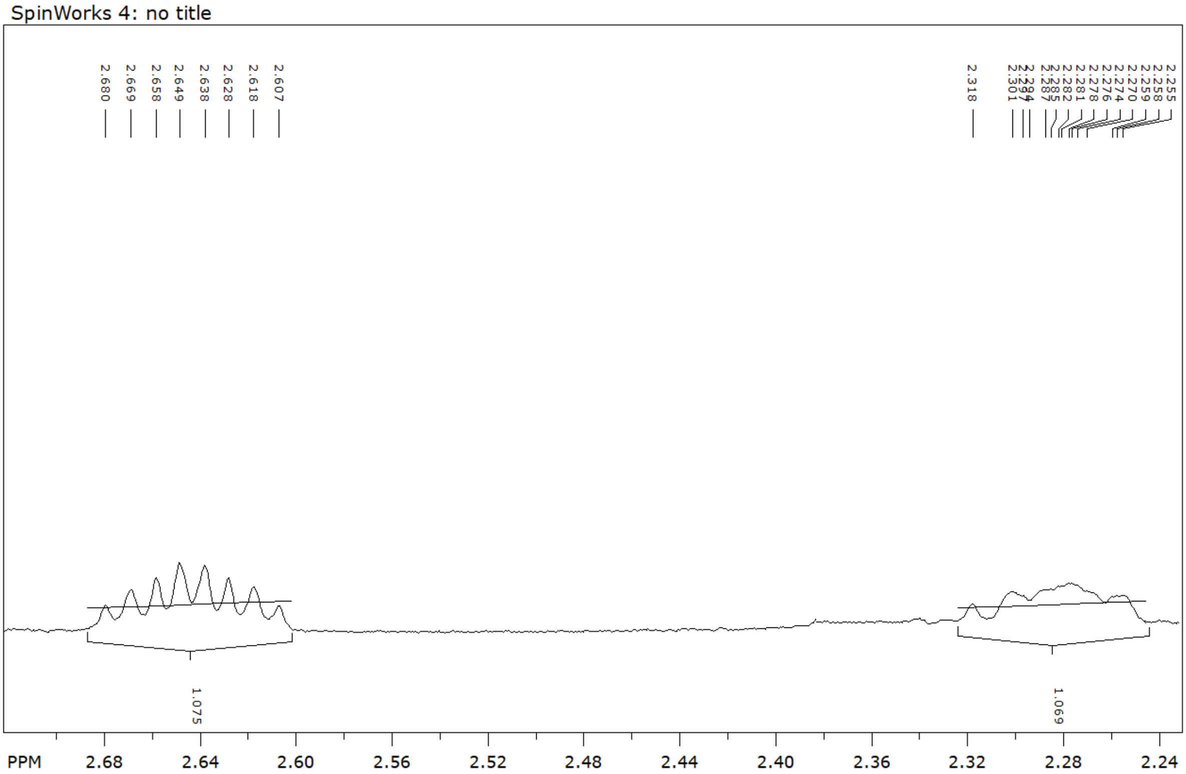

file: ... Łodzi\Desktop\nmr\mfl-2897\10\fid expt: <zg30>
transmitter freq.: 600.263707 MHz
time domain size: 65536 points
width: 12335.53 Hz = 20.5502 ppm = 0.188225 Hz/pt
number of scans: 16

freq. of 0 ppm: 600.260000 MHz
processed size: 32768 complex points
LB: 0.300 GF: 0.0000

Figure S51: ^{31}P NMR Spectrum for *trans*-**16e** in $CDCl_3$

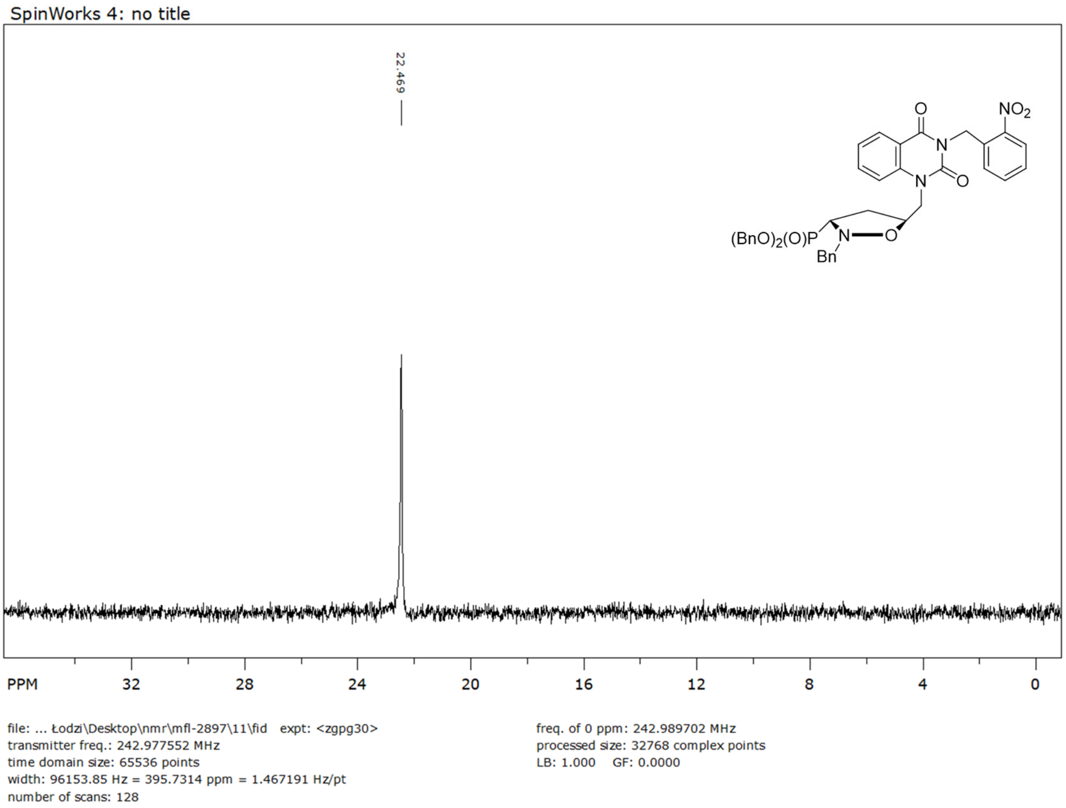

Figure S52: ^{13}C NMR Spectrum for *trans*-**16e** in $CDCl_3$ and expanded spectral regions

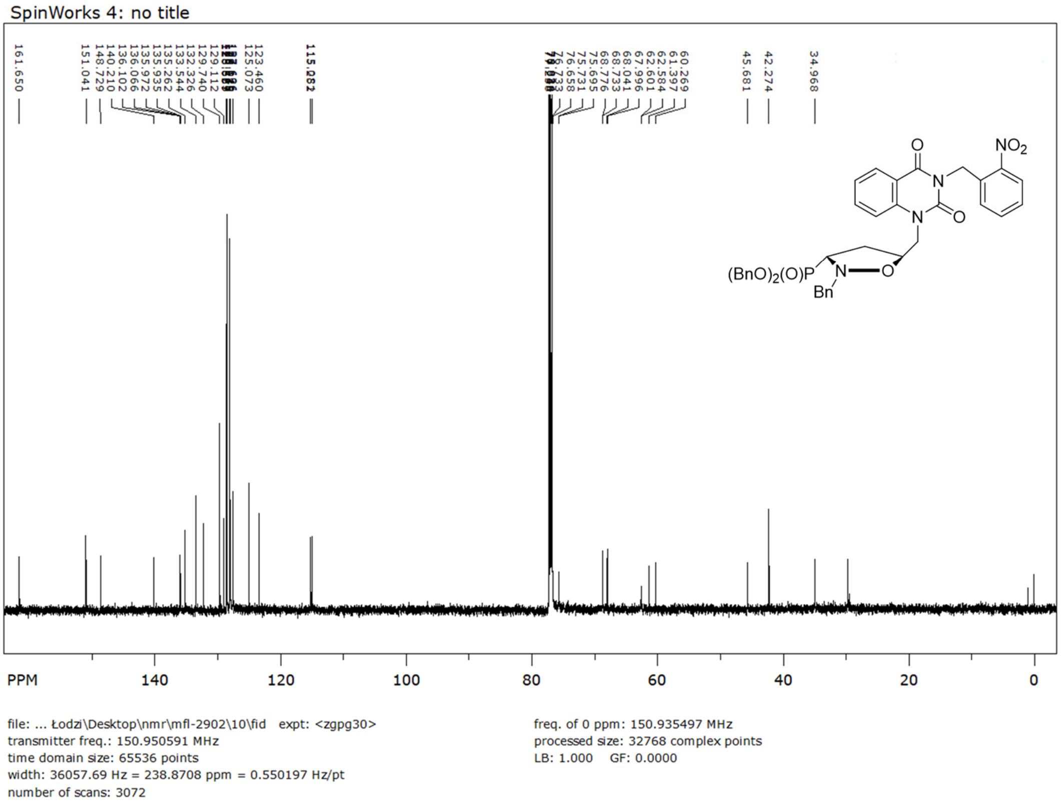

SpinWorks 4: no title

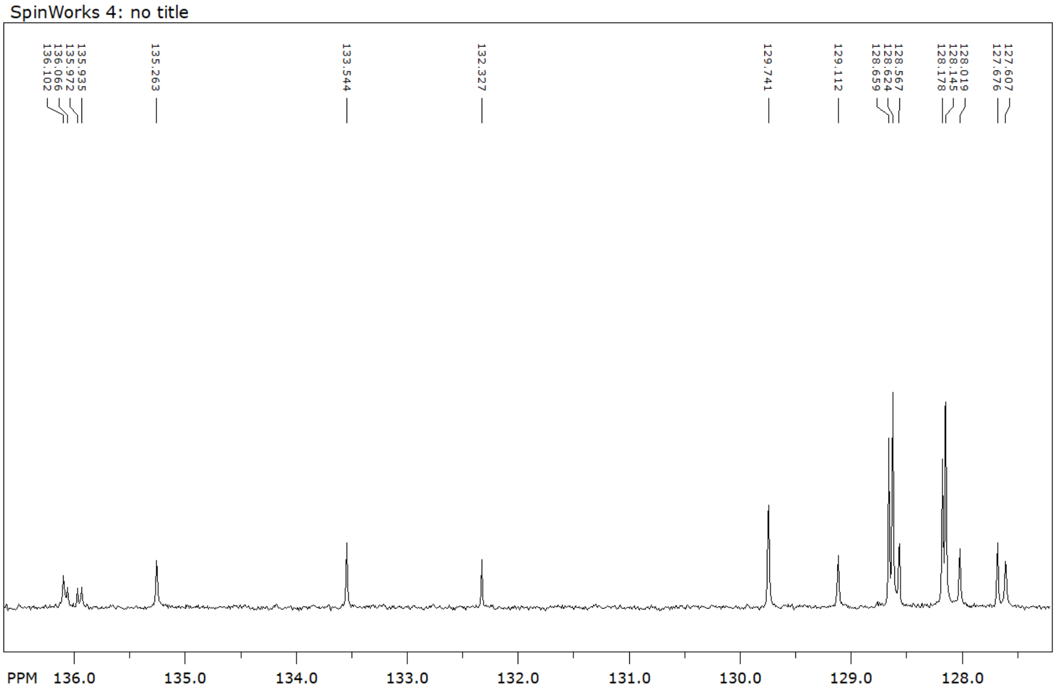

file: ...fl widma\widma 13C\mfl-2902\10\fid expt: <zgpg30>
transmitter freq.: 150.950591 MHz
time domain size: 65536 points
width: 36057.69 Hz = 238.8708 ppm = 0.550197 Hz/pt
number of scans: 3072

freq. of 0 ppm: 150.935497 MHz
processed size: 32768 complex points
LB: 1.000 GF: 0.0000

SpinWorks 4: no title

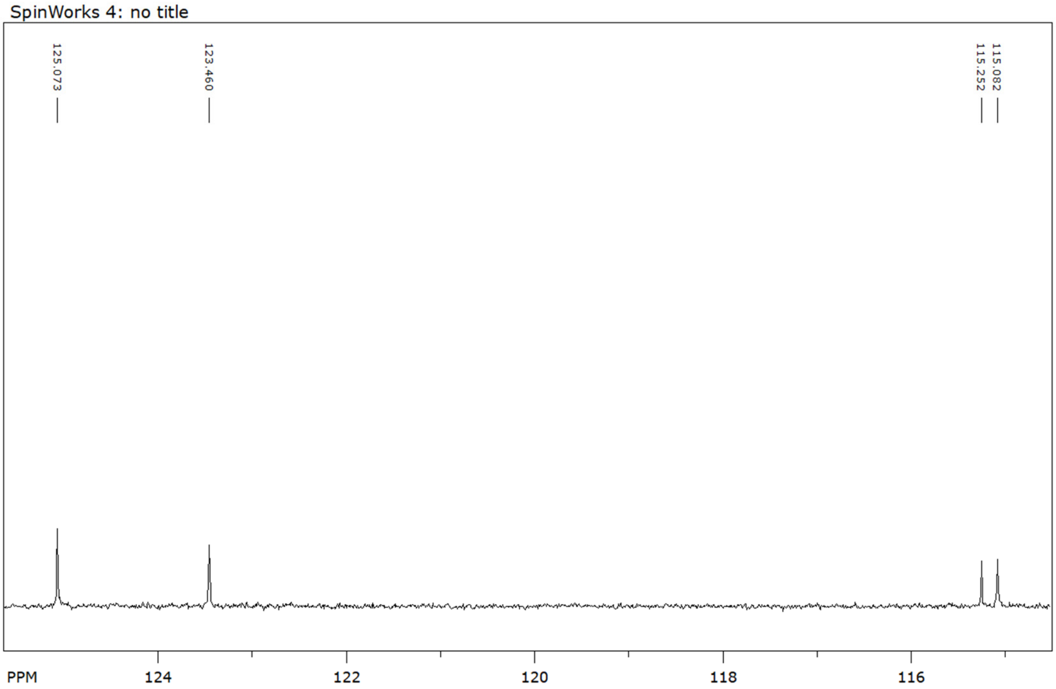

file: ...fl widma\widma 13C\mfl-2902\10\fid expt: <zgpg30>
transmitter freq.: 150.950591 MHz
time domain size: 65536 points
width: 36057.69 Hz = 238.8708 ppm = 0.550197 Hz/pt
number of scans: 3072

freq. of 0 ppm: 150.935497 MHz
processed size: 32768 complex points
LB: 1.000 GF: 0.0000

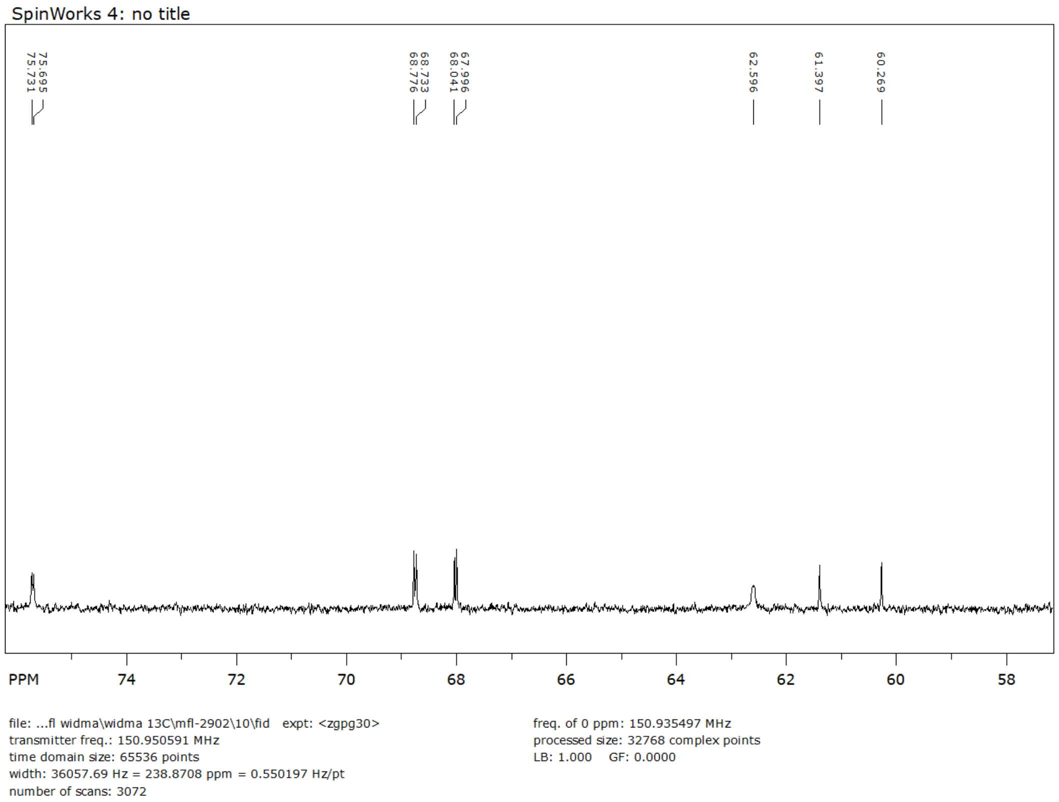

Figure S53: HPLC chromatogram for *trans*-**16e**

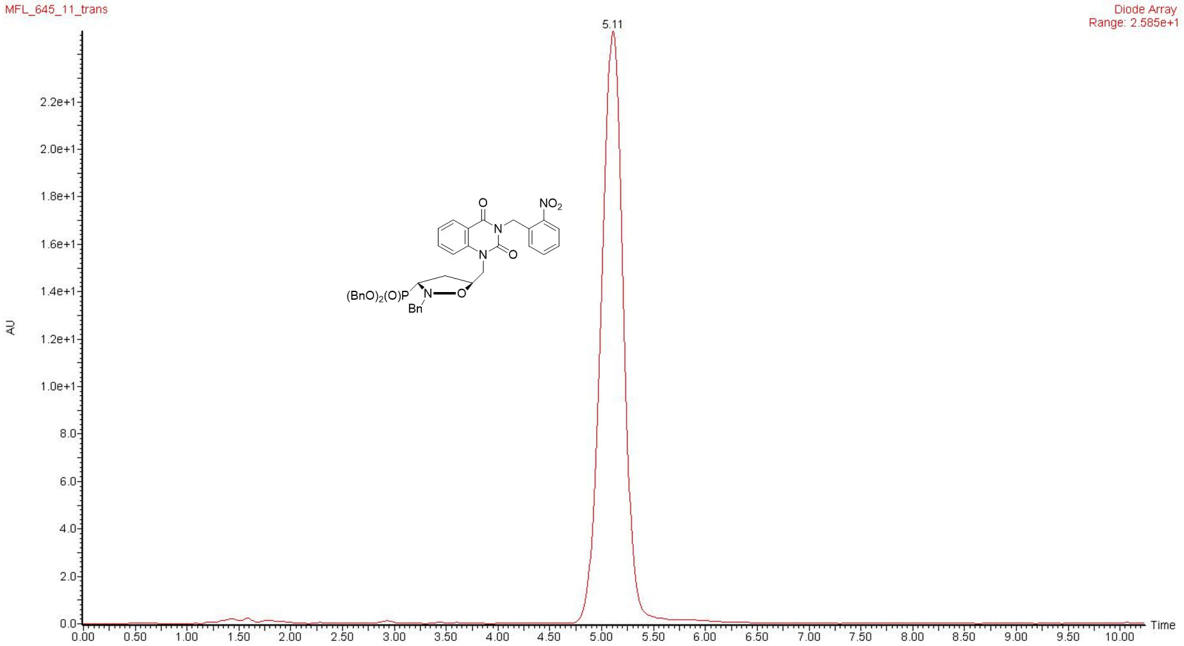

Figure S54: ^{1}H NMR Spectrum for mixture of *cis*-**16f**/*trans*-**16f** (96:4) in $CDCl_3$ and expanded spectral regions

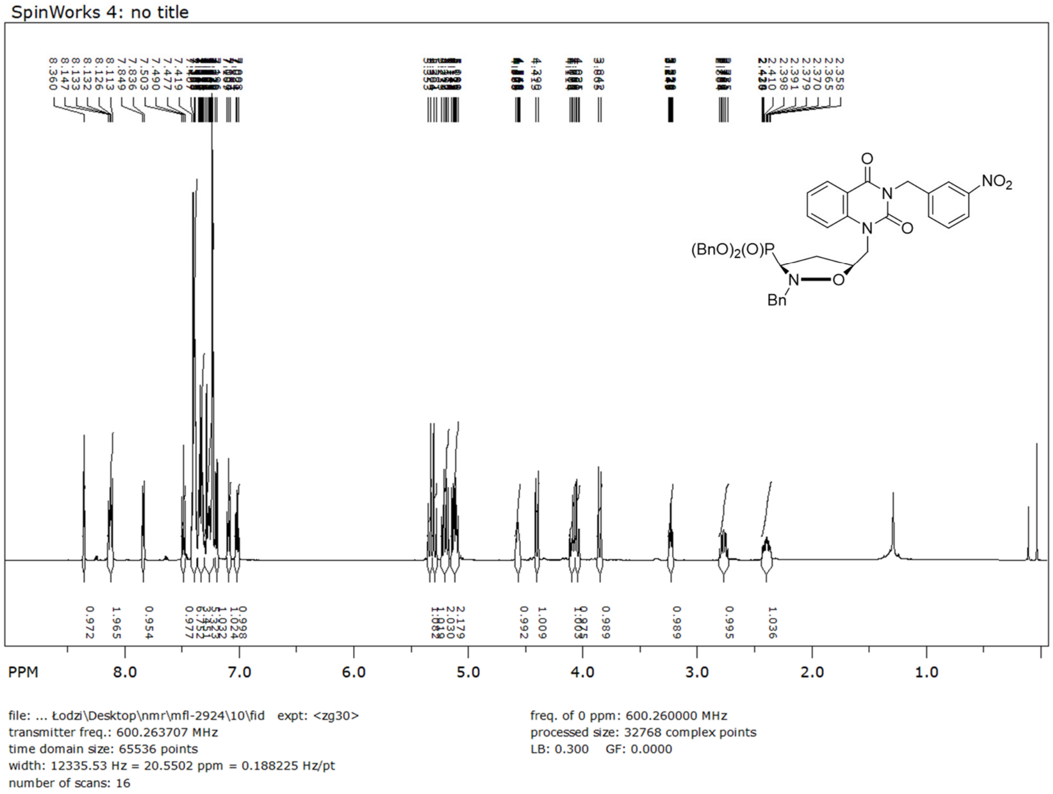

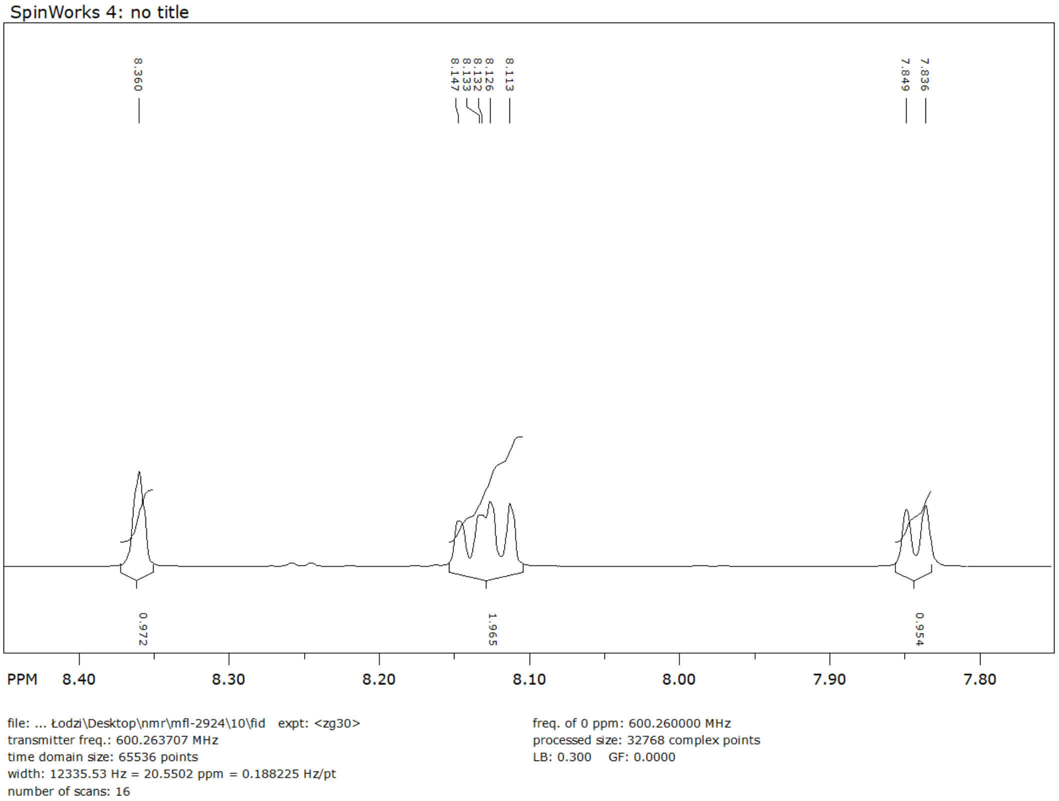

SpinWorks 4: no title

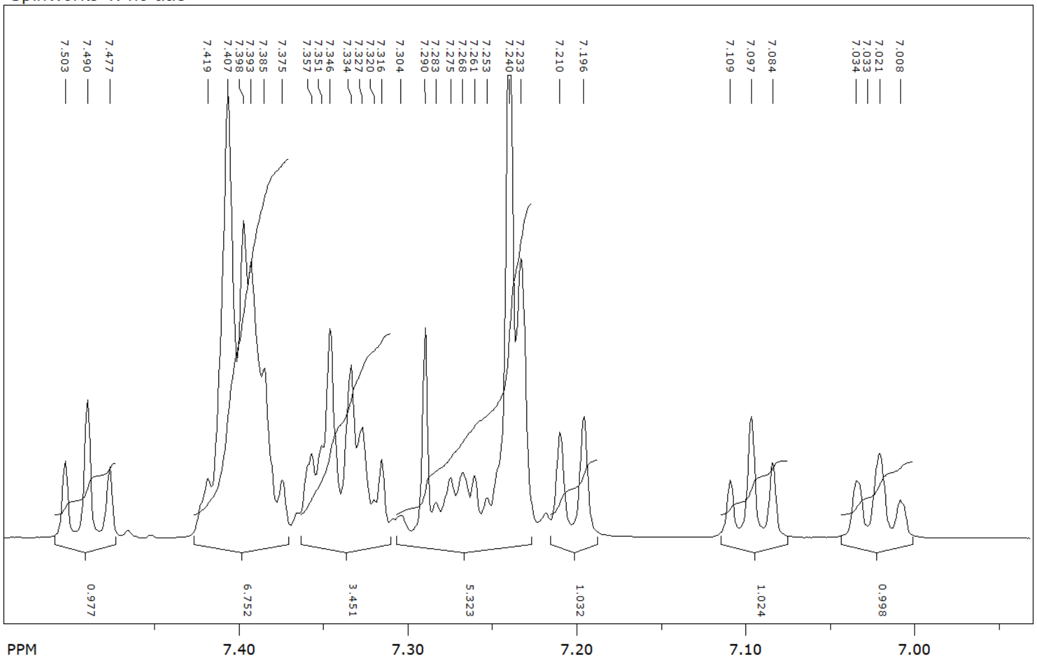

file: ... Łodzi\Desktop\nmr\mfl-2924\10\fid expt: <zg30>
transmitter freq.: 600.263707 MHz
time domain size: 65536 points
width: 12335.53 Hz = 20.5502 ppm = 0.188225 Hz/pt
number of scans: 16

freq. of 0 ppm: 600.260000 MHz
processed size: 32768 complex points
LB: 0.300 GF: 0.0000

SpinWorks 4: no title

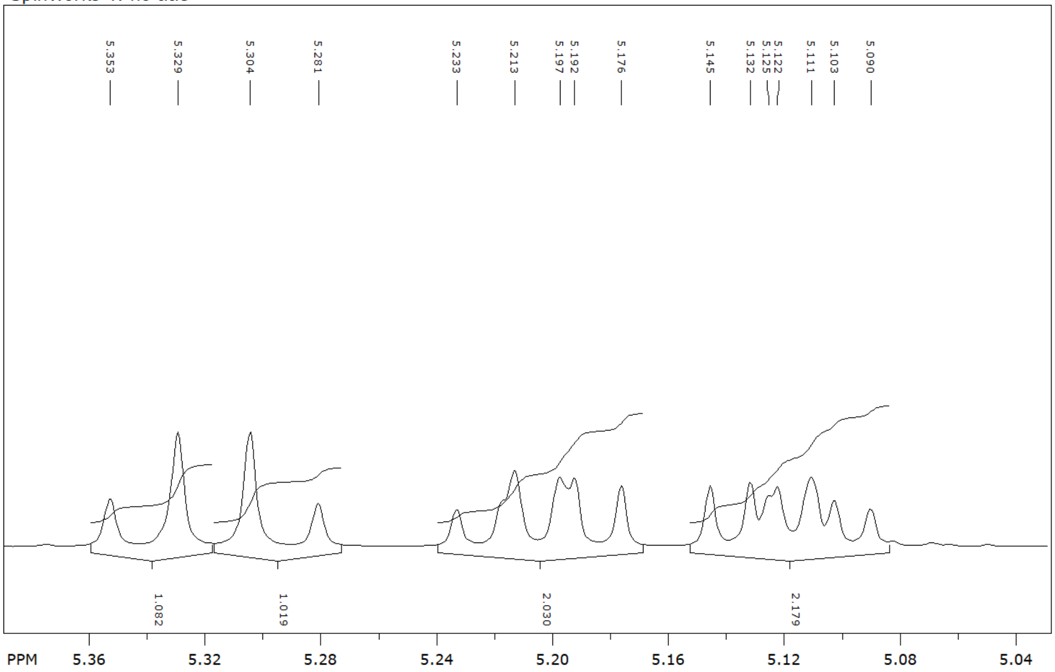

file: ... Łodzi\Desktop\nmr\mfl-2924\10\fid expt: <zg30>
transmitter freq.: 600.263707 MHz
time domain size: 65536 points
width: 12335.53 Hz = 20.5502 ppm = 0.188225 Hz/pt
number of scans: 16

freq. of 0 ppm: 600.260000 MHz
processed size: 32768 complex points
LB: 0.300 GF: 0.0000

SpinWorks 4: no title

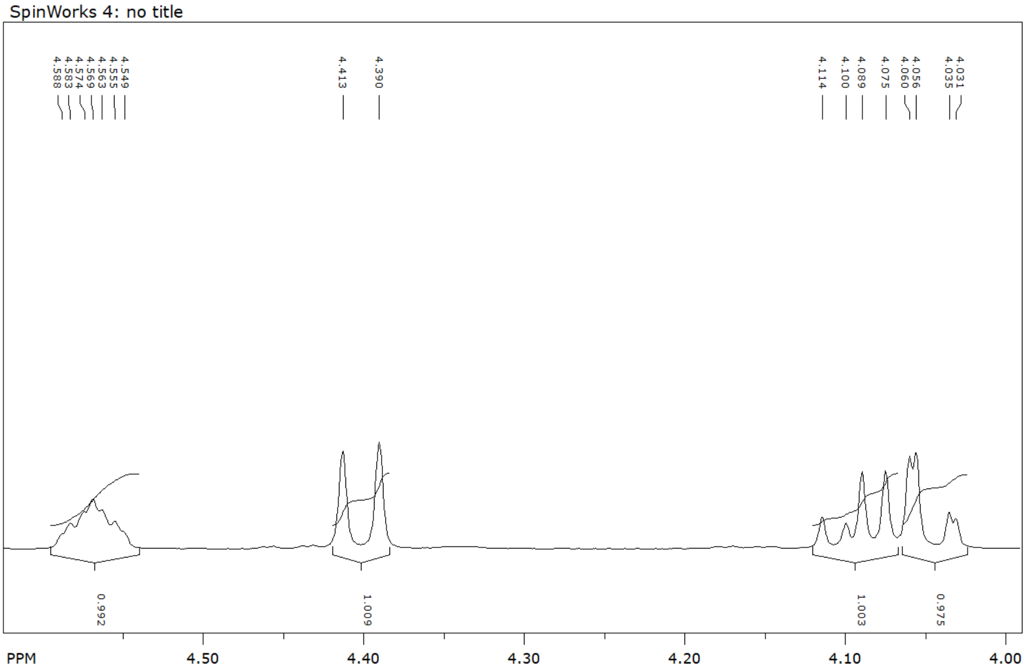

file: ... Łodzi\Desktop\nmr\mfl-2924\10\fid   expt: <zg30>
transmitter freq.: 600.263707 MHz
time domain size: 65536 points
width: 12335.53 Hz = 20.5502 ppm = 0.188225 Hz/pt
number of scans: 16

freq. of 0 ppm: 600.260000 MHz
processed size: 32768 complex points
LB: 0.300   GF: 0.0000

SpinWorks 4: no title

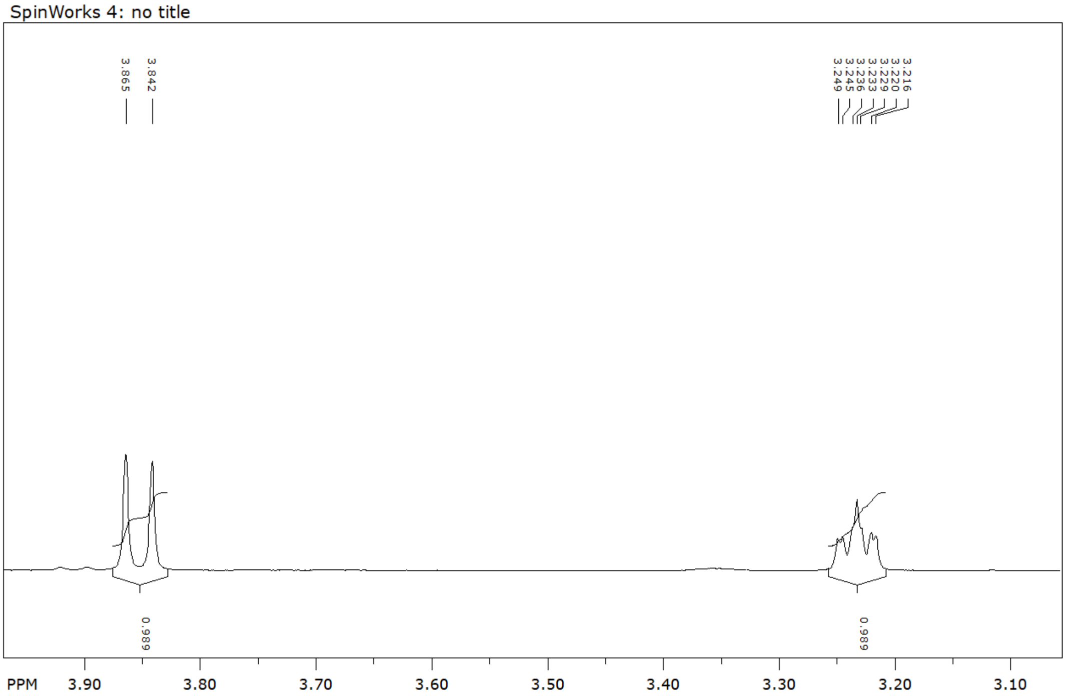

file: ... Łodzi\Desktop\nmr\mfl-2924\10\fid   expt: <zg30>
transmitter freq.: 600.263707 MHz
time domain size: 65536 points
width: 12335.53 Hz = 20.5502 ppm = 0.188225 Hz/pt
number of scans: 16

freq. of 0 ppm: 600.260000 MHz
processed size: 32768 complex points
LB: 0.300   GF: 0.0000

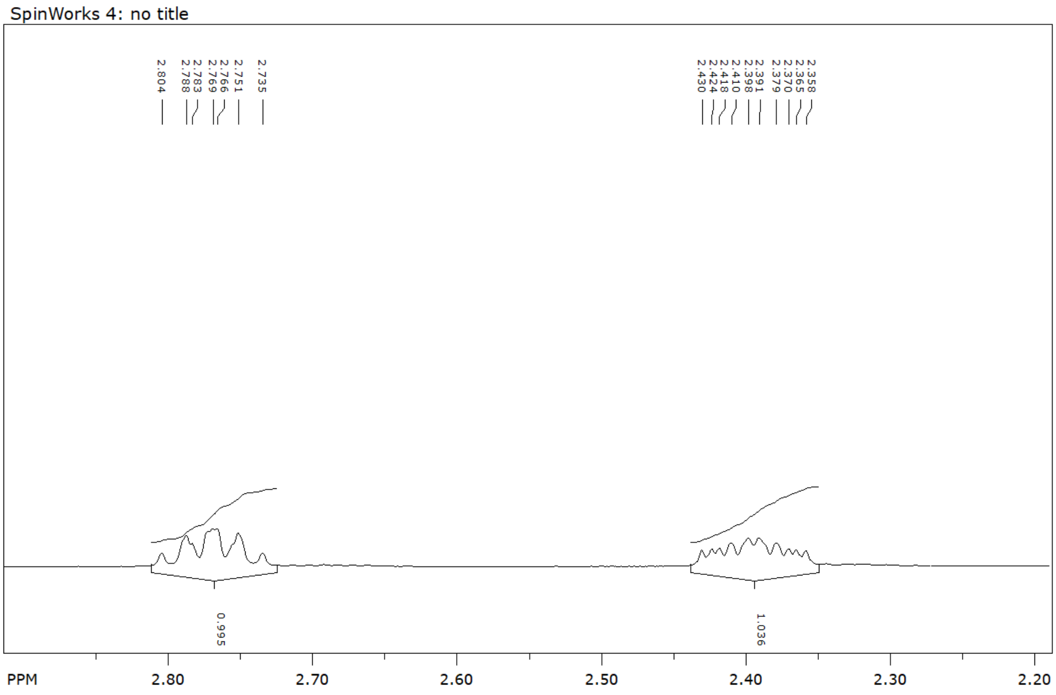

file: ... Łodzi\Desktop\nmr\mfl-2924\10\fid expt: <zg30>
transmitter freq.: 600.263707 MHz
time domain size: 65536 points
width: 12335.53 Hz = 20.5502 ppm = 0.188225 Hz/pt
number of scans: 16

freq. of 0 ppm: 600.260000 MHz
processed size: 32768 complex points
LB: 0.300 GF: 0.0000

Figure S55: ^{31}P NMR Spectrum for mixture of *cis*-**16f**/*trans*-**16f** (96:4) in $CDCl_3$

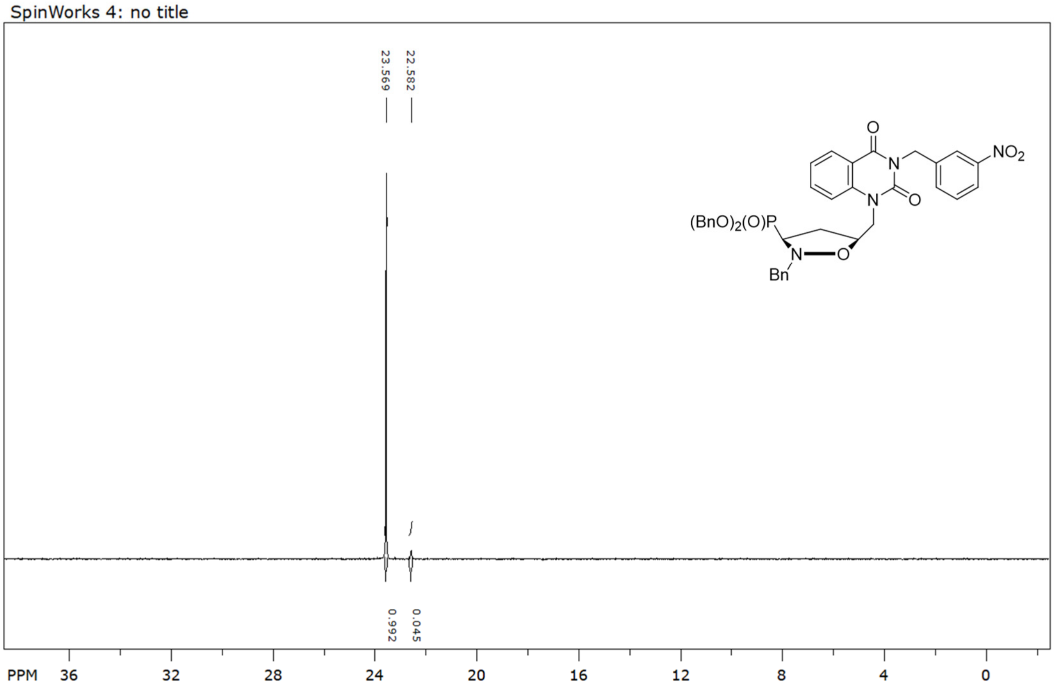

file: ... Łodzi\Desktop\nmr\mfl-2924\11\fid expt: <zgpg30>
transmitter freq.: 242.977552 MHz
time domain size: 65536 points
width: 96153.85 Hz = 395.7314 ppm = 1.467191 Hz/pt
number of scans: 128

freq. of 0 ppm: 242.989702 MHz
processed size: 32768 complex points
LB: 1.000 GF: 0.0000

Figure S56: ^{13}C NMR Spectrum for mixture of *cis*-**16f**/*trans*-**16f** (96:4) in $CDCl_3$ and expanded spectral regions

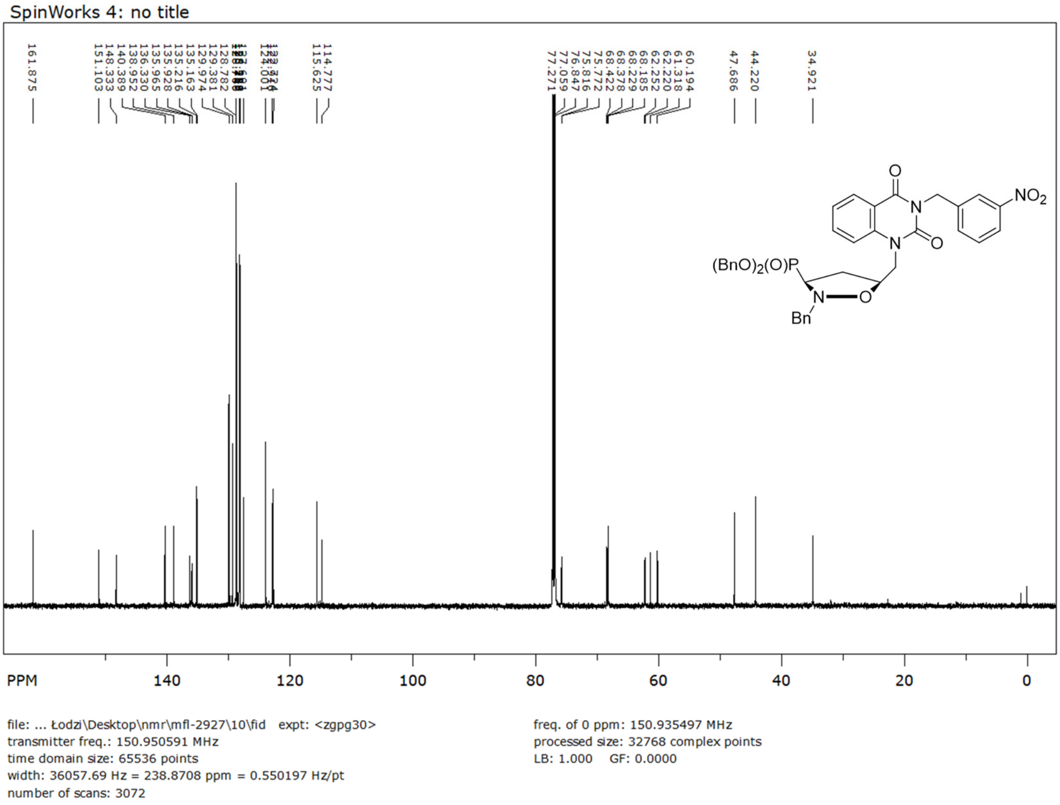

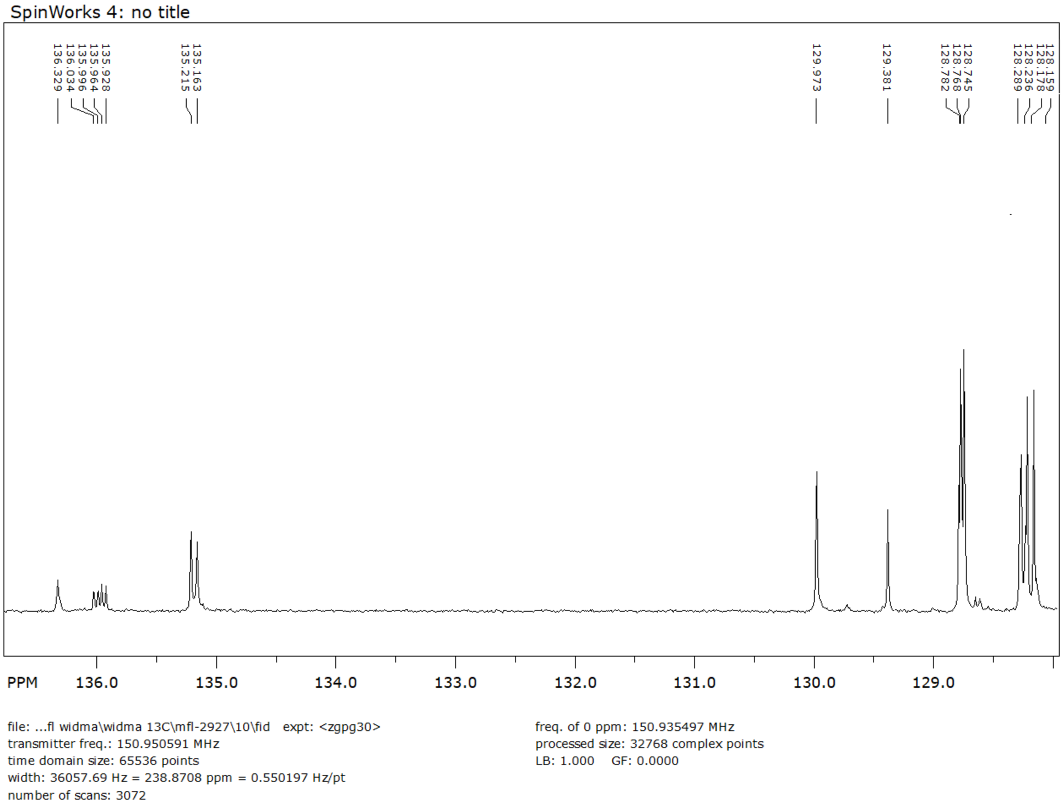

SpinWorks 4: no title

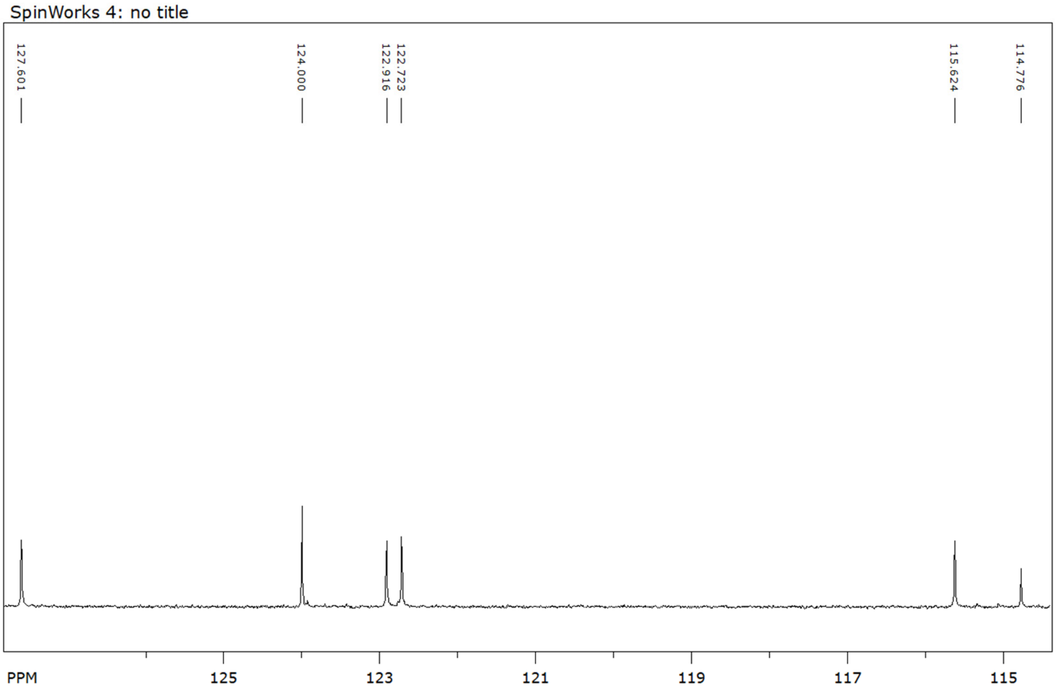

file: ...fl widma\widma 13C\mfl-2927\10\fid expt: <zgpg30>
transmitter freq.: 150.950591 MHz
time domain size: 65536 points
width: 36057.69 Hz = 238.8708 ppm = 0.550197 Hz/pt
number of scans: 3072

freq. of 0 ppm: 150.935497 MHz
processed size: 32768 complex points
LB: 1.000 GF: 0.0000

SpinWorks 4: no title

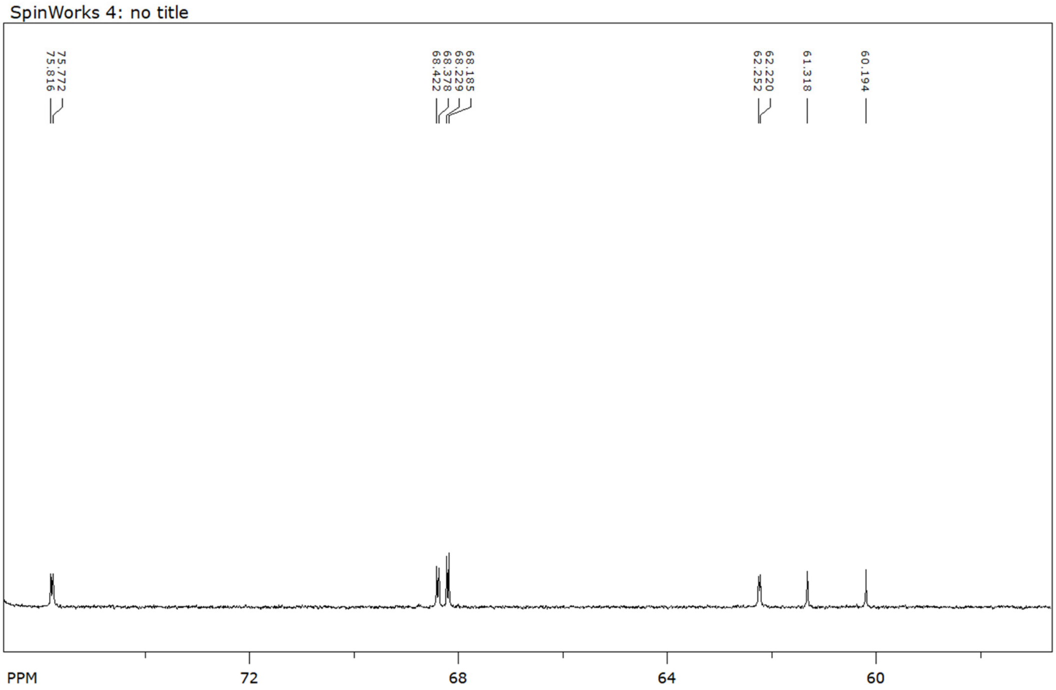

file: ...fl widma\widma 13C\mfl-2927\10\fid expt: <zgpg30>
transmitter freq.: 150.950591 MHz
time domain size: 65536 points
width: 36057.69 Hz = 238.8708 ppm = 0.550197 Hz/pt
number of scans: 3072

freq. of 0 ppm: 150.935497 MHz
processed size: 32768 complex points
LB: 1.000 GF: 0.0000

Figure S57: HPLC chromatogram for mixture of *cis*-**16f**/*trans*-**16f** (96:4)

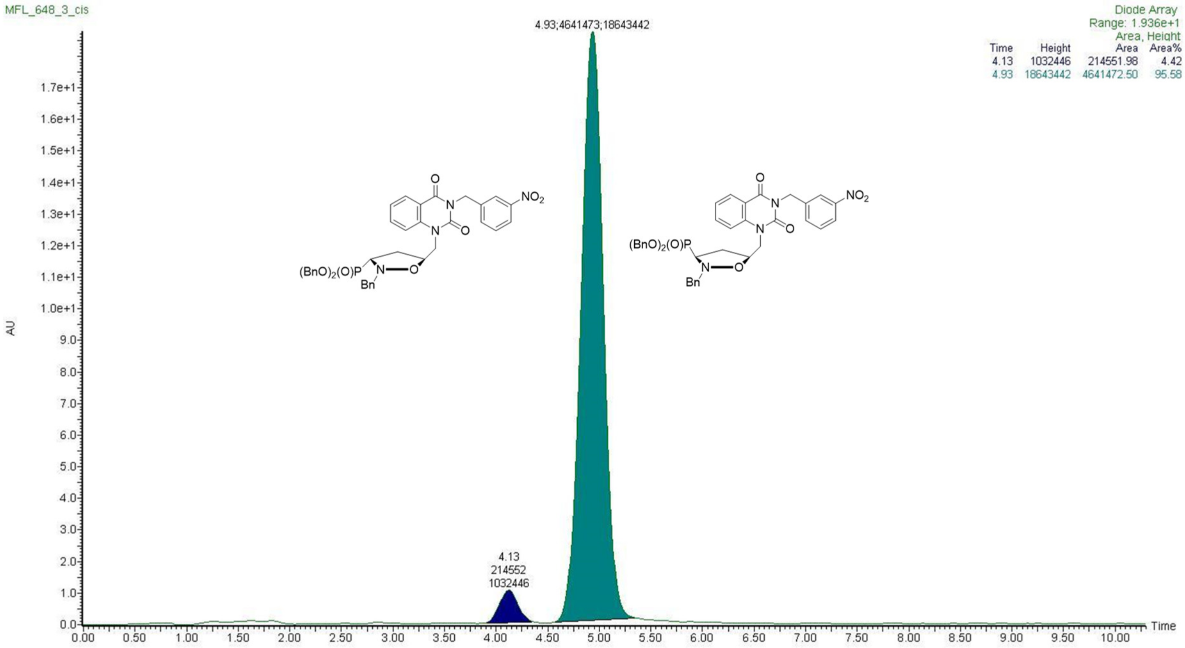

Figure S58: ^{1}H NMR Spectrum for *trans*-**16f** in CDCl3 and expanded spectral regions

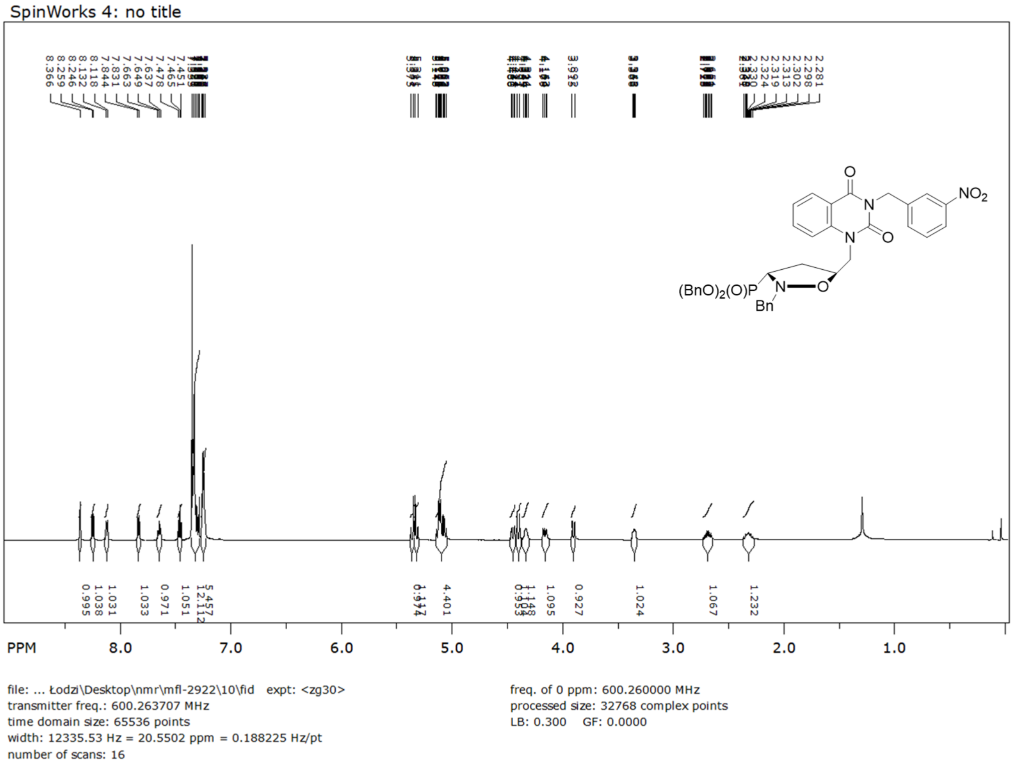

SpinWorks 4: no title

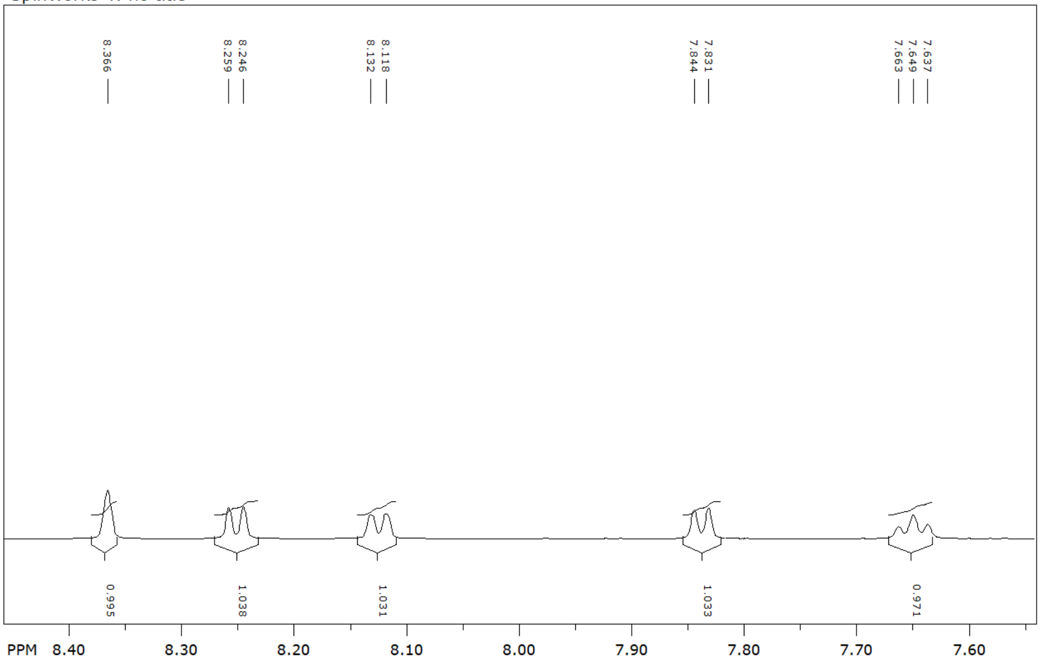

file: ... Łodzi\Desktop\nmr\mfl-2922\10\fid expt: <zg30>
transmitter freq.: 600.263707 MHz
time domain size: 65536 points
width: 12335.53 Hz = 20.5502 ppm = 0.188225 Hz/pt
number of scans: 16

freq. of 0 ppm: 600.260000 MHz
processed size: 32768 complex points
LB: 0.300 GF: 0.0000

SpinWorks 4: no title

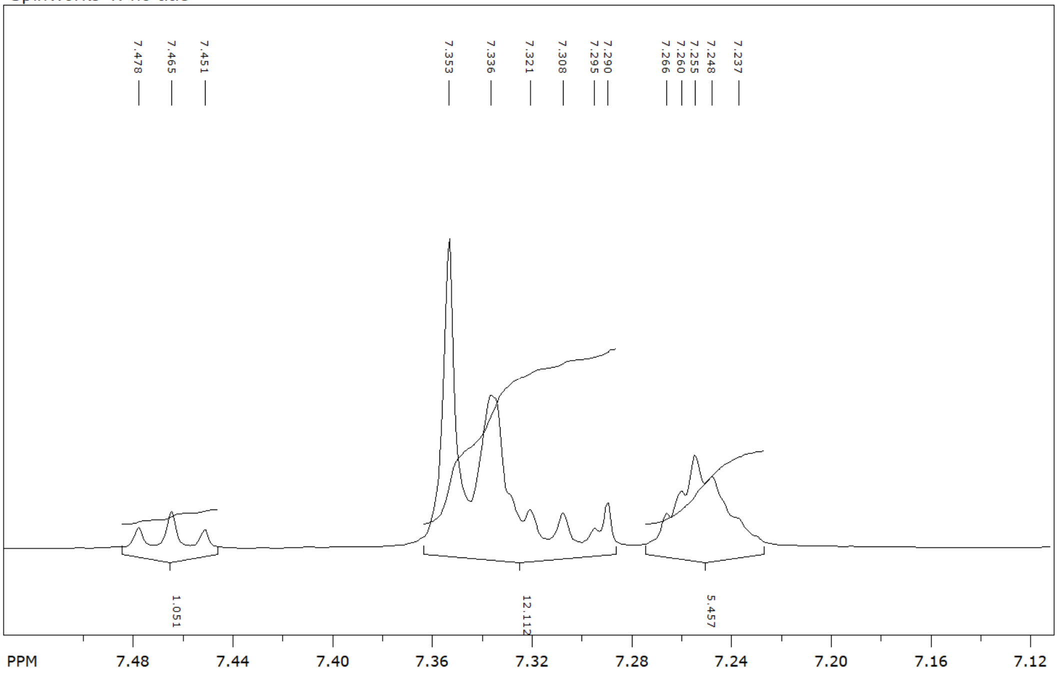

file: ... Łodzi\Desktop\nmr\mfl-2922\10\fid expt: <zg30>
transmitter freq.: 600.263707 MHz
time domain size: 65536 points
width: 12335.53 Hz = 20.5502 ppm = 0.188225 Hz/pt
number of scans: 16

freq. of 0 ppm: 600.260000 MHz
processed size: 32768 complex points
LB: 0.300 GF: 0.0000

SpinWorks 4: no title

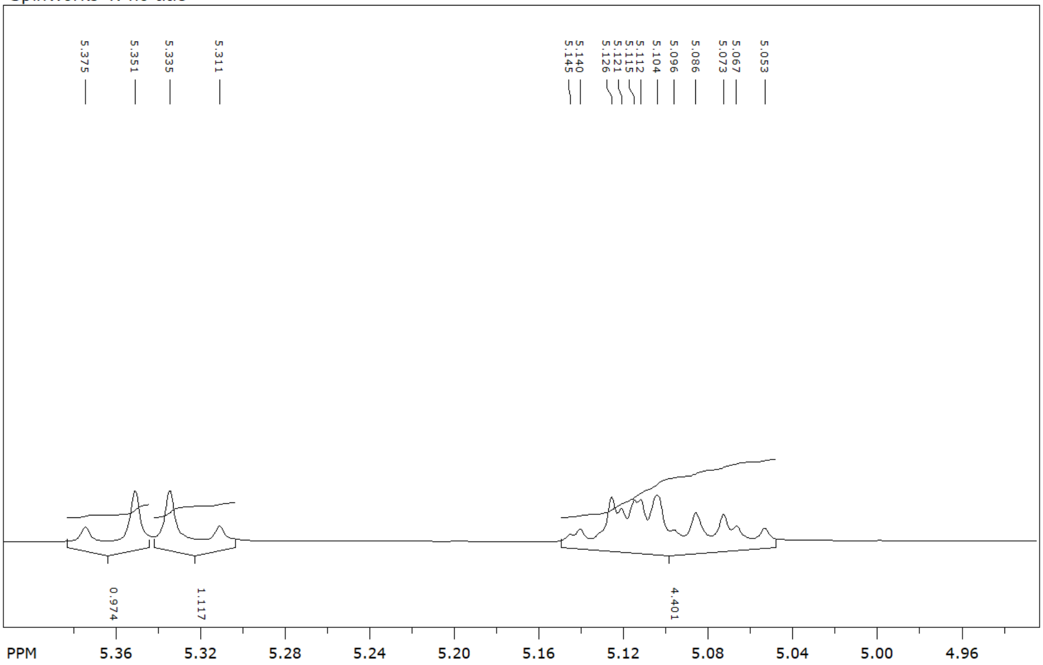

file: ... Łodzi\Desktop\nmr\mfl-2922\10\fid expt: <zg30>
transmitter freq.: 600.263707 MHz
time domain size: 65536 points
width: 12335.53 Hz = 20.5502 ppm = 0.188225 Hz/pt
number of scans: 16

freq. of 0 ppm: 600.260000 MHz
processed size: 32768 complex points
LB: 0.300 GF: 0.0000

SpinWorks 4: no title

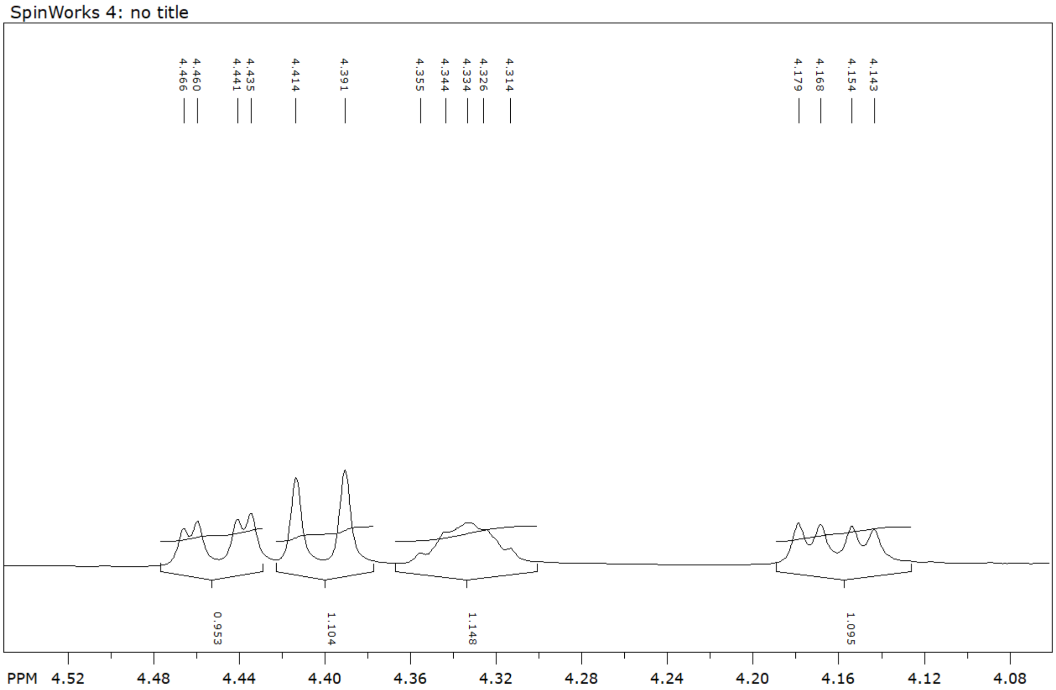

file: ... Łodzi\Desktop\nmr\mfl-2922\10\fid expt: <zg30>
transmitter freq.: 600.263707 MHz
time domain size: 65536 points
width: 12335.53 Hz = 20.5502 ppm = 0.188225 Hz/pt
number of scans: 16

freq. of 0 ppm: 600.260000 MHz
processed size: 32768 complex points
LB: 0.300 GF: 0.0000

SpinWorks 4: no title

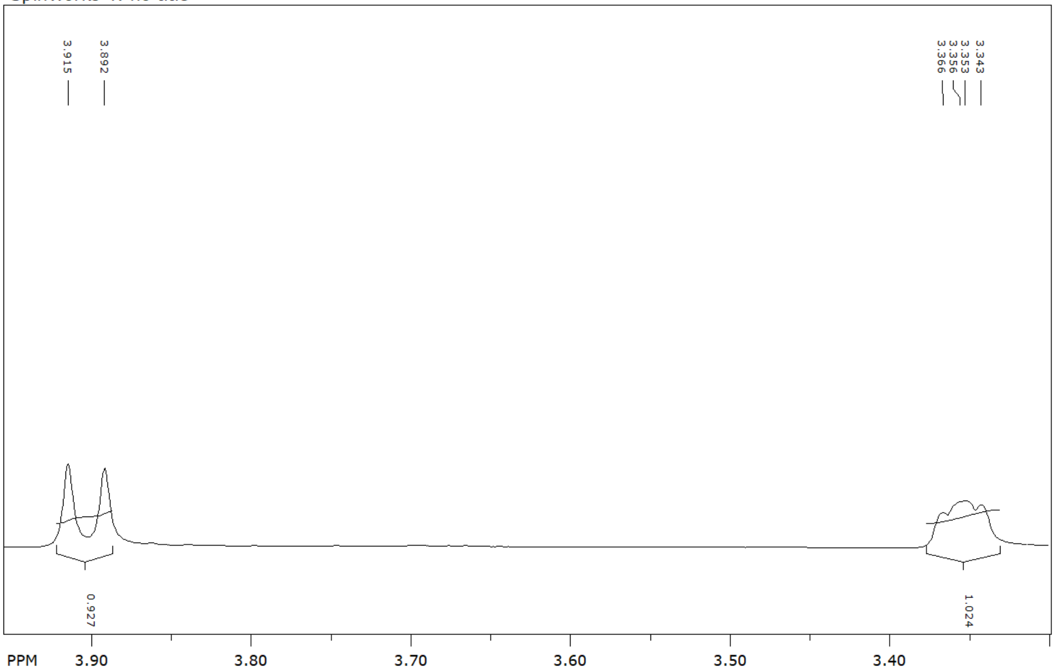

file: ... Łodzi\Desktop\nmr\mfl-2922\10\fid expt: <zg30>
transmitter freq.: 600.263707 MHz
time domain size: 65536 points
width: 12335.53 Hz = 20.5502 ppm = 0.188225 Hz/pt
number of scans: 16

freq. of 0 ppm: 600.260000 MHz
processed size: 32768 complex points
LB: 0.300 GF: 0.0000

SpinWorks 4: no title

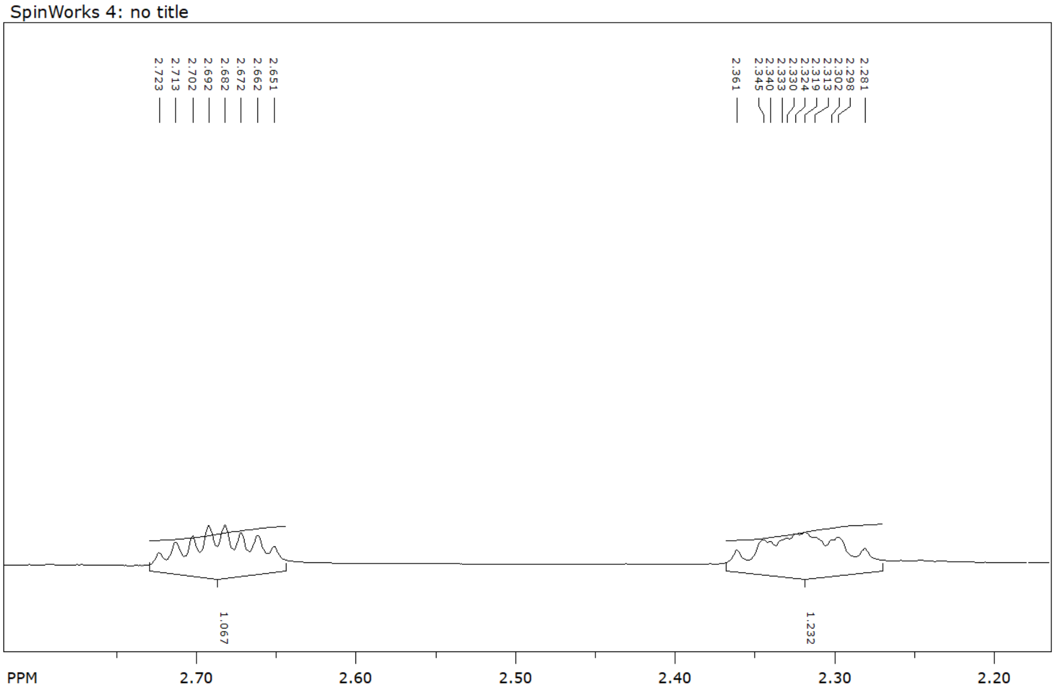

file: ... Łodzi\Desktop\nmr\mfl-2922\10\fid expt: <zg30>
transmitter freq.: 600.263707 MHz
time domain size: 65536 points
width: 12335.53 Hz = 20.5502 ppm = 0.188225 Hz/pt
number of scans: 16

freq. of 0 ppm: 600.260000 MHz
processed size: 32768 complex points
LB: 0.300 GF: 0.0000

Figure S59: ^{31}P NMR Spectrum for *trans*-**16f** in $CDCl_3$

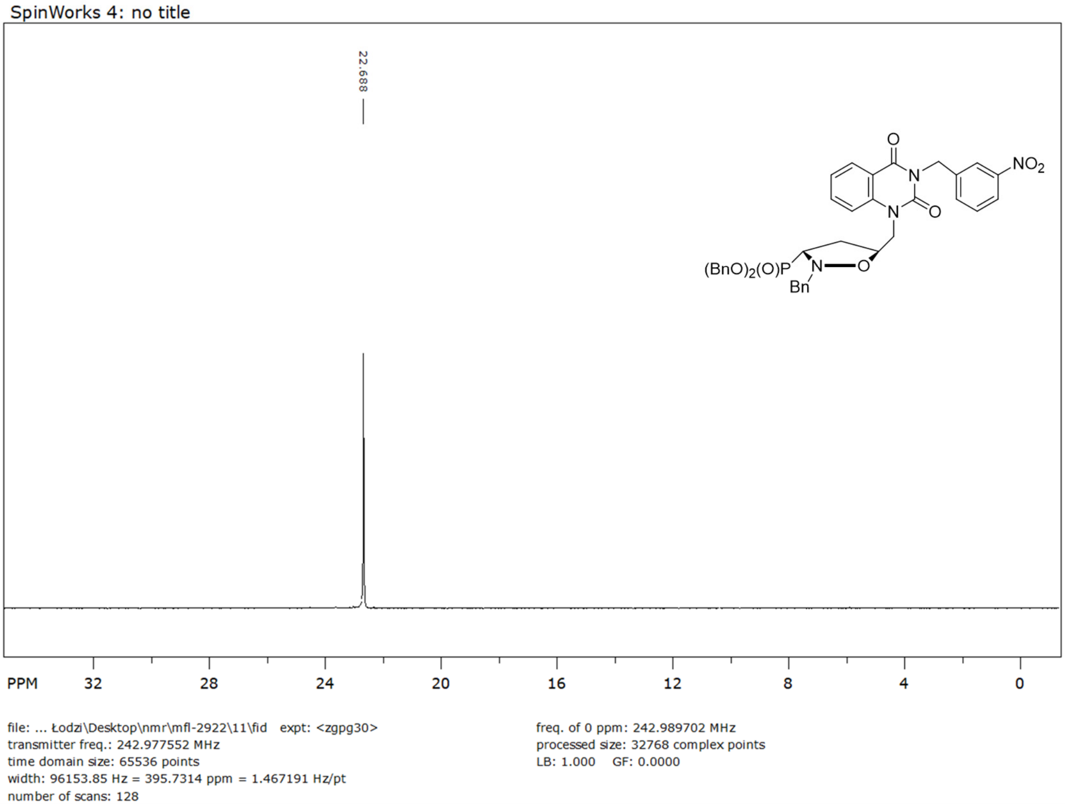

Figure S60: ^{13}C NMR Spectrum for *trans*-**16f** in $CDCl_3$ and expanded spectral regions

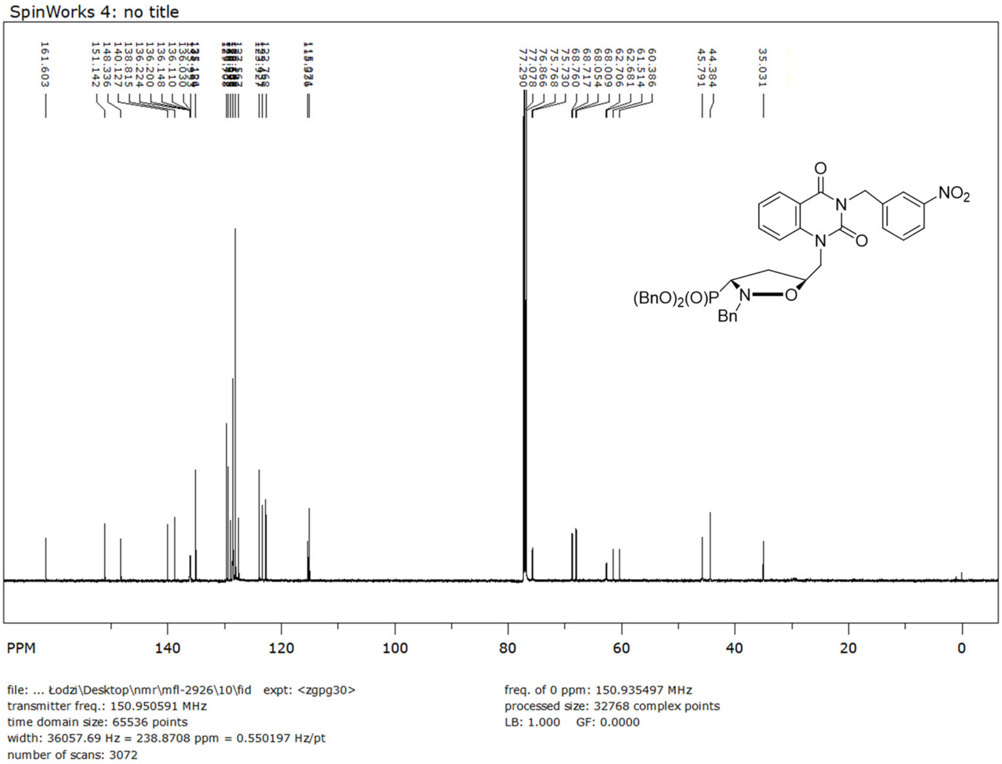

SpinWorks 4: no title

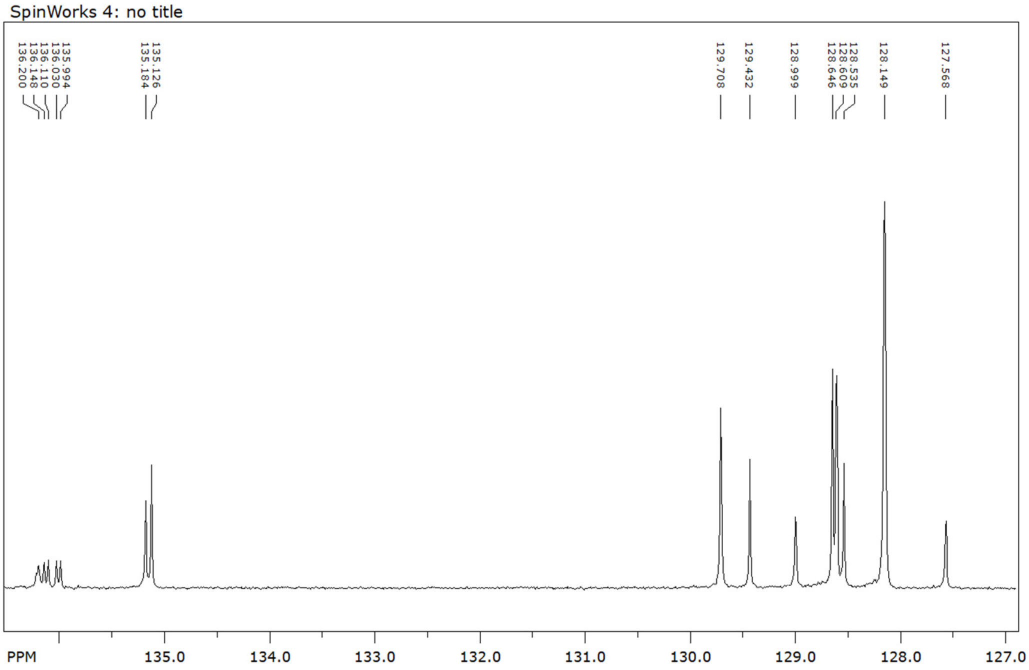

file: ...fl widma\widma 13C\mfl-2926\10\fid   expt: <zgpg30>
transmitter freq.: 150.950591 MHz
time domain size: 65536 points
width: 36057.69 Hz = 238.8708 ppm = 0.550197 Hz/pt
number of scans: 3072

freq. of 0 ppm: 150.935497 MHz
processed size: 32768 complex points
LB: 1.000   GF: 0.0000

SpinWorks 4: no title

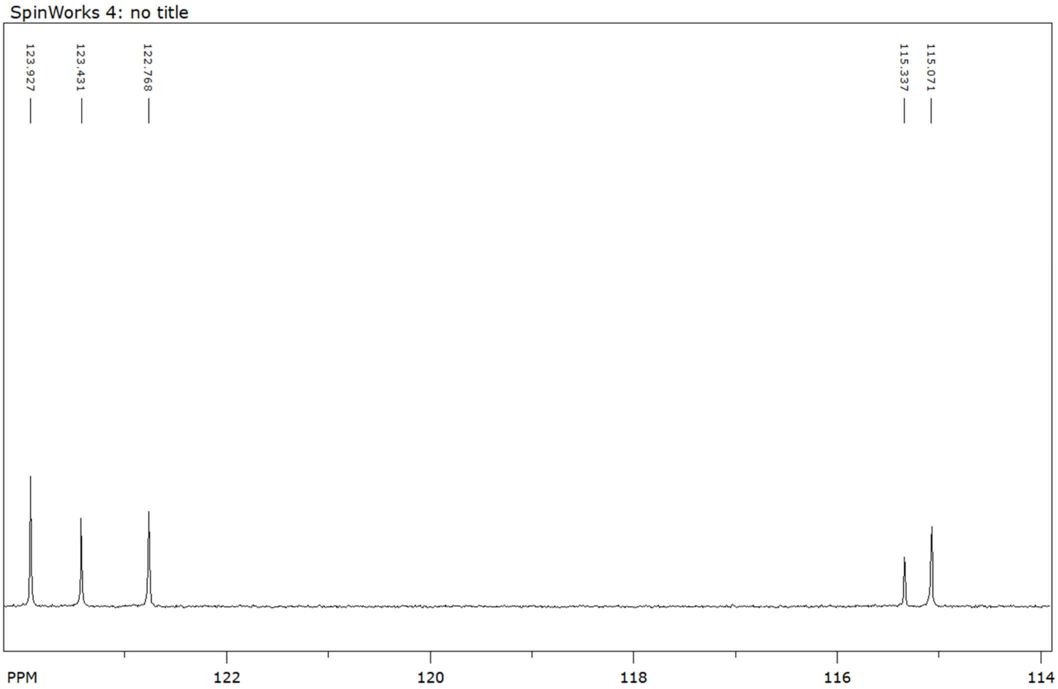

file: ...fl widma\widma 13C\mfl-2926\10\fid   expt: <zgpg30>
transmitter freq.: 150.950591 MHz
time domain size: 65536 points
width: 36057.69 Hz = 238.8708 ppm = 0.550197 Hz/pt
number of scans: 3072

freq. of 0 ppm: 150.935497 MHz
processed size: 32768 complex points
LB: 1.000   GF: 0.0000

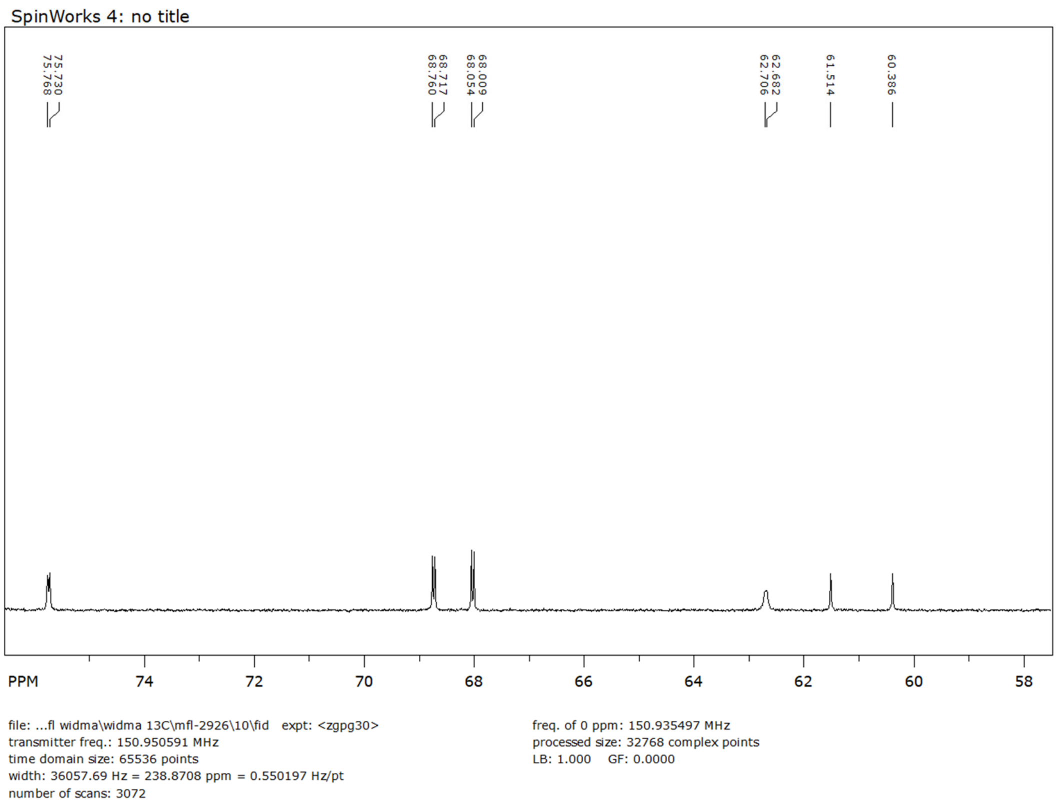

Figure S61: HPLC chromatogram for *trans*-**16f**

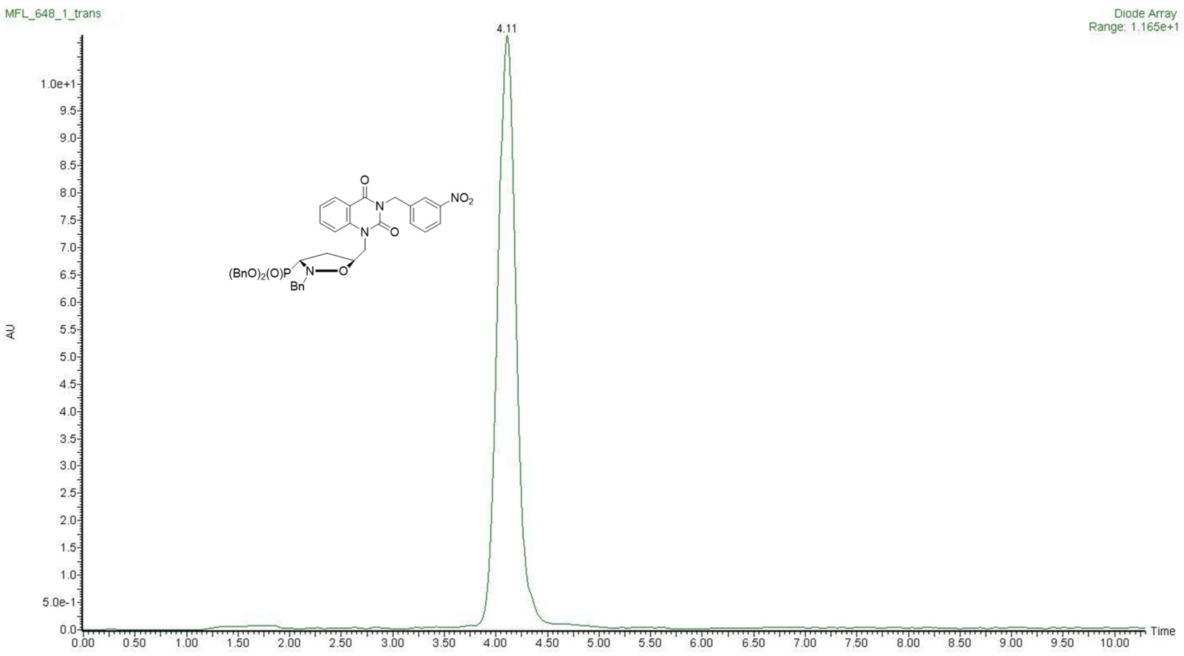

Figure S62: ^{1}H NMR Spectrum for mixture of *cis*-**16g**/*trans*-**16g** (88:12) in $CDCl_3$ and expanded spectral regions

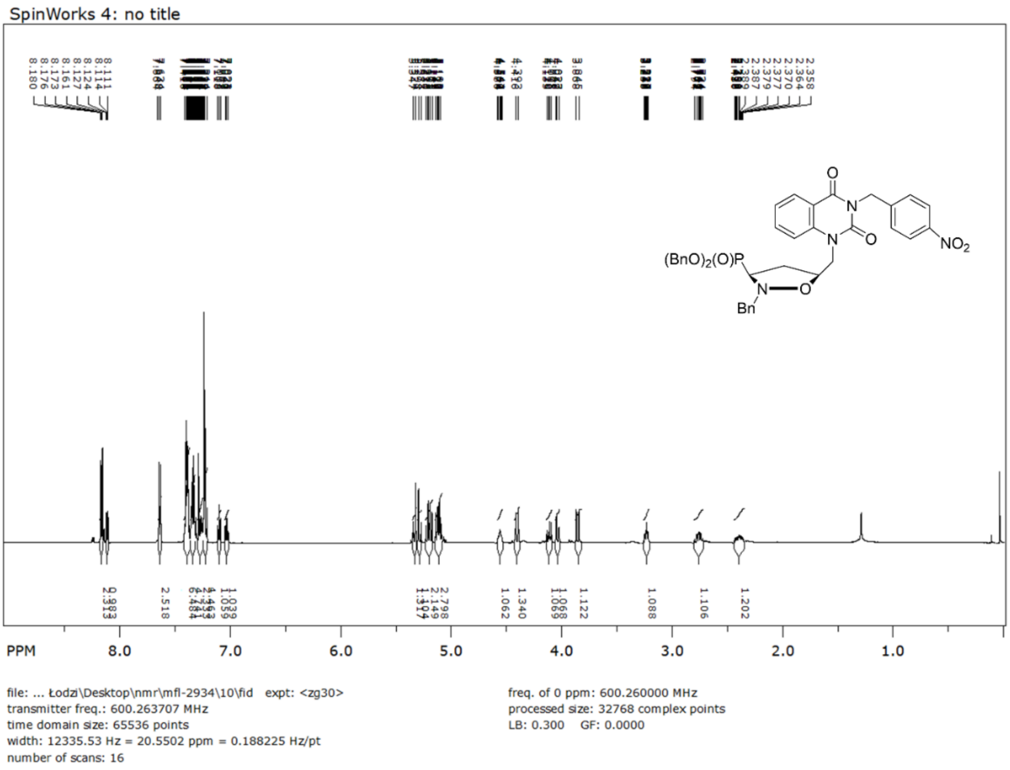

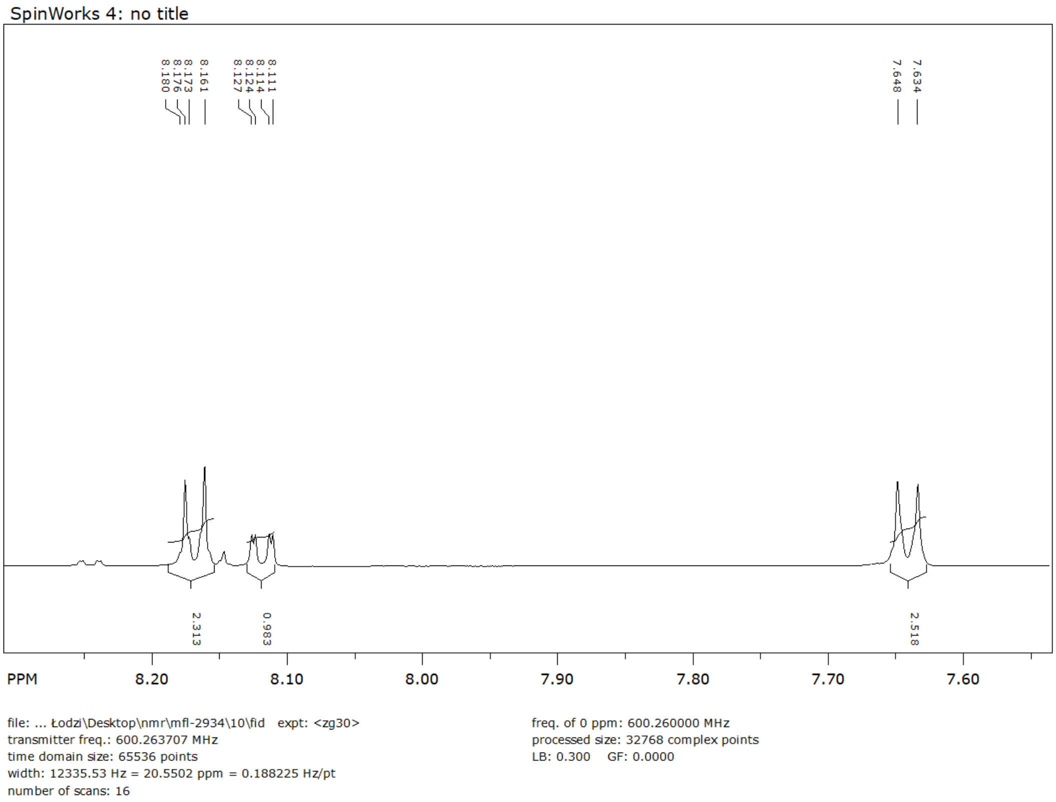

SpinWorks 4: no title

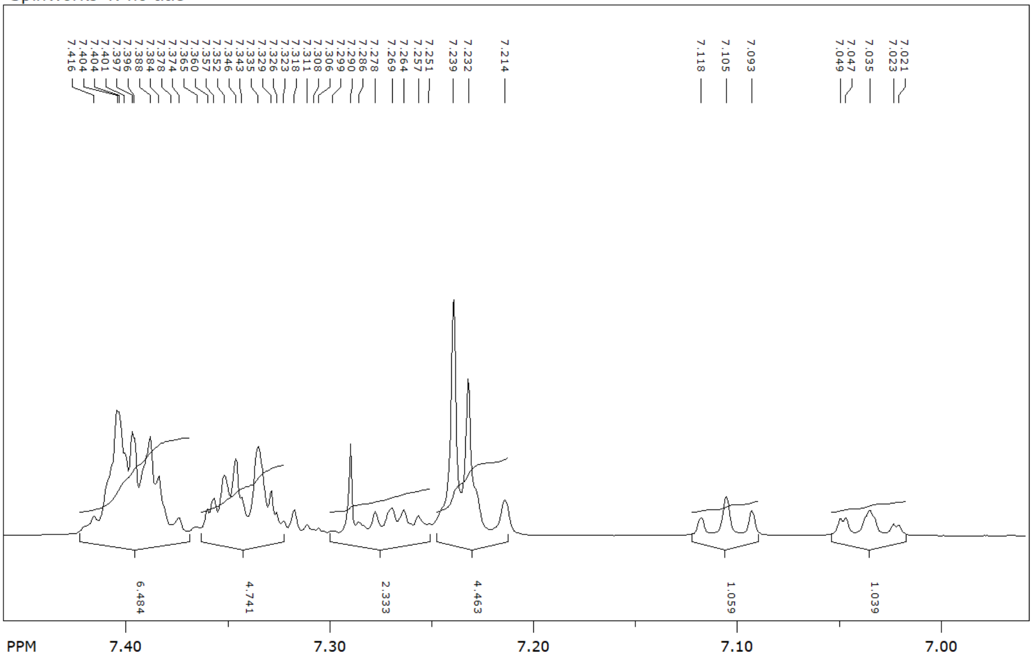

file: ... Łodzi\Desktop\nmr\mfl-2934\10\fid expt: <zg30>
transmitter freq.: 600.263707 MHz
time domain size: 65536 points
width: 12335.53 Hz = 20.5502 ppm = 0.188225 Hz/pt
number of scans: 16

freq. of 0 ppm: 600.260000 MHz
processed size: 32768 complex points
LB: 0.300 GF: 0.0000

SpinWorks 4: no title

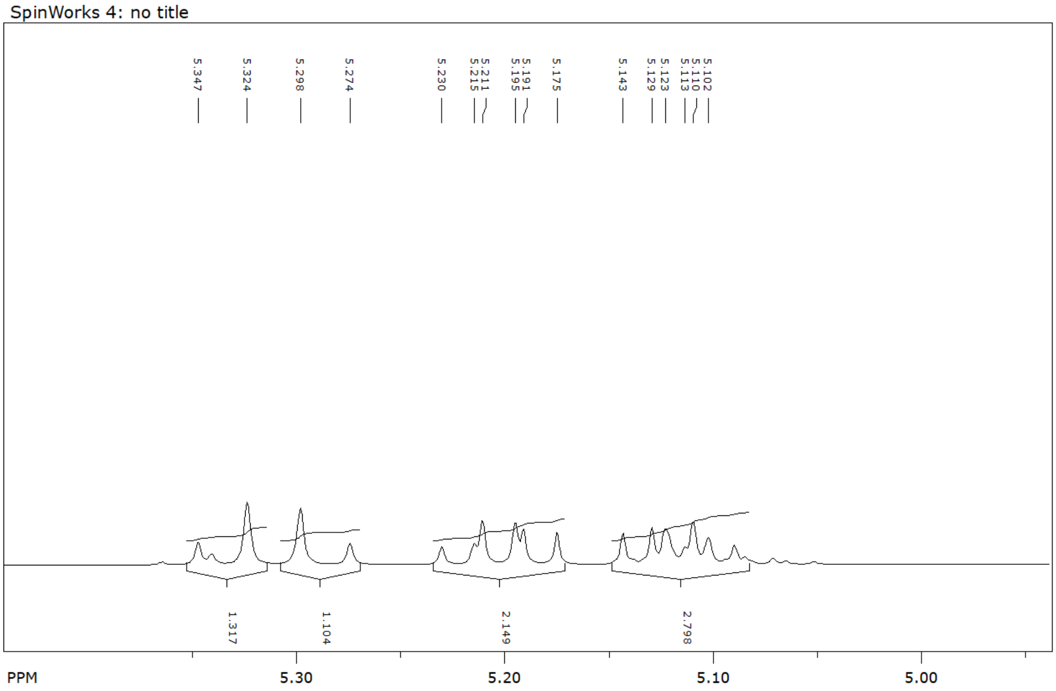

file: ... Łodzi\Desktop\nmr\mfl-2934\10\fid expt: <zg30>
transmitter freq.: 600.263707 MHz
time domain size: 65536 points
width: 12335.53 Hz = 20.5502 ppm = 0.188225 Hz/pt
number of scans: 16

freq. of 0 ppm: 600.260000 MHz
processed size: 32768 complex points
LB: 0.300 GF: 0.0000

SpinWorks 4: no title

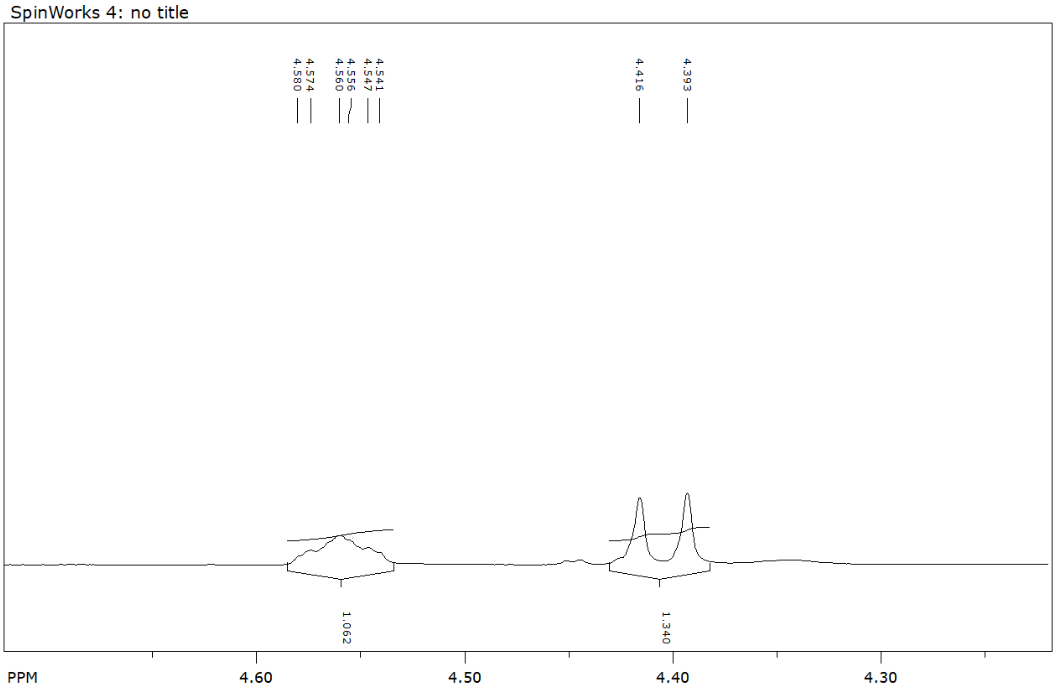

file: ... Łodzi\Desktop\nmr\mfl-2934\10\fid expt: <zg30>
transmitter freq.: 600.263707 MHz
time domain size: 65536 points
width: 12335.53 Hz = 20.5502 ppm = 0.188225 Hz/pt
number of scans: 16

freq. of 0 ppm: 600.260000 MHz
processed size: 32768 complex points
LB: 0.300 GF: 0.0000

SpinWorks 4: no title

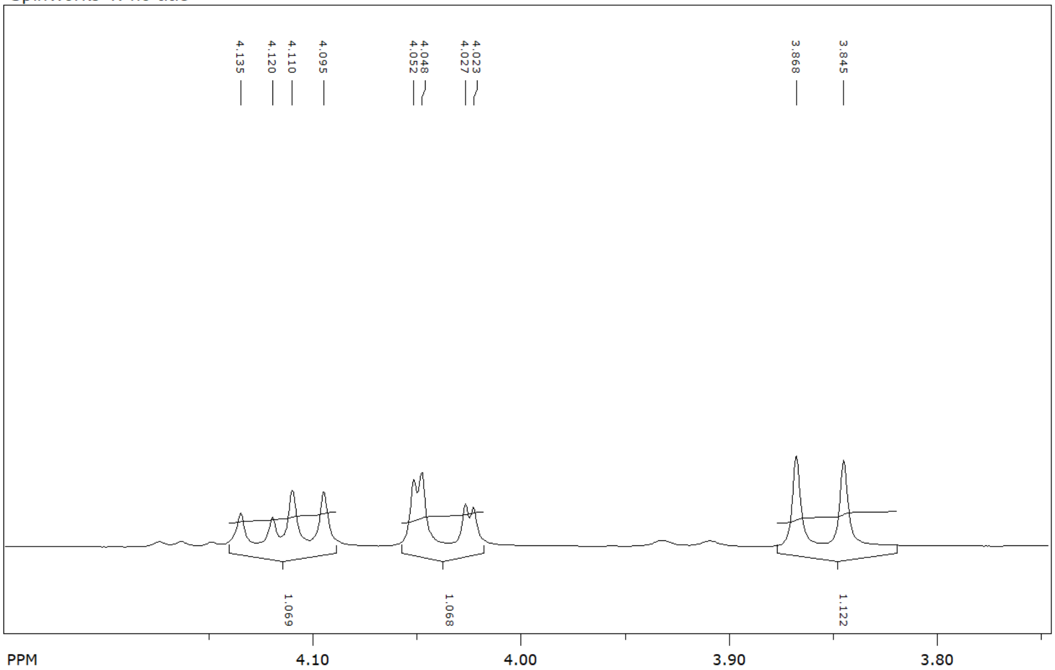

file: ... Łodzi\Desktop\nmr\mfl-2934\10\fid expt: <zg30>
transmitter freq.: 600.263707 MHz
time domain size: 65536 points
width: 12335.53 Hz = 20.5502 ppm = 0.188225 Hz/pt
number of scans: 16

freq. of 0 ppm: 600.260000 MHz
processed size: 32768 complex points
LB: 0.300 GF: 0.0000

SpinWorks 4: no title

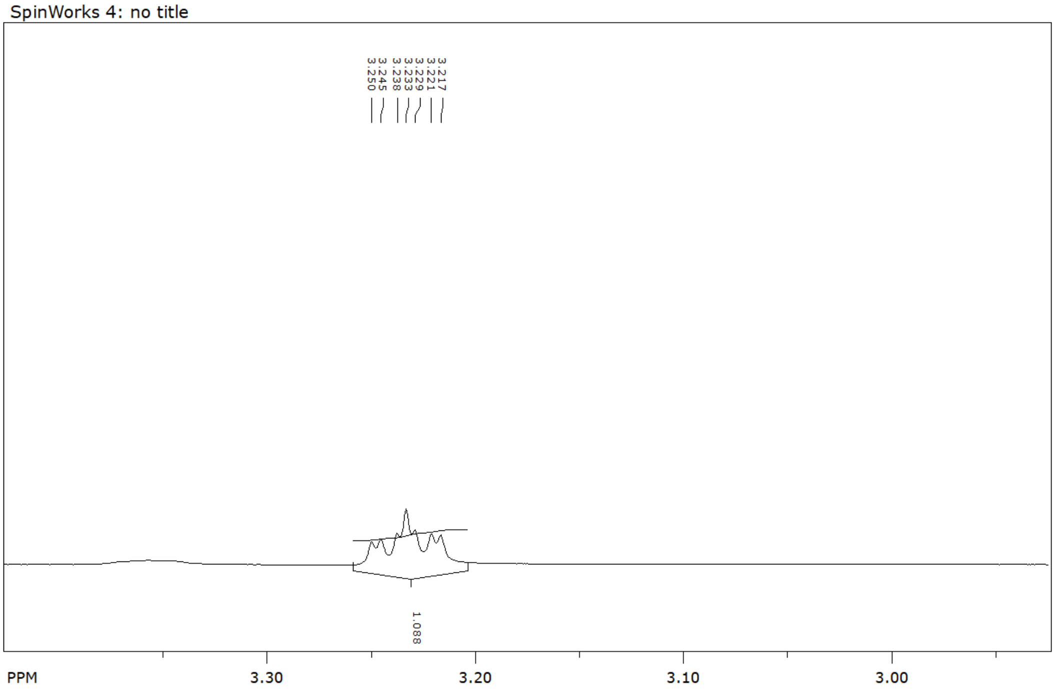

file: ... Łodzi\Desktop\nmr\mfl-2934\10\fid expt: <zg30>
transmitter freq.: 600.263707 MHz
time domain size: 65536 points
width: 12335.53 Hz = 20.5502 ppm = 0.188225 Hz/pt
number of scans: 16

freq. of 0 ppm: 600.260000 MHz
processed size: 32768 complex points
LB: 0.300 GF: 0.0000

SpinWorks 4: no title

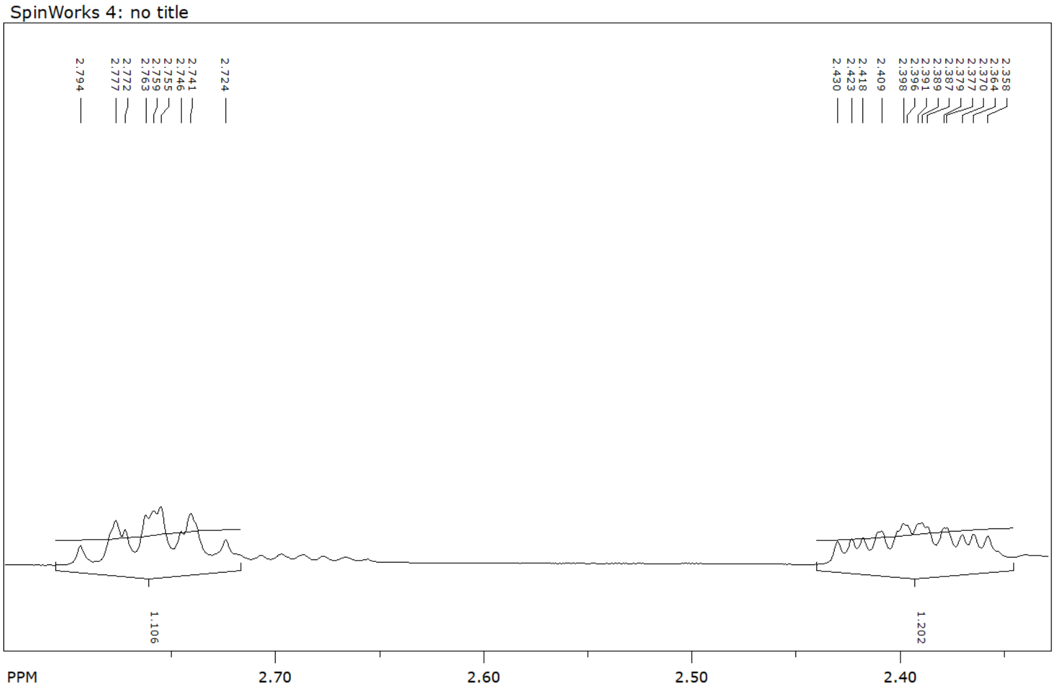

file: ... Łodzi\Desktop\nmr\mfl-2934\10\fid expt: <zg30>
transmitter freq.: 600.263707 MHz
time domain size: 65536 points
width: 12335.53 Hz = 20.5502 ppm = 0.188225 Hz/pt
number of scans: 16

freq. of 0 ppm: 600.260000 MHz
processed size: 32768 complex points
LB: 0.300 GF: 0.0000

Figure S63: ^{31}P NMR Spectrum for mixture of *cis*-**16g**/*trans*-**16g** (88:12) in $CDCl_3$

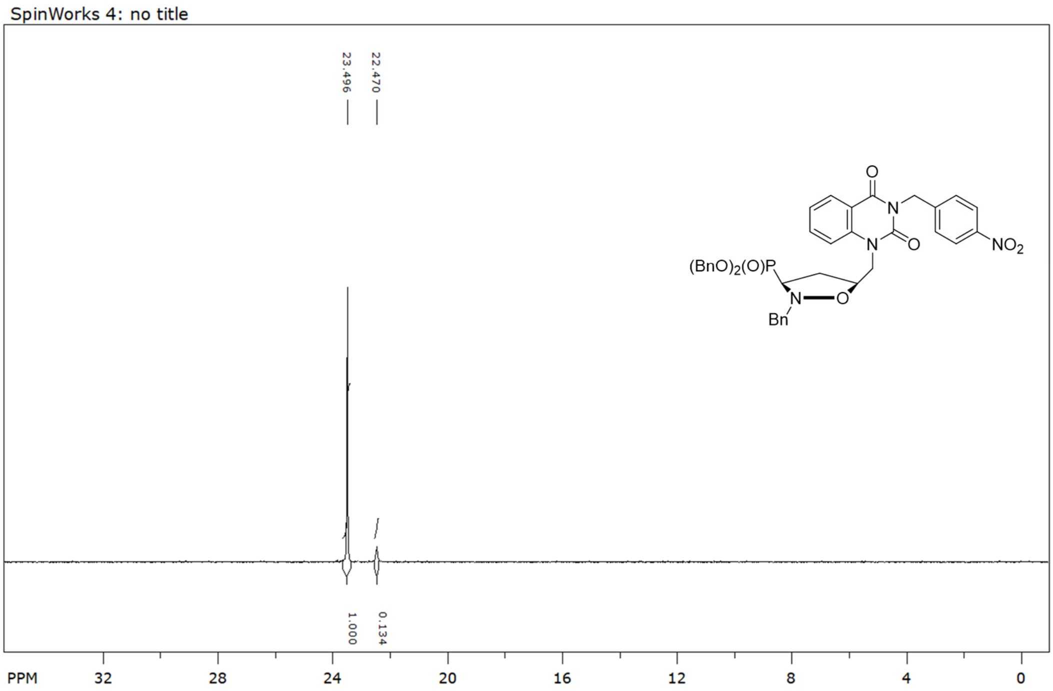

Figure S64: ^{13}C NMR Spectrum for mixture of *cis*-**16g**/*trans*-**16g** (88:12) in $CDCl_3$ and expanded spectral regions

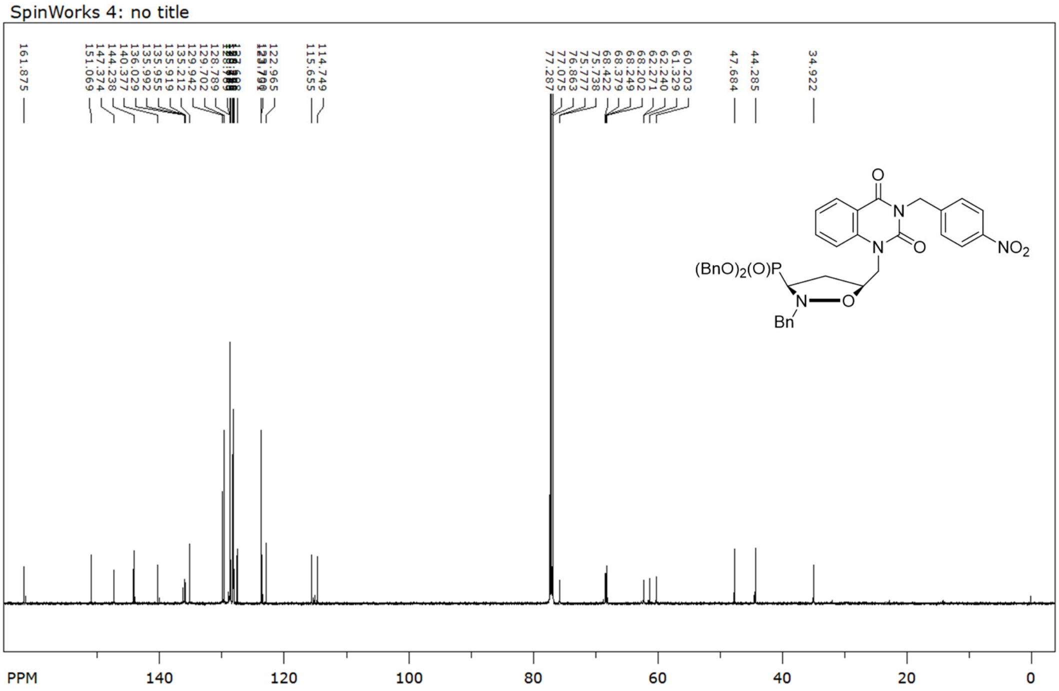

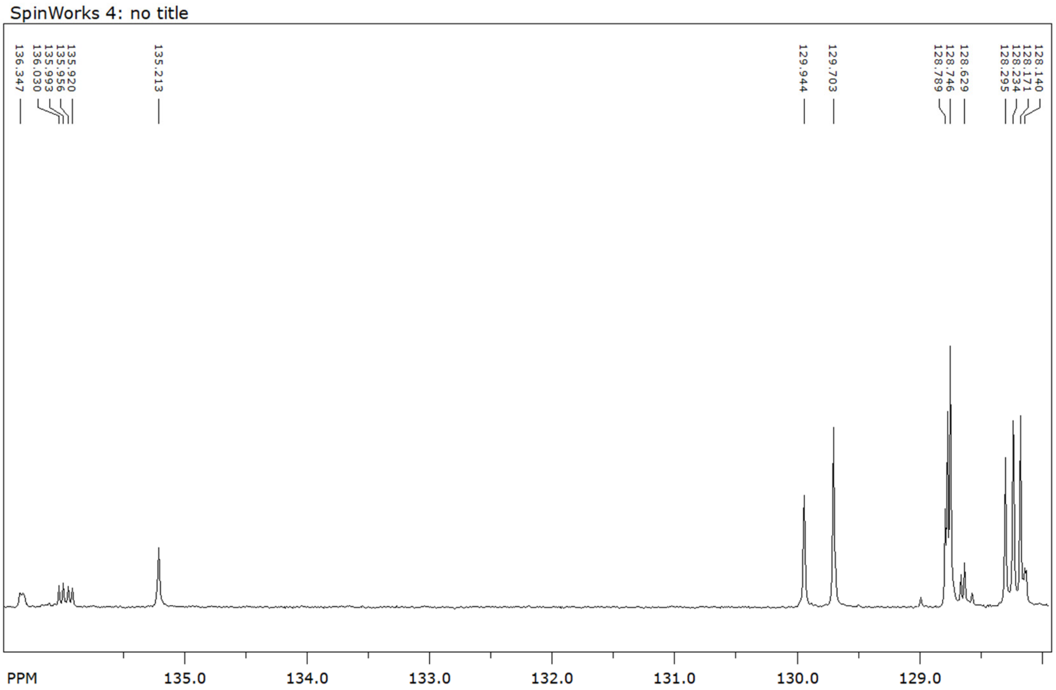

file: ...fl widma\widma 13C\mfl-2937\10\fid expt: <zgpg30>
transmitter freq.: 150.950591 MHz
time domain size: 65536 points
width: 36057.69 Hz = 238.8708 ppm = 0.550197 Hz/pt
number of scans: 3072

freq. of 0 ppm: 150.935497 MHz
processed size: 32768 complex points
LB: 1.000 GF: 0.0000

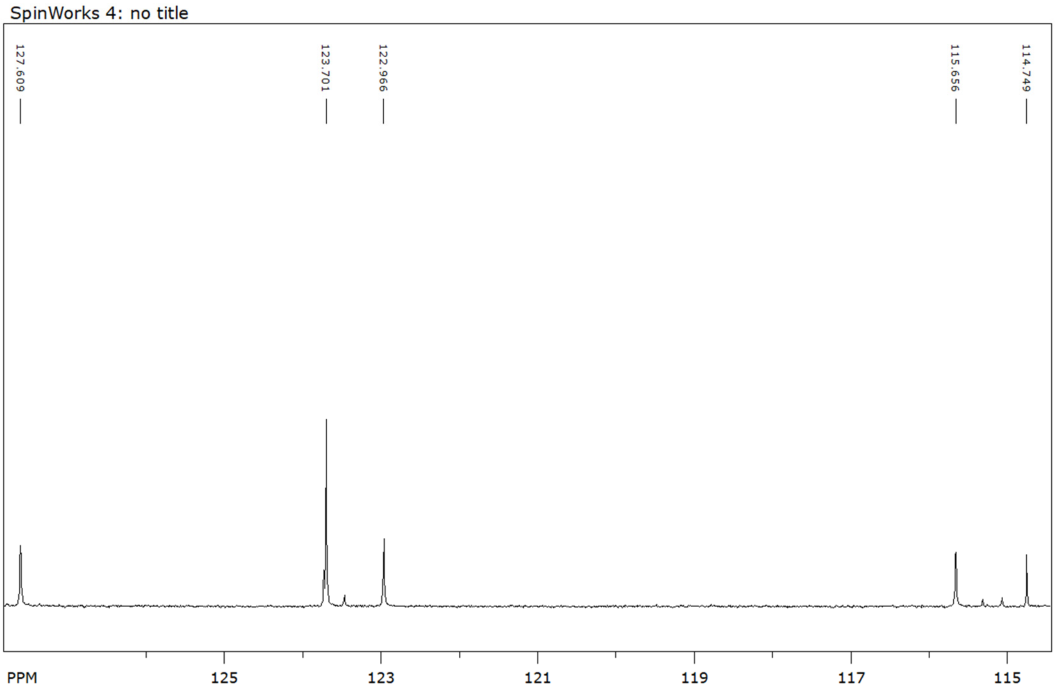

file: ...fl widma\widma 13C\mfl-2937\10\fid expt: <zgpg30>
transmitter freq.: 150.950591 MHz
time domain size: 65536 points
width: 36057.69 Hz = 238.8708 ppm = 0.550197 Hz/pt
number of scans: 3072

freq. of 0 ppm: 150.935497 MHz
processed size: 32768 complex points
LB: 1.000 GF: 0.0000

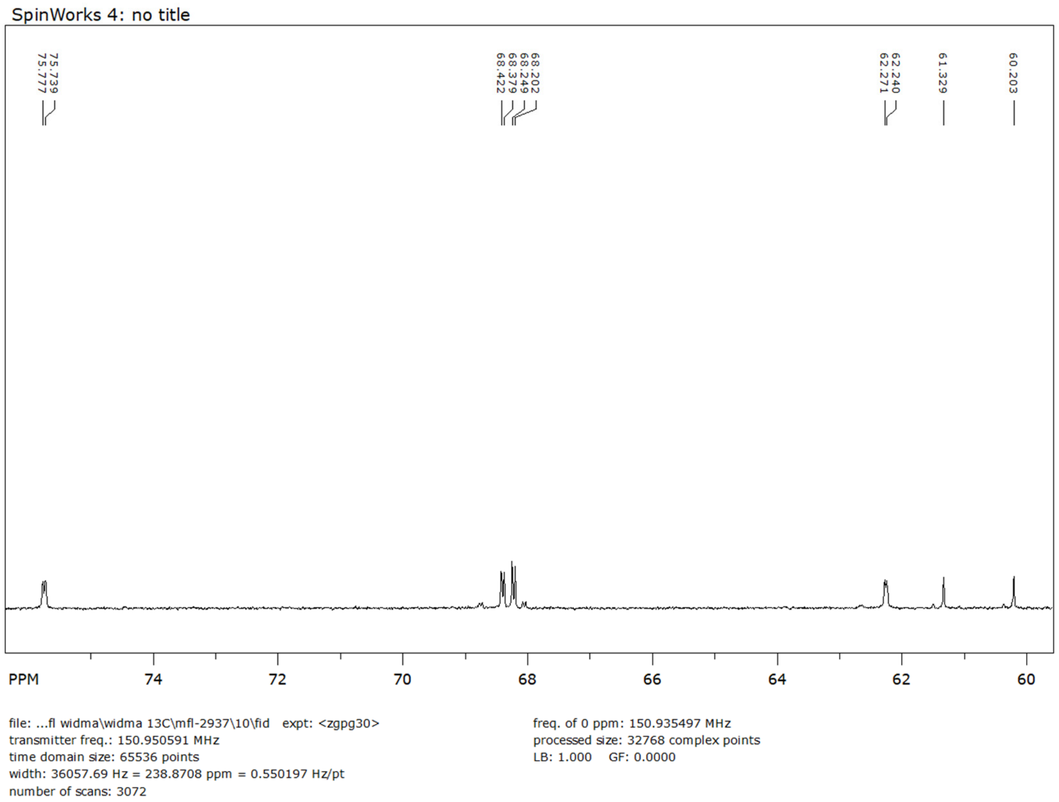

Figure S65: HPLC chromatogram for mixture of *cis*-**16g**/*trans*-**16g** (88:12)

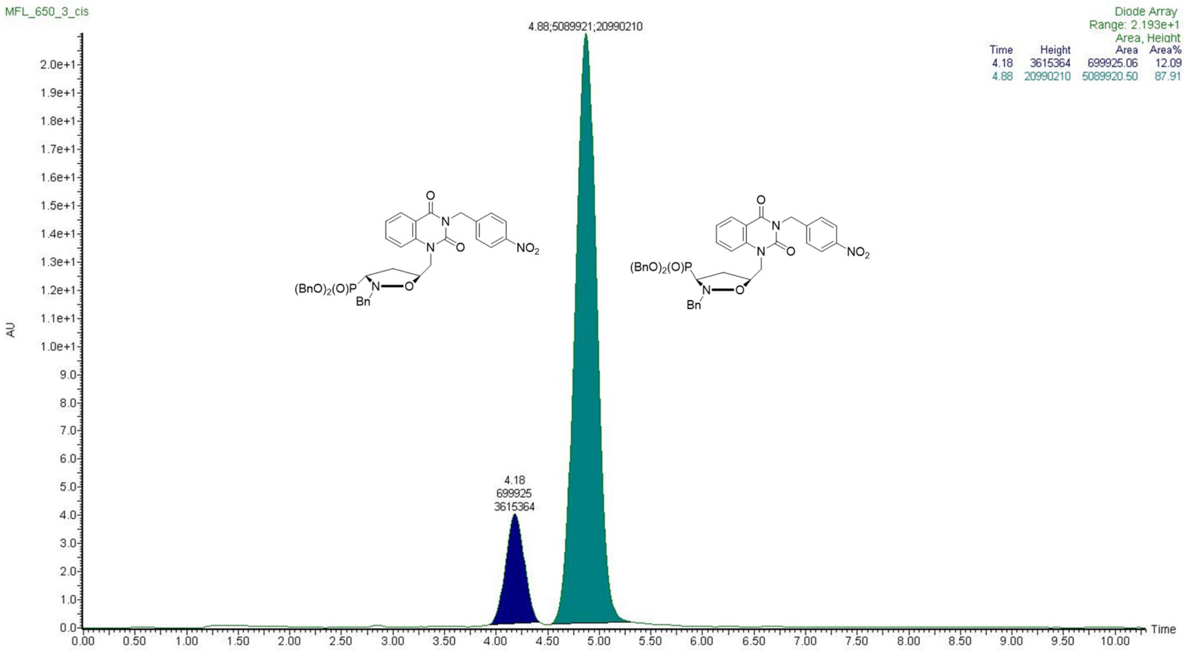

Figure S66: ^{1}H NMR Spectrum for *trans*-**16g** in $CDCl_3$ and expanded spectral regions

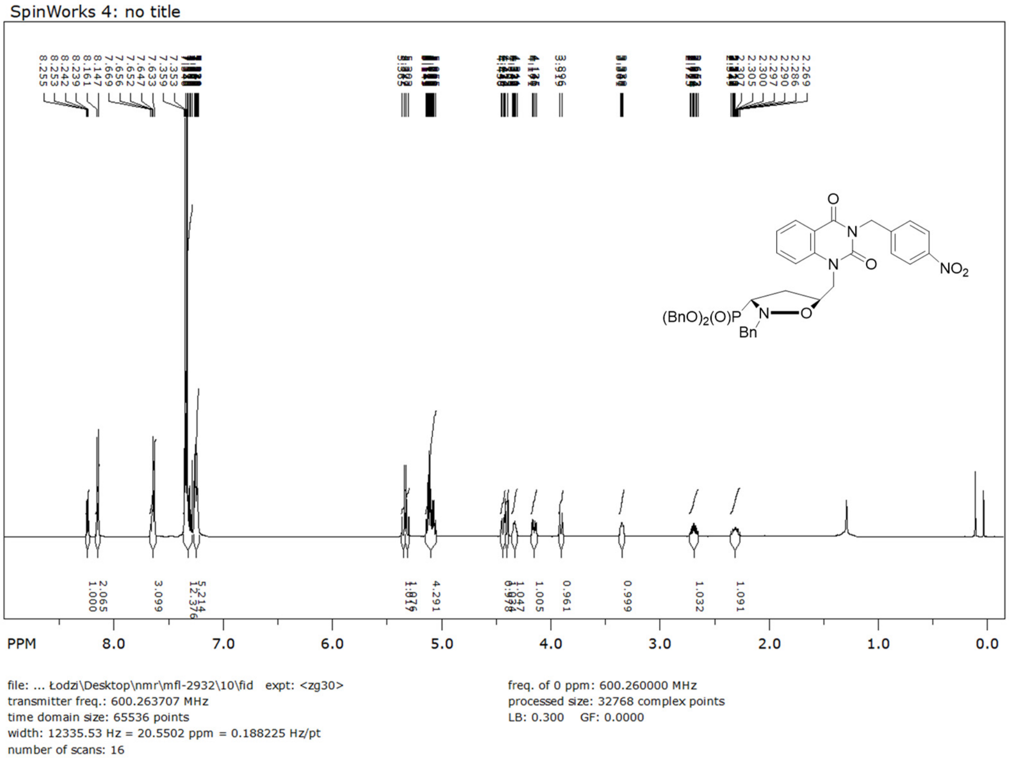

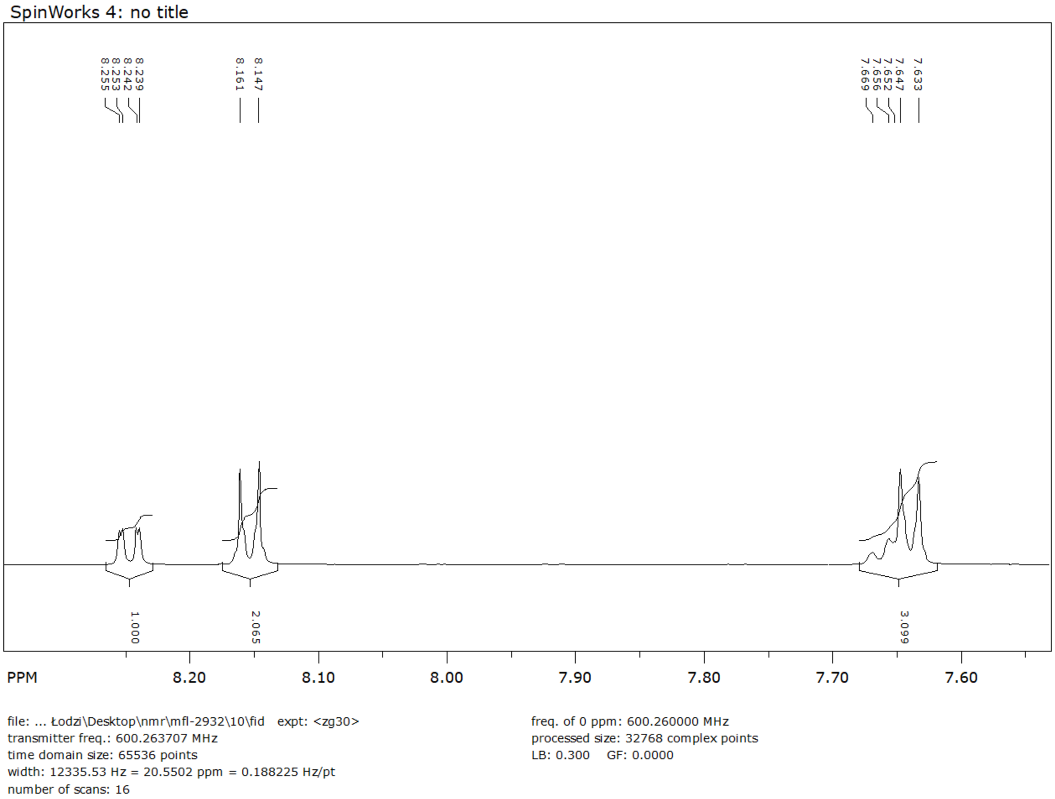

SpinWorks 4: no title

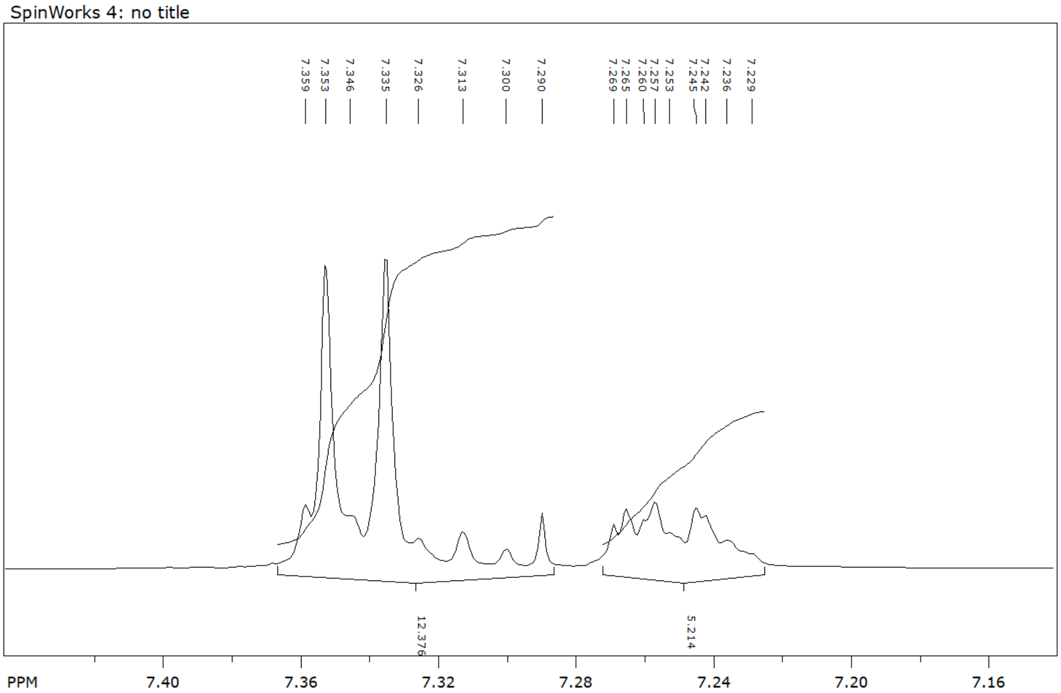

file: ... Łodzi\Desktop\nmr\mfl-2932\10\fid expt: <zg30>
transmitter freq.: 600.263707 MHz
time domain size: 65536 points
width: 12335.53 Hz = 20.5502 ppm = 0.188225 Hz/pt
number of scans: 16

freq. of 0 ppm: 600.260000 MHz
processed size: 32768 complex points
LB: 0.300 GF: 0.0000

SpinWorks 4: no title

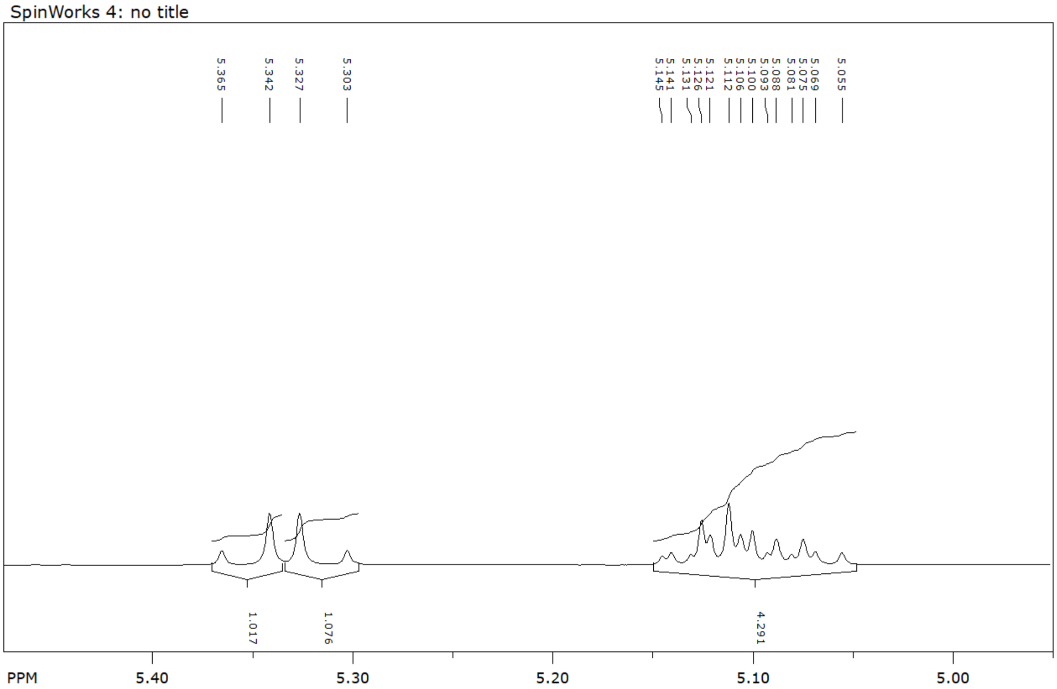

file: ... Łodzi\Desktop\nmr\mfl-2932\10\fid expt: <zg30>
transmitter freq.: 600.263707 MHz
time domain size: 65536 points
width: 12335.53 Hz = 20.5502 ppm = 0.188225 Hz/pt
number of scans: 16

freq. of 0 ppm: 600.260000 MHz
processed size: 32768 complex points
LB: 0.300 GF: 0.0000

SpinWorks 4: no title

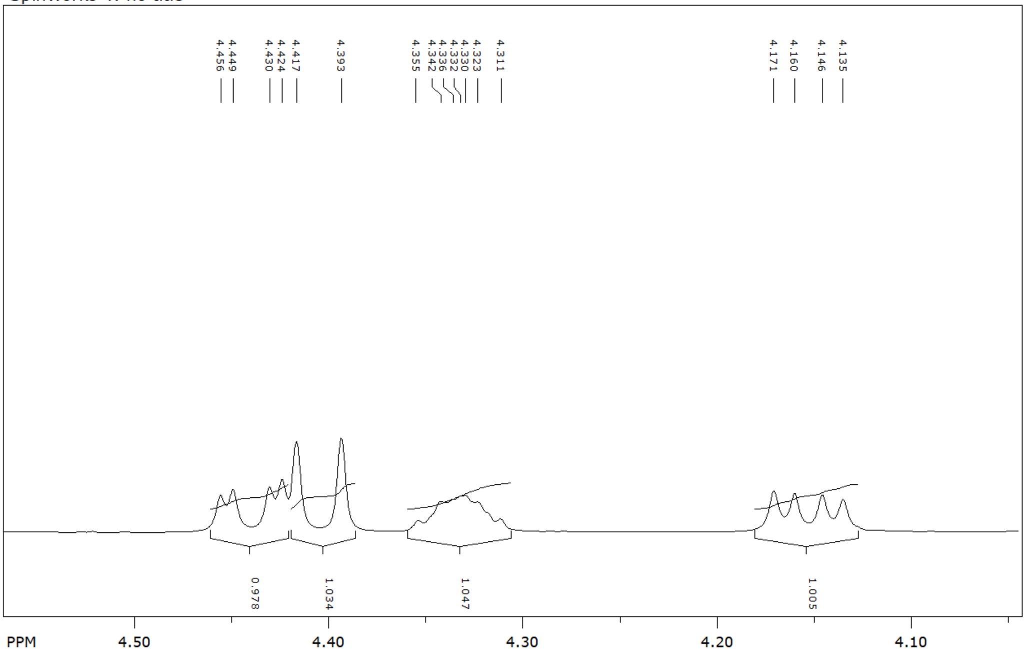

file: ... Łodzi\Desktop\nmr\mfl-2932\10\fid expt: <zg30>
transmitter freq.: 600.263707 MHz
time domain size: 65536 points
width: 12335.53 Hz = 20.5502 ppm = 0.188225 Hz/pt
number of scans: 16

freq. of 0 ppm: 600.260000 MHz
processed size: 32768 complex points
LB: 0.300 GF: 0.0000

SpinWorks 4: no title

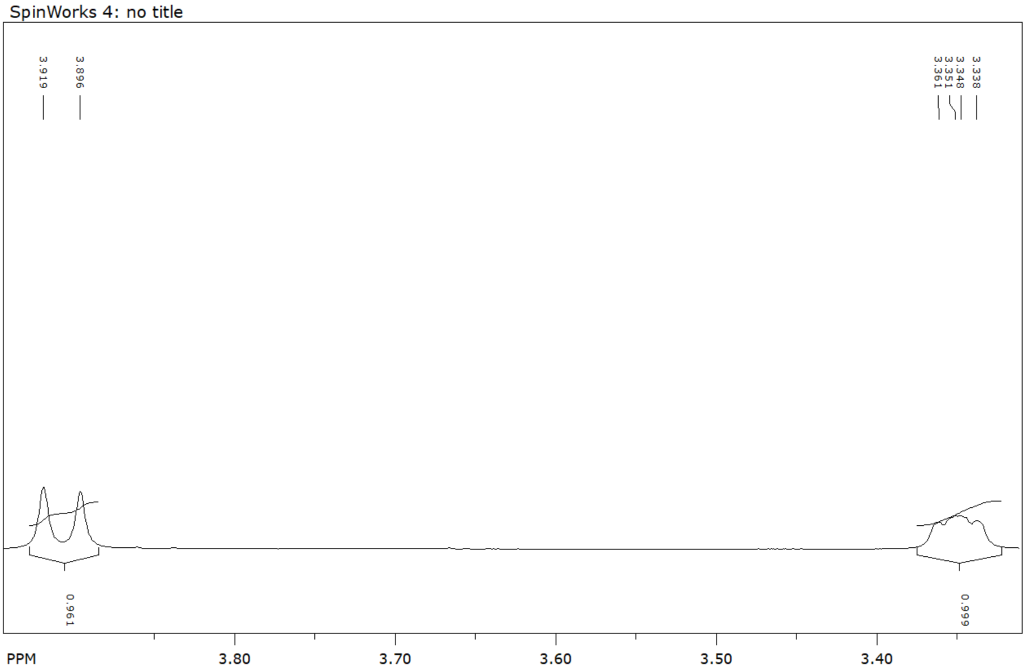

file: ... Łodzi\Desktop\nmr\mfl-2932\10\fid expt: <zg30>
transmitter freq.: 600.263707 MHz
time domain size: 65536 points
width: 12335.53 Hz = 20.5502 ppm = 0.188225 Hz/pt
number of scans: 16

freq. of 0 ppm: 600.260000 MHz
processed size: 32768 complex points
LB: 0.300 GF: 0.0000

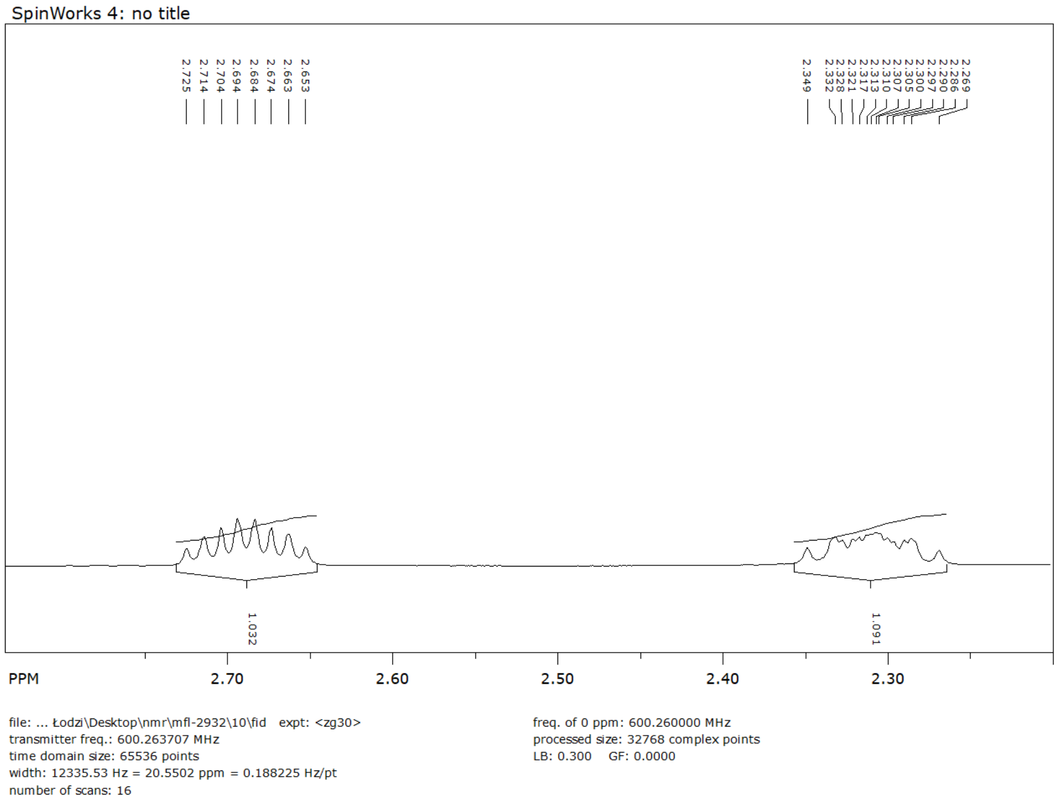

Figure S67: ^{31}P NMR Spectrum for *trans*-**16g** in $CDCl_3$

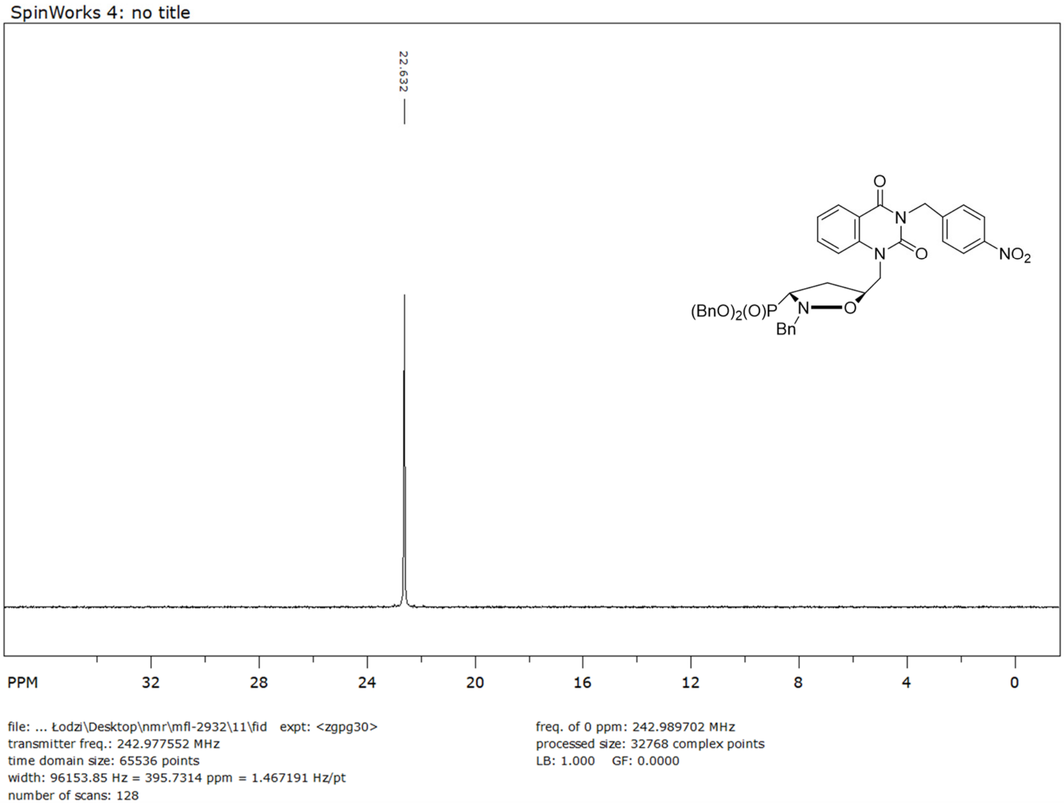

Figure S68: ^{13}C NMR Spectrum for *trans*-**16g** in $CDCl_3$ and expanded spectral regions

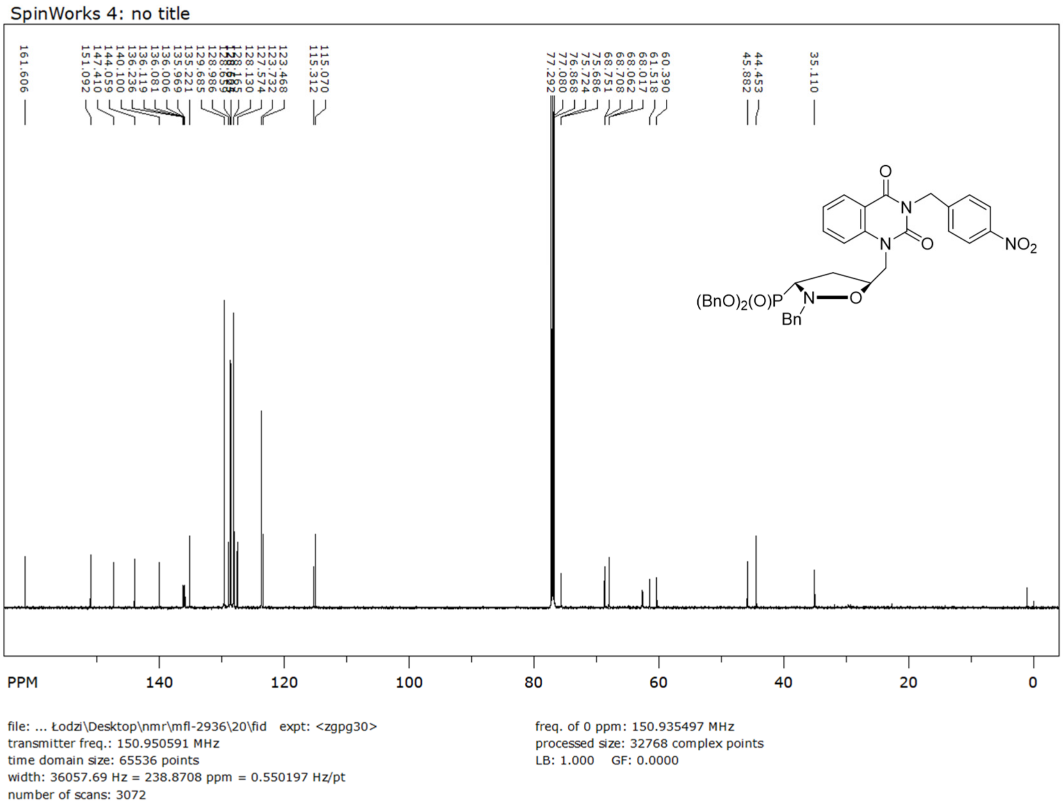

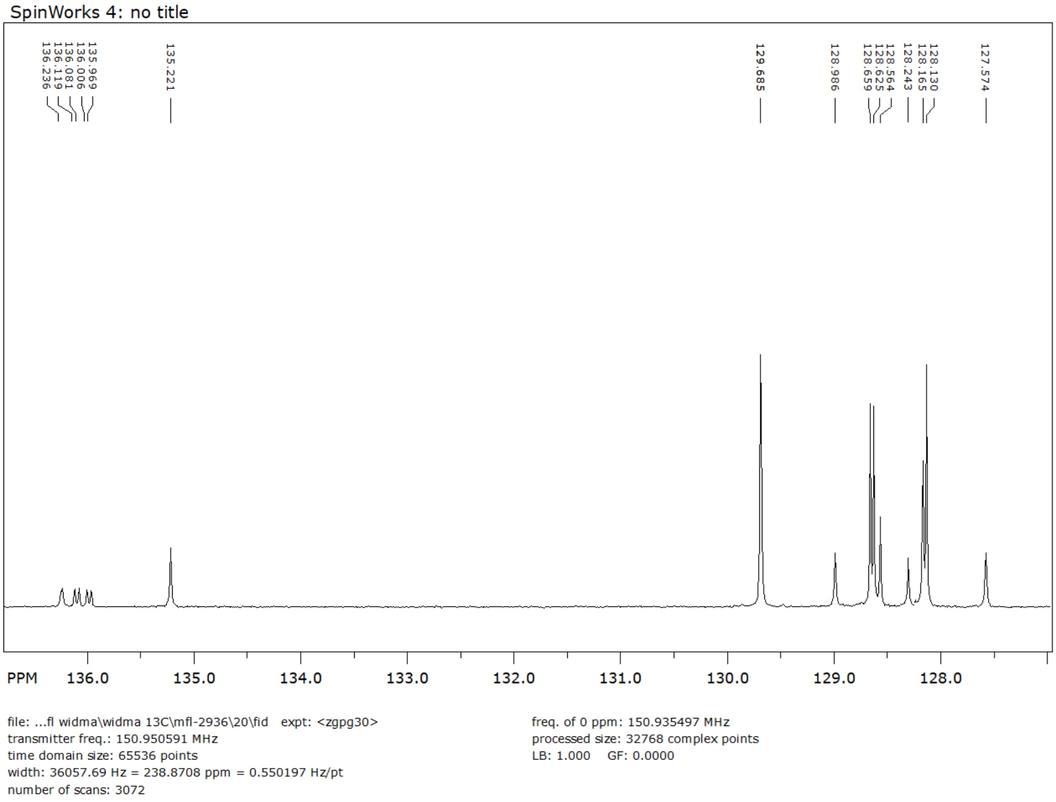

SpinWorks 4: no title

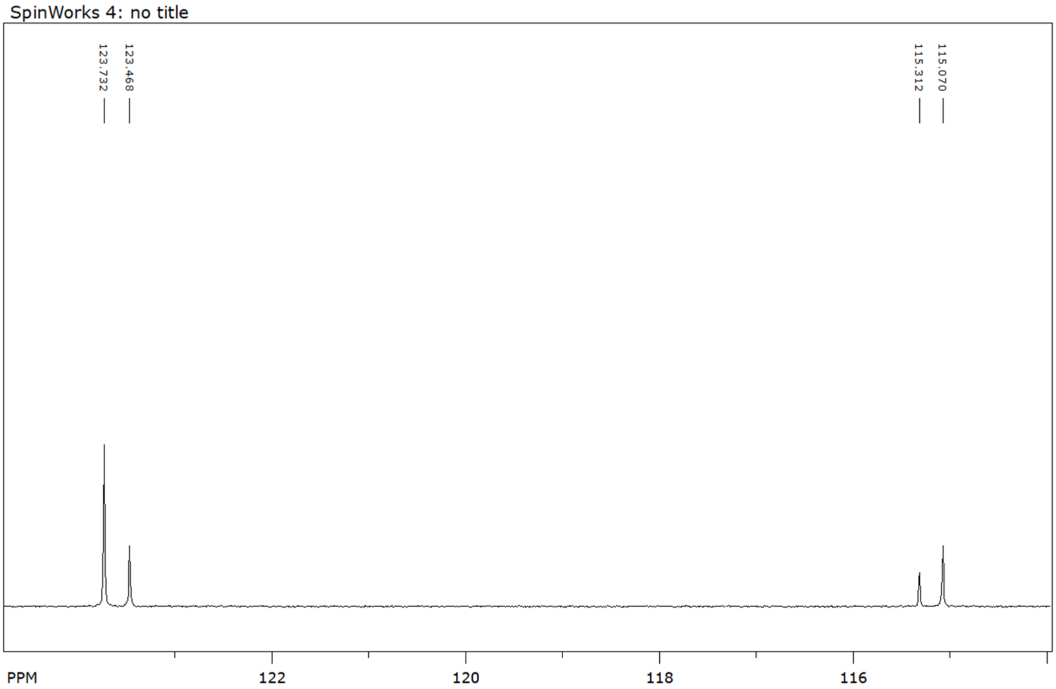

file: ...fl widma\widma 13C\mfl-2936\20\fid expt: <zgpg30>
transmitter freq.: 150.950591 MHz
time domain size: 65536 points
width: 36057.69 Hz = 238.8708 ppm = 0.550197 Hz/pt
number of scans: 3072

freq. of 0 ppm: 150.935497 MHz
processed size: 32768 complex points
LB: 1.000 GF: 0.0000

SpinWorks 4: no title

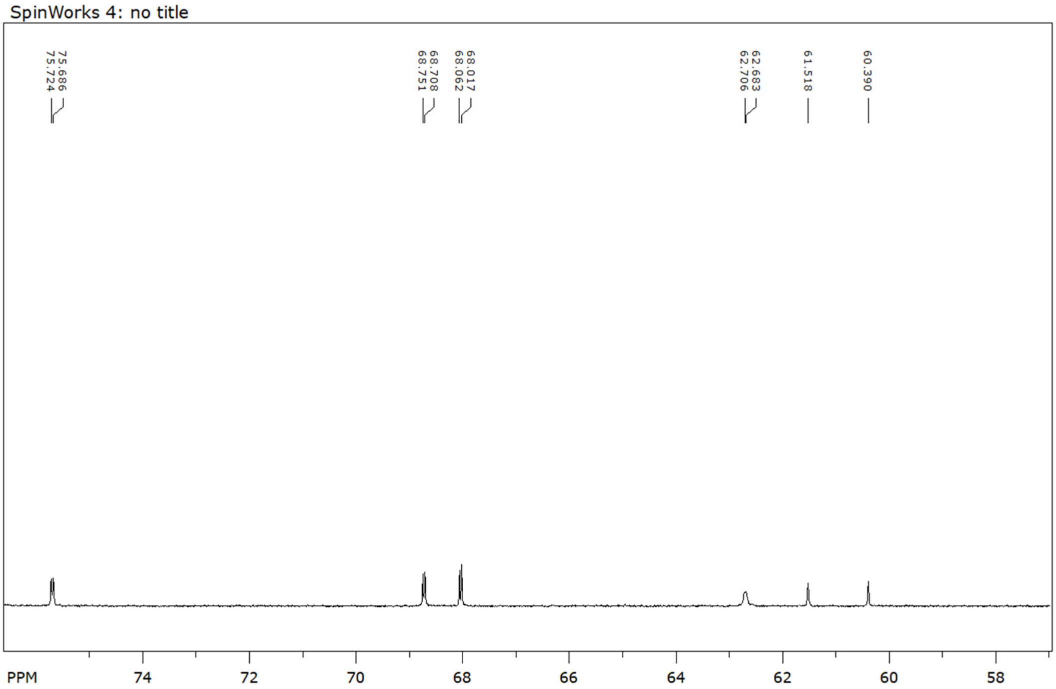

file: ...fl widma\widma 13C\mfl-2936\20\fid expt: <zgpg30>
transmitter freq.: 150.950591 MHz
time domain size: 65536 points
width: 36057.69 Hz = 238.8708 ppm = 0.550197 Hz/pt
number of scans: 3072

freq. of 0 ppm: 150.935497 MHz
processed size: 32768 complex points
LB: 1.000 GF: 0.0000

Figure S69: HPLC chromatogram for *trans*-**16g**

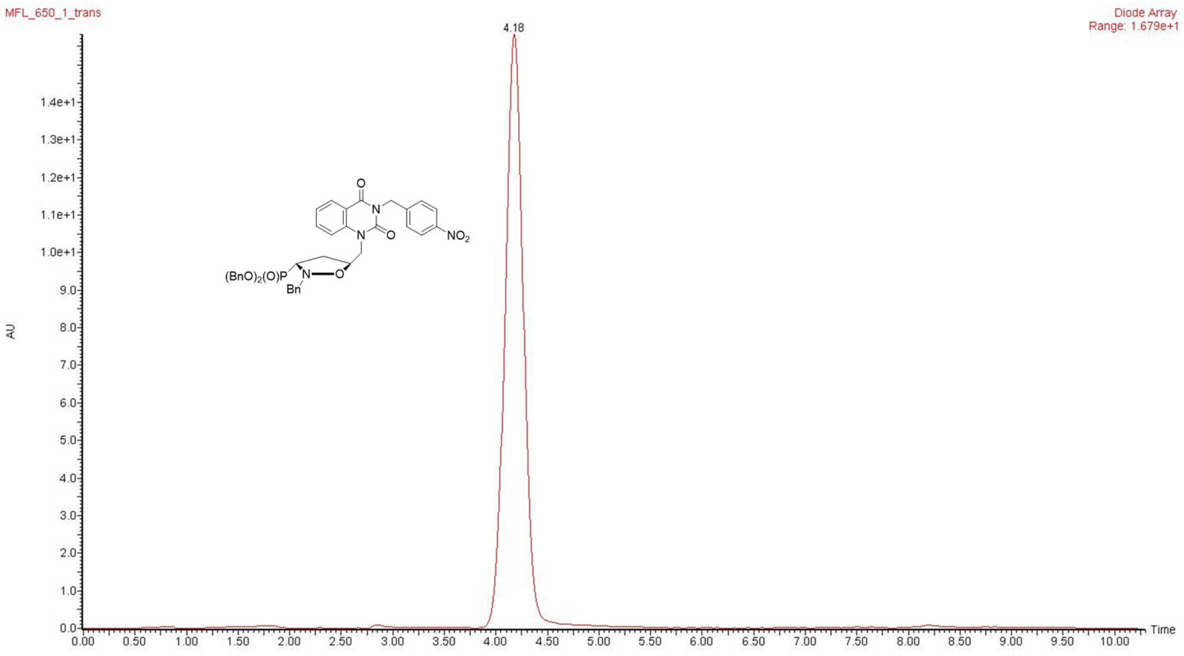

Figure S70: ^{1}H–^{1}H COSY Spectrum for mixture of *cis*-**16a**/*trans*-**16a** (97:3) in $CDCl_3$

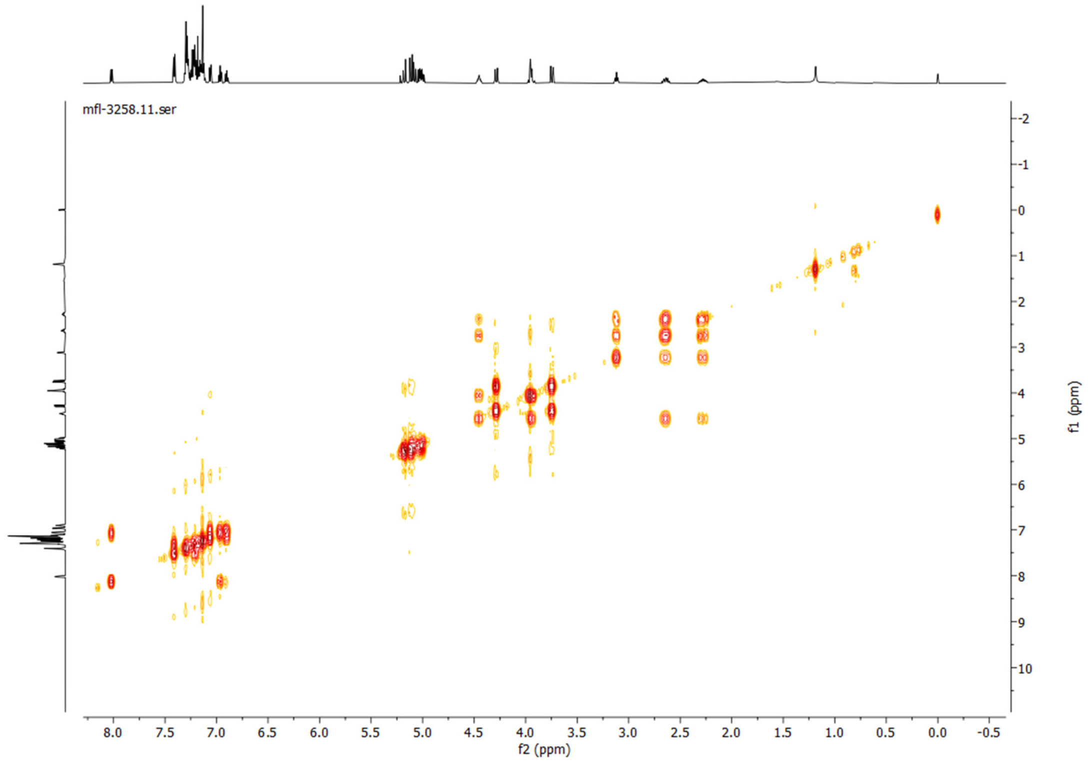

Figure S71: NOESY Spectrum for mixture of *cis*-**16a**/*trans*-**16a** (97:3) in CDCl3

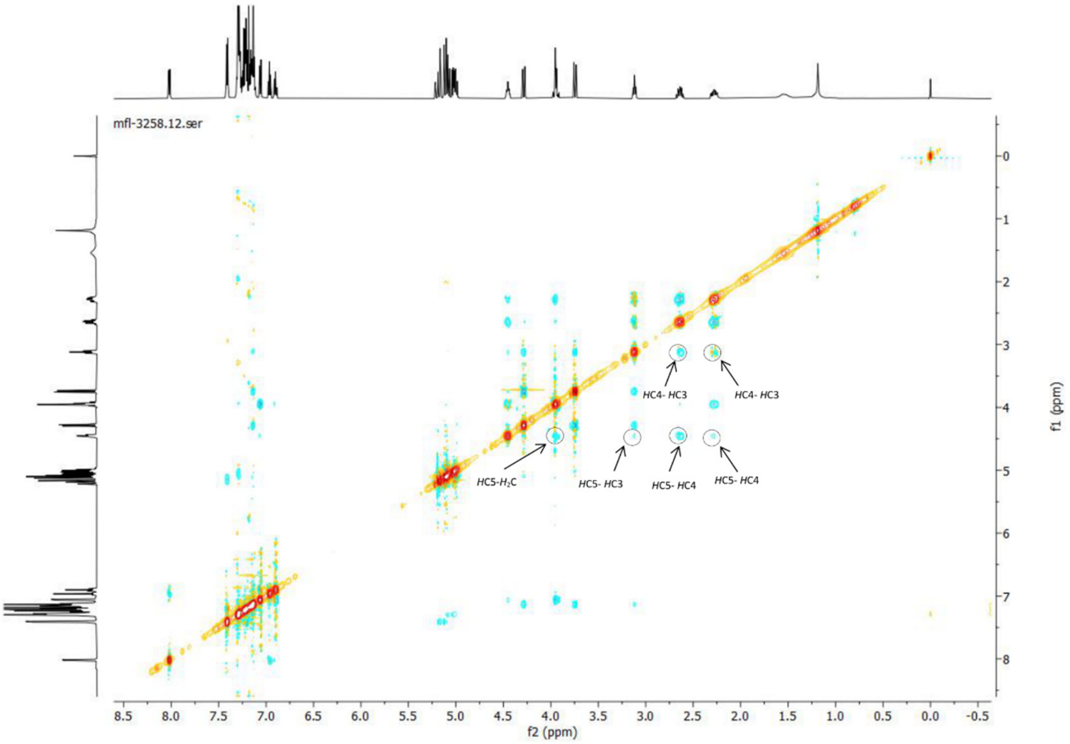

Figure S72: ^{1}H–^{1}H COSY Spectrum for mixture of *trans*-**16a** in CDCl3

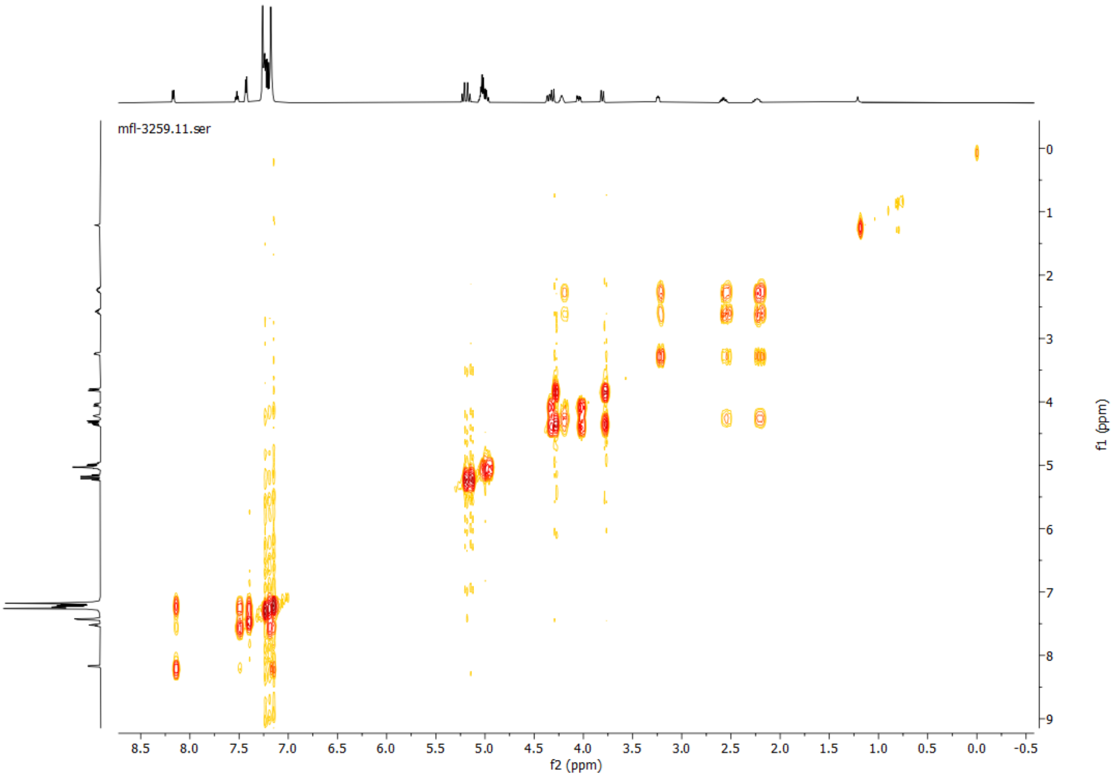

Figure S73: NOESY Spectrum for mixture of *trans*-**16a** in $CDCl_3$

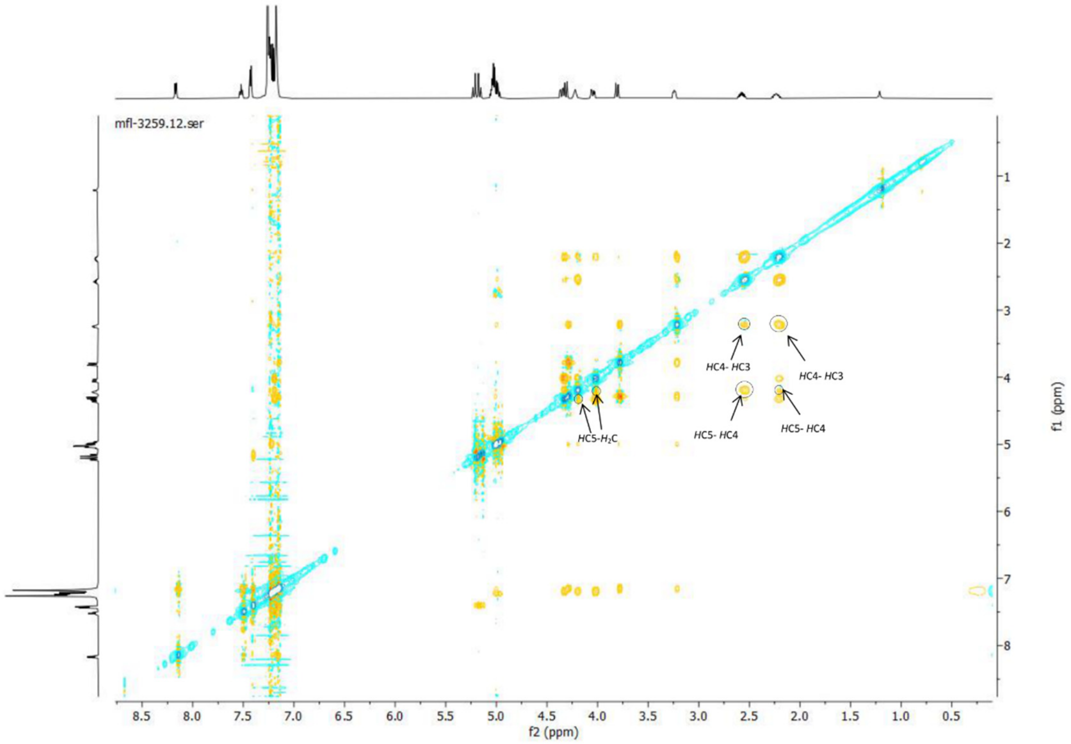

Table S1: ADMET properties in silico for compound *trans*-**16a**

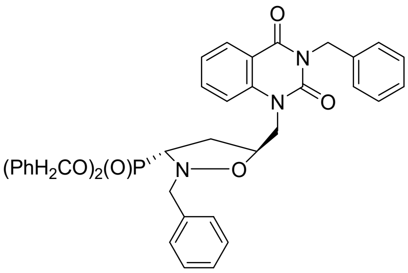

Property	Model Name	Predicted Value	Unit
Absorption	Water solubility	**-4.466**	Numeric (log mol/L)
Absorption	Caco2 permeability	**0.766**	Numeric (log Papp in 10^{-6} cm/s)
Absorption	Intestinal absorption (human)	**100**	Numeric (% Absorbed)
Absorption	Skin Permeability	**-2.735**	Numeric (log Kp)
Absorption	P-glycoprotein substrate	**No**	Categorical (Yes/No)
Absorption	P-glycoprotein I inhibitor	**Yes**	Categorical (Yes/No)
Absorption	P-glycoprotein II inhibitor	**Yes**	Categorical (Yes/No)
Distribution	VDss (human)	**-0.221**	Numeric (log L/kg)
Distribution	Fraction unbound (human)	**0.272**	Numeric (Fu)
Distribution	BBB permeability	**-1.494**	Numeric (log BB)
Distribution	CNS permeability	**-2.441**	Numeric (log PS)

Property	Model Name	Predicted Value	Unit
Metabolism	CYP2D6 substrate	**No**	Categorical (Yes/No)
Metabolism	CYP3A4 substrate	**Yes**	Categorical (Yes/No)
Metabolism	CYP1A2 inhibitior	**No**	Categorical (Yes/No)
Metabolism	CYP2C19 inhibitior	**Yes**	Categorical (Yes/No)
Metabolism	CYP2C9 inhibitior	**Yes**	Categorical (Yes/No)
Metabolism	CYP2D6 inhibitior	**No**	Categorical (Yes/No)
Metabolism	CYP3A4 inhibitior	**No**	Categorical (Yes/No)
Excretion	Total Clearance	**0.447**	Numeric (log ml/min/kg)
Excretion	Renal OCT2 substrate	**No**	Categorical (Yes/No)
Toxicity	AMES toxicity	**No**	Categorical (Yes/No)
Toxicity	Max. tolerated dose (human)	**0.478**	Numeric (log mg/kg/day)
Toxicity	hERG I inhibitor	**No**	Categorical (Yes/No)
Toxicity	hERG II inhibitor	**Yes**	Categorical (Yes/No)
Toxicity	Oral Rat Acute Toxicity (LD50)	**3.387**	Numeric (mol/kg)

Property	Model Name	Predicted Value	Unit
Toxicity	Oral Rat Chronic Toxicity (LOAEL)	**-0.072**	Numeric (log mg/kg_bw/day)
Toxicity	Hepatotoxicity	**Yes**	Categorical (Yes/No)
Toxicity	Skin Sensitisation	**No**	Categorical (Yes/No)
Toxicity	*T.Pyriformis* toxicity	**0.285**	Numeric (log ug/L)
Toxicity	Minnow toxicity	**-6.29**	Numeric (log mM)

Table S2: ADMET properties in silico for compound *cis*-**16a**

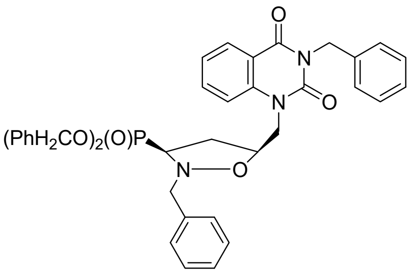

Property	Model Name	Predicted Value	Unit
Absorption	Water solubility	**-4.466**	Numeric (log mol/L)
Absorption	Caco2 permeability	**0.766**	Numeric (log Papp in 10^{-6} cm/s)
Absorption	Intestinal absorption (human)	**100**	Numeric (% Absorbed)

Property	Model Name	Predicted Value	Unit
Absorption	Skin Permeability	**-2.735**	Numeric (log Kp)
Absorption	P-glycoprotein substrate	**No**	Categorical (Yes/No)
Absorption	P-glycoprotein I inhibitor	**Yes**	Categorical (Yes/No)
Absorption	P-glycoprotein II inhibitor	**Yes**	Categorical (Yes/No)
Distribution	VDss (human)	**-0.221**	Numeric (log L/kg)
Distribution	Fraction unbound (human)	**0.272**	Numeric (Fu)
Distribution	BBB permeability	**-1.494**	Numeric (log BB)
Distribution	CNS permeability	**-2.441**	Numeric (log PS)
Metabolism	CYP2D6 substrate	**No**	Categorical (Yes/No)
Metabolism	CYP3A4 substrate	**Yes**	Categorical (Yes/No)
Metabolism	CYP1A2 inhibitior	**No**	Categorical (Yes/No)
Metabolism	CYP2C19 inhibitior	**Yes**	Categorical (Yes/No)
Metabolism	CYP2C9 inhibitior	**Yes**	Categorical (Yes/No)
Metabolism	CYP2D6 inhibitior	**No**	Categorical (Yes/No)
Metabolism	CYP3A4 inhibitior	**No**	Categorical (Yes/No)

Property	Model Name	Predicted Value	Unit
Excretion	Total Clearance	**0.447**	Numeric (log ml/min/kg)
Excretion	Renal OCT2 substrate	**No**	Categorical (Yes/No)
Toxicity	AMES toxicity	**No**	Categorical (Yes/No)
Toxicity	Max. tolerated dose (human)	**0.478**	Numeric (log mg/kg/day)
Toxicity	hERG I inhibitor	**No**	Categorical (Yes/No)
Toxicity	hERG II inhibitor	**Yes**	Categorical (Yes/No)
Toxicity	Oral Rat Acute Toxicity (LD50)	**3.387**	Numeric (mol/kg)
Toxicity	Oral Rat Chronic Toxicity (LOAEL)	**-0.072**	Numeric (log mg/kg_bw/day)
Toxicity	Hepatotoxicity	**Yes**	Categorical (Yes/No)
Toxicity	Skin Sensitisation	**No**	Categorical (Yes/No)
Toxicity	*T.Pyriformis* toxicity	**0.285**	Numeric (log ug/L)
Toxicity	Minnow toxicity	**-6.29**	Numeric (log mM)

Table S3: ADMET properties in silico for compound *trans*-**16b**

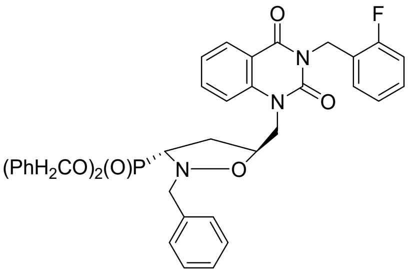

Property	Model Name	Predicted Value	Unit
Absorption	Water solubility	**-4.333**	Numeric (log mol/L)
Absorption	Caco2 permeability	**0.727**	Numeric (log Papp in 10^{-6} cm/s)
Absorption	Intestinal absorption (human)	**100**	Numeric (% Absorbed)
Absorption	Skin Permeability	**-2.735**	Numeric (log Kp)
Absorption	P-glycoprotein substrate	**No**	Categorical (Yes/No)
Absorption	P-glycoprotein I inhibitor	**Yes**	Categorical (Yes/No)
Absorption	P-glycoprotein II inhibitor	**Yes**	Categorical (Yes/No)
Distribution	VDss (human)	**-0.302**	Numeric (log L/kg)
Distribution	Fraction unbound (human)	**0.274**	Numeric (Fu)
Distribution	BBB permeability	**-1.712**	Numeric (log BB)
Distribution	CNS permeability	**-2.557**	Numeric (log PS)
Metabolism	CYP2D6 substrate	**No**	Categorical (Yes/No)

Property	Model Name	Predicted Value	Unit
Metabolism	CYP3A4 substrate	**Yes**	Categorical (Yes/No)
Metabolism	CYP1A2 inhibitior	**No**	Categorical (Yes/No)
Metabolism	CYP2C19 inhibitior	**Yes**	Categorical (Yes/No)
Metabolism	CYP2C9 inhibitior	**Yes**	Categorical (Yes/No)
Metabolism	CYP2D6 inhibitior	**No**	Categorical (Yes/No)
Metabolism	CYP3A4 inhibitior	**No**	Categorical (Yes/No)
Excretion	Total Clearance	**0.419**	Numeric (log ml/min/kg)
Excretion	Renal OCT2 substrate	**No**	Categorical (Yes/No)
Toxicity	AMES toxicity	**No**	Categorical (Yes/No)
Toxicity	Max. tolerated dose (human)	**0.478**	Numeric (log mg/kg/day)
Toxicity	hERG I inhibitor	**No**	Categorical (Yes/No)
Toxicity	hERG II inhibitor	**Yes**	Categorical (Yes/No)
Toxicity	Oral Rat Acute Toxicity (LD50)	**3.358**	Numeric (mol/kg)
Toxicity	Oral Rat Chronic Toxicity (LOAEL)	**-0.261**	Numeric (log mg/kg_bw/day)

Property	Model Name	Predicted Value	Unit
Toxicity	Hepatotoxicity	**Yes**	Categorical (Yes/No)
Toxicity	Skin Sensitisation	**No**	Categorical (Yes/No)
Toxicity	*T.Pyriformis* toxicity	**0.285**	Numeric (log ug/L)
Toxicity	Minnow toxicity	**-6.474**	Numeric (log mM)

Table S4: ADMET properties in silico for compound *cis*-**16b**

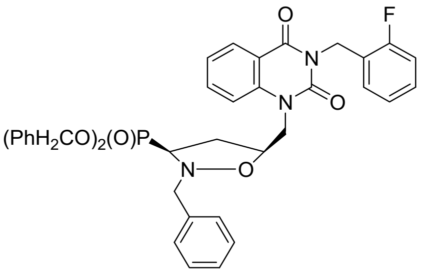

Property	Model Name	Predicted Value	Unit
Absorption	Water solubility	**-4.333**	Numeric (log mol/L)
Absorption	Caco2 permeability	**0.727**	Numeric (log Papp in 10^{-6} cm/s)
Absorption	Intestinal absorption (human)	**100**	Numeric (% Absorbed)
Absorption	Skin Permeability	**-2.735**	Numeric (log Kp)

Property	Model Name	Predicted Value	Unit
Absorption	P-glycoprotein substrate	**No**	Categorical (Yes/No)
Absorption	P-glycoprotein I inhibitor	**Yes**	Categorical (Yes/No)
Absorption	P-glycoprotein II inhibitor	**Yes**	Categorical (Yes/No)
Distribution	VDss (human)	**-0.302**	Numeric (log L/kg)
Distribution	Fraction unbound (human)	**0.274**	Numeric (Fu)
Distribution	BBB permeability	**-1.712**	Numeric (log BB)
Distribution	CNS permeability	**-2.557**	Numeric (log PS)
Metabolism	CYP2D6 substrate	**No**	Categorical (Yes/No)
Metabolism	CYP3A4 substrate	**Yes**	Categorical (Yes/No)
Metabolism	CYP1A2 inhibitior	**No**	Categorical (Yes/No)
Metabolism	CYP2C19 inhibitior	**Yes**	Categorical (Yes/No)
Metabolism	CYP2C9 inhibitior	**Yes**	Categorical (Yes/No)
Metabolism	CYP2D6 inhibitior	**No**	Categorical (Yes/No)
Metabolism	CYP3A4 inhibitior	**No**	Categorical (Yes/No)
Excretion	Total Clearance	**0.419**	Numeric (log ml/min/kg)

Property	Model Name	Predicted Value	Unit
Excretion	Renal OCT2 substrate	**No**	Categorical (Yes/No)
Toxicity	AMES toxicity	**No**	Categorical (Yes/No)
Toxicity	Max. tolerated dose (human)	**0.478**	Numeric (log mg/kg/day)
Toxicity	hERG I inhibitor	**No**	Categorical (Yes/No)
Toxicity	hERG II inhibitor	**Yes**	Categorical (Yes/No)
Toxicity	Oral Rat Acute Toxicity (LD50)	**3.358**	Numeric (mol/kg)
Toxicity	Oral Rat Chronic Toxicity (LOAEL)	**-0.261**	Numeric (log mg/kg_bw/day)
Toxicity	Hepatotoxicity	**Yes**	Categorical (Yes/No)
Toxicity	Skin Sensitisation	**No**	Categorical (Yes/No)
Toxicity	*T.Pyriformis* toxicity	**0.285**	Numeric (log ug/L)
Toxicity	Minnow toxicity	**-6.474**	Numeric (log mM)

Table S5: ADMET properties in silico for compound *trans*-**16c**

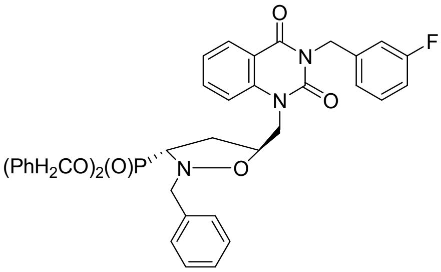

Property	Model Name	Predicted Value	Unit
Absorption	Water solubility	**-4.297**	Numeric (log mol/L)
Absorption	Caco2 permeability	**0.734**	Numeric (log Papp in 10^{-6} cm/s)
Absorption	Intestinal absorption (human)	**100**	Numeric (% Absorbed)
Absorption	Skin Permeability	**-2.735**	Numeric (log Kp)
Absorption	P-glycoprotein substrate	**No**	Categorical (Yes/No)
Absorption	P-glycoprotein I inhibitor	**Yes**	Categorical (Yes/No)
Absorption	P-glycoprotein II inhibitor	**Yes**	Categorical (Yes/No)
Distribution	VDss (human)	**-0.305**	Numeric (log L/kg)
Distribution	Fraction unbound (human)	**0.28**	Numeric (Fu)
Distribution	BBB permeability	**-1.709**	Numeric (log BB)
Distribution	CNS permeability	**-2.566**	Numeric (log PS)
Metabolism	CYP2D6 substrate	**No**	Categorical (Yes/No)

Property	Model Name	Predicted Value	Unit
Metabolism	CYP3A4 substrate	**Yes**	Categorical (Yes/No)
Metabolism	CYP1A2 inhibitior	**No**	Categorical (Yes/No)
Metabolism	CYP2C19 inhibitior	**Yes**	Categorical (Yes/No)
Metabolism	CYP2C9 inhibitior	**Yes**	Categorical (Yes/No)
Metabolism	CYP2D6 inhibitior	**No**	Categorical (Yes/No)
Metabolism	CYP3A4 inhibitior	**No**	Categorical (Yes/No)
Excretion	Total Clearance	**0.304**	Numeric (log ml/min/kg)
Excretion	Renal OCT2 substrate	**No**	Categorical (Yes/No)
Toxicity	AMES toxicity	**No**	Categorical (Yes/No)
Toxicity	Max. tolerated dose (human)	**0.497**	Numeric (log mg/kg/day)
Toxicity	hERG I inhibitor	**No**	Categorical (Yes/No)
Toxicity	hERG II inhibitor	**Yes**	Categorical (Yes/No)
Toxicity	Oral Rat Acute Toxicity (LD50)	**3.354**	Numeric (mol/kg)
Toxicity	Oral Rat Chronic Toxicity (LOAEL)	**-0.329**	Numeric (log mg/kg_bw/day)

Property	Model Name	Predicted Value	Unit
Toxicity	Hepatotoxicity	**Yes**	Categorical (Yes/No)
Toxicity	Skin Sensitisation	**No**	Categorical (Yes/No)
Toxicity	*T.Pyriformis* toxicity	**0.285**	Numeric (log ug/L)
Toxicity	Minnow toxicity	**-6.656**	Numeric (log mM)

Table S6: ADMET properties in silico for compound *cis*-**16c**

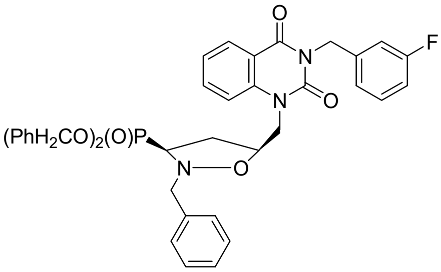

Property	Model Name	Predicted Value	Unit
Absorption	Water solubility	**-4.297**	Numeric (log mol/L)
Absorption	Caco2 permeability	**0.734**	Numeric (log Papp in 10^{-6} cm/s)
Absorption	Intestinal absorption (human)	**100**	Numeric (% Absorbed)
Absorption	Skin Permeability	**-2.735**	Numeric (log Kp)

Property	Model Name	Predicted Value	Unit
Absorption	P-glycoprotein substrate	**No**	Categorical (Yes/No)
Absorption	P-glycoprotein I inhibitor	**Yes**	Categorical (Yes/No)
Absorption	P-glycoprotein II inhibitor	**Yes**	Categorical (Yes/No)
Distribution	VDss (human)	**-0.305**	Numeric (log L/kg)
Distribution	Fraction unbound (human)	**0.28**	Numeric (Fu)
Distribution	BBB permeability	**-1.709**	Numeric (log BB)
Distribution	CNS permeability	**-2.566**	Numeric (log PS)
Metabolism	CYP2D6 substrate	**No**	Categorical (Yes/No)
Metabolism	CYP3A4 substrate	**Yes**	Categorical (Yes/No)
Metabolism	CYP1A2 inhibitior	**No**	Categorical (Yes/No)
Metabolism	CYP2C19 inhibitior	**Yes**	Categorical (Yes/No)
Metabolism	CYP2C9 inhibitior	**Yes**	Categorical (Yes/No)
Metabolism	CYP2D6 inhibitior	**No**	Categorical (Yes/No)
Metabolism	CYP3A4 inhibitior	**No**	Categorical (Yes/No)
Excretion	Total Clearance	**0.304**	Numeric (log ml/min/kg)

Property	Model Name	Predicted Value	Unit
Excretion	Renal OCT2 substrate	**No**	Categorical (Yes/No)
Toxicity	AMES toxicity	**No**	Categorical (Yes/No)
Toxicity	Max. tolerated dose (human)	**0.497**	Numeric (log mg/kg/day)
Toxicity	hERG I inhibitor	**No**	Categorical (Yes/No)
Toxicity	hERG II inhibitor	**Yes**	Categorical (Yes/No)
Toxicity	Oral Rat Acute Toxicity (LD50)	**3.354**	Numeric (mol/kg)
Toxicity	Oral Rat Chronic Toxicity (LOAEL)	**-0.329**	Numeric (log mg/kg_bw/day)
Toxicity	Hepatotoxicity	**Yes**	Categorical (Yes/No)
Toxicity	Skin Sensitisation	**No**	Categorical (Yes/No)
Toxicity	*T.Pyriformis* toxicity	**0.285**	Numeric (log ug/L)
Toxicity	Minnow toxicity	**-6.656**	Numeric (log mM)

Table S7: ADMET properties in silico for compound *trans*-**16d**

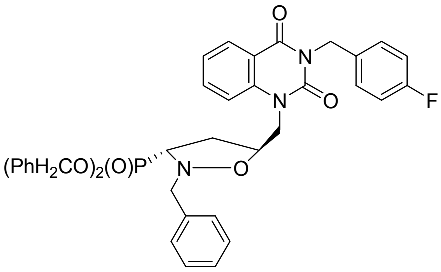

Property	Model Name	Predicted Value	Unit
Absorption	Water solubility	**-4.221**	Numeric (log mol/L)
Absorption	Caco2 permeability	**0.741**	Numeric (log Papp in 10^{-6} cm/s)
Absorption	Intestinal absorption (human)	**100**	Numeric (% Absorbed)
Absorption	Skin Permeability	**-2.735**	Numeric (log Kp)
Absorption	P-glycoprotein substrate	**No**	Categorical (Yes/No)
Absorption	P-glycoprotein I inhibitor	**Yes**	Categorical (Yes/No)
Absorption	P-glycoprotein II inhibitor	**Yes**	Categorical (Yes/No)
Distribution	VDss (human)	**-0.374**	Numeric (log L/kg)
Distribution	Fraction unbound (human)	**0.298**	Numeric (Fu)
Distribution	BBB permeability	**-1.714**	Numeric (log BB)
Distribution	CNS permeability	**-2.575**	Numeric (log PS)
Metabolism	CYP2D6 substrate	**No**	Categorical (Yes/No)

Property	Model Name	Predicted Value	Unit
Metabolism	CYP3A4 substrate	**Yes**	Categorical (Yes/No)
Metabolism	CYP1A2 inhibitior	**No**	Categorical (Yes/No)
Metabolism	CYP2C19 inhibitior	**Yes**	Categorical (Yes/No)
Metabolism	CYP2C9 inhibitior	**Yes**	Categorical (Yes/No)
Metabolism	CYP2D6 inhibitior	**No**	Categorical (Yes/No)
Metabolism	CYP3A4 inhibitior	**No**	Categorical (Yes/No)
Excretion	Total Clearance	**0.303**	Numeric (log ml/min/kg)
Excretion	Renal OCT2 substrate	**No**	Categorical (Yes/No)
Toxicity	AMES toxicity	**No**	Categorical (Yes/No)
Toxicity	Max. tolerated dose (human)	**0.51**	Numeric (log mg/kg/day)
Toxicity	hERG I inhibitor	**No**	Categorical (Yes/No)
Toxicity	hERG II inhibitor	**Yes**	Categorical (Yes/No)
Toxicity	Oral Rat Acute Toxicity (LD50)	**3.34**	Numeric (mol/kg)
Toxicity	Oral Rat Chronic Toxicity (LOAEL)	**-0.419**	Numeric (log mg/kg_bw/day)

Property	Model Name	Predicted Value	Unit
Toxicity	Hepatotoxicity	**Yes**	Categorical (Yes/No)
Toxicity	Skin Sensitisation	**No**	Categorical (Yes/No)
Toxicity	*T.Pyriformis* toxicity	**0.285**	Numeric (log ug/L)
Toxicity	Minnow toxicity	**-6.231**	Numeric (log mM)

Table S8: ADMET properties in silico for compound *cis*-**16d**

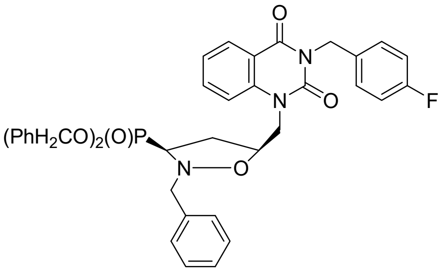

Property	Model Name	Predicted Value	Unit
Absorption	Water solubility	**-4.221**	Numeric (log mol/L)
Absorption	Caco2 permeability	**0.741**	Numeric (log Papp in 10^{-6} cm/s)
Absorption	Intestinal absorption (human)	**100**	Numeric (% Absorbed)
Absorption	Skin Permeability	**-2.735**	Numeric (log Kp)

Property	Model Name	Predicted Value	Unit
Absorption	P-glycoprotein substrate	**No**	Categorical (Yes/No)
Absorption	P-glycoprotein I inhibitor	**Yes**	Categorical (Yes/No)
Absorption	P-glycoprotein II inhibitor	**Yes**	Categorical (Yes/No)
Distribution	VDss (human)	**-0.374**	Numeric (log L/kg)
Distribution	Fraction unbound (human)	**0.298**	Numeric (Fu)
Distribution	BBB permeability	**-1.714**	Numeric (log BB)
Distribution	CNS permeability	**-2.575**	Numeric (log PS)
Metabolism	CYP2D6 substrate	**No**	Categorical (Yes/No)
Metabolism	CYP3A4 substrate	**Yes**	Categorical (Yes/No)
Metabolism	CYP1A2 inhibitior	**No**	Categorical (Yes/No)
Metabolism	CYP2C19 inhibitior	**Yes**	Categorical (Yes/No)
Metabolism	CYP2C9 inhibitior	**Yes**	Categorical (Yes/No)
Metabolism	CYP2D6 inhibitior	**No**	Categorical (Yes/No)

Property	Model Name	Predicted Value	Unit
Metabolism	CYP3A4 inhibitior	**No**	Categorical (Yes/No)
Excretion	Total Clearance	**0.303**	Numeric (log ml/min/kg)
Excretion	Renal OCT2 substrate	**No**	Categorical (Yes/No)
Toxicity	AMES toxicity	**No**	Categorical (Yes/No)
Toxicity	Max. tolerated dose (human)	**0.51**	Numeric (log mg/kg/day)
Toxicity	hERG I inhibitor	**No**	Categorical (Yes/No)
Toxicity	hERG II inhibitor	**Yes**	Categorical (Yes/No)
Toxicity	Oral Rat Acute Toxicity (LD50)	**3.34**	Numeric (mol/kg)
Toxicity	Oral Rat Chronic Toxicity (LOAEL)	**-0.419**	Numeric (log mg/kg_bw/day)
Toxicity	Hepatotoxicity	**Yes**	Categorical (Yes/No)
Toxicity	Skin Sensitisation	**No**	Categorical (Yes/No)
Toxicity	*T.Pyriformis* toxicity	**0.285**	Numeric (log ug/L)
Toxicity	Minnow toxicity	**-6.231**	Numeric (log mM)

Table S9: ADMET properties in silico for compound *trans*-**16e**

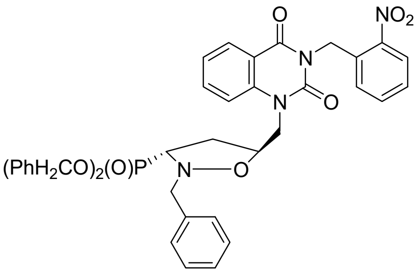

Property	Model Name	Predicted Value	Unit
Absorption	Water solubility	**-3.888**	Numeric (log mol/L)
Absorption	Caco2 permeability	**0.616**	Numeric (log Papp in 10^{-6} cm/s)
Absorption	Intestinal absorption (human)	**100**	Numeric (% Absorbed)
Absorption	Skin Permeability	**-2.735**	Numeric (log Kp)
Absorption	P-glycoprotein substrate	**No**	Categorical (Yes/No)
Absorption	P-glycoprotein I inhibitor	**Yes**	Categorical (Yes/No)
Absorption	P-glycoprotein II inhibitor	**Yes**	Categorical (Yes/No)
Distribution	VDss (human)	**-0.827**	Numeric (log L/kg)
Distribution	Fraction unbound (human)	**0.315**	Numeric (Fu)
Distribution	BBB permeability	**-1.999**	Numeric (log BB)
Distribution	CNS permeability	**-2.615**	Numeric (log PS)

Property	Model Name	Predicted Value	Unit
Metabolism	CYP2D6 substrate	**No**	Categorical (Yes/No)
Metabolism	CYP3A4 substrate	**Yes**	Categorical (Yes/No)
Metabolism	CYP1A2 inhibitior	**No**	Categorical (Yes/No)
Metabolism	CYP2C19 inhibitior	**No**	Categorical (Yes/No)
Metabolism	CYP2C9 inhibitior	**No**	Categorical (Yes/No)
Metabolism	CYP2D6 inhibitior	**No**	Categorical (Yes/No)
Metabolism	CYP3A4 inhibitior	**No**	Categorical (Yes/No)
Excretion	Total Clearance	**0.531**	Numeric (log ml/min/kg)
Excretion	Renal OCT2 substrate	**No**	Categorical (Yes/No)
Toxicity	AMES toxicity	**No**	Categorical (Yes/No)
Toxicity	Max. tolerated dose (human)	**0.428**	Numeric (log mg/kg/day)
Toxicity	hERG I inhibitor	**No**	Categorical (Yes/No)
Toxicity	hERG II inhibitor	**Yes**	Categorical (Yes/No)
Toxicity	Oral Rat Acute Toxicity (LD50)	**2.93**	Numeric (mol/kg)

Property	Model Name	Predicted Value	Unit
Toxicity	Oral Rat Chronic Toxicity (LOAEL)	**-0.325**	Numeric (log mg/kg_bw/day)
Toxicity	Hepatotoxicity	**No**	Categorical (Yes/No)
Toxicity	Skin Sensitisation	**No**	Categorical (Yes/No)
Toxicity	*T.Pyriformis* toxicity	**0.285**	Numeric (log ug/L)
Toxicity	Minnow toxicity	**-6.772**	Numeric (log mM)

Table S10: ADMET properties in silico for compound *cis*-**16e**

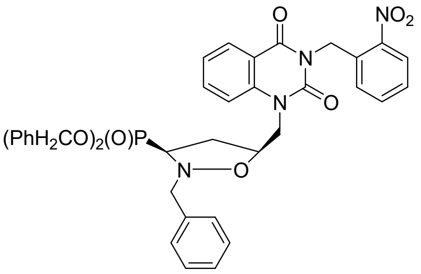

Property	Model Name	Predicted Value	Unit
Absorption	Water solubility	**-3.888**	Numeric (log mol/L)
Absorption	Caco2 permeability	**0.616**	Numeric (log Papp in 10^{-6} cm/s)
Absorption	Intestinal absorption (human)	**100**	Numeric (% Absorbed)

Property	Model Name	Predicted Value	Unit
Absorption	Skin Permeability	**-2.735**	Numeric (log Kp)
Absorption	P-glycoprotein substrate	**No**	Categorical (Yes/No)
Absorption	P-glycoprotein I inhibitor	**Yes**	Categorical (Yes/No)
Absorption	P-glycoprotein II inhibitor	**Yes**	Categorical (Yes/No)
Distribution	VDss (human)	**-0.827**	Numeric (log L/kg)
Distribution	Fraction unbound (human)	**0.315**	Numeric (Fu)
Distribution	BBB permeability	**-1.999**	Numeric (log BB)
Distribution	CNS permeability	**-2.615**	Numeric (log PS)
Metabolism	CYP2D6 substrate	**No**	Categorical (Yes/No)
Metabolism	CYP3A4 substrate	**Yes**	Categorical (Yes/No)
Metabolism	CYP1A2 inhibitior	**No**	Categorical (Yes/No)
Metabolism	CYP2C19 inhibitior	**No**	Categorical (Yes/No)
Metabolism	CYP2C9 inhibitior	**No**	Categorical (Yes/No)
Metabolism	CYP2D6 inhibitior	**No**	Categorical (Yes/No)
Metabolism	CYP3A4 inhibitior	**No**	Categorical (Yes/No)

Property	Model Name	Predicted Value	Unit
Excretion	Total Clearance	**0.531**	Numeric (log ml/min/kg)
Excretion	Renal OCT2 substrate	**No**	Categorical (Yes/No)
Toxicity	AMES toxicity	**No**	Categorical (Yes/No)
Toxicity	Max. tolerated dose (human)	**0.428**	Numeric (log mg/kg/day)
Toxicity	hERG I inhibitor	**No**	Categorical (Yes/No)
Toxicity	hERG II inhibitor	**Yes**	Categorical (Yes/No)
Toxicity	Oral Rat Acute Toxicity (LD50)	**2.93**	Numeric (mol/kg)
Toxicity	Oral Rat Chronic Toxicity (LOAEL)	**-0.325**	Numeric (log mg/kg_bw/day)
Toxicity	Hepatotoxicity	**No**	Categorical (Yes/No)
Toxicity	Skin Sensitisation	**No**	Categorical (Yes/No)
Toxicity	*T.Pyriformis* toxicity	**0.285**	Numeric (log ug/L)
Toxicity	Minnow toxicity	**-6.772**	Numeric (log mM)

Table S11: ADMET properties in silico for compound *trans*-**16f**

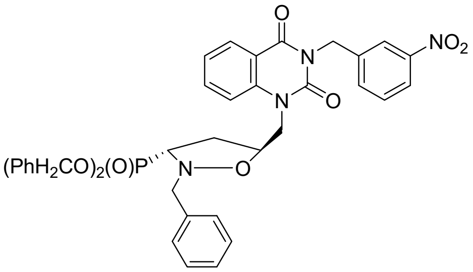

Property	Model Name	Predicted Value	Unit
Absorption	Water solubility	**-3.952**	Numeric (log mol/L)
Absorption	Caco2 permeability	**0.567**	Numeric (log Papp in 10^{-6} cm/s)
Absorption	Intestinal absorption (human)	**100**	Numeric (% Absorbed)
Absorption	Skin Permeability	**-2.735**	Numeric (log Kp)
Absorption	P-glycoprotein substrate	**No**	Categorical (Yes/No)
Absorption	P-glycoprotein I inhibitor	**Yes**	Categorical (Yes/No)
Absorption	P-glycoprotein II inhibitor	**Yes**	Categorical (Yes/No)
Distribution	VDss (human)	**-0.69**	Numeric (log L/kg)
Distribution	Fraction unbound (human)	**0.306**	Numeric (Fu)
Distribution	BBB permeability	**-2.012**	Numeric (log BB)
Distribution	CNS permeability	**-2.602**	Numeric (log PS)
Metabolism	CYP2D6 substrate	**No**	Categorical (Yes/No)

Property	Model Name	Predicted Value	Unit
Metabolism	CYP3A4 substrate	**Yes**	Categorical (Yes/No)
Metabolism	CYP1A2 inhibitior	**No**	Categorical (Yes/No)
Metabolism	CYP2C19 inhibitior	**No**	Categorical (Yes/No)
Metabolism	CYP2C9 inhibitior	**Yes**	Categorical (Yes/No)
Metabolism	CYP2D6 inhibitior	**No**	Categorical (Yes/No)
Metabolism	CYP3A4 inhibitior	**No**	Categorical (Yes/No)
Excretion	Total Clearance	**0.475**	Numeric (log ml/min/kg)
Excretion	Renal OCT2 substrate	**No**	Categorical (Yes/No)
Toxicity	AMES toxicity	**No**	Categorical (Yes/No)
Toxicity	Max. tolerated dose (human)	**0.421**	Numeric (log mg/kg/day)
Toxicity	hERG I inhibitor	**No**	Categorical (Yes/No)
Toxicity	hERG II inhibitor	**Yes**	Categorical (Yes/No)
Toxicity	Oral Rat Acute Toxicity (LD50)	**2.933**	Numeric (mol/kg)
Toxicity	Oral Rat Chronic Toxicity (LOAEL)	**-0.217**	Numeric (log mg/kg_bw/day)

Property	Model Name	Predicted Value	Unit
Toxicity	Hepatotoxicity	**No**	Categorical (Yes/No)
Toxicity	Skin Sensitisation	**No**	Categorical (Yes/No)
Toxicity	*T.Pyriformis* toxicity	**0.285**	Numeric (log ug/L)
Toxicity	Minnow toxicity	**-7.347**	Numeric (log mM)

Table S12: ADMET properties in silico for compound *cis*-**16f**

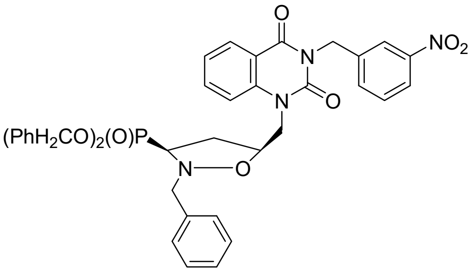

Property	Model Name	Predicted Value	Unit
Absorption	Water solubility	**-3.952**	Numeric (log mol/L)
Absorption	Caco2 permeability	**0.567**	Numeric (log Papp in 10-6 cm/s)
Absorption	Intestinal absorption (human)	**100**	Numeric (% Absorbed)
Absorption	Skin Permeability	**-2.735**	Numeric (log Kp)

Property	Model Name	Predicted Value	Unit
Absorption	P-glycoprotein substrate	**No**	Categorical (Yes/No)
Absorption	P-glycoprotein I inhibitor	**Yes**	Categorical (Yes/No)
Absorption	P-glycoprotein II inhibitor	**Yes**	Categorical (Yes/No)
Distribution	VDss (human)	**-0.69**	Numeric (log L/kg)
Distribution	Fraction unbound (human)	**0.306**	Numeric (Fu)
Distribution	BBB permeability	**-2.012**	Numeric (log BB)
Distribution	CNS permeability	**-2.602**	Numeric (log PS)
Metabolism	CYP2D6 substrate	**No**	Categorical (Yes/No)
Metabolism	CYP3A4 substrate	**Yes**	Categorical (Yes/No)
Metabolism	CYP1A2 inhibitior	**No**	Categorical (Yes/No)
Metabolism	CYP2C19 inhibitior	**No**	Categorical (Yes/No)
Metabolism	CYP2C9 inhibitior	**Yes**	Categorical (Yes/No)
Metabolism	CYP2D6 inhibitior	**No**	Categorical (Yes/No)
Metabolism	CYP3A4 inhibitior	**No**	Categorical (Yes/No)
Excretion	Total Clearance	**0.475**	Numeric (log ml/min/kg)

Property	Model Name	Predicted Value	Unit
Excretion	Renal OCT2 substrate	**No**	Categorical (Yes/No)
Toxicity	AMES toxicity	**No**	Categorical (Yes/No)
Toxicity	Max. tolerated dose (human)	**0.421**	Numeric (log mg/kg/day)
Toxicity	hERG I inhibitor	**No**	Categorical (Yes/No)
Toxicity	hERG II inhibitor	**Yes**	Categorical (Yes/No)
Toxicity	Oral Rat Acute Toxicity (LD50)	**2.933**	Numeric (mol/kg)
Toxicity	Oral Rat Chronic Toxicity (LOAEL)	**-0.217**	Numeric (log mg/kg_bw/day)
Toxicity	Hepatotoxicity	**No**	Categorical (Yes/No)
Toxicity	Skin Sensitisation	**No**	Categorical (Yes/No)
Toxicity	*T.Pyriformis* toxicity	**0.285**	Numeric (log ug/L)
Toxicity	Minnow toxicity	**-7.347**	Numeric (log mM)

Table S13: ADMET properties in silico for compound *trans*-**16g**

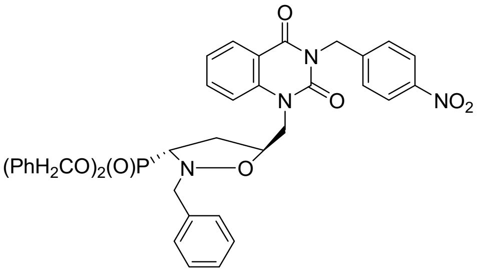

Property	Model Name	Predicted Value	Unit
Absorption	Water solubility	**-3.957**	Numeric (log mol/L)
Absorption	Caco2 permeability	**0.541**	Numeric (log Papp in 10^{-6} cm/s)
Absorption	Intestinal absorption (human)	**100**	Numeric (% Absorbed)
Absorption	Skin Permeability	**-2.735**	Numeric (log Kp)
Absorption	P-glycoprotein substrate	**No**	Categorical (Yes/No)
Absorption	P-glycoprotein I inhibitor	**Yes**	Categorical (Yes/No)
Absorption	P-glycoprotein II inhibitor	**Yes**	Categorical (Yes/No)
Distribution	VDss (human)	**-0.651**	Numeric (log L/kg)
Distribution	Fraction unbound (human)	**0.301**	Numeric (Fu)
Distribution	BBB permeability	**-2.008**	Numeric (log BB)
Distribution	CNS permeability	**-2.607**	Numeric (log PS)
Metabolism	CYP2D6 substrate	**No**	Categorical (Yes/No)

Property	Model Name	Predicted Value	Unit
Metabolism	CYP3A4 substrate	**Yes**	Categorical (Yes/No)
Metabolism	CYP1A2 inhibitior	**No**	Categorical (Yes/No)
Metabolism	CYP2C19 inhibitior	**No**	Categorical (Yes/No)
Metabolism	CYP2C9 inhibitior	**Yes**	Categorical (Yes/No)
Metabolism	CYP2D6 inhibitior	**No**	Categorical (Yes/No)
Metabolism	CYP3A4 inhibitior	**No**	Categorical (Yes/No)
Excretion	Total Clearance	**0.47**	Numeric (log ml/min/kg)
Excretion	Renal OCT2 substrate	**No**	Categorical (Yes/No)
Toxicity	AMES toxicity	**No**	Categorical (Yes/No)
Toxicity	Max. tolerated dose (human)	**0.42**	Numeric (log mg/kg/day)
Toxicity	hERG I inhibitor	**No**	Categorical (Yes/No)
Toxicity	hERG II inhibitor	**Yes**	Categorical (Yes/No)
Toxicity	Oral Rat Acute Toxicity (LD50)	**2.937**	Numeric (mol/kg)
Toxicity	Oral Rat Chronic Toxicity (LOAEL)	**-0.158**	Numeric (log mg/kg_bw/day)

Property	Model Name	Predicted Value	Unit
Toxicity	Hepatotoxicity	**Yes**	Categorical (Yes/No)
Toxicity	Skin Sensitisation	**No**	Categorical (Yes/No)
Toxicity	*T.Pyriformis* toxicity	**0.285**	Numeric (log ug/L)
Toxicity	Minnow toxicity	**-7.013**	Numeric (log mM)

Table S14: ADMET properties in silico for compound *cis*-**16g**

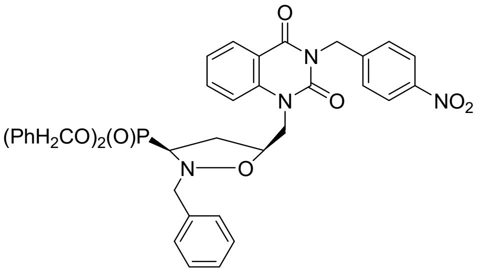

Property	Model Name	Predicted Value	Unit
Absorption	Water solubility	**-3.957**	Numeric (log mol/L)
Absorption	Caco2 permeability	**0.541**	Numeric (log Papp in 10-6 cm/s)
Absorption	Intestinal absorption (human)	**100**	Numeric (% Absorbed)
Absorption	Skin Permeability	**-2.735**	Numeric (log Kp)

Property	Model Name	Predicted Value	Unit
Absorption	P-glycoprotein substrate	**No**	Categorical (Yes/No)
Absorption	P-glycoprotein I inhibitor	**Yes**	Categorical (Yes/No)
Absorption	P-glycoprotein II inhibitor	**Yes**	Categorical (Yes/No)
Distribution	VDss (human)	**-0.651**	Numeric (log L/kg)
Distribution	Fraction unbound (human)	**0.301**	Numeric (Fu)
Distribution	BBB permeability	**-2.008**	Numeric (log BB)
Distribution	CNS permeability	**-2.607**	Numeric (log PS)
Metabolism	CYP2D6 substrate	**No**	Categorical (Yes/No)
Metabolism	CYP3A4 substrate	**Yes**	Categorical (Yes/No)
Metabolism	CYP1A2 inhibitior	**No**	Categorical (Yes/No)
Metabolism	CYP2C19 inhibitior	**No**	Categorical (Yes/No)
Metabolism	CYP2C9 inhibitior	**Yes**	Categorical (Yes/No)
Metabolism	CYP2D6 inhibitior	**No**	Categorical (Yes/No)
Metabolism	CYP3A4 inhibitior	**No**	Categorical (Yes/No)
Excretion	Total Clearance	**0.47**	Numeric (log ml/min/kg)

Property	Model Name	Predicted Value	Unit
Excretion	Renal OCT2 substrate	**No**	Categorical (Yes/No)
Toxicity	AMES toxicity	**No**	Categorical (Yes/No)
Toxicity	Max. tolerated dose (human)	**0.42**	Numeric (log mg/kg/day)
Toxicity	hERG I inhibitor	**No**	Categorical (Yes/No)
Toxicity	hERG II inhibitor	**Yes**	Categorical (Yes/No)
Toxicity	Oral Rat Acute Toxicity (LD50)	**2.937**	Numeric (mol/kg)
Toxicity	Oral Rat Chronic Toxicity (LOAEL)	**-0.158**	Numeric (log mg/kg_bw/day)
Toxicity	Hepatotoxicity	**Yes**	Categorical (Yes/No)
Toxicity	Skin Sensitisation	**No**	Categorical (Yes/No)
Toxicity	*T.Pyriformis* toxicity	**0.285**	Numeric (log ug/L)
Toxicity	Minnow toxicity	**-7.013**	Numeric (log mM)

Table S15: ADMET properties in silico for Doxorubicin

Property	Model Name	Predicted Value	Unit
Absorption	Water solubility	**-3.46**	Numeric (log mol/L)
Absorption	Caco2 permeability	**0.151**	Numeric (log Papp in 10^{-6} cm/s)
Absorption	Intestinal absorption (human)	**74.084**	Numeric (% Absorbed)
Absorption	Skin Permeability	**-2.735**	Numeric (log Kp)
Absorption	P-glycoprotein substrate	**Yes**	Categorical (Yes/No)
Absorption	P-glycoprotein I inhibitor	**No**	Categorical (Yes/No)
Absorption	P-glycoprotein II inhibitor	**No**	Categorical (Yes/No)
Distribution	VDss (human)	**1.145**	Numeric (log L/kg)
Distribution	Fraction unbound (human)	**0.232**	Numeric (Fu)
Distribution	BBB permeability	**-1.635**	Numeric (log BB)
Distribution	CNS permeability	**-4.461**	Numeric (log PS)
Metabolism	CYP2D6 substrate	**No**	Categorical (Yes/No)
Metabolism	CYP3A4 substrate	**No**	Categorical (Yes/No)
Metabolism	CYP1A2 inhibitior	**No**	Categorical (Yes/No)

Property	Model Name	Predicted Value	Unit
Metabolism	CYP2C19 inhibitior	**No**	Categorical (Yes/No)
Metabolism	CYP2C9 inhibitior	**No**	Categorical (Yes/No)
Metabolism	CYP2D6 inhibitior	**No**	Categorical (Yes/No)
Metabolism	CYP3A4 inhibitior	**No**	Categorical (Yes/No)
Excretion	Total Clearance	**0.834**	Numeric (log ml/min/kg)
Excretion	Renal OCT2 substrate	**No**	Categorical (Yes/No)
Toxicity	AMES toxicity	**Yes**	Categorical (Yes/No)
Toxicity	Max. tolerated dose (human)	**0.654**	Numeric (log mg/kg/day)
Toxicity	hERG I inhibitor	**No**	Categorical (Yes/No)
Toxicity	hERG II inhibitor	**No**	Categorical (Yes/No)
Toxicity	Oral Rat Acute Toxicity (LD50)	**3.978**	Numeric (mol/kg)
Toxicity	Oral Rat Chronic Toxicity (LOAEL)	**3.296**	Numeric (log mg/kg_bw/day)
Toxicity	Hepatotoxicity	**No**	Categorical (Yes/No)
Toxicity	Skin Sensitisation	**No**	Categorical (Yes/No)

Property	Model Name	Predicted Value	Unit
Toxicity	*T.Pyriformis* toxicity	**0.285**	Numeric (log ug/L)
Toxicity	Minnow toxicity	**8.359**	Numeric (log mM)
